# Epigenome-wide association study for pesticide (Permethrin and DEET) induced DNA methylation epimutation biomarkers for specific transgenerational disease

**DOI:** 10.1186/s12940-020-00666-y

**Published:** 2020-11-04

**Authors:** Jennifer L. M. Thorson, Daniel Beck, Millissia Ben Maamar, Eric E. Nilsson, Michael K. Skinner

**Affiliations:** Center for Reproductive Biology, School of Biological Sciences, Washington State University, Pullman, WA 99164-4236 USA

**Keywords:** EWAS, Permethrin, N,N-diethyl-meta-toluamide, Transgenerational, Sperm, Testis, Prostate, Kidney, Multiple disease, Pathology

## Abstract

**Background:**

Permethrin and N,N-diethyl-meta-toluamide (DEET) are the pesticides and insect repellent most commonly used by humans. These pesticides have been shown to promote the epigenetic transgenerational inheritance of disease in rats. The current study was designed as an epigenome-wide association study (EWAS) to identify potential sperm DNA methylation epimutation biomarkers for specific transgenerational disease.

**Methods:**

Outbred Sprague Dawley gestating female rats (F0) were transiently exposed during fetal gonadal sex determination to the pesticide combination including Permethrin and DEET. The F3 generation great-grand offspring within the pesticide lineage were aged to 1 year. The transgenerational adult male rat sperm were collected from individuals with single and multiple diseases and compared to non-diseased animals to identify differential DNA methylation regions (DMRs) as biomarkers for specific transgenerational disease.

**Results:**

The exposure of gestating female rats to a permethrin and DEET pesticide combination promoted transgenerational testis disease, prostate disease, kidney disease, and the presence of multiple disease in the subsequent F3 generation great-grand offspring. The disease DMRs were found to be disease specific with negligible overlap between different diseases. The genomic features of CpG density, DMR length, and chromosomal locations of the disease specific DMRs were investigated. Interestingly, the majority of the disease specific sperm DMR associated genes have been previously found to be linked to relevant disease specific genes.

**Conclusions:**

Observations demonstrate the EWAS approach identified disease specific biomarkers that can be potentially used to assess transgenerational disease susceptibility and facilitate the clinical management of environmentally induced pathology.

**Supplementary information:**

**Supplementary information** accompanies this paper at 10.1186/s12940-020-00666-y.

## Background

The human population is nearly universally exposed to pesticides, and these chemicals are often toxic to human health [1]. Billions of pounds of pesticides are applied worldwide on an annual basis, and pesticide poisoning is a common occurrence among agricultural workers [1]. Many commonly used pesticides, such as permethrin and the insect repellent N,N-diethyl-meta-toluamide, DEET, have been deemed safe and relatively benign by the United States Environmental Protection Agency [2, 3]. However, recent research efforts have identified numerous toxicities and health effects resulting from exposure to these environmental chemicals [2, 4–6]. Initial concern regarding permethrin was low due to the fact that it is rapidly metabolized and excreted in urine [4]. However, bioaccumulation of permethrin in both adipose and brain tissue is cause for concern [4]. Permethrin has been established as a neurotoxin [5]. A mixture of DDT and permethrin results in disruption of homeostatic function in hepatocytes, and at high levels, results in inflammation and oxidative stress [6]. The benign status of commonly used pesticides is being challenged [7–9], but the regulatory agencies have not responded by updating guidelines and policies [2].

The toxicological effects of widely used pesticides on mammals are numerous [7]. A recent review [7] supported effects of oxidative stress and toxicity resulting from permethrin exposure. Reproductive malfunctions have also been shown in the offspring of mammals exposed to environmentally-relevant doses of permethrin in their drinking water [10]. The mixture of two common pesticides, permethrin and DEET, is proposed to have a synergistic effect on the health of exposed mammals, leading to enhanced neurotoxicity, increased mortality, and behavioral alterations [11]. This combination of these pesticides have also been suggested as a possible cause of Gulf War Illness (GWI) among humans [12]. Symptoms related to GWI, most significantly neurodegeration, have been observed in rodent models exposed to these pesticides [13–19]. An extensive review of the chronic exposure to pesticides experienced by humans shows increased risk of chronic diseases, reproductive abnormalities, and birth defects, which are all linked to genetic, epigenetic, and other biochemical alterations [12].

The exposure of human and animal populations to pesticide chemicals results in health problems in both the exposed individuals and in future generations [20]. A multigenerational exposure occurs on an individual and their germ line, and can also include exposure of a pregnant female and the developing fetus. The developing fetus is particularly susceptible to developmental perturbations which can be caused by exposure to environmental toxicants [21–23]. This is well established in animal populations, but recent investigation into human populations has shown the same phenomenon [12]. The developing fetus and infant are directly exposed to pesticides, which enter the placenta and are found in breast milk [21]. Decreased birth weight and length are found among minority women, a demographic known to experience more pesticide exposure [22]. In addition, there is an association among childhood leukemia incidence and parents who are occupationally exposed to pesticides, such as agricultural workers [23].

Epigenetic alterations, rather than de novo genetic alterations, are a likely mechanism of inheritance for these increased incidences of disease among mammalian and human populations [24, 25]. The mechanisms of inheritance for the increased incidence of disease in the human populations likely involves epigenetic inheritance. Lind, et al [26]. demonstrate an association between persistent organic pollutants and DNA hypermethylation in the exposed individual. The direct exposure to pesticides is more prevalent among some populations, particularly agricultural laborers. Among such a population, increased methylation in or near 72 genes was found as a result of exposure to pesticides, mostly involving immune response genes [27]. Prenatal exposure to persistent pollutants yields hypomethylation in fetal blood [28]. Both hyper- and hypomethylation can have detrimental effects on gene expression, and this has greater impact early in development. Alterations in DNA methylation can also be passed on to future generations and lead to an increased incidence of disease [20, 29]. A large number of studies have demonstrated the cumulative effects of a wide variety of endocrine disrupting chemicals, prominent among them many pesticides. The long-lasting effect of these exposures is proposed to persist through epigenetic alterations which include DNA methylation, histone alterations, and ncRNA modifications [30, 31]. These epigenetic alterations can impact the germline, so can be inherited in subsequent generations. An epigenetic transgenerational inheritance effect is established when an individual has experienced no direct exposure to a toxicant and still shows increased incidence of disease due to ancestral exposure [20]. The multigenerational and transgenerational inheritance of epigenetic alterations in response to exposure to toxicants such as pesticides increases the risk of disease in later generations. The lifelong and multigenerational effects of pesticide chemicals on the human population are numerous and include increased incidence of neurotoxicity [32]. Therefore, it is crucial to establish the extent of the effects caused by widely used pesticides such as permethrin and DEET.

Transgenerational inheritance of both epigenetic alterations and increased incidence of disease from the ancestral exposure to the pesticide mixture of permethrin and DEET have been demonstrated in rats [33]. A significant increase in total disease incidence, pubertal abnormalities, testis disease and ovarian disease was found in the F3 generation (transgenerational) of ancestrally exposed individuals [33]. Significant DNA methylation alterations were also observed in the sperm of the pesticide lineage animals compared to control animals [33]. Similar epigenetic transgenerational inheritance of sperm epigenetic alterations and associated increased disease incidence have been demonstrated as a result of ancestral exposure to the fungicide vinclozolin [34], the pesticide DDT [35] the herbicide glyphosate [36] and herbicide atrazine [37].

The association between altered epigenetics and increased disease incidence has been established in many species [20]. The epigenetic alterations induced by exposure to environmental toxicants is not only a mechanism for long-term multigenerational direct exposure effects, but also promote detrimental transgenerational health effects. The epigenetic alterations can potentially serve as biomarkers of the susceptibility of adverse health prospects for the directly exposed individuals and their progeny [20, 30]. Transgenerational epigenetic alterations associated with increased incidence of disease can possibly be used as biomarkers for ancestral exposure and disease [38] and have been established for multiple widespread and persistent environmental toxicants [37, 39, 40]. The current study was designed to investigate the incidence of disease and inherited differential methylation of DNA in the transgenerational F3 generation (great-grand offspring) of rats whose ancestors were exposed to the pesticide mixture of permethrin and DEET [33] in order to identify epigenetic biomarkers of differential DNA methylation regions (DMRs) for specific disease using an epigenome-wide association study (EWAS) approach.

## Methods

### Animal studies and breeding

As previously described [34, 41], female and male rats of an outbred strain Hsd:Sprague Dawley SD (Harlan) at 70 to 100 days of age were fed ad lib with a standard rat diet and ad lib tap water. Timed-pregnant females (*n* = 4) were mated and on days 8 through 14 of gestation [42] were administered daily intraperitoneal injections of pesticides permethrin (150 mg/kg BW/day dissolved in DMSO) and DEET (40 mg/kg BW/day dissolved in DMSO) (Chem Service, Westchester PA) or vehicle control dimethyl sulfoxide (DMSO), as previously described [38]. The transient exposure was during gestation at embryonic day 8–14 (E8-E14) associated with gonadal sex determination and epigenetic programming of the germline.

The gestating female rats treated were designated as the F0 generation. At each generation approximately 5 different litters were generated and culled to 10 pups to reduce litter bias. No sibling or cousin breeding was used to avoid inbreeding artifacts. The F1- F3 generation control and pesticide lineages were housed in the same room and racks with lighting, food and water as previously described [38, 42, 43]. Non-littermate females and males aged 70–100 days from the F1 generation of pesticides or control lineages were bred to others within their treatment group to obtain F2 generation offspring. Un-related F2 generation rats were bred to obtain F3 generation offspring. Only the F0 generation received treatments. All experimental protocols for the procedures with rats were pre-approved by the Washington State University Animal Care and Use Committee (IACUC approval # 2568). All methods were performed in accordance with the relevant guidelines and regulations. The sperm samples and tissue sections from the previous study [44] were archived and used for the current study with new histological and molecular analysis.

#### Tissue harvest and histology processing

As previously described [44], at 12 months of age, rats were euthanized by CO_2_ inhalation and cervical dislocation for tissue harvest. Testis, prostate, and kidney were fixed in Bouin’s solution (Sigma) followed by 70% ethanol, then processed for paraffin embedding, and hematoxylin and eosin (H & E) staining by standard procedures for histopathological examination. Paraffin five microns sections were processed, stained, and processed by Nationwide Histology, Spokane WA, USA.

#### Histopathology examination and disease classification

Archived histology slides or paraffin blocks from the previous study [44] were used for a new histology analysis for the current study. Stained testis, prostate, and kidney slides were imaged through a microscope using 4x objective lenses (testis and prostate) or 10x objective lenses (kidney). Tiled images were captured using a digital camera. Tiled images for each tissue were photo-merged into a single image using Adobe Photoshop (ver. 21.1.2, Adobe, Inc.). Images were evaluated and pathology features digitally marked using Photoshop software. The Washington Animal Disease Diagnostic Laboratory (WADDL) at the Washington State University College of Veterinary Medicine has board certified veterinary pathologists, and assisted in initially establishing the criteria for the pathology analyses and identifying parameters to assess [45]. The tissues evaluated histologically were selected from previous literature showing them to have pathology in transgenerational models [29, 33, 37, 44, 46–50], with an emphasis on reproductive organs. Histopathology readers were trained to recognize the specific abnormalities evaluated for this study in rat testis, ventral prostate and kidney. Two individuals blinded to the exposure evaluated each tissue image for abnormalities. If there was disagreement about the disease status, then a third individual blinded to the exposure evaluated the tissue. Sets of quality control (QC) slides were generated for each tissue, and were read by each reader prior to evaluating any set of experimental slides. These QC slide results were monitored for reader accuracy and concordance.

As previously described [20], testis histopathology criteria included the presence of vacuoles in the seminiferous tubules, azoospermic atretic seminiferous tubules, and ‘other’ abnormalities including sloughed spermatogenic cells in center of the tubule and a lack of a tubule lumen. As previously described [51, 52], prostate histopathology criteria included the presence of vacuoles in the glandular epithelium, atrophic glandular epithelium and hyperplasia of prostatic gland epithelium. Kidney histopathology criteria included reduced size of glomerulus, thickened Bowman’s capsule, and the presence of proteinaceous fluid-filled cysts > 50 μm in diameter. A cutoff was established to declare a tissue ‘diseased’ based on the mean number of histopathological abnormalities plus two standard deviations from the mean of control group tissues, as assessed by each of the individual observers blinded to the treatment groups. This number (i.e. greater than two standard deviations) was used to classify rats into those with and without testis, prostate, or kidney disease in each lineage. A rat tissue section was finally declared ‘diseased’ only when at least two of the three observers marked the same tissue section ‘diseased’. Onset of puberty was assessed in males starting at 35 days of age by the presence of balano-preputial separation. Obesity was assessed with an increase in body mass and a qualitative evaluation of abdominal adiposity, as previously described [33, 47, 53–55]. The statistical analyses for pathology results were expressed as the proportion of affected animals that exceeded a pre-determined threshold (testis, prostate or kidney disease frequency, tumor frequency, obese frequency). Groups were analyzed using Fisher’s exact test.

#### Epididymal sperm collection and DNA isolation

The protocol is described in detail in reference [44]. Briefly, the epididymis was dissected free of fat and connective tissue, then, after cutting open the cauda, placed into 6 ml of phosphate buffer saline (PBS) for 20 min at room temperature. Further incubation at 4 °C will immobilize the sperm. The tissue was then minced, the released sperm pelleted at 4 °C 3000 x *g* for 10 min, then resuspended in NIM buffer and stored at − 80 °C for further processing. An appropriate amount of rat sperm suspension was used for DNA extraction. Previous studies have shown mammalian sperm heads are resistant to sonication unlike somatic cells [56, 57]. Somatic cells and debris were therefore removed by brief sonication (Fisher Sonic Dismembrator, model 300, power 25), then centrifugation and washing 1–2 times in 1X PBS. The resulting pellet was resuspended in 820 μL DNA extraction buffer and 80 μl 0.1 M DTT added, then incubated at 65 °C for 15 min. Eighty microliter proteinase K (20 mg/ml) was added and the sample was incubated at 55 °C for 2–3 h under constant rotation. Protein was removed by addition of protein precipitation solution (300 μl, Promega A795A), incubation for 15 min on ice, then centrifugation at 13,500 rpm for 30 min at 4 °C. One ml of the supernatant was precipitated with 2 μl of Glycoblue (Invitrogen, AM9516) and 1 ml of cold 100% isopropanol. After incubation, the sample was spun at 13,500 x *g* for 30 min at 4 °C, then washed with 70% cold ethanol. The pellet was air-dried for about 5 min then resuspended in 100 μl of nuclease free water.

#### Methylated DNA Immunoprecipitation (MeDIP)

The archived sperm samples were prepped as previously described [44]. Genomic DNA was sonicated and run on 1.5% agarose gel for fragment size verification. The sonicated DNA was then diluted with 1X TE buffer to 400 μl, then heat-denatured for 10 min at 95 °C, and immediately cooled on ice for 10 min to create single-stranded DNA fragments. Then 100 μl of 5X IP buffer and 5 μg of antibody (monoclonal mouse anti 5-methyl cytidine; Diagenode #C15200006) were added, and the mixture was incubated overnight on a rotator at 4 °C. The following day magnetic beads (Dynabeads M280 Sheep anti-Mouse IgG; Life Technologies 11201D) were pre-washed per manufacturer’s instructions, and 50 μl of beads were added to the 500 μl of DNA-antibody mixture from the overnight incubation, then incubated for 2 h on a rotator at 4 °C. After this incubation, the samples were washed three times with 1X IP buffer using a magnetic rack. The washed samples were then resuspended in 250 μl digestion buffer (5 mM Tris PH 8, 10.mM EDTA, 0.5% SDS) with 3.5 μl Proteinase K (20 mg/ml), and incubated for 2–3 h on a rotator at 55 °C. DNA clean-up was performed using a Phenol-Chloroform-Isoamyl-Alcohol extraction, and the supernatant precipitated with 2 μl of Glycoblue (20 mg/ml), 20 μl of 5 M NaCl and 500 μl ethanol in − 20 °C freezer for one to several hours. The DNA precipitate was pelleted, washed with 70% ethanol, then dried and resuspended in 20 μl H_2_O or 1X TE. DNA concentration was measured in Qubit (Life Technologies) with the ssDNA kit (Molecular Probes Q10212).

#### MeDIP-Seq analysis

As previously described [31], MeDIP DNA was used to create libraries for next generation sequencing (NGS) using the NEBNext Ultra RNA Library Prep Kit for Illumina (San Diego, CA) starting at step 1.4 of the manufacturer’s protocol to generate double stranded DNA from the single-stranded DNA resulting from MeDIP. After this step, the manufacturer’s protocol was followed indexing each sample individually with NEBNext Multiplex Oligos for Illumina. The WSU Spokane Genomics Core sequenced the samples on the Illumina HiSeq 2500 at PE50, with a read size of approximately 50 bp and approximately 10–20 million reads per pool. Twelve libraries were run in one lane.

#### Statistics and bioinformatics

The DMR identification and annotation methods follow those presented in previous published papers [31, 37]. Data quality was assessed using the FastQC program (https://www.bioinformatics.babraham.ac.uk/projects/fastqc/). The data was cleaned and filtered to remove adapters and low-quality bases using Trimmomatic [58]. The reads for each MeDIP sample were mapped to the Rnor 6.0 rat genome using Bowtie2 [59] with default parameter options. The mapped read files were then converted to sorted BAM files using SAMtools [60]. The MEDIPS R package [61] was used to calculate differential coverage between disease and non-disease sample groups. The edgeR *p*-value [62] was used to determine the relative difference between the two groups for each genomic window. Windows with an edgeR *p*-value less than an arbitrarily selected threshold were considered DMR. The site edges were extended until no genomic window with an edgeR p-value less than 0.1 remained within 1000 bp of the DMR. The edgeR p-value was used to assess the significance of the DMR identified. A false discovery rate (FDR) analysis for each comparison was performed and provided a *p* < 0.05 for the multiple disease DMRs and a *p* < 0.1 for 162 kidney disease DMRs. The remaining analysis had an FDR *p* > 0.1. Due to the lower number of individuals with one specific disease type, an FDR analysis was generally not useful, nor was a permutation analysis, for the specific disease biomarkers [63–68]. Differential epimutation sites were annotated using the biomaRt R package [69] to access the Ensembl database [70]. The DMR associated genes were then automatically sorted into functional groups using information provided by the DAVID [71] and Panther [72] databases incorporated into an internal curated database (www.skinner.wsu.edu under genomic data). A Pathway Studio, Elsevier, database and network tool was used to assess physiological and disease process gene correlations. All molecular data has been deposited into the public database at NCBI (GEO # GSE158254) and R code computational tools available at GitHub (https://github.com/skinnerlab/MeDIP-seq) and www.skinner.wsu.edu.

## Results

As previously described [33], outbred Sprague Dawley gestating female rats (F0 generation) received a transient exposure during the developmental period of fetal gonadal sex determination to permethrin (150 mg/kg bodyweight/day) and DEET (40 mg/kg bodyweight/day). The no observable adverse effect level (NOAEL) for long-term exposure to permethrin in rats ranges from 100 to 500 mg/kg/day, while there has been no lowest observable adverse effect level established in humans [reviewed in [73]]. The NOAEL for exposure to DEET is 200 mg/kg/day [13]. These relatively low doses, that are below the NOAEL, were administered during embryonic days 8–14 (E8-E14) at the time of fetal gonadal sex determination. The F1 generation offspring were directly exposed as a fetus, and F2 generation grand-offspring exposed as the germline in the F1 generation. These were each bred at 90 days of age within the exposure lineage. Approximately five litters were generated at each generation with no sibling or cousin breeding to avoid any inbreeding artifacts [33]. The F3 generation great-grand-offspring is required to establish the transgenerational inheritance generation of ancestral exposure. A control lineage was established that used F0 gestating rats exposed to the vehicle control dimethyl sulfoxide (DMSO). The archived sperm samples and histology paraffin blocks from the initial study [33] were reanalyzed for the current study. Disease pathology was evaluated in pesticide exposure and control lineages at 1 year of age. The pesticides exposure lineage transgenerational individuals with specific disease or pathology were grouped as representatives of the pathology exhibited. The remaining individuals with no disease were grouped as “no disease.” Comparisons between these two groups were made for analysis of sperm DNA methylation (i.e. methylome). The differential DNA methylation regions (DMRs) in sperm used a methylated DNA immunoprecipitation (MeDIP) followed by next generation sequencing (Seq) of an MeDIP-Seq analysis for an EWAS approach for the identification of specific disease associated epigenetic biomarkers.

As previously described [33] in the Methods, pathology analysis was assessed by examining sections of male testis, kidney, and prostate. The new histological sections obtained from archived paraffin blocks and slides [33] were captured and analyzed digitally for greater accuracy. The digital images were analyzed by two different observers blinded to the exposure. When discrepancies arose, a third counter blinded to the exposure analyzed the histological section. The raw count of abnormalities was obtained from each digital image. The pixel area was also determined from each image, allowing for a size correction between individual organ samples in the study. The final counts used for the analysis represent the number of abnormalities seen per mm^2^ for each individual image. The pathology parameters identified are as previously described in the Methods [33]. In brief, each counter records the incidence of abnormalities in each tissue. In testis, atrophy of a seminiferous tubule, the arrest of maturation of sperm (indicated by sloughed cells in the center of the tubule), and the presence of vacuoles were indicated disease pathologies. The abnormalities counted in kidney include a reduction in size of glomeruli, a thickening of the Bowman’s capsule, and the presence of cysts. Prostate abnormalities counted included atrophy of the epithelial cells, hyperplasia in the epithelial layer, and the presence of vacuoles within the epithelial layer of the prostatic glands. Obese and lean phenotypes were assigned following assessment of body and abdominal adiposity. The individual F3 generation animals are listed as (+) which indicates presence of disease or (−) the absence of disease for the current F3 generation pesticide lineage male pathology (Table 1). Only those individuals with a single disease for a specific pathology were used for that pathology molecular analysis to avoid any comorbidity effects and variables in the analysis. Animals exhibiting more than one disease are all listed under the category “Multiple Disease.” Those animals used in the molecular analysis are indicated by gray highlights in Table 1. Due to low prevalence of disease in the control animal groups, control lineage animals were not used in the identification of epigenetic biomarkers.

**Table 1:** F3 generation pesticides lineage males pathology. The individual animals for the pesticides lineage males are listed and a (+) indicates presence of disease and (−) absence of disease. The shaded disease identifies those animals used for that disease molecular analysis. The number of animals with disease versus total number of animals and percentage is presented. The comparison no disease animals are indicated with a (0)

Molecular ID	Late Puberty	Testis	Prostate	Kidney	Obesity	Tumor	Multiple Disease	Total Disease
PS1	–	–	–	–	–	–	–	0
PS2	–	–	–	–	–	–	–	0
PS3	–	+	–	–	–	–	–	1
PS4	–	+	–	–	–	–	–	1
PS5	–	–	–	–	–	–	–	0
PS6	–	–	–	–	–	–	–	0
PS7	–	–	–	–	–	–	–	0
PS8	–	–	–	–	–	–	–	0
PS9	–	+	–	–	–	–	–	1
PS10	–	–	–	–	–	–	–	0
PS11	–	–	–	–	–	–	–	0
PS12	–	–	–	–	–	–	–	0
PS13	–	–	–	–	–	–	–	0
PS14	–	+	–	+	–	–	+	2
PS15	–	–	–	–	–	–	–	0
PS16	–	–	–	+	–	–	–	1
PS17	–	n/a	+	–	–	–	–	1
PS18	–	–	–	–	–	–	–	0
PS19	–	–	+	–	–	–	–	1
PS20	–	+	+	+	+	–	+	4
PS21	–	–	–	–	–	–	–	0
PS22	–	+	+	–	–	–	+	2
PS23	–	–	–	+	–	–	–	1
PS24	–	–	–	–	–	–	–	0
PS25	–	–	–	+	–	–	–	1
PS26	–	–	+	–	–	–	–	1
PS27	+	+	–	+	+	–	+	4
PS28	–	–	–	+	–	–	–	1
PS29	–	+	–	–	+	–	+	2
PS30	–	+	–	+	–	–	+	2
PS31	–	–	–	+	–	–	–	1
PS32	+	+	–	–	–	–	+	2
PS33	+	–	–	–	–	–	–	1
PS34	+	–	–	+	–	–	+	2
PS35	n/a	+	+	–	–	–	+	2
PS36	n/a	+	–	+	–	–	+	2
PS37	n/a	+	+	+	–	–	+	3
PS38	n/a	+	–	+	–	–	+	2
PS39	–	–	–	–	–	+	–	1
PS40	–	–	+	–	–	–	–	1
PS41	–	–	–	+	–	–	–	1
**Totals**	4/37 = 11%	14/40 = 35%	8/41 = 20%	14/41 = 34%	3/41 = 7%	1/41 = 2%	12/41 = 29%	

**Fig. 1:**
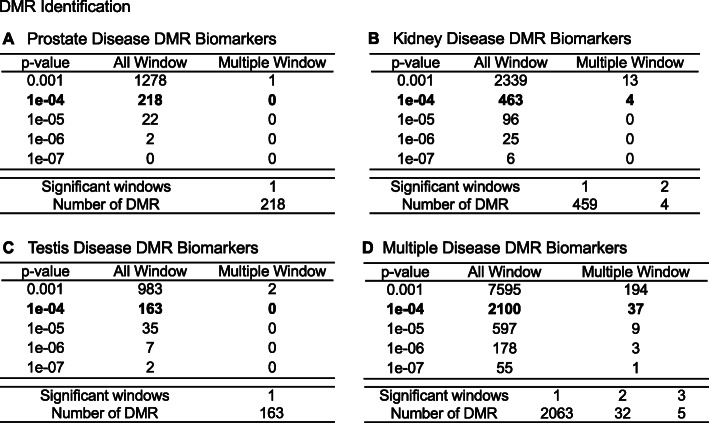
DMR identification and numbers. The number of DMRs found using different p-value cutoff thresholds. The All Window column shows all DMRs. The Multiple Window column shows the number of DMRs containing at least two significant windows (1000 bp each). The number of DMRs with the number of significant windows (1000 bp per window) at a p-value threshold of *p* < 1e-04 for DMR is presented. **a** Prostate disease DMRs; **b** Kidney disease DMRs; **c** Testes disease DMRs; and **d** Multiple disease DMRs

The experimental design was focused on the identification of transgenerational DMRs in sperm. Sperm collected and archived from the pesticide lineage F3 generation males [33] were used for epigenetic analysis. DNA from the sperm was isolated and fragmented with sonication, as described in the Methods. The methylated DNA immunoprecipitation (MeDIP) using a methyl-cytosine antibody was used to identify alterations in DNA methylation. The methylated DNA fragments were then sequenced for an MeDIP-Seq analysis, as described in the Methods [34, 40]. The differential DNA methylation regions (DMRs) were identified between the disease versus non-disease within the pesticides lineage animals (Fig. 1). The transgenerational sperm DMR numbers are presented in Fig. 1 for different edgeR statistical *p*-value cutoff thresholds, and *p* < 1e− 04 (diseased versus non-diseased) for the pesticide lineage were selected as the threshold for subsequent analyses. Disease-specific DMRs were identified among the pesticides treated animals as shown in Table 1. The pesticides lineage animals disease included prostate disease, kidney disease, testis disease and multiple diseases (Fig. 1a-d). The all windows column represents all DMR windows, and multiple window column includes those with neighboring 1 kb sites. Only kidney disease and multiple disease DMRs had multiple adjacent sites. In the current analysis, 1000 bp windows were used in the identification of DMRs.

The previous reported control versus exposure disease associated DMRs [33] were a result of pooled sample analyses, so it was not feasible to identify individual sites as epigenetic biomarkers for disease. In the current study, the MeDIP-seq analysis was performed on individual animal samples allowing for the identification of specific epigenetic biomarkers shared among all pesticide lineage animals with a specific disease. Analysis revealed 463 DMRs associated with kidney disease among which 197 (43%) show an increase in DNA methylation. There were 218 DMRs associated with prostate disease and 102 (47%) show an increase in DNA methylation. There were 163 DMRs associated with testis disease and 52 (32%) show an increase in DNA methylation. The multiple disease group showed 2100 unique DMRs that have 1045 (50%) with an increase in DNA methylation. The specific disease DMR lists and log-fold change information are presented in Supplemental Tables S1-S4.

**Fig. 2:**
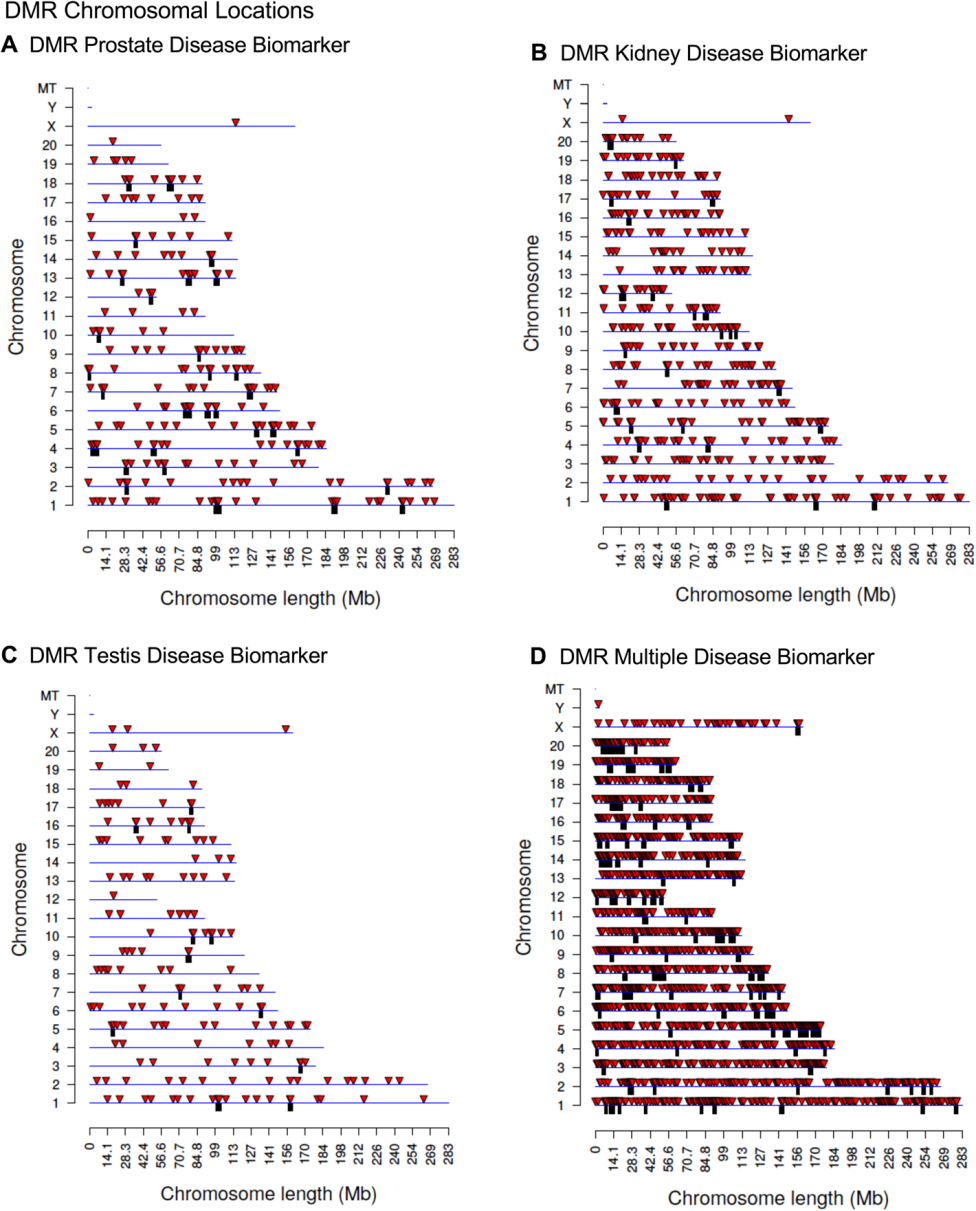
DMR chromosomal locations. The DMR locations on the individual chromosomes is represented with an arrowhead and a cluster of DMRs with a black box. All DMRs containing at least one significant window at a p-value threshold of 1e-04 for DMR are shown. **a** Prostate disease DMRs; **b** Kidney disease DMRs; **c** Testes disease DMRs; and **d** Multiple disease DMRs

**Fig. 3:**
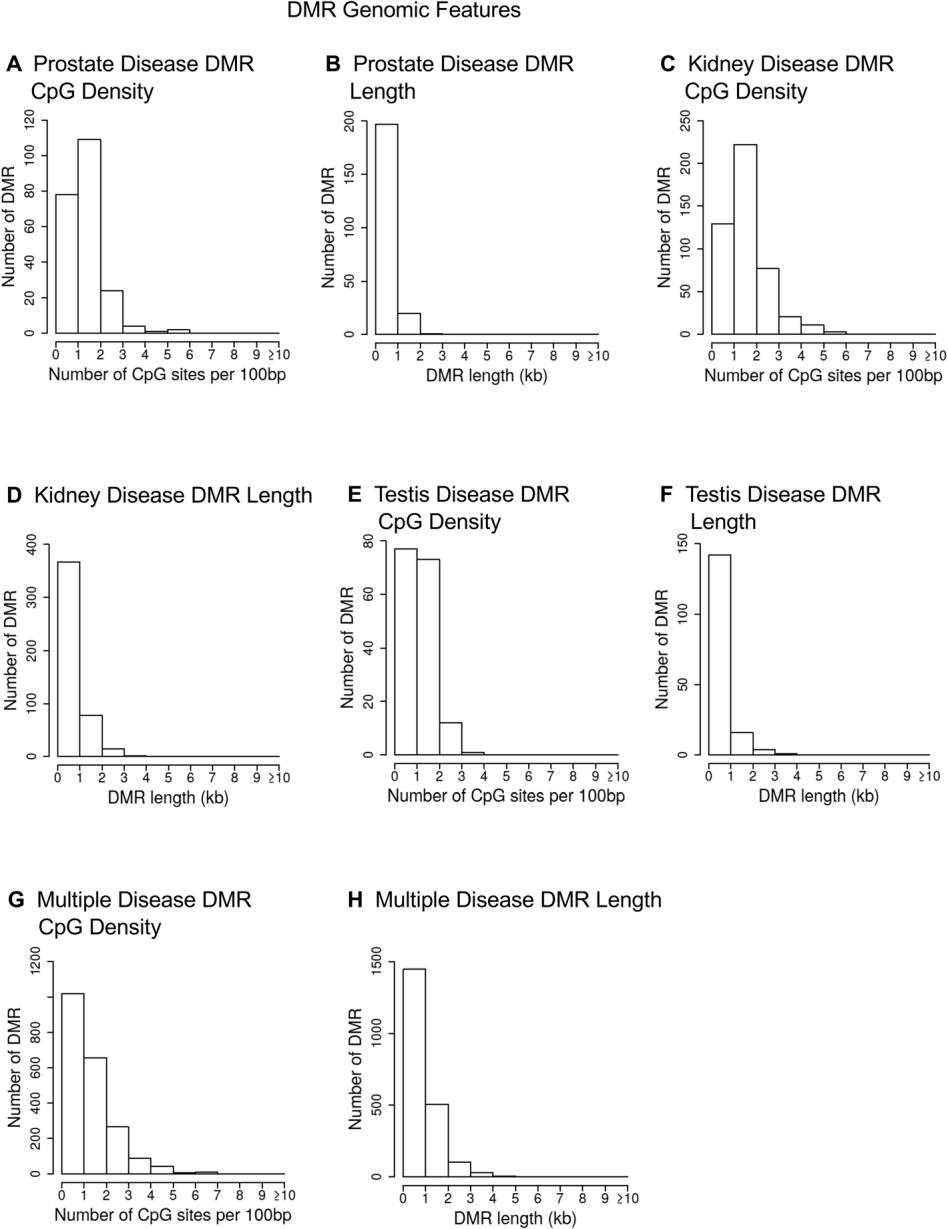
DMR genomic features. The number of DMRs at different CpG densities and length (kb). All DMRs at a p-value threshold of 1e-04 are shown. **a** Prostate disease DMR CpG density; **b** Prostate disease DMR length; **c** Kidney disease DMR CpG density; **d** Kidney disease DMR length; **e** Testes disease DMR CpG density; **f** Testes disease DMR length; **g** Multiple disease DMR CpG density; and **h** Multiple disease DMR length

**Fig. 4:**
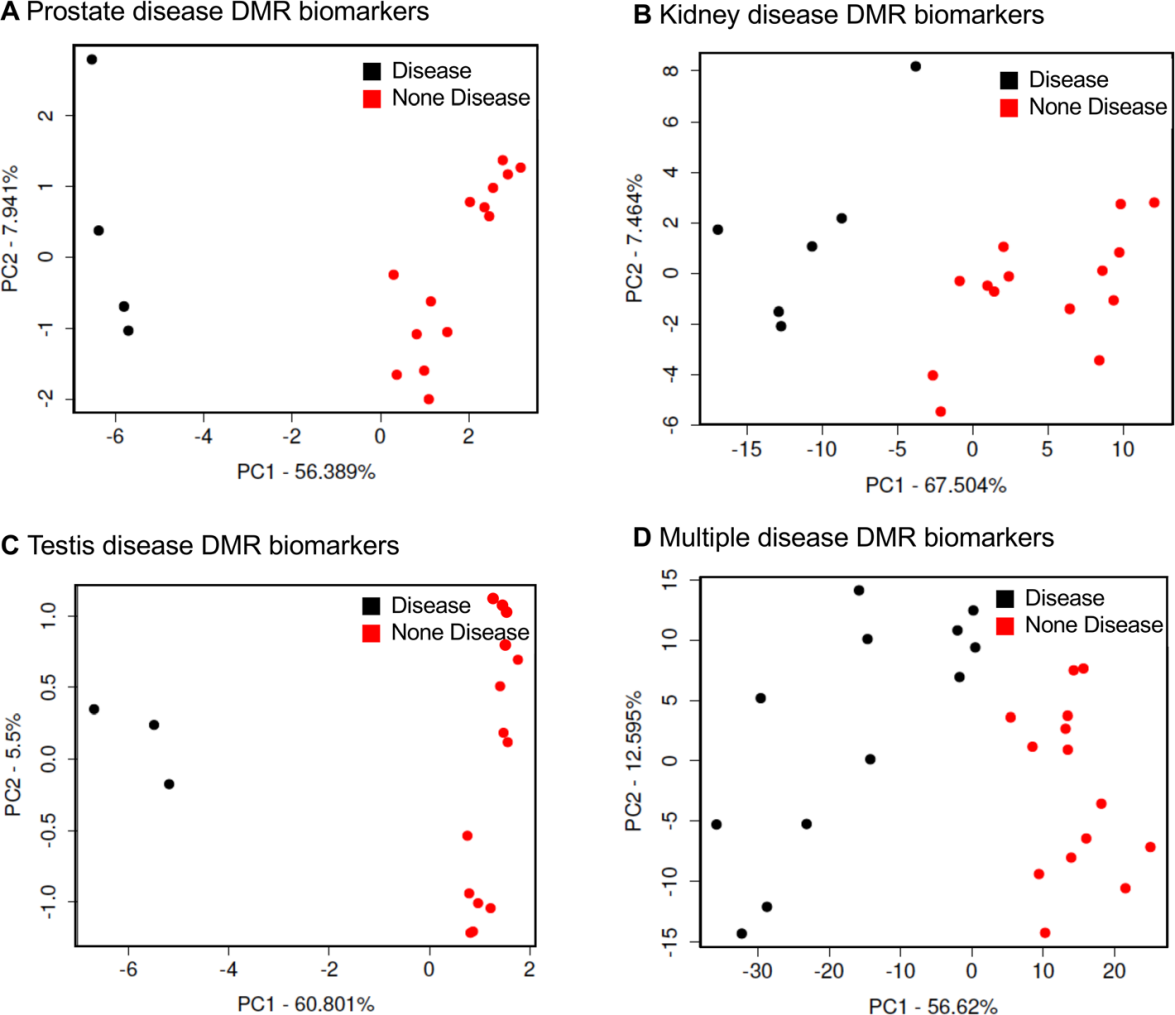
DMR principal component analysis. The first two principal components used. The underlying data is the RPKM read depth for DMR genomic windows. **a** Prostate disease DMRs PCA; **b** Kidney disease DMRs PCA; **c** Testes disease DMRs PCA; and **d** Multiple disease DMRs PCA

Chromosomal locations of the disease associated DMRs are shown in Fig. 2. DMRs are represented as arrowheads and DMR clusters are represented by black boxes. DMRs are present for each individual disease on all chromosomes, except the Y chromosome and mitochondrial DNA (MT). Interestingly, the multiple disease signatures are present on the Y chromosome, as well as all other chromosomes. These results support the idea that the transgenerational epigenetic effects of ancestral pesticides exposure are genome-wide. Genomic features are shown in Fig. 3. The CpG density of DMRs is low, 1–3 CpG/100 bp, and DMR length for most disease associations is between 1 and 3 kilobases. The principal component analyses (PCA) of the RPKM adjusted read depths at differential DMR sites for each sample are shown in Fig. 4. The PCA plots show how the samples cluster according to disease compared to non-disease when DMR sites are considered (Fig. 4).

**Fig. 5:**
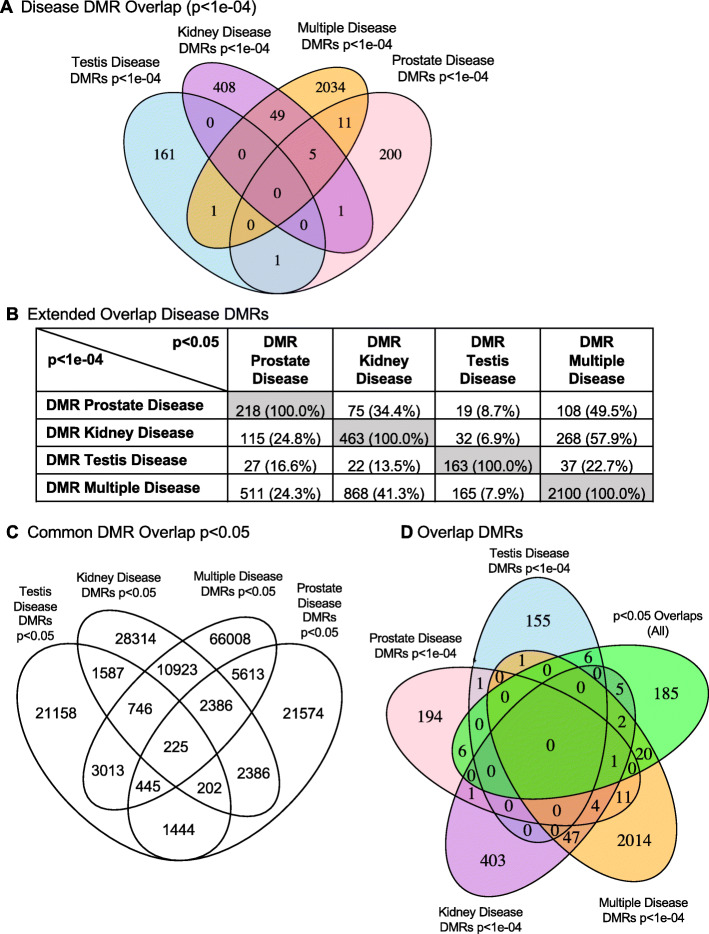
Disease DMR Overlap. **a** Venn diagram overlap of disease DMRs at *p* < 1e-04. **b** Extended overlap disease DMRs. The p-value data set at *p* < 1e-04 is compared to the *p* < 0.05 data to identify potential overlap between the different pathologies with DMR number and percentage of the total presented. The gray highlight is the expected 100% overlap. **c** Venn diagram overlap of disease DMRs at *p* < 0.05 to identify the 225 overlapping DMRs. **d** Venn diagram overlap of disease DMRs at *p* < 1e-04 and combined overlap 225 DMRs at *p* < 0.05

An overlap analysis of the different disease specific sperm DMRs is presented in Fig. 5. A Venn diagram overlap of the different disease DMRs at *p* < 1e-04 demonstrates no DMRs in common and the majority of DMRs are disease specific, Fig. 5a. An extended overlap of the disease associated DMRs (*p* < 1e-04), with each disease DMR set at a reduced significance level of *p* < 0.05 (Fig. 5b), shows 34% overlap between kidney disease and prostate disease DMRs. There is higher overlap between individual diseases and the multiple disease category, with 49% overlap between prostate and multiple disease and 58% overlap between kidney disease and multiple disease. This is anticipated as the multiple disease category includes individuals with several of the individual diseases.

Potential common DMRs among all the different disease specific DMRs was investigated. The overlap was determined at a statistical threshold of DMRs at p < 0.05, Fig. 5c. As expected, the total number of DMRs increase and 225 DMR were found to be in common between the testis, kidney, prostate, and multiple disease DMRs. A Venn diagram of the disease specific DMRs at *p* < 1e-04 and those overlapping 225 DMR at *p* < 0.05 demonstrated no common DMR among all the diseases, Fig. 5d. The highest overlap was with individual comparisons and the multiple disease, but none common with all. Observations indicate that the DMRs are predominantly disease specific with no common set of DMRs, Fig. 5.

**Fig. 6:**
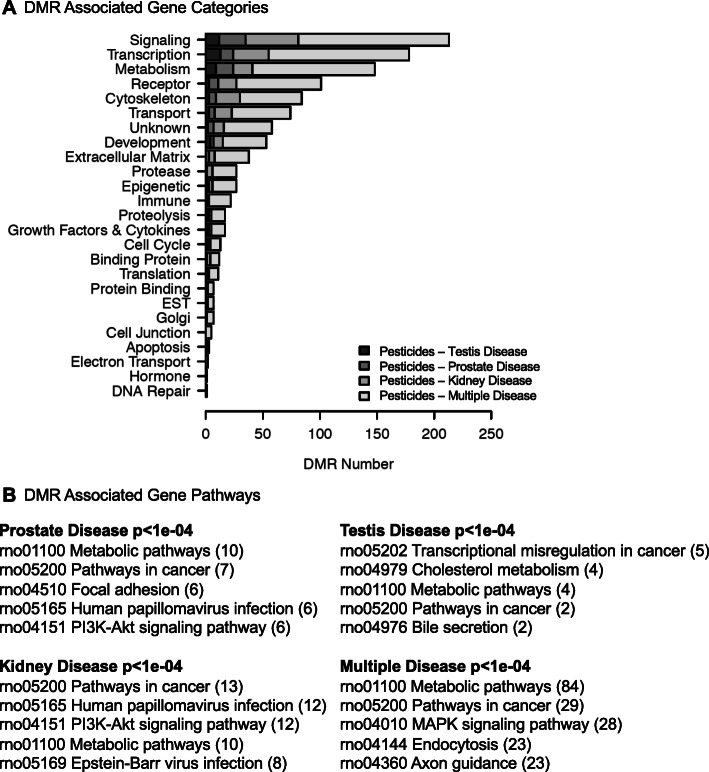
DMR associated gene categories and pathways. **a** DMR associated gene categories. The gene categories and number of associated genes are presented for each disease group. **b** DMR associated gene pathways for each disease at *p* < 1e-04 with top five KEGG pathways listed and number of associated genes in brackets

The disease specific DMR epimutation gene associations were identified and presented in Supplemental Tables S1-S4. Approximately 50% of the DMRs had gene associations for each disease set of DMRs. The gene associations were sorted with functional gene categories and presented in Fig. 6a. The signaling, transcriptional, and metabolism were predominant. The pattern of DMR associated gene categories was similar for all the disease specific epimutations. The DMR associated gene pathways were identified through a KEGG pathway analysis, as described in the Methods. The top five pathways with associated genes are presented in Fig. 6b. The number of DMR associated genes within the pathway are presented in brackets for each disease. The metabolism, pathways in cancer and various signaling pathways were common to all the diseases.

**Fig. 7:**
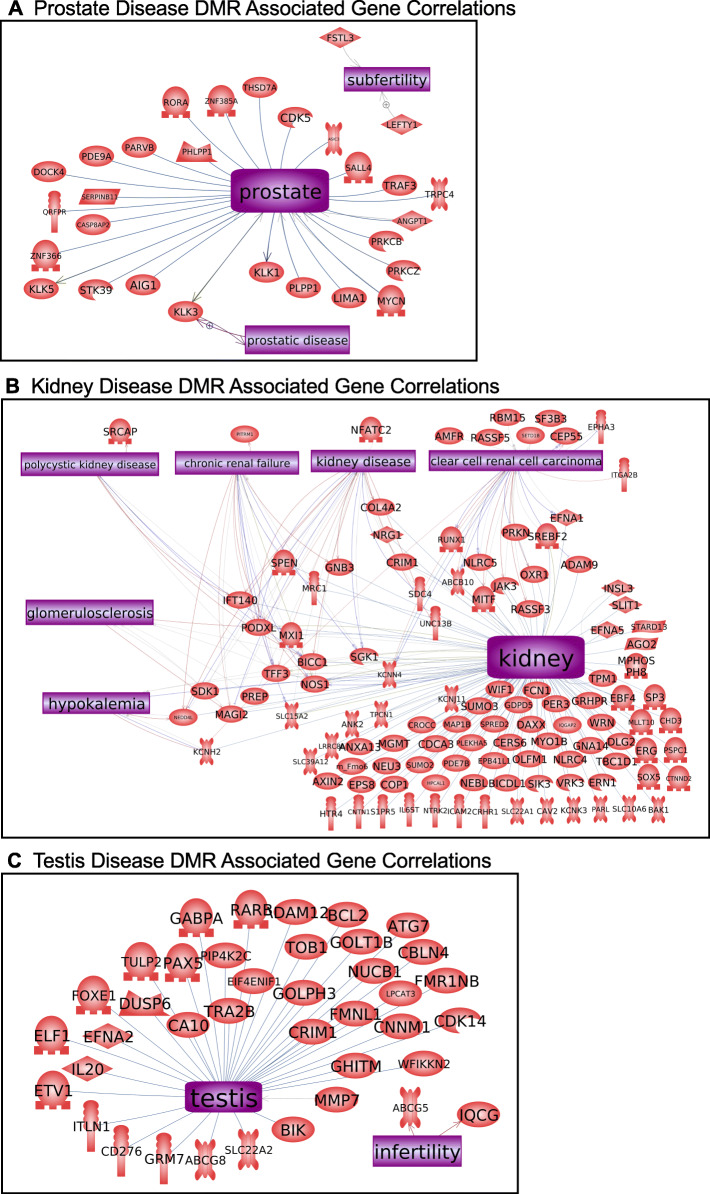
Specific disease DMR associated gene correlation with previously identified disease genes. **a** Epimutation associated prostate disease genes. **b** Epimutation associated kidney disease genes. **c** Epimutation associated testis disease genes. The gene functional category shapes are identified as follows inset in Fig. 8

**Fig. 8:**
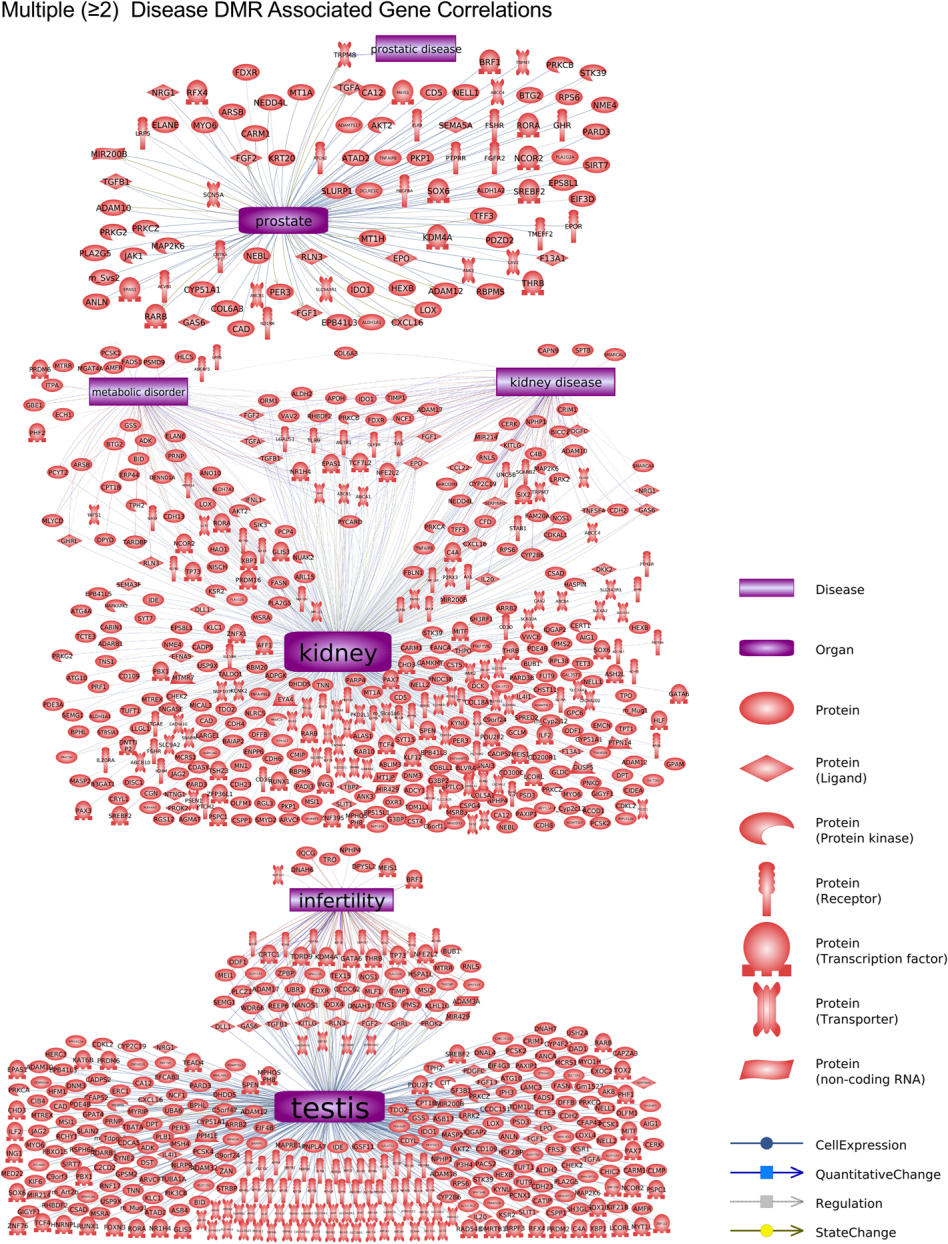
Multiple disease DMR associated gene correlations with previously identified disease genes. The disease categories and gene identified shapes are presented. Gene functional category shapes presented in the inset

The final analysis correlated the disease specific DMR associated genes with previously identified disease linked genes. A Pathway Studio gene network was used, as described in the Methods. Interestingly, the prostate, kidney and testis disease associated genes had correlations with each of the specific diseases, Fig. 7. The prostate disease DMR associated genes had links with subfertility and prostatic disease, Fig. 7a. The kidney disease DMR associated genes had links with kidney disease, chronic renal failure, and polycystic kidney disease, among others Fig. 7b. The testis disease DMR associated genes had links with infertility, Fig. 7c. The multiple disease DMR associated genes had links with all the various diseases, Fig. 8.

## Discussion

Pesticides exposure is nearly ubiquitous in the human population [1, 12, 74, 75]. Many pesticide chemicals in common use are associated with abnormalities in reproduction, development, the nervous system, and overall health [12, 74, 75]. The detrimental effects of exposure to pesticides occur not only among the individuals directly exposed, but can have transgenerational detrimental health effects in subsequent generations [33]. Direct exposure impacts an individual and their germ line [20]. If the germline epigenetics is modified, then the offspring generated with the affected germ cell can have epigenetic impacts on health and physiology. Epigenetic transgenerational inheritance occurs when future generations in the absence of exposure also exhibit alterations and increased disease incidence [20]. The molecular mechanisms for epigenetic transgenerational inheritance involve an environmental perturbation that induces germline epigenetic alterations in the DNA methylation pattern, histone modifications, and altered ncRNA expression [31, 35]. Each of these mechanisms has been associated with transgenerational exposure to environmental toxicants and increased incidence of disease [20, 31, 35]. Both the ubiquity of pesticide applications and the extent of their detrimental health effects demand careful examination of the use and regulation of these chemicals. The current study was designed to use the F3 generation males for association of sperm epigenetic alterations with specific diseases resulting from an ancestral exposure to commonly used pesticides. The objective was to potentially identify disease specific biomarkers for the associated disease susceptibility.

The combination of the pesticides permethrin and the insect repellant N,N-diethyl-meta-toluamide, DEET is commonly used in homes and public places. There are numerous health effects associated with direct exposure to permethrin and DEET, including increased neurotoxity, reduced skeletal muscle function and behavior alterations in mammals [1, 2, 4–7, 10–19]. Exposure to this specific pesticides combination (below or near the previously established NOAEL) has been linked to epigenetic transgenerational inheritance of epigenetic alterations in sperm and associated increased incidence of disease [33]. The current study extends this analysis to identify epigenetic biomarkers associated with transgenerational incidence of specific diseases resulting from ancestral exposure to the pesticide mixture. The pathologies examined are relevant to human populations including prostate, testis and kidney disease, as well as multiple disease incidence in individuals, similar to the pattern of Gulf War Illness (GWI), which has been linked to exposure to organophosphate pesticide chemicals [13–19]. The negative health effects of pesticides exposure do not stop with the individuals directly exposed but are shown to occur transgenerationally in subsequent generations [20, 30]. The identification of epigenetic biomarkers can be potentially used for determining later life health effects and health effects on future generations.

Epigenetic alterations are more common among individuals with disease [34, 37, 40, 41] than specific genetic alterations or mutations, which often appear in a very small percentage of individuals with the associated disease. The current study examined differential DNA methylation regions (DMRs) between specific diseased and non-diseased individuals whose great-grandparents were exposed to the pesticides mixture. Several hundred transgenerational epigenetic biomarkers are associated with each disease resulting from ancestral exposure to pesticides (Fig. 1). There are two thousand DMRs associated with the multiple disease condition, where individual animals each exhibit multiple diseases. These epimutations are distributed widely across the genome (Fig. 2) leading to the conclusion that the epigenetic effect of the ancestral exposure are epigenome-wide. The transgenerational disease specific DMRs identified were found to be predominantly unique for the individual disease with no DMRs in common, Fig. 5. An extended overlap of DMRs for two disease comparisons shows between 7 and 34% overlap with a relaxed significance level. Overlaps between individual and multiple diseases are higher, ranging between 22 and 50%. The overlapping DMRs at *p* < 0.05 also had no common DMRs between disease specific DMRs at *p* < 1e-04. Therefore, no common DMR among the different transgenerational disease DMR biomarkers was identified. Observations suggest a common set of epimutations is not present between different diseases to alter general disease susceptibility. Although suggestions of such general molecular impacts for disease susceptibility may exist, the current study suggests predominately disease specific epimutations. This supports the potential ability of epigenetic biomarkers to be used in the future as disease diagnostics. These transgenerational disease DNA methylations alterations helps provide an explanation for the system-wide health effects of exposure to pesticides. This is exemplified by the Gulf War GWI population, which often has exposure to permethrin and DEET, among other chemicals [11–13, 76]. Exposure to these toxicant chemicals results in a direct, multigenerational, and transgenerational epigenome-wide effects [33], and this is associated with increased incidence of negative health effects and disease.

A limitation of the current study was the low numbers of animals with a specific individual disease. Although an edgeR *p*-value was used to identify and analyze the disease associated DMRs, further analyses adjusting for multiple testing using the false discovery rate (FDR) resulted in FDR *p*-values for the disease epimutations of > 0.1 in all comparisons except the multiple disease phenotype. The low sample number is likely the most important limitation in the current analysis. Potential higher variability in the data needs to be considered even though higher edgeR values were used, but this does not address multiple testing corrections. Future studies will need to use higher n-values and/or better statistical models to reduce this FDR analysis limitation [63–68].

## Conclusions

The current study used an epigenome-wide association analysis to identify an epigenetic signature of transgenerational disease present in sperm [33]. The biomarkers identified herein may potentially be used to asses paternal transmission of disease susceptibilities to future generations. Preconception diagnoses could be used to determine health trajectories for offspring of exposed individuals. This could be particularly important among populations known to experience higher and more chronic levels of exposure to pesticides, such as military and minority populations [12, 22, 32]. DNA methylation alterations are found in the sperm of agricultural workers [27], another population known to experience higher levels of pesticides exposure. These epigenetic biomarkers associated with disease among ancestrally exposed populations will potentially provide a crucial tool for assessing the health effects in later life for the current generation and the health prospects for future generations.

## Supplementary information

**Additional file 1K Supplemental Table S1.** DMR Site List Prostate *p* < 1e-04. DMR name, chromosome, start, stop, length, number signature windows, minimum *p*-value, max log-fold change, CpG number, CpG density, gene annotation, and gene category are presented. **Supplemental Table S2.** DMR Site List Kidney *p* < 1e-04. DMR name, chromosome, start, stop, length, number signature windows, minimum p-value, max log-fold change, CpG number, CpG density, gene annotation, and gene category are presented. **Supplemental Table S3.** DMR Site List Testis *p* < 1e-04. DMR name, chromosome, start, stop, length, number signature windows, minimum p-value, max log-fold change, CpG number, CpG density, gene annotation, and gene category are presented. **Supplemental Table S4.** DMR Site List Multiple *p* < 1e-04. DMR name, chromosome, start, stop, length, number signature windows, minimum p-value, max log-fold change, CpG number, CpG density, gene annotation, and gene category are presented.

## Data Availability

All molecular data has been deposited into the public database at NCBI (GEO # GSE158254) and R code computational tools available at GitHub (https://github.com/skinnerlab/MeDIP-seq) and www.skinner.wsu.edu.

## Abbreviations

DEET: N,N-diethyl-meta-toluamide
EWAS: Epigenome-wide association study
DMRs: DNA methylation regions
GWI: Gulf War Illness
NOAEL: No observable adverse effect level
LOAEL: Lowest observable adverse effect level
DMSO: Dimethyl sulfoxide
BMI: Body mass index
MeDIP: Methylated DNA immunoprecipitation
MT: Mitochondrial DNA
PCA: Principal component analyses
FDR: False discovery rate
H & E: Hematoxylin, and eosin
WADDL: Washington Animal Disease Diagnostic Laboratory
QC: Quality control
PBS: Phosphate buffer saline
NGS: Next generation sequencing
