# Supplementary material for: Epigenome-wide association study for pesticide (Permethrin and DEET) induced DNA methylation epimutation biomarkers for specific transgenerational disease
Source: Environ Health. 2020 Nov 4;19:109. doi: 10.1186/s12940-020-00666-y (PMC7643320; doi:10.1186/s12940-020-00666-y)
Supplement: Supplementary file 1 — Additional file 1K Supplemental Table S1. DMR Site List Prostate p < 1e-04. DMR name, chromosome, start, stop, length, number signature windows, minimum p-value, max log-fold change, CpG number, CpG density, gene annotation, and gene category are presented. Supplemental Table S2. DMR Site List Kidney p < 1e-04. DMR name, chromosome, start, stop, length, number signature windows, minimum p-value, max log-fold change, CpG number, CpG density, gene annotation, and gene category are presented. Supplemental Table S3. DMR Site List Testis p < 1e-04. DMR name, chromosome, start, stop, length, number signature windows, minimum p-value, max log-fold change, CpG number, CpG density, gene annotation, and gene category are presented. Supplemental Table S4. DMR Site List Multiple p < 1e-04. DMR name, chromosome, start, stop, length, number signature windows, minimum p-value, max log-fold change, CpG number, CpG density, gene annotation, and gene category are presented. [file 12940_2020_666_MOESM1_ESM.zip › SuppTable-S1_siteTable.pesticides.prostate.1e-04R2.pdf]

Supplemental Table S1
DMR Site Table Pesticides Prostate Disease p<1e-04

DMR Name	Chr	Start	Stop	Length	# Sig Win	minP	maxLFC	CpG #	CpG Density	Gene Annotation	Gene Category
DMR1:4165001	1	4165001	4166000	1000	1	1.49E-05	-2.0427042	2	0.2		
DMR1:8008001	1	8008001	8009000	1000	1	4.93E-05	-0.8378083	30	3	Aig1	
DMR1:11314001	1	11314001	11315000	1000	1	5.63E-05	-1.0648991	8	0.8		
DMR1:19008001	1	19008001	19009000	1000	1	2.70E-05	0.8892744	9	0.9	Lama2	Extracellular Matrix
DMR1:29270001	1	29270001	29272000	2000	1	9.67E-05	-0.9985928	27	1.35	Ncoa7	
DMR1:47222001	1	47222001	47223000	1000	1	2.90E-05	-0.9196588	19	1.9	Dynlt1;Sytl3	Development;Transport
DMR1:50115001	1	50115001	50116000	1000	1	5.91E-05	-0.8829326	15	1.5		
DMR1:52902001	1	52902001	52903000	1000	1	9.43E-05	-0.7119201	10	1	Tbxt;T2	
DMR1:86464001	1	86464001	86465000	1000	1	1.93E-05	-0.947703	10	1		
DMR1:95640001	1	95640001	95641000	1000	1	1.98E-05	-0.8947304	12	1.2		
DMR1:98872001	1	98872001	98873000	1000	1	2.17E-05	1.0359546	2	0.2		
DMR1:99781001	1	99781001	99782000	1000	1	4.53E-05	-1.6439741	16	1.6	Klk5	Protease
DMR1:100772001	1	100772001	100773000	1000	1	5.09E-05	0.9945841	13	1.3	Vrk3	Signaling
DMR1:100826001	1	100826001	100827000	1000	1	4.83E-05	-1.0481354	14	1.4	Nup62;Il4i1;Tbc1d17	Metabolism;Signaling
DMR1:114498001	1	114498001	114500000	2000	1	2.49E-05	1.121732	10	0.5	Herc2	Metabolism
DMR1:129852001	1	129852001	129853000	1000	1	2.29E-05	0.9765007	15	1.5		
DMR1:188674001	1	188674001	188675000	1000	1	2.49E-05	-1.2434827	10	1	Iqck	
DMR1:190648001	1	190648001	190649000	1000	1	7.51E-05	-0.7904916	9	0.9		
DMR1:192409001	1	192409001	192411000	2000	1	2.79E-06	-1.1950767	33	1.65	Prkcb;7SK	Signaling
DMR1:217417001	1	217417001	217418000	1000	1	1.58E-05	1.0024945	16	1.6	Shank2;5S_rRNA	Protein Binding
DMR1:223748001	1	223748001	223749000	1000	1	5.91E-05	-0.6253766	46	4.6		
DMR1:226015001	1	226015001	226016000	1000	1	2.71E-05	-1.2437591	13	1.3		
DMR1:242677001	1	242677001	242678000	1000	1	6.98E-05	0.5857485	0	0	Pgm5	Metabolism
DMR1:243459001	1	243459001	243460000	1000	1	1.66E-05	-1.1432017	18	1.8		
DMR1:248827001	1	248827001	248828000	1000	1	3.31E-05	-0.9402592	0	0		
DMR1:260873001	1	260873001	260874000	1000	1	1.73E-05	-0.9259854	21	2.1	Slit1	Receptor
DMR1:267610001	1	267610001	267611000	1000	1	4.43E-05	-0.8203005	29	2.9	Gsto2;Gsto1	Metabolism
DMR2:506001	2	506001	508000	2000	1	5.62E-05	0.8702618	9	0.45		
DMR2:24782001	2	24782001	24783000	1000	1	1.98E-05	1.253867	16	1.6	Pde8b	Signaling
DMR2:29468001	2	29468001	29469000	1000	1	5.07E-05	0.6979455	4	0.4	Zfp366	Transcription
DMR2:30749001	2	30749001	30750000	1000	1	3.19E-05	-1.0385766	17	1.7	Mrps36	
DMR2:44680001	2	44680001	44681000	1000	1	1.33E-05	-1.0967749	11	1.1	Plpp1	
DMR2:63889001	2	63889001	63890000	1000	1	4.31E-05	-1.4500297	0	0		
DMR2:104629001	2	104629001	104630000	1000	1	7.56E-05	-1.4586677	2	0.2		
DMR2:112329001	2	112329001	112330000	1000	1	4.97E-05	0.8527433	7	0.7	Spata16	
DMR2:118219001	2	118219001	118220000	1000	1	2.02E-06	-1.3873591	13	1.3		
DMR2:122919001	2	122919001	122920000	1000	1	9.00E-05	-1.0936835	23	2.3	Qrfpr	Receptor
DMR2:143469001	2	143469001	143470000	1000	1	3.92E-05	-0.9336295	9	0.9	Trpc4	Development
DMR2:187010001	2	187010001	187011000	1000	1	5.55E-06	-1.305898	13	1.3	Arhgef11	Signaling
DMR2:194961001	2	194961001	194962000	1000	1	1.02E-05	-0.8090492	10	1		
DMR2:231162001	2	231162001	231163000	1000	1	2.52E-05	1.0883022	11	1.1		
DMR2:231738001	2	231738001	231739000	1000	1	2.65E-05	-1.5148337	25	2.5		
DMR2:247106001	2	247106001	247107000	1000	1	2.20E-05	-1.2638253	9	0.9		
DMR2:250059001	2	250059001	250060000	1000	1	9.50E-05	-0.8775279	23	2.3		
DMR2:260707001	2	260707001	260708000	1000	1	8.74E-05	0.6608728	14	1.4		
DMR2:264603001	2	264603001	264604000	1000	1	4.43E-05	0.7089819	14	1.4	U6	
DMR3:29318001	3	29318001	29319000	1000	1	5.32E-05	0.8532274	13	1.3		
DMR3:29657001	3	29657001	29658000	1000	1	7.83E-05	-1.0543532	3	0.3	Gtdc1	Metabolism
DMR3:33415001	3	33415001	33416000	1000	1	3.12E-05	1.1510917	5	0.5		
DMR3:45657001	3	45657001	45658000	1000	1	9.74E-05	-0.7162727	17	1.7		
DMR3:54597001	3	54597001	54598000	1000	1	7.87E-05	0.6772414	21	2.1	Stk39	
DMR3:58609001	3	58609001	58610000	1000	1	4.40E-05	-0.901571	18	1.8		
DMR3:59532001	3	59532001	59533000	1000	1	8.97E-05	0.579953	10	1		
DMR3:75422001	3	75422001	75424000	2000	1	4.85E-05	0.6423682	23	1.15	Olr560;AC118490.1	
DMR3:77597001	3	77597001	77598000	1000	1	8.10E-05	-0.8495996	14	1.4	Olr665	Receptor
DMR3:101257001	3	101257001	101258000	1000	1	4.09E-05	-1.02448	12	1.2		
DMR3:113144001	3	113144001	113146000	2000	1	8.77E-06	0.6072478	4	0.2	Tubgcp4	Cytoskeleton
DMR3:127987001	3	127987001	127988000	1000	1	9.60E-05	-1.1303213	5	0.5		
DMR3:159541001	3	159541001	159542000	1000	1	6.45E-05	-0.723537	19	1.9		
DMR3:165521001	3	165521001	165522000	1000	1	1.07E-05	0.9830886	14	1.4	Sall4	Transcription
DMR4:2880001	4	2880001	2881000	1000	1	2.19E-05	1.1509419	1	0.1		
DMR4:4585001	4	4585001	4587000	2000	1	6.13E-05	-1.2254468	14	0.7		
DMR4:6769001	4	6769001	6770000	1000	1	9.09E-05	0.8948203	12	1.2	Prkag2	Signaling
DMR4:7290001	4	7290001	7291000	1000	1	6.41E-05	0.96682	26	2.6	Slc4a2;Cdk5;Asic3;Abcb8	Metabolism;Signaling;Transport

DMR4:39011001	4	39011001	39012000	1000	1	6.61E-06	1.0934206	10	1	Thsd7a	Extracellular Matrix
DMR4:50987001	4	50987001	50988000	1000	1	8.50E-05	0.790195	7	0.7		
DMR4:51115001	4	51115001	51116000	1000	1	7.22E-05	1.0044855	10	1	7SK;Slc13a1	Transport
DMR4:57184001	4	57184001	57185000	1000	1	5.30E-05	-0.4753067	7	0.7	Ahcyl2	Metabolism
DMR4:61360001	4	61360001	61361000	1000	1	3.80E-05	0.8742168	7	0.7	Exoc4	Transport
DMR4:133249001	4	133249001	133250000	1000	1	6.80E-05	0.7404946	9	0.9	Gxylt2	
DMR4:142106001	4	142106001	142107000	1000	1	5.08E-05	-0.709773	11	1.1		
DMR4:154052001	4	154052001	154053000	1000	1	3.42E-05	-0.8002809	19	1.9	Iqsec3	Signaling
DMR4:161823001	4	161823001	161825000	2000	1	5.94E-06	-0.9360543	50	2.5	Pzp;AC118427.1	Development
DMR4:162795001	4	162795001	162796000	1000	1	5.15E-05	-0.6081398	7	0.7	Klrb1	
DMR4:167839001	4	167839001	167840000	1000	1	7.69E-05	0.7216882	15	1.5	Etv6	Transcription
DMR4:170561001	4	170561001	170562000	1000	1	1.97E-05	-1.2928594	17	1.7	Atf7ip;Plbd1	EST
DMR4:178191001	4	178191001	178192000	1000	1	1.27E-05	-1.2563699	17	1.7	Sox5	Transcription
DMR4:180761001	4	180761001	180762000	1000	1	4.72E-05	-0.6859614	20	2		
DMR5:8616001	5	8616001	8617000	1000	1	8.41E-05	0.9872263	13	1.3	AABR07046830.1	
DMR5:22383001	5	22383001	22384000	1000	1	7.04E-05	0.9732973	9	0.9	Clvs1	
DMR5:25571001	5	25571001	25572000	1000	1	2.30E-05	-1.0151071	6	0.6	Pdp1	Transcription
DMR5:47981001	5	47981001	47982000	1000	1	4.78E-05	1.0736005	7	0.7	AABR07047789.1	
DMR5:59213001	5	59213001	59214000	1000	1	2.57E-05	0.7405296	12	1.2	Olr834	Receptor
DMR5:68459001	5	68459001	68460000	1000	1	7.85E-05	0.6287253	7	0.7		
DMR5:95863001	5	95863001	95864000	1000	1	4.39E-06	-1.7957473	8	0.8		
DMR5:113044001	5	113044001	113045000	1000	1	3.13E-05	-0.7530502	52	5.2		
DMR5:130105001	5	130105001	130106000	1000	1	1.69E-05	0.9089493	11	1.1		
DMR5:130765001	5	130765001	130766000	1000	1	1.42E-05	0.4757109	2	0.2		
DMR5:134756001	5	134756001	134757000	1000	1	5.79E-05	0.9149543	13	1.3		
DMR5:142298001	5	142298001	142299000	1000	1	2.27E-05	-1.3231152	17	1.7	LOC103689941	
DMR5:143467001	5	143467001	143468000	1000	1	5.67E-05	-1.3717032	10	1		
DMR5:143738001	5	143738001	143739000	1000	1	6.09E-05	0.730998	11	1.1		
DMR5:147226001	5	147226001	147227000	1000	1	8.87E-05	1.102293	16	1.6		
DMR5:156338001	5	156338001	156340000	2000	1	1.00E-05	0.8138921	21	1.05		
DMR5:159410001	5	159410001	159411000	1000	1	1.59E-05	-0.9887539	37	3.7		
DMR5:172728001	5	172728001	172729000	1000	1	7.50E-06	0.8825794	14	1.4	Prkcz	Signaling
DMR6:38230001	6	38230001	38231000	1000	1	1.48E-05	-1.1872241	11	1.1	Mycn	Transcription
DMR6:58579001	6	58579001	58580000	1000	1	7.17E-05	0.8441809	4	0.4		
DMR6:60587001	6	60587001	60588000	1000	1	5.40E-05	-1.2457331	9	0.9	Dock4	Signaling
DMR6:74709001	6	74709001	74710000	1000	1	5.94E-05	-1.0830407	11	1.1		
DMR6:75873001	6	75873001	75874000	1000	1	9.28E-05	1.0543215	14	1.4	Baz1a	Metabolism
DMR6:78149001	6	78149001	78150000	1000	1	4.69E-05	0.8042336	17	1.7		
DMR6:79340001	6	79340001	79342000	2000	1	1.23E-05	0.6620027	14	0.7		
DMR6:92692001	6	92692001	92694000	2000	1	8.11E-05	0.958382	37	1.85	Trim9	Unknown
DMR6:92765001	6	92765001	92766000	1000	1	4.96E-05	-0.9940464	13	1.3	Trim9	Unknown
DMR6:98660001	6	98660001	98661000	1000	1	1.64E-05	0.9340361	6	0.6	AABR07064867.1	
DMR6:99140001	6	99140001	99141000	1000	1	7.48E-05	-1.0214367	11	1.1	AABR07064873.1	
DMR6:118886001	6	118886001	118887000	1000	1	5.85E-05	0.8795385	2	0.2		
DMR6:135617001	6	135617001	135618000	1000	1	4.54E-05	1.0179811	12	1.2	Traf3	Apoptosis
DMR7:1974001	7	1974001	1975000	1000	1	2.32E-05	-2.0539215	3	0.3		
DMR7:11053001	7	11053001	11054000	1000	1	3.90E-05	1.1909152	14	1.4	Gna11;Gna15	Signaling
DMR7:12813001	7	12813001	12814000	1000	1	1.68E-05	-1.2526059	21	2.1	Prss57;Fstl3;Rnf126	Signaling
DMR7:54208001	7	54208001	54210000	2000	1	6.00E-05	-0.8052565	32	1.6	Nap1l1	Signaling
DMR7:78379001	7	78379001	78380000	1000	1	5.05E-05	0.7427281	4	0.4	Rims2	Signaling
DMR7:81594001	7	81594001	81595000	1000	1	4.40E-05	0.9286494	15	1.5	Angpt1	Growth Factors & Cytokines
DMR7:89795001	7	89795001	89796000	1000	1	4.17E-05	-1.075596	6	0.6		
DMR7:123867001	7	123867001	123868000	1000	1	9.47E-06	-1.2354107	24	2.4	Nfam1	
DMR7:125245001	7	125245001	125247000	2000	1	6.74E-05	1.1761986	14	0.7	Parvb	Cytoskeleton
DMR7:125909001	7	125909001	125910000	1000	1	4.78E-06	0.8721013	9	0.9		
DMR7:128384001	7	128384001	128385000	1000	1	6.54E-05	-1.1346816	17	1.7		
DMR7:138619001	7	138619001	138620000	1000	1	6.86E-05	1.0244232	20	2		
DMR7:141439001	7	141439001	141440000	1000	1	9.83E-05	0.9375661	7	0.7	Lima1	Cytoskeleton
DMR7:144970001	7	144970001	144971000	1000	1	2.30E-05	-1.0199044	17	1.7	Zfp385a;Itga5	Transcription;Signaling
DMR8:334001	8	334001	335000	1000	1	8.69E-05	-1.5459674	3	0.3		
DMR8:1461001	8	1461001	1462000	1000	1	9.56E-05	0.7026418	6	0.6	Kbtbd3	Transcription
DMR8:20524001	8	20524001	20525000	1000	1	2.40E-05	0.9473564	7	0.7		
DMR8:72675001	8	72675001	72676000	1000	1	9.47E-05	1.1226261	10	1	Rab8b	Signaling
DMR8:75521001	8	75521001	75522000	1000	1	6.07E-05	-0.9280595	13	1.3	Rora	Receptor
DMR8:87101001	8	87101001	87102000	1000	1	6.46E-05	1.008382	9	0.9	Col12a1	Cytoskeleton
DMR8:93693001	8	93693001	93694000	1000	1	7.66E-07	1.0817129	10	1		
DMR8:94976001	8	94976001	94977000	1000	1	4.34E-05	0.9749537	16	1.6		
DMR8:106718001	8	106718001	106719000	1000	1	7.70E-05	-1.4124658	24	2.4		

DMR8:114591001	8	114591001	114592000	1000	1	7.41E-05	0.9057541	9	0.9	Col6a5	
DMR8:115226001	8	115226001	115229000	3000	1	8.39E-05	1.0063741	53	1.77	Iqcf3	
DMR8:121432001	8	121432001	121433000	1000	1	8.35E-05	-0.7440165	17	1.7	LOC102554058	
DMR8:125340001	8	125340001	125341000	1000	1	3.18E-05	-1.201349	22	2.2	Rbms3	Epigenetic
DMR9:17009001	9	17009001	17010000	1000	1	5.61E-05	0.4002774	5	0.5		
DMR9:36616001	9	36616001	36617000	1000	1	1.95E-05	1.0411629	1	0.1		
DMR9:45959001	9	45959001	45960000	1000	1	2.13E-05	-1.094026	17	1.7	Npas2	Transcription
DMR9:56815001	9	56815001	56816000	1000	1	4.37E-05	0.8028507	12	1.2		
DMR9:85322001	9	85322001	85323000	1000	1	5.59E-05	-1.3187073	9	0.9		
DMR9:87037001	9	87037001	87038000	1000	1	3.67E-05	-1.3833931	12	1.2	Nyap2	
DMR9:91352001	9	91352001	91353000	1000	1	9.67E-06	-1.2557758	13	1.3		
DMR9:99018001	9	99018001	99019000	1000	1	8.49E-05	-1.1677302	16	1.6		
DMR9:106376001	9	106376001	106377000	1000	1	3.21E-05	0.7103532	9	0.9		
DMR9:113393001	9	113393001	113394000	1000	1	7.09E-05	0.8075859	15	1.5	Rab31	Signaling
DMR9:115403001	9	115403001	115404000	1000	1	5.93E-05	-1.4878065	15	1.5	Ptprm	Receptor
DMR9:118527001	9	118527001	118528000	1000	1	2.91E-05	0.8746215	11	1.1		
DMR10:4240001	10	4240001	4241000	1000	1	1.36E-05	-1.1488488	20	2	Snx29	Cytoskeleton
DMR10:8386001	10	8386001	8387000	1000	1	6.07E-05	0.9140955	11	1.1	Rbfox1	Unknown
DMR10:9433001	10	9433001	9434000	1000	1	8.97E-05	-1.1205247	16	1.6		
DMR10:17462001	10	17462001	17464000	2000	1	3.88E-05	0.9028018	9	0.45	Stk10	Signaling
DMR10:42675001	10	42675001	42676000	1000	1	2.04E-06	-1.2829681	12	1.2	Gria1	Receptor
DMR10:58362001	10	58362001	58363000	1000	1	3.91E-05	-0.5772156	24	2.4	Wscd1	Metabolism
DMR11:13260001	11	13260001	13262000	2000	1	2.68E-05	0.8069679	19	0.95		
DMR11:34690001	11	34690001	34691000	1000	1	2.37E-05	1.1762268	15	1.5	Ttc3	Unknown
DMR11:73340001	11	73340001	73341000	1000	1	5.11E-05	-1.0415702	17	1.7		
DMR11:82267001	11	82267001	82268000	1000	1	4.52E-06	0.8656827	17	1.7		
DMR12:39335001	12	39335001	39336000	1000	1	1.05E-06	0.8388749	17	1.7	P2rx4	Metabolism
DMR12:48069001	12	48069001	48070000	1000	1	7.90E-05	-0.6959747	32	3.2	AABR07036556.1	
DMR12:49763001	12	49763001	49764000	1000	1	9.77E-05	0.9689338	10	1	Myo18b	Cytoskeleton
DMR13:2044001	13	2044001	2045000	1000	1	8.50E-05	1.1220112	3	0.3		
DMR13:16531001	13	16531001	16532000	1000	1	8.31E-05	1.0208931	8	0.8		
DMR13:26340001	13	26340001	26341000	1000	1	6.61E-05	-0.732048	23	2.3	Phlpp1	Signaling
DMR13:27244001	13	27244001	27245000	1000	1	8.00E-05	0.890641	6	0.6	Serpinb11	Signaling
DMR13:73442001	13	73442001	73443000	1000	1	4.01E-05	1.0011592	8	0.8	Qsox1	Metabolism
DMR13:77830001	13	77830001	77831000	1000	1	7.73E-05	-0.9087785	17	1.7	AABR07021544.1;AABR07021544.2;Tnn	Extracellular Matrix
DMR13:77966001	13	77966001	77967000	1000	1	8.70E-05	-0.4826226	40	4	Cacybp	Receptor
DMR13:79888001	13	79888001	79889000	1000	1	1.59E-05	-0.7947445	24	2.4	RGD1309106;Pigc	Unknown;Metabolism
DMR13:82583001	13	82583001	82585000	2000	1	7.57E-06	-0.9063883	21	1.05	Ccdc181;Blzf1	Transcription
DMR13:99204001	13	99204001	99205000	1000	1	6.12E-05	-0.8414826	10	1	Lefty1	Signaling
DMR13:99396001	13	99396001	99397000	1000	1	5.97E-06	1.0863681	12	1.2	RGD1564463	
DMR13:100133001	13	100133001	100134000	1000	1	7.00E-05	-0.9412695	13	1.3	Eif3el1	
DMR13:109113001	13	109113001	109114000	1000	1	2.28E-05	0.9915178	14	1.4		
DMR14:6609001	14	6609001	6610000	1000	1	3.36E-05	1.1341603	20	2	Pkd2	Transport
DMR14:23173001	14	23173001	23174000	1000	1	2.72E-05	-0.8904326	15	1.5	Tmprss11g	
DMR14:36725001	14	36725001	36726000	1000	1	2.01E-05	-1.5307622	16	1.6	Usp46	Proteolysis
DMR14:59026001	14	59026001	59027000	1000	1	6.87E-05	0.7978846	18	1.8		
DMR14:64782001	14	64782001	64783000	1000	1	2.05E-05	-0.9374484	21	2.1	Adgra3	
DMR14:72502001	14	72502001	72503000	1000	1	6.38E-05	-1.1403395	10	1		
DMR14:94364001	14	94364001	94365000	1000	1	2.14E-05	-0.5573462	60	6		
DMR14:95682001	14	95682001	95683000	1000	1	2.30E-05	0.5719498	14	1.4		
DMR14:95879001	14	95879001	95880000	1000	1	8.30E-05	0.6093625	12	1.2		
DMR15:2906001	15	2906001	2908000	2000	1	8.70E-05	-0.7305242	55	2.75	Kat6b	Epigenetic
DMR15:36824001	15	36824001	36826000	2000	1	5.12E-05	0.318836	28	1.4	AABR07018038.1	
DMR15:37638001	15	37638001	37639000	1000	1	4.97E-05	-1.6569065	16	1.6	Cryl1	Metabolism
DMR15:50052001	15	50052001	50053000	1000	1	5.48E-06	0.9415179	17	1.7		
DMR15:64672001	15	64672001	64673000	1000	1	6.89E-05	-0.6376706	13	1.3		
DMR15:78735001	15	78735001	78736000	1000	1	6.08E-06	0.8462953	5	0.5		
DMR15:108469001	15	108469001	108470000	1000	1	5.55E-05	-1.0692985	16	1.6		
DMR16:2040001	16	2040001	2041000	1000	1	9.54E-05	0.5882924	12	1.2	Zcchc24	
DMR16:73916001	16	73916001	73917000	1000	1	5.84E-05	1.0243425	25	2.5		
DMR16:82634001	16	82634001	82635000	1000	1	8.75E-07	-0.945389	21	2.1		
DMR17:13859001	17	13859001	13860000	1000	1	2.09E-05	-0.864294	15	1.5	Metazoa_SRP	
DMR17:28033001	17	28033001	28034000	1000	1	9.80E-05	-1.5686828	23	2.3		
DMR17:33754001	17	33754001	33755000	1000	1	5.22E-05	1.1704039	5	0.5	Gmds	Metabolism
DMR17:37155001	17	37155001	37156000	1000	1	6.58E-05	0.9328142	7	0.7	Cdkal1	Cell Cycle
DMR17:49012001	17	49012001	49014000	2000	1	1.16E-05	0.8756811	21	1.05		
DMR17:70038001	17	70038001	70039000	1000	1	5.28E-05	0.6217101	12	1.2	AABR07028446.1	

DMR17:80310001	17	80310001	80311000	1000	1	1.66E-06	-1.4830389	19	1.9	Pter;C1ql3	Metabolism;Immune
DMR17:86172001	17	86172001	86173000	1000	1	3.23E-05	-0.7273304	6	0.6		
DMR18:28919001	18	28919001	28920000	1000	1	4.86E-05	-1.0561241	14	1.4	Nrg2	Signaling
DMR18:31812001	18	31812001	31814000	2000	1	5.60E-05	-0.8297977	41	2.05	Arhgap26	Signaling
DMR18:32374001	18	32374001	32375000	1000	1	9.05E-05	-1.3578621	17	1.7	AC096226.1	
DMR18:51591001	18	51591001	51592000	1000	1	1.56E-05	0.9515831	32	3.2	Gramd2b	
DMR18:62744001	18	62744001	62745000	1000	1	6.03E-05	-1.4296408	23	2.3	AABR07032361.1	
DMR18:63063001	18	63063001	63064000	1000	1	4.94E-05	-1.0252066	14	1.4		
DMR18:64536001	18	64536001	64537000	1000	1	1.46E-05	-0.9202338	21	2.1		
DMR18:64862001	18	64862001	64863000	1000	1	7.17E-05	-0.7926462	12	1.2		
DMR18:73017001	18	73017001	73018000	1000	1	2.68E-05	-0.9371011	14	1.4		
DMR18:84487001	18	84487001	84488000	1000	1	8.56E-05	0.8497824	13	1.3		
DMR19:4822001	19	4822001	4823000	1000	1	1.41E-06	-1.0352801	18	1.8		
DMR19:20210001	19	20210001	20211000	1000	1	9.41E-05	1.0300542	9	0.9	Zfp423	Transcription
DMR19:22556001	19	22556001	22557000	1000	1	8.97E-05	-0.6500241	9	0.9		
DMR19:29258001	19	29258001	29259000	1000	1	9.26E-05	0.4354217	9	0.9	AABR07043564.1	
DMR19:33688001	19	33688001	33689000	1000	1	8.83E-05	-0.9699507	14	1.4		
DMR20:19381001	20	19381001	19382000	1000	1	4.01E-05	0.9480159	19	1.9	Fam13c	
DMRX:114265001	X	114265001	114266000	1000	1	7.92E-05	-0.6681248	15	1.5		
