# Supplementary material for: Epigenome-wide association study for pesticide (Permethrin and DEET) induced DNA methylation epimutation biomarkers for specific transgenerational disease
Source: Environ Health. 2020 Nov 4;19:109. doi: 10.1186/s12940-020-00666-y (PMC7643320; doi:10.1186/s12940-020-00666-y)
Supplement: Supplementary file 1 — Additional file 1K Supplemental Table S1. DMR Site List Prostate p < 1e-04. DMR name, chromosome, start, stop, length, number signature windows, minimum p-value, max log-fold change, CpG number, CpG density, gene annotation, and gene category are presented. Supplemental Table S2. DMR Site List Kidney p < 1e-04. DMR name, chromosome, start, stop, length, number signature windows, minimum p-value, max log-fold change, CpG number, CpG density, gene annotation, and gene category are presented. Supplemental Table S3. DMR Site List Testis p < 1e-04. DMR name, chromosome, start, stop, length, number signature windows, minimum p-value, max log-fold change, CpG number, CpG density, gene annotation, and gene category are presented. Supplemental Table S4. DMR Site List Multiple p < 1e-04. DMR name, chromosome, start, stop, length, number signature windows, minimum p-value, max log-fold change, CpG number, CpG density, gene annotation, and gene category are presented. [file 12940_2020_666_MOESM1_ESM.zip › SuppTable-S2_siteTable.pesticides.kidney.1e-04R2.pdf]

## Supplemental Table S2
## DMR Site Table Pesticides Kidney Disease p<1e-04

| DMR Name | Chr | Start | Stop | Length | # Sig Win | minP | maxLFC | CpG # | CpG Density | Gene Annotation | Gene Category |
|---|---|---|---|---|---|---|---|---|---|---|---|
| DMR1:626001 | 1 | 626001 | 627000 | 1000 | 1 | 2.59E-05 | -0.9615309 | 53 | 5.3 | | |
| DMR1:15965001 | 1 | 15965001 | 15966000 | 1000 | 1 | 8.82E-05 | -0.5288945 | 23 | 2.3 | Pde7b | Signaling |
| DMR1:24177001 | 1 | 24177001 | 24179000 | 2000 | 1 | 6.77E-05 | 0.977734 | 23 | 1.15 | Sgk1 | Signaling |
| DMR1:24209001 | 1 | 24209001 | 24211000 | 2000 | 1 | 3.18E-06 | -0.9085937 | 35 | 1.75 | Sgk1 | Signaling |
| DMR1:39721001 | 1 | 39721001 | 39722000 | 1000 | 1 | 6.95E-05 | -0.7867433 | 30 | 3 | | |
| DMR1:45304001 | 1 | 45304001 | 45305000 | 1000 | 1 | 4.65E-05 | -1.0783258 | 13 | 1.3 | | |
| DMR1:45404001 | 1 | 45404001 | 45406000 | 2000 | 1 | 3.63E-05 | -1.4864352 | 49 | 2.45 | AABR07001452.1 | |
| DMR1:48282001 | 1 | 48282001 | 48284000 | 2000 | 1 | 1.92E-06 | 0.7532671 | 25 | 1.25 | Slc22a1 | Metabolism |
| DMR1:48974001 | 1 | 48974001 | 48975000 | 1000 | 1 | 4.05E-05 | -0.5716136 | 31 | 3.1 | | |
| DMR1:49378001 | 1 | 49378001 | 49379000 | 1000 | 1 | 4.67E-05 | -0.8669466 | 17 | 1.7 | AABR07001519.1 | |
| DMR1:49934001 | 1 | 49934001 | 49935000 | 1000 | 1 | 2.99E-05 | -1.042203 | 11 | 1.1 | | |
| DMR1:52679001 | 1 | 52679001 | 52680000 | 1000 | 1 | 2.15E-06 | -0.8587429 | 14 | 1.4 | | |
| DMR1:54852001 | 1 | 54852001 | 54853000 | 1000 | 1 | 5.81E-05 | 0.5395217 | 18 | 1.8 | Smok2a | Unknown |
| DMR1:60365001 | 1 | 60365001 | 60367000 | 2000 | 1 | 1.48E-05 | -0.692173 | 65 | 3.25 | | |
| DMR1:61212001 | 1 | 61212001 | 61213000 | 1000 | 1 | 9.58E-05 | 0.6477501 | 14 | 1.4 | AABR07001888.1 | |
| DMR1:63563001 | 1 | 63563001 | 63565000 | 2000 | 1 | 6.63E-05 | 0.4418251 | 19 | 0.95 | Vom2r26 | |
| DMR1:74954001 | 1 | 74954001 | 74955000 | 1000 | 1 | 2.20E-06 | 0.8293128 | 4 | 0.4 | | |
| DMR1:75048001 | 1 | 75048001 | 75051000 | 3000 | 1 | 1.41E-05 | 1.095997 | 9 | 0.3 | | |
| DMR1:81230001 | 1 | 81230001 | 81231000 | 1000 | 1 | 8.37E-06 | -0.8410121 | 26 | 2.6 | Kcnn4 | Transport |
| DMR1:84290001 | 1 | 84290001 | 84291000 | 1000 | 1 | 2.66E-05 | -1.3429122 | 12 | 1.2 | Sertad3;Sertad1 | Transcription |
| DMR1:85075001 | 1 | 85075001 | 85076000 | 1000 | 1 | 4.97E-05 | 0.7857465 | 20 | 2 | Fcgbpl1 | |
| DMR1:86788001 | 1 | 86788001 | 86790000 | 2000 | 1 | 5.96E-05 | 0.7895447 | 10 | 0.5 | | |
| DMR1:88792001 | 1 | 88792001 | 88793000 | 1000 | 1 | 8.16E-05 | 0.7388093 | 14 | 1.4 | | |
| DMR1:91415001 | 1 | 91415001 | 91417000 | 2000 | 1 | 2.61E-05 | -1.2729511 | 32 | 1.6 | | |
| DMR1:100783001 | 1 | 100783001 | 100784000 | 1000 | 1 | 7.79E-05 | -0.6163343 | 14 | 1.4 | Vrk3 | Signaling |
| DMR1:102099001 | 1 | 102099001 | 102100000 | 1000 | 1 | 1.04E-05 | -0.9825296 | 15 | 1.5 | AC120807.1;Kcnj11 | Transport |
| DMR1:103785001 | 1 | 103785001 | 103786000 | 1000 | 1 | 7.68E-05 | -0.4747018 | 34 | 3.4 | | |
| DMR1:128882001 | 1 | 128882001 | 128883000 | 1000 | 1 | 2.80E-05 | -0.6657967 | 22 | 2.2 | | |
| DMR1:129616001 | 1 | 129616001 | 129617000 | 1000 | 1 | 7.41E-07 | -1.4358057 | 10 | 1 | AABR07004190.1 | |
| DMR1:142095001 | 1 | 142095001 | 142096000 | 1000 | 1 | 5.95E-05 | -1.3173318 | 8 | 0.8 | Prc1 | Cytoskeleton |
| DMR1:143530001 | 1 | 143530001 | 143531000 | 1000 | 1 | 8.47E-07 | -0.6793773 | 27 | 2.7 | Homer2 | Signaling |
| DMR1:144612001 | 1 | 144612001 | 144613000 | 1000 | 1 | 5.82E-05 | 1.0097339 | 9 | 0.9 | Efl1 | |
| DMR1:147671001 | 1 | 147671001 | 147673000 | 2000 | 1 | 7.59E-05 | 1.0956913 | 13 | 0.65 | | |
| DMR1:157028001 | 1 | 157028001 | 157029000 | 1000 | 1 | 5.46E-05 | -0.6566651 | 4 | 0.4 | Dlg2 | |
| DMR1:163454001 | 1 | 163454001 | 163455000 | 1000 | 1 | 7.30E-05 | -0.8946721 | 22 | 2.2 | Lrrc32;AABR07004868.2;U1 | Cytoskeleton |
| DMR1:164368001 | 1 | 164368001 | 164370000 | 2000 | 1 | 9.70E-05 | 0.9026999 | 15 | 0.75 | | |
| DMR1:164406001 | 1 | 164406001 | 164407000 | 1000 | 1 | 2.36E-05 | -1.1265408 | 18 | 1.8 | Gdpd5 | Metabolism |
| DMR1:164806001 | 1 | 164806001 | 164807000 | 1000 | 1 | 3.13E-05 | 0.9380223 | 12 | 1.2 | Neu3;AABR07004876.1;AABR07004 | Development |
| DMR1:179684001 | 1 | 179684001 | 179685000 | 1000 | 1 | 7.99E-05 | 0.6485341 | 4 | 0.4 | | |
| DMR1:183573001 | 1 | 183573001 | 183575000 | 2000 | 1 | 7.27E-05 | 0.8591219 | 27 | 1.35 | | |
| DMR1:187733001 | 1 | 187733001 | 187734000 | 1000 | 1 | 3.92E-05 | -0.8665003 | 18 | 1.8 | | |
| DMR1:209371001 | 1 | 209371001 | 209372000 | 1000 | 1 | 1.88E-05 | 0.9262014 | 14 | 1.4 | Mgmt | Transcription |
| DMR1:209541001 | 1 | 209541001 | 209542000 | 1000 | 1 | 3.98E-05 | 0.7943269 | 19 | 1.9 | Ebf3 | |
| DMR1:210163001 | 1 | 210163001 | 210164000 | 1000 | 1 | 2.17E-07 | 0.8100795 | 13 | 1.3 | | |
| DMR1:211210001 | 1 | 211210001 | 211211000 | 1000 | 1 | 2.16E-05 | 0.9865046 | 7 | 0.7 | Ppp2r2d | Signaling |
| DMR1:213848001 | 1 | 213848001 | 213849000 | 1000 | 1 | 2.51E-05 | 0.7963974 | 14 | 1.4 | | |
| DMR1:221797001 | 1 | 221797001 | 221798000 | 1000 | 1 | 1.65E-05 | 0.7832989 | 16 | 1.6 | Rasgrp2;Nrxn2 | Signaling;Receptor |
| DMR1:235261001 | 1 | 235261001 | 235262000 | 1000 | 1 | 4.18E-08 | -1.1411597 | 12 | 1.2 | Gna14;7SK | Signaling |
| DMR1:252513001 | 1 | 252513001 | 252514000 | 1000 | 1 | 3.91E-06 | -1.0449783 | 16 | 1.6 | Stambpl1 | Growth Factors & Cytokines |
| DMR1:256762001 | 1 | 256762001 | 256763000 | 1000 | 1 | 4.04E-05 | 0.7654457 | 16 | 1.6 | Cep55 | |
| DMR1:259407001 | 1 | 259407001 | 259409000 | 2000 | 1 | 4.17E-05 | 0.9174224 | 31 | 1.55 | Sorbs1 | Cytoskeleton |
| DMR1:260904001 | 1 | 260904001 | 260905000 | 1000 | 1 | 6.37E-05 | -0.8286753 | 22 | 2.2 | Slit1 | Receptor |
| DMR1:274020001 | 1 | 274020001 | 274021000 | 1000 | 1 | 2.10E-05 | 0.9543078 | 26 | 2.6 | Mxi1 | Transcription |
| DMR1:276046001 | 1 | 276046001 | 276048000 | 2000 | 1 | 3.90E-05 | -0.4914977 | 47 | 2.35 | | |
| DMR2:5738001 | 2 | 5738001 | 5739000 | 1000 | 1 | 3.23E-06 | -0.859373 | 16 | 1.6 | | |
| DMR2:15494001 | 2 | 15494001 | 15495000 | 1000 | 1 | 8.48E-05 | 0.8358327 | 4 | 0.4 | | |
| DMR2:26347001 | 2 | 26347001 | 26348000 | 1000 | 1 | 6.40E-05 | -0.5388306 | 19 | 1.9 | Iqgap2 | Signaling |
| DMR2:26358001 | 2 | 26358001 | 26359000 | 1000 | 1 | 3.45E-05 | -0.9208916 | 27 | 2.7 | Iqgap2 | Signaling |
| DMR2:29686001 | 2 | 29686001 | 29687000 | 1000 | 1 | 3.04E-06 | -0.7229829 | 49 | 4.9 | Map1b | Cytoskeleton |
| DMR2:32740001 | 2 | 32740001 | 32742000 | 2000 | 1 | 1.45E-05 | -0.8085631 | 31 | 1.55 | | |
| DMR2:39440001 | 2 | 39440001 | 39441000 | 1000 | 1 | 9.61E-05 | -0.72931 | 7 | 0.7 | Ndufaf2;Ercc8 | |
| DMR2:44261001 | 2 | 44261001 | 44262000 | 1000 | 1 | 1.69E-05 | 1.0394735 | 7 | 0.7 | AABR07008118.1 | |

| | | | | | | | | | | | |
|---|---|---|---|---|---|---|---|---|---|---|---|
| DMR2:49171001 | 2 | 49171001 | 49173000 | 2000 | 2 | 4.34E-07 | -1.0643053 | 29 | 1.45 | Parp8 | Unknown |
| DMR2:74343001 | 2 | 74343001 | 74344000 | 1000 | 1 | 2.69E-05 | 0.962528 | 12 | 1.2 | | |
| DMR2:83751001 | 2 | 83751001 | 83753000 | 2000 | 1 | 2.50E-05 | 0.8867971 | 25 | 1.25 | Ctnnd2 | Extracellular Matrix |
| DMR2:98006001 | 2 | 98006001 | 98007000 | 1000 | 1 | 9.77E-05 | 0.6802586 | 7 | 0.7 | | |
| DMR2:112315001 | 2 | 112315001 | 112316000 | 1000 | 1 | 8.59E-05 | 0.9217167 | 15 | 1.5 | Spata16 | |
| DMR2:124489001 | 2 | 124489001 | 124490000 | 1000 | 1 | 1.10E-05 | 1.141537 | 10 | 1 | | |
| DMR2:135488001 | 2 | 135488001 | 135489000 | 1000 | 1 | 4.33E-07 | 1.0384242 | 10 | 1 | | |
| DMR2:160252001 | 2 | 160252001 | 160253000 | 1000 | 1 | 9.71E-05 | 0.3351165 | 12 | 1.2 | | |
| DMR2:161678001 | 2 | 161678001 | 161679000 | 1000 | 1 | 7.83E-05 | 0.9012595 | 15 | 1.5 | | |
| DMR2:169788001 | 2 | 169788001 | 169789000 | 1000 | 1 | 4.08E-05 | 0.9359464 | 7 | 0.7 | | |
| DMR2:198106001 | 2 | 198106001 | 198108000 | 2000 | 1 | 7.84E-05 | 1.0584872 | 34 | 1.7 | Plekho1 | Signaling |
| DMR2:217089001 | 2 | 217089001 | 217090000 | 1000 | 1 | 9.59E-05 | 0.6868302 | 10 | 1 | | |
| DMR2:220331001 | 2 | 220331001 | 220332000 | 1000 | 1 | 5.11E-06 | 1.1327072 | 8 | 0.8 | Plppr4 | |
| DMR2:228163001 | 2 | 228163001 | 228164000 | 1000 | 1 | 8.68E-05 | 0.6503711 | 7 | 0.7 | | |
| DMR2:231649001 | 2 | 231649001 | 231650000 | 1000 | 1 | 9.54E-06 | -0.4992341 | 24 | 2.4 | Ank2 | Cytoskeleton |
| DMR2:251234001 | 2 | 251234001 | 251235000 | 1000 | 1 | 6.88E-05 | -0.958942 | 18 | 1.8 | Col24a1 | Unknown |
| DMR2:251506001 | 2 | 251506001 | 251508000 | 2000 | 1 | 1.20E-05 | -0.5896881 | 38 | 1.9 | | |
| DMR2:262437001 | 2 | 262437001 | 262438000 | 1000 | 1 | 4.53E-06 | -0.9034789 | 8 | 0.8 | | |
| DMR3:1697001 | 3 | 1697001 | 1698000 | 1000 | 1 | 1.62E-05 | -0.425945 | 43 | 4.3 | | |
| DMR3:2863001 | 3 | 2863001 | 2864000 | 1000 | 1 | 4.26E-05 | -0.844242 | 14 | 1.4 | Fcna;Lcn8 | Binding Protein |
| DMR3:6797001 | 3 | 6797001 | 6798000 | 1000 | 1 | 5.37E-05 | -0.5571079 | 46 | 4.6 | Olfm1 | Receptor |
| DMR3:8798001 | 3 | 8798001 | 8799000 | 1000 | 1 | 7.00E-05 | -0.7141338 | 17 | 1.7 | Lrrc8a | Unknown |
| DMR3:23643001 | 3 | 23643001 | 23644000 | 1000 | 1 | 4.62E-06 | 1.0828784 | 8 | 0.8 | | |
| DMR3:24657001 | 3 | 24657001 | 24659000 | 2000 | 1 | 2.85E-05 | 1.0590026 | 34 | 1.7 | | |
| DMR3:35191001 | 3 | 35191001 | 35193000 | 2000 | 1 | 3.55E-05 | -0.880228 | 34 | 1.7 | Kif5c | Cytoskeleton |
| DMR3:55210001 | 3 | 55210001 | 55211000 | 1000 | 1 | 5.61E-05 | -0.5097923 | 28 | 2.8 | Cers6 | |
| DMR3:59621001 | 3 | 59621001 | 59622000 | 1000 | 1 | 7.13E-05 | 0.8833854 | 23 | 2.3 | | |
| DMR3:59637001 | 3 | 59637001 | 59638000 | 1000 | 1 | 4.00E-08 | -1.5210852 | 10 | 1 | Sp3 | Transcription |
| DMR3:62105001 | 3 | 62105001 | 62106000 | 1000 | 1 | 2.97E-05 | -0.6448954 | 23 | 2.3 | | |
| DMR3:64258001 | 3 | 64258001 | 64259000 | 1000 | 1 | 5.69E-05 | -0.8864341 | 20 | 2 | | |
| DMR3:66449001 | 3 | 66449001 | 66450000 | 1000 | 1 | 9.35E-05 | -0.5527614 | 9 | 0.9 | | |
| DMR3:75422001 | 3 | 75422001 | 75424000 | 2000 | 1 | 2.89E-05 | 0.5485954 | 23 | 1.15 | Olr560;AC118490.1 | |
| DMR3:79907001 | 3 | 79907001 | 79908000 | 1000 | 1 | 8.03E-05 | -0.7280363 | 17 | 1.7 | | |
| DMR3:80462001 | 3 | 80462001 | 80463000 | 1000 | 1 | 6.44E-05 | 0.9674613 | 10 | 1 | Ckap5 | Cytoskeleton |
| DMR3:88674001 | 3 | 88674001 | 88675000 | 1000 | 1 | 5.07E-05 | -1.0350895 | 4 | 0.4 | | |
| DMR3:91238001 | 3 | 91238001 | 91239000 | 1000 | 1 | 7.31E-05 | -0.5562189 | 59 | 5.9 | | |
| DMR3:97585001 | 3 | 97585001 | 97586000 | 1000 | 1 | 6.69E-06 | 0.8506332 | 13 | 1.3 | | |
| DMR3:115611001 | 3 | 115611001 | 115612000 | 1000 | 1 | 1.46E-05 | 1.0056818 | 3 | 0.3 | AABR07053596.1 | |
| DMR3:128764001 | 3 | 128764001 | 128765000 | 1000 | 1 | 2.44E-05 | -0.6262555 | 14 | 1.4 | Plcb4 | Metabolism |
| DMR3:140723001 | 3 | 140723001 | 140724000 | 1000 | 1 | 8.50E-06 | -1.0853065 | 14 | 1.4 | Ralgapa2 | Signaling |
| DMR3:149852001 | 3 | 149852001 | 149853000 | 1000 | 1 | 6.32E-06 | -0.5671682 | 35 | 3.5 | Cdk5rap1 | Signaling |
| DMR3:152581001 | 3 | 152581001 | 152582000 | 1000 | 1 | 7.14E-05 | -1.0451241 | 41 | 4.1 | LOC100911769 | |
| DMR3:153142001 | 3 | 153142001 | 153144000 | 2000 | 1 | 8.15E-05 | -0.7977844 | 42 | 2.1 | Soga1 | |
| DMR3:160865001 | 3 | 160865001 | 160866000 | 1000 | 1 | 7.03E-05 | -0.555856 | 20 | 2 | Rbpjl;Sdc4 | Transcription;Cytoskeleton |
| DMR3:165369001 | 3 | 165369001 | 165370000 | 1000 | 1 | 2.31E-05 | 0.9164526 | 16 | 1.6 | Nfatc2 | Transcription |
| DMR3:166531001 | 3 | 166531001 | 166532000 | 1000 | 1 | 5.48E-05 | -1.2947155 | 40 | 4 | | |
| DMR3:168840001 | 3 | 168840001 | 168841000 | 1000 | 1 | 5.50E-05 | -0.5080949 | 23 | 2.3 | | |
| DMR3:169188001 | 3 | 169188001 | 169190000 | 2000 | 1 | 1.20E-05 | 0.8682252 | 28 | 1.4 | | |
| DMR4:11490001 | 4 | 11490001 | 11491000 | 1000 | 1 | 2.80E-05 | 0.9169802 | 5 | 0.5 | Magi2 | Metabolism |
| DMR4:18466001 | 4 | 18466001 | 18467000 | 1000 | 1 | 5.27E-05 | -0.8446736 | 7 | 0.7 | | |
| DMR4:27154001 | 4 | 27154001 | 27155000 | 1000 | 1 | 7.40E-06 | 0.9780839 | 11 | 1.1 | | |
| DMR4:27308001 | 4 | 27308001 | 27309000 | 1000 | 1 | 1.94E-05 | -0.9136146 | 12 | 1.2 | Akap9 | Signaling |
| DMR4:28392001 | 4 | 28392001 | 28393000 | 1000 | 1 | 2.40E-05 | -1.3580823 | 11 | 1.1 | | |
| DMR4:29303001 | 4 | 29303001 | 29304000 | 1000 | 1 | 2.59E-05 | 0.7120811 | 7 | 0.7 | | |
| DMR4:32768001 | 4 | 32768001 | 32769000 | 1000 | 1 | 7.11E-08 | 0.9434094 | 3 | 0.3 | | |
| DMR4:42266001 | 4 | 42266001 | 42267000 | 1000 | 1 | 7.90E-05 | 0.8947677 | 5 | 0.5 | Mdfic | Transcription |
| DMR4:44582001 | 4 | 44582001 | 44583000 | 1000 | 1 | 6.73E-05 | 0.9420506 | 6 | 0.6 | Cav2 | Epigenetic |
| DMR4:47290001 | 4 | 47290001 | 47291000 | 1000 | 1 | 3.43E-05 | 0.680542 | 12 | 1.2 | AABR07059974.1 | |
| DMR4:54798001 | 4 | 54798001 | 54799000 | 1000 | 1 | 5.22E-05 | -1.1119372 | 4 | 0.4 | Grm8 | Receptor |
| DMR4:57908001 | 4 | 57908001 | 57909000 | 1000 | 1 | 2.21E-06 | -0.7898886 | 26 | 2.6 | Cpa4 | Proteolysis |
| DMR4:58844001 | 4 | 58844001 | 58845000 | 1000 | 1 | 1.94E-05 | -1.1197072 | 6 | 0.6 | Podxl | Metabolism |
| DMR4:69248001 | 4 | 69248001 | 69249000 | 1000 | 1 | 9.45E-05 | 0.9216919 | 6 | 0.6 | | |
| DMR4:80753001 | 4 | 80753001 | 80754000 | 1000 | 1 | 8.45E-05 | -0.5434015 | 19 | 1.9 | | |
| DMR4:81338001 | 4 | 81338001 | 81340000 | 2000 | 1 | 1.01E-05 | -0.7935857 | 26 | 1.3 | Snx10 | Transport |
| DMR4:81493001 | 4 | 81493001 | 81495000 | 2000 | 1 | 1.11E-05 | -0.8976488 | 37 | 1.85 | | |
| DMR4:84224001 | 4 | 84224001 | 84225000 | 1000 | 1 | 4.04E-07 | -0.9405398 | 18 | 1.8 | Chn2 | Signaling |
| DMR4:109689001 | 4 | 109689001 | 109690000 | 1000 | 1 | 6.30E-05 | 0.7470766 | 6 | 0.6 | | |
| DMR4:130281001 | 4 | 130281001 | 130282000 | 1000 | 1 | 1.96E-05 | -0.7030733 | 18 | 1.8 | Mitf | Transcription |

| | | | | | | | | | | | |
|---|---|---|---|---|---|---|---|---|---|---|---|
| DMR4:137241001 | 4 | 137241001 | 137243000 | 2000 | 1 | 4.16E-05 | -0.6877757 | 56 | 2.8 | | |
| DMR4:139748001 | 4 | 139748001 | 139749000 | 1000 | 1 | 9.71E-05 | 0.9232665 | 20 | 2 | | |
| DMR4:157355001 | 4 | 157355001 | 157356000 | 1000 | 1 | 7.25E-05 | -0.7250299 | 24 | 2.4 | Usp5;Cdca3;Gnb3;P3h3 | Protease;Cell Cycle;Signaling |
| DMR4:171519001 | 4 | 171519001 | 171520000 | 1000 | 1 | 2.32E-05 | -1.1227422 | 18 | 1.8 | Eps8 | Signaling |
| DMR4:174682001 | 4 | 174682001 | 174684000 | 2000 | 1 | 9.11E-05 | -0.658864 | 28 | 1.4 | Plekha5 | Signaling |
| DMR4:178134001 | 4 | 178134001 | 178135000 | 1000 | 1 | 3.92E-05 | -0.5621895 | 20 | 2 | Sox5 | Transcription |
| DMR5:5001 | 5 | 5001 | 7000 | 2000 | 1 | 5.56E-06 | 1.0616279 | 51 | 2.55 | | |
| DMR5:462001 | 5 | 462001 | 463000 | 1000 | 1 | 3.62E-05 | -0.8669001 | 33 | 3.3 | 7SK | |
| DMR5:10880001 | 5 | 10880001 | 10881000 | 1000 | 1 | 2.45E-05 | 0.8676455 | 7 | 0.7 | | |
| DMR5:12232001 | 5 | 12232001 | 12233000 | 1000 | 1 | 4.52E-05 | -0.8946669 | 12 | 1.2 | | |
| DMR5:21019001 | 5 | 21019001 | 21020000 | 1000 | 1 | 3.37E-05 | 0.8359882 | 3 | 0.3 | | |
| DMR5:21948001 | 5 | 21948001 | 21949000 | 1000 | 1 | 6.76E-05 | 0.7303675 | 26 | 2.6 | Chd7 | Epigenetic |
| DMR5:22138001 | 5 | 22138001 | 22139000 | 1000 | 1 | 6.35E-05 | 0.6743981 | 14 | 1.4 | | |
| DMR5:52649001 | 5 | 52649001 | 52650000 | 1000 | 1 | 6.56E-05 | 0.7084585 | 4 | 0.4 | AABR07047899.1 | |
| DMR5:59965001 | 5 | 59965001 | 59966000 | 1000 | 1 | 6.75E-05 | 0.8328127 | 11 | 1.1 | | |
| DMR5:60519001 | 5 | 60519001 | 60521000 | 2000 | 1 | 5.00E-05 | 0.6659418 | 23 | 1.15 | AABR07048075.1;AABR07048075.2;Grhpr | |
| DMR5:62357001 | 5 | 62357001 | 62358000 | 1000 | 1 | 2.84E-05 | 1.0742529 | 20 | 2 | Gabbr2 | Receptor |
| DMR5:62392001 | 5 | 62392001 | 62393000 | 1000 | 1 | 4.03E-05 | -0.7665441 | 21 | 2.1 | Gabbr2 | Receptor |
| DMR5:68459001 | 5 | 68459001 | 68460000 | 1000 | 1 | 7.05E-05 | 0.5947481 | 7 | 0.7 | | |
| DMR5:107321001 | 5 | 107321001 | 107322000 | 1000 | 1 | 1.41E-06 | 1.2981107 | 10 | 1 | Klhl9;AABR07049134.1 | Transcription |
| DMR5:113044001 | 5 | 113044001 | 113045000 | 1000 | 1 | 6.76E-06 | -0.7569138 | 52 | 5.2 | | |
| DMR5:122445001 | 5 | 122445001 | 122446000 | 1000 | 1 | 3.62E-05 | -0.8527197 | 19 | 1.9 | Sgip1 | |
| DMR5:123060001 | 5 | 123060001 | 123061000 | 1000 | 1 | 5.76E-05 | -0.6455811 | 47 | 4.7 | 5_8S_rRNA | |
| DMR5:143945001 | 5 | 143945001 | 143946000 | 1000 | 1 | 8.86E-05 | 0.920835 | 3 | 0.3 | | |
| DMR5:144327001 | 5 | 144327001 | 144328000 | 1000 | 1 | 5.76E-05 | 0.7293299 | 8 | 0.8 | Col8a2;Adprhl2 | Extracellular Matrix;Metabolism |
| DMR5:147663001 | 5 | 147663001 | 147664000 | 1000 | 1 | 1.79E-05 | 0.7937576 | 4 | 0.4 | Bsdc1;AC132627.2 | |
| DMR5:148207001 | 5 | 148207001 | 148208000 | 1000 | 1 | 8.21E-05 | -0.4203221 | 16 | 1.6 | Adgrb2 | |
| DMR5:150395001 | 5 | 150395001 | 150396000 | 1000 | 1 | 3.87E-05 | 0.9978525 | 9 | 0.9 | Ythdf2;Gmeb1 | Transcription |
| DMR5:151051001 | 5 | 151051001 | 151052000 | 1000 | 1 | 6.80E-05 | 0.9722965 | 14 | 1.4 | Stx12 | Transport |
| DMR5:152455001 | 5 | 152455001 | 152456000 | 1000 | 1 | 5.51E-06 | -0.8722963 | 20 | 2 | Catsper4;Cnksr1;Zfp593 | Transport;Signaling;Transcription |
| DMR5:159559001 | 5 | 159559001 | 159560000 | 1000 | 1 | 4.93E-05 | -0.9821745 | 29 | 2.9 | Crocc | Signaling |
| DMR5:160009001 | 5 | 160009001 | 160010000 | 1000 | 1 | 1.87E-05 | -0.9280504 | 19 | 1.9 | Zbtb17;Spen | Transcription |
| DMR5:165498001 | 5 | 165498001 | 165500000 | 2000 | 1 | 9.65E-05 | -1.0207033 | 50 | 2.5 | | |
| DMR5:167938001 | 5 | 167938001 | 167939000 | 1000 | 1 | 3.51E-05 | 0.8726514 | 14 | 1.4 | | |
| DMR5:168097001 | 5 | 168097001 | 168099000 | 2000 | 1 | 1.41E-05 | -0.6152862 | 38 | 1.9 | Per3 | Development |
| DMR5:168453001 | 5 | 168453001 | 168454000 | 1000 | 1 | 6.26E-06 | -0.7472994 | 10 | 1 | Camta1 | Transcription |
| DMR5:171094001 | 5 | 171094001 | 171096000 | 2000 | 1 | 6.46E-05 | -0.6654059 | 50 | 2.5 | | |
| DMR5:171499001 | 5 | 171499001 | 171500000 | 1000 | 1 | 9.75E-05 | -0.8407595 | 19 | 1.9 | LOC100911486 | Growth Factors & Cytokines |
| DMR6:419001 | 6 | 419001 | 420000 | 1000 | 1 | 1.83E-05 | -0.8307121 | 21 | 2.1 | | |
| DMR6:831001 | 6 | 831001 | 833000 | 2000 | 1 | 2.88E-06 | -0.6145893 | 46 | 2.3 | Crim1 | Development |
| DMR6:6682001 | 6 | 6682001 | 6684000 | 2000 | 1 | 3.44E-05 | 0.9975215 | 28 | 1.4 | Eml4 | Cytoskeleton |
| DMR6:8797001 | 6 | 8797001 | 8798000 | 1000 | 1 | 4.98E-05 | -0.8040489 | 22 | 2.2 | | |
| DMR6:10465001 | 6 | 10465001 | 10468000 | 3000 | 2 | 2.51E-05 | -0.8449755 | 130 | 4.333 | | |
| DMR6:10805001 | 6 | 10805001 | 10809000 | 4000 | 2 | 1.46E-05 | -0.7018037 | 138 | 3.45 | | |
| DMR6:10810001 | 6 | 10810001 | 10816000 | 6000 | 1 | 8.01E-05 | -0.5436022 | 254 | 4.233 | | |
| DMR6:22177001 | 6 | 22177001 | 22179000 | 2000 | 1 | 2.27E-05 | 0.9111289 | 45 | 2.25 | Nlrc4 | Signaling |
| DMR6:27145001 | 6 | 27145001 | 27146000 | 1000 | 1 | 9.51E-05 | 0.7888977 | 11 | 1.1 | Kcnk3 | Transport |
| DMR6:40943001 | 6 | 40943001 | 40944000 | 1000 | 1 | 3.73E-05 | 0.8064474 | 21 | 2.1 | | |
| DMR6:42045001 | 6 | 42045001 | 42046000 | 1000 | 1 | 9.68E-06 | -0.5132867 | 19 | 1.9 | | |
| DMR6:43044001 | 6 | 43044001 | 43045000 | 1000 | 1 | 1.42E-05 | -0.7713052 | 19 | 1.9 | Hpcal1 | Signaling |
| DMR6:58579001 | 6 | 58579001 | 58580000 | 1000 | 1 | 5.66E-05 | 0.7822219 | 4 | 0.4 | | |
| DMR6:68092001 | 6 | 68092001 | 68094000 | 2000 | 1 | 8.14E-05 | 0.820205 | 7 | 0.35 | | |
| DMR6:78354001 | 6 | 78354001 | 78355000 | 1000 | 1 | 7.83E-05 | -0.733184 | 7 | 0.7 | Mipol1 | |
| DMR6:93834001 | 6 | 93834001 | 93835000 | 1000 | 1 | 2.89E-07 | 1.0106762 | 12 | 1.2 | | |
| DMR6:101024001 | 6 | 101024001 | 101025000 | 1000 | 1 | 1.72E-05 | -0.9108282 | 6 | 0.6 | | |
| DMR6:106539001 | 6 | 106539001 | 106540000 | 1000 | 1 | 5.49E-08 | -0.9054256 | 15 | 1.5 | Rgs6 | Signaling |
| DMR6:122977001 | 6 | 122977001 | 122978000 | 1000 | 1 | 6.85E-05 | -0.8346186 | 12 | 1.2 | Ttc8 | Metabolism |
| DMR6:128303001 | 6 | 128303001 | 128305000 | 2000 | 1 | 8.58E-05 | -0.9721214 | 47 | 2.35 | | |
| DMR6:137835001 | 6 | 137835001 | 137836000 | 1000 | 1 | 5.27E-05 | 0.6182525 | 5 | 0.5 | Pacs2 | |
| DMR6:141346001 | 6 | 141346001 | 141348000 | 2000 | 1 | 7.27E-05 | -0.5013511 | 77 | 3.85 | | |
| DMR7:12127001 | 7 | 12127001 | 12130000 | 3000 | 1 | 9.01E-05 | -0.7797129 | 119 | 3.967 | | |
| DMR7:15926001 | 7 | 15926001 | 15927000 | 1000 | 1 | 8.86E-06 | 0.6712197 | 4 | 0.4 | | |
| DMR7:63137001 | 7 | 63137001 | 63138000 | 1000 | 1 | 8.97E-05 | -0.7252004 | 21 | 2.1 | Wif1 | Signaling |
| DMR7:63543001 | 7 | 63543001 | 63545000 | 2000 | 1 | 2.92E-05 | -0.7970644 | 58 | 2.9 | Rassf3 | Signaling |
| DMR7:66898001 | 7 | 66898001 | 66899000 | 1000 | 1 | 7.18E-05 | -0.6485175 | 19 | 1.9 | Ppm1h | Signaling |
| DMR7:73330001 | 7 | 73330001 | 73333000 | 3000 | 1 | 6.53E-05 | -0.4324255 | 98 | 3.267 | Nipal2 | Development |

| | | | | | | | | | | | |
|---|---|---|---|---|---|---|---|---|---|---|---|
| DMR7:74980001 | 7 | 74980001 | 74981000 | 1000 | 1 | 1.96E-06 | 0.7390309 | 23 | 2.3 | Polr2k | Transcription |
| DMR7:75949001 | 7 | 75949001 | 75950000 | 1000 | 1 | 5.12E-05 | -0.7663704 | 19 | 1.9 | | |
| DMR7:77471001 | 7 | 77471001 | 77472000 | 1000 | 1 | 8.86E-05 | -0.6536051 | 38 | 3.8 | | |
| DMR7:80695001 | 7 | 80695001 | 80696000 | 1000 | 1 | 2.40E-06 | -0.5628651 | 11 | 1.1 | Oxr1 | Development |
| DMR7:93585001 | 7 | 93585001 | 93587000 | 2000 | 1 | 6.09E-05 | 0.724754 | 15 | 0.75 | | |
| DMR7:97814001 | 7 | 97814001 | 97815000 | 1000 | 1 | 5.51E-06 | -0.7272035 | 22 | 2.2 | Tbc1d31 | |
| DMR7:98230001 | 7 | 98230001 | 98232000 | 2000 | 1 | 5.39E-05 | -1.0819576 | 24 | 1.2 | Anxa13 | Signaling |
| DMR7:101973001 | 7 | 101973001 | 101975000 | 2000 | 1 | 6.78E-05 | 0.7826572 | 17 | 0.85 | AABR07058134.1 | |
| DMR7:114377001 | 7 | 114377001 | 114378000 | 1000 | 1 | 3.18E-05 | -0.6877239 | 24 | 2.4 | Ago2 | Transcription |
| DMR7:120868001 | 7 | 120868001 | 120869000 | 1000 | 1 | 6.01E-05 | -1.0531528 | 10 | 1 | Fam227a | |
| DMR7:122579001 | 7 | 122579001 | 122580000 | 1000 | 1 | 5.52E-05 | 0.7274073 | 10 | 1 | Slc25a17;St13 | Transport;Cytoskeleton |
| DMR7:123397001 | 7 | 123397001 | 123399000 | 2000 | 2 | 3.38E-05 | -0.5873018 | 93 | 4.65 | Srebf2 | Transcription |
| DMR7:127652001 | 7 | 127652001 | 127653000 | 1000 | 1 | 7.68E-05 | -0.8297718 | 14 | 1.4 | | |
| DMR7:127813001 | 7 | 127813001 | 127814000 | 1000 | 1 | 3.53E-05 | 0.7156761 | 11 | 1.1 | | |
| DMR7:133588001 | 7 | 133588001 | 133589000 | 1000 | 1 | 4.00E-07 | 1.2056852 | 7 | 0.7 | Cntn1;AABR07058699.2 | Extracellular Matrix |
| DMR7:134315001 | 7 | 134315001 | 134316000 | 1000 | 1 | 5.80E-05 | -0.8674844 | 25 | 2.5 | | |
| DMR7:136039001 | 7 | 136039001 | 136042000 | 3000 | 1 | 1.72E-05 | -0.8108821 | 51 | 1.7 | AABR07058745.1 | |
| DMR7:136269001 | 7 | 136269001 | 136270000 | 1000 | 1 | 4.93E-05 | -1.3964811 | 9 | 0.9 | Tmem117 | Unknown |
| DMR7:137606001 | 7 | 137606001 | 137607000 | 1000 | 1 | 1.06E-06 | -0.7869565 | 22 | 2.2 | | |
| DMR8:8166001 | 8 | 8166001 | 8167000 | 1000 | 1 | 1.45E-05 | 0.6803422 | 2 | 0.2 | Cntn5 | Extracellular Matrix |
| DMR8:12221001 | 8 | 12221001 | 12222000 | 1000 | 1 | 9.12E-07 | 1.1726826 | 15 | 1.5 | Maml2 | Transcription |
| DMR8:14247001 | 8 | 14247001 | 14248000 | 1000 | 1 | 1.12E-05 | -0.5341318 | 27 | 2.7 | Slc36a4 | Transport |
| DMR8:14259001 | 8 | 14259001 | 14260000 | 1000 | 1 | 7.99E-05 | -0.3241209 | 32 | 3.2 | Slc36a4 | Transport |
| DMR8:24084001 | 8 | 24084001 | 24085000 | 1000 | 1 | 4.20E-05 | 1.0758787 | 14 | 1.4 | Bbs9;AABR07069490.1 | Development |
| DMR8:26263001 | 8 | 26263001 | 26264000 | 1000 | 1 | 6.12E-07 | 1.120088 | 14 | 1.4 | Herpud2 | |
| DMR8:49059001 | 8 | 49059001 | 49060000 | 1000 | 1 | 6.72E-05 | 1.0369123 | 6 | 0.6 | Arcn1 | Transport |
| DMR8:49844001 | 8 | 49844001 | 49845000 | 1000 | 1 | 9.19E-05 | -0.4726951 | 25 | 2.5 | Dscaml1 | Development |
| DMR8:50357001 | 8 | 50357001 | 50358000 | 1000 | 1 | 6.14E-06 | 1.0661877 | 14 | 1.4 | Sik3 | Receptor |
| DMR8:59258001 | 8 | 59258001 | 59259000 | 1000 | 1 | 1.15E-06 | 1.0029332 | 11 | 1.1 | | |
| DMR8:72807001 | 8 | 72807001 | 72808000 | 1000 | 1 | 2.26E-06 | -0.9001308 | 11 | 1.1 | Tpm1 | Cytoskeleton |
| DMR8:78636001 | 8 | 78636001 | 78637000 | 1000 | 1 | 9.14E-05 | -1.1138292 | 3 | 0.3 | Tcf12 | Transcription |
| DMR8:81514001 | 8 | 81514001 | 81515000 | 1000 | 1 | 2.02E-05 | -0.6205482 | 10 | 1 | AABR07070714.1 | |
| DMR8:100424001 | 8 | 100424001 | 100426000 | 2000 | 1 | 1.44E-05 | 1.0914467 | 17 | 0.85 | | |
| DMR8:103458001 | 8 | 103458001 | 103459000 | 1000 | 1 | 8.51E-05 | -0.7999805 | 18 | 1.8 | PCOLCE2 | Signaling |
| DMR8:107296001 | 8 | 107296001 | 107297000 | 1000 | 1 | 9.38E-05 | 1.1155643 | 11 | 1.1 | Pik3cb | Signaling |
| DMR8:109770001 | 8 | 109770001 | 109771000 | 1000 | 1 | 8.14E-05 | 0.9233736 | 13 | 1.3 | AABR07071332.2 | |
| DMR8:112294001 | 8 | 112294001 | 112295000 | 1000 | 1 | 4.15E-06 | -1.1492063 | 18 | 1.8 | | |
| DMR8:114500001 | 8 | 114500001 | 114501000 | 1000 | 1 | 3.36E-05 | 1.0300297 | 13 | 1.3 | | |
| DMR8:115228001 | 8 | 115228001 | 115229000 | 1000 | 1 | 5.57E-05 | -0.4086591 | 40 | 4 | Iqcf3 | |
| DMR8:126200001 | 8 | 126200001 | 126201000 | 1000 | 1 | 1.46E-05 | 1.137069 | 12 | 1.2 | | |
| DMR8:130009001 | 8 | 130009001 | 130010000 | 1000 | 1 | 3.15E-05 | -0.5597061 | 35 | 3.5 | Trak1 | Metabolism |
| DMR9:16257001 | 9 | 16257001 | 16258000 | 1000 | 1 | 3.20E-05 | -0.768421 | 39 | 3.9 | | |
| DMR9:16605001 | 9 | 16605001 | 16606000 | 1000 | 1 | 9.95E-05 | -0.9161264 | 23 | 2.3 | Ppp2r5d;Mea1;Klhdc3 | Signaling;Unknown;Transcription |
| DMR9:18234001 | 9 | 18234001 | 18235000 | 1000 | 1 | 2.26E-06 | -0.7169025 | 28 | 2.8 | | |
| DMR9:21486001 | 9 | 21486001 | 21489000 | 3000 | 1 | 7.37E-05 | 0.51905 | 19 | 0.633 | | |
| DMR9:21495001 | 9 | 21495001 | 21496000 | 1000 | 1 | 9.08E-05 | 0.7967038 | 6 | 0.6 | | |
| DMR9:25707001 | 9 | 25707001 | 25708000 | 1000 | 1 | 3.40E-05 | 0.6529383 | 12 | 1.2 | | |
| DMR9:28332001 | 9 | 28332001 | 28333000 | 1000 | 1 | 6.56E-05 | -0.9586157 | 3 | 0.3 | | |
| DMR9:45481001 | 9 | 45481001 | 45482000 | 1000 | 1 | 5.34E-05 | -0.5372766 | 20 | 2 | Lonrf2 | Proteolysis |
| DMR9:54062001 | 9 | 54062001 | 54065000 | 3000 | 1 | 3.29E-07 | 0.9323059 | 30 | 1 | | |
| DMR9:54614001 | 9 | 54614001 | 54615000 | 1000 | 1 | 1.29E-05 | -0.658807 | 17 | 1.7 | Myo1b | Cytoskeleton |
| DMR9:62113001 | 9 | 62113001 | 62114000 | 1000 | 1 | 1.26E-06 | -1.2079086 | 8 | 0.8 | | |
| DMR9:70640001 | 9 | 70640001 | 70641000 | 1000 | 1 | 2.04E-05 | 1.0143718 | 9 | 0.9 | | |
| DMR9:89269001 | 9 | 89269001 | 89270000 | 1000 | 1 | 2.52E-05 | 0.6741744 | 13 | 1.3 | | |
| DMR9:95818001 | 9 | 95818001 | 95819000 | 1000 | 1 | 3.56E-06 | 1.1402345 | 12 | 1.2 | | |
| DMR9:98048001 | 9 | 98048001 | 98052000 | 4000 | 1 | 6.16E-05 | -0.6355326 | 192 | 4.8 | | |
| DMR9:110130001 | 9 | 110130001 | 110131000 | 1000 | 1 | 9.45E-07 | 1.166417 | 18 | 1.8 | Efna5 | Signaling |
| DMR9:111576001 | 9 | 111576001 | 111577000 | 1000 | 1 | 6.00E-05 | 0.9311375 | 13 | 1.3 | | |
| DMR9:119965001 | 9 | 119965001 | 119966000 | 1000 | 1 | 1.10E-06 | 0.8794781 | 4 | 0.4 | | |
| DMR9:120741001 | 9 | 120741001 | 120742000 | 1000 | 1 | 7.96E-05 | 0.7371273 | 13 | 1.3 | | |
| DMR10:7591001 | 10 | 7591001 | 7592000 | 1000 | 1 | 1.77E-05 | -0.7911835 | 21 | 2.1 | AABR07029107.1 | |
| DMR10:7901001 | 10 | 7901001 | 7902000 | 1000 | 1 | 9.02E-05 | -0.9522485 | 4 | 0.4 | | |
| DMR10:14467001 | 10 | 14467001 | 14468000 | 1000 | 1 | 6.17E-05 | -0.689261 | 19 | 1.9 | Ift140;Telo2 | Cytoskeleton |
| DMR10:17462001 | 10 | 17462001 | 17464000 | 2000 | 1 | 7.26E-05 | 0.7353021 | 9 | 0.45 | Stk10 | Signaling |
| DMR10:21237001 | 10 | 21237001 | 21238000 | 1000 | 1 | 1.29E-05 | -0.6875842 | 18 | 1.8 | Tenm2 | |
| DMR10:23572001 | 10 | 23572001 | 23573000 | 1000 | 1 | 8.67E-05 | -0.5425212 | 20 | 2 | | |
| DMR10:28664001 | 10 | 28664001 | 28665000 | 1000 | 1 | 1.03E-05 | 1.0302417 | 5 | 0.5 | Atp10b | Transport |

| | | | | | | | | | | | |
|---|---|---|---|---|---|---|---|---|---|---|---|
| DMR10:42557001 | 10 | 42557001 | 42558000 | 1000 | 1 | 2.53E-05 | 0.7651667 | 12 | 1.2 | | |
| DMR10:42980001 | 10 | 42980001 | 42981000 | 1000 | 1 | 5.00E-05 | -0.8899079 | 18 | 1.8 | | |
| DMR10:50117001 | 10 | 50117001 | 50118000 | 1000 | 1 | 1.10E-05 | -1.1289016 | 11 | 1.1 | | |
| DMR10:51431001 | 10 | 51431001 | 51432000 | 1000 | 1 | 3.03E-05 | -1.0006785 | 12 | 1.2 | | |
| DMR10:52923001 | 10 | 52923001 | 52924000 | 1000 | 1 | 8.91E-05 | 0.7350091 | 9 | 0.9 | Shisa6 | Development |
| DMR10:71903001 | 10 | 71903001 | 71904000 | 1000 | 1 | 2.80E-05 | -0.7542934 | 15 | 1.5 | | |
| DMR10:74594001 | 10 | 74594001 | 74595000 | 1000 | 1 | 9.57E-05 | -0.9395765 | 7 | 0.7 | Ppm1e | Signaling |
| DMR10:79456001 | 10 | 79456001 | 79457000 | 1000 | 1 | 2.74E-05 | -1.3002999 | 5 | 0.5 | | |
| DMR10:85400001 | 10 | 85400001 | 85402000 | 2000 | 1 | 9.40E-05 | -0.6013985 | 32 | 1.6 | Arhgap23 | Signaling |
| DMR10:90422001 | 10 | 90422001 | 90423000 | 1000 | 1 | 9.45E-08 | -0.9412266 | 13 | 1.3 | Itga2b;Gpatch8 | Extracellular Matrix |
| DMR10:90599001 | 10 | 90599001 | 90600000 | 1000 | 1 | 4.32E-07 | -1.1526799 | 24 | 2.4 | | |
| DMR10:92188001 | 10 | 92188001 | 92189000 | 1000 | 1 | 6.54E-05 | -0.936042 | 21 | 2.1 | Crhr1 | Receptor |
| DMR10:94580001 | 10 | 94580001 | 94582000 | 2000 | 1 | 2.48E-05 | -0.5207835 | 49 | 2.45 | Icam2;Ern1 | Signaling |
| DMR10:97350001 | 10 | 97350001 | 97352000 | 2000 | 1 | 5.94E-06 | -0.5989028 | 48 | 2.4 | AABR07030642.1 | |
| DMR10:98498001 | 10 | 98498001 | 98499000 | 1000 | 1 | 1.69E-05 | 0.7674403 | 14 | 1.4 | Abca6 | Transport |
| DMR10:99118001 | 10 | 99118001 | 99119000 | 1000 | 1 | 2.12E-06 | 0.8056166 | 10 | 1 | | |
| DMR10:102111001 | 10 | 102111001 | 102112000 | 1000 | 1 | 1.64E-05 | -0.909901 | 23 | 2.3 | | |
| DMR10:102259001 | 10 | 102259001 | 102260000 | 1000 | 1 | 7.71E-05 | 1.0547487 | 16 | 1.6 | Cdc42ep4 | Signaling |
| DMR10:103598001 | 10 | 103598001 | 103599000 | 1000 | 1 | 1.71E-05 | -0.6555036 | 48 | 4.8 | Cd300le;Cd300e | Receptor |
| DMR11:540001 | 11 | 540001 | 541000 | 1000 | 1 | 6.31E-05 | -0.7530991 | 6 | 0.6 | Epha3 | Receptor |
| DMR11:15134001 | 11 | 15134001 | 15135000 | 1000 | 1 | 7.91E-05 | 0.6278124 | 9 | 0.9 | | |
| DMR11:28798001 | 11 | 28798001 | 28800000 | 2000 | 1 | 6.64E-05 | -1.0625572 | 20 | 1 | | |
| DMR11:31508001 | 11 | 31508001 | 31511000 | 3000 | 1 | 7.95E-05 | -0.6441733 | 39 | 1.3 | | |
| DMR11:32799001 | 11 | 32799001 | 32800000 | 1000 | 1 | 2.21E-05 | 1.0280587 | 20 | 2 | Runx1 | Transcription |
| DMR11:35658001 | 11 | 35658001 | 35659000 | 1000 | 1 | 9.52E-05 | -0.6940101 | 21 | 2.1 | Erg | Transcription |
| DMR11:51425001 | 11 | 51425001 | 51426000 | 1000 | 1 | 1.52E-05 | 0.7539784 | 6 | 0.6 | | |
| DMR11:66967001 | 11 | 66967001 | 66968000 | 1000 | 1 | 8.79E-05 | -0.6241018 | 9 | 0.9 | Slc15a2 | Metabolism |
| DMR11:69805001 | 11 | 69805001 | 69807000 | 2000 | 1 | 5.19E-05 | -0.8355276 | 45 | 2.25 | Kalrn | Signaling |
| DMR11:71434001 | 11 | 71434001 | 71435000 | 1000 | 1 | 8.53E-05 | -0.7781643 | 13 | 1.3 | U6 | |
| DMR11:71454001 | 11 | 71454001 | 71455000 | 1000 | 1 | 9.43E-05 | -0.6164625 | 13 | 1.3 | | |
| DMR11:79417001 | 11 | 79417001 | 79418000 | 1000 | 1 | 7.72E-05 | -1.0663995 | 13 | 1.3 | Lpp | Cytoskeleton |
| DMR11:79529001 | 11 | 79529001 | 79530000 | 1000 | 1 | 5.29E-06 | -1.02283 | 20 | 2 | Lpp | Cytoskeleton |
| DMR11:79561001 | 11 | 79561001 | 79562000 | 1000 | 1 | 1.04E-05 | 0.7909805 | 14 | 1.4 | Lpp | Cytoskeleton |
| DMR11:80484001 | 11 | 80484001 | 80485000 | 1000 | 1 | 2.63E-05 | 0.9112375 | 12 | 1.2 | | |
| DMR11:82454001 | 11 | 82454001 | 82455000 | 1000 | 1 | 5.55E-05 | -0.8887091 | 19 | 1.9 | | |
| DMR11:84548001 | 11 | 84548001 | 84549000 | 1000 | 1 | 5.59E-05 | -0.9577638 | 5 | 0.5 | Yeats2;Parl;AABR07034697.1 | Transcription;Protease |
| DMR11:87418001 | 11 | 87418001 | 87419000 | 1000 | 1 | 2.95E-05 | -1.08004 | 22 | 2.2 | Lrrc74b | |
| DMR12:46001 | 12 | 46001 | 47000 | 1000 | 1 | 6.76E-05 | 0.3964953 | 3 | 0.3 | AABR07034833.1;AABR07034833.2 | |
| DMR12:1065001 | 12 | 1065001 | 1068000 | 3000 | 1 | 6.07E-06 | -0.7681792 | 46 | 1.533 | Stard13 | Metabolism |
| DMR12:14291001 | 12 | 14291001 | 14292000 | 1000 | 1 | 7.91E-05 | -0.6446486 | 13 | 1.3 | | |
| DMR12:14585001 | 12 | 14585001 | 14586000 | 1000 | 1 | 5.06E-05 | 0.8915947 | 16 | 1.6 | AABR07035437.1 | |
| DMR12:15002001 | 12 | 15002001 | 15003000 | 1000 | 1 | 2.08E-05 | -0.6100734 | 23 | 2.3 | AABR07035437.1 | |
| DMR12:15630001 | 12 | 15630001 | 15633000 | 3000 | 1 | 1.57E-06 | 1.1248439 | 13 | 0.433 | | |
| DMR12:16237001 | 12 | 16237001 | 16238000 | 1000 | 1 | 1.40E-05 | 0.9933304 | 12 | 1.2 | AC117065.1 | |
| DMR12:16761001 | 12 | 16761001 | 16763000 | 2000 | 1 | 6.82E-05 | -0.7065513 | 34 | 1.7 | Elfn1 | Receptor |
| DMR12:26219001 | 12 | 26219001 | 26220000 | 1000 | 1 | 9.12E-05 | 1.1157737 | 14 | 1.4 | | |
| DMR12:28571001 | 12 | 28571001 | 28573000 | 2000 | 1 | 2.65E-06 | -0.7888659 | 14 | 0.7 | Galnt17 | |
| DMR12:32181001 | 12 | 32181001 | 32182000 | 1000 | 1 | 3.81E-05 | -0.6212413 | 13 | 1.3 | Tmem132d | Unknown |
| DMR12:37886001 | 12 | 37886001 | 37887000 | 1000 | 1 | 7.06E-05 | 1.1858392 | 14 | 1.4 | Pitpnm2 | Metabolism |
| DMR12:38728001 | 12 | 38728001 | 38729000 | 1000 | 1 | 5.79E-05 | -0.9733919 | 28 | 2.8 | Wdr66 | Unknown |
| DMR12:38861001 | 12 | 38861001 | 38862000 | 1000 | 1 | 7.01E-05 | 0.7673348 | 20 | 2 | Setd1b | |
| DMR12:41533001 | 12 | 41533001 | 41534000 | 1000 | 1 | 4.01E-05 | -0.8204622 | 20 | 2 | Tpcn1 | Metabolism |
| DMR12:44399001 | 12 | 44399001 | 44401000 | 2000 | 1 | 1.47E-06 | -0.8868965 | 56 | 2.8 | Nos1 | Metabolism |
| DMR12:45833001 | 12 | 45833001 | 45834000 | 1000 | 1 | 6.48E-05 | 0.93869 | 18 | 1.8 | Srrm4 | Translation |
| DMR12:46705001 | 12 | 46705001 | 46708000 | 3000 | 1 | 1.91E-05 | -0.6223392 | 81 | 2.7 | Bicdl1;Rab35 | Signaling |
| DMR13:12984001 | 13 | 12984001 | 12985000 | 1000 | 1 | 6.52E-05 | -0.6118899 | 34 | 3.4 | | |
| DMR13:41552001 | 13 | 41552001 | 41553000 | 1000 | 1 | 6.47E-05 | -1.1319348 | 21 | 2.1 | | |
| DMR13:45263001 | 13 | 45263001 | 45265000 | 2000 | 1 | 9.36E-07 | -0.8317446 | 43 | 2.15 | | |
| DMR13:54461001 | 13 | 54461001 | 54462000 | 1000 | 1 | 2.97E-05 | -1.1303996 | 7 | 0.7 | | |
| DMR13:55430001 | 13 | 55430001 | 55431000 | 1000 | 1 | 2.71E-07 | 1.0399136 | 8 | 0.8 | | |
| DMR13:60250001 | 13 | 60250001 | 60252000 | 2000 | 1 | 1.69E-05 | 0.6484502 | 7 | 0.35 | | |
| DMR13:61615001 | 13 | 61615001 | 61617000 | 2000 | 1 | 3.11E-06 | -0.8660917 | 56 | 2.8 | | |
| DMR13:74726001 | 13 | 74726001 | 74727000 | 1000 | 1 | 2.16E-07 | 0.9800297 | 17 | 1.7 | Tex35 | |
| DMR13:77455001 | 13 | 77455001 | 77456000 | 1000 | 1 | 4.88E-05 | -0.8521023 | 2 | 0.2 | AABR07021536.1 | |
| DMR13:80400001 | 13 | 80400001 | 80401000 | 1000 | 1 | 1.05E-05 | -1.2143901 | 7 | 0.7 | AABR07021596.2 | |
| DMR13:80799001 | 13 | 80799001 | 80801000 | 2000 | 1 | 2.54E-05 | 0.840257 | 23 | 1.15 | Fmo6 | |
| DMR13:84908001 | 13 | 84908001 | 84910000 | 2000 | 1 | 8.80E-06 | -0.7647563 | 29 | 1.45 | AABR07021666.2 | |
| DMR13:93577001 | 13 | 93577001 | 93578000 | 1000 | 1 | 6.47E-05 | 0.7429989 | 6 | 0.6 | | |

| | | | | | | | | | | | |
|---|---|---|---|---|---|---|---|---|---|---|---|
| DMR13:99653001 | 13 | 99653001 | 99654000 | 1000 | 1 | 9.84E-05 | -0.5929679 | 17 | 1.7 | Cnih3 | Signaling |
| DMR13:103837001 | 13 | 103837001 | 103838000 | 1000 | 1 | 3.69E-07 | -0.8941589 | 16 | 1.6 | | |
| DMR13:104983001 | 13 | 104983001 | 104985000 | 2000 | 1 | 3.06E-06 | 1.0026852 | 24 | 1.2 | | |
| DMR13:106234001 | 13 | 106234001 | 106235000 | 1000 | 1 | 1.22E-06 | -0.8859774 | 16 | 1.6 | | |
| DMR13:109691001 | 13 | 109691001 | 109692000 | 1000 | 1 | 3.81E-06 | -0.8191195 | 19 | 1.9 | Nsl1 | |
| DMR13:110195001 | 13 | 110195001 | 110196000 | 1000 | 1 | 9.56E-05 | -0.462764 | 16 | 1.6 | | |
| DMR14:4558001 | 14 | 4558001 | 4560000 | 2000 | 1 | 2.68E-05 | -0.8831827 | 39 | 1.95 | | |
| DMR14:7676001 | 14 | 7676001 | 7678000 | 2000 | 1 | 2.29E-05 | -1.1504838 | 38 | 1.9 | Slc10a6;7SK | Transport |
| DMR14:7708001 | 14 | 7708001 | 7710000 | 2000 | 1 | 7.16E-05 | 0.8725639 | 44 | 2.2 | Ptpn13 | Signaling |
| DMR14:10459001 | 14 | 10459001 | 10460000 | 1000 | 1 | 8.82E-07 | -1.0823615 | 13 | 1.3 | Helq | Transcription |
| DMR14:39102001 | 14 | 39102001 | 39103000 | 1000 | 1 | 9.13E-06 | 1.0169075 | 11 | 1.1 | Gabrb1 | Receptor |
| DMR14:44845001 | 14 | 44845001 | 44846000 | 1000 | 1 | 9.92E-05 | -0.7115146 | 19 | 1.9 | Klhl5 | Cytoskeleton |
| DMR14:45657001 | 14 | 45657001 | 45658000 | 1000 | 1 | 2.71E-05 | -0.8796939 | 10 | 1 | Tbc1d1 | Signaling |
| DMR14:47215001 | 14 | 47215001 | 47216000 | 1000 | 1 | 4.89E-05 | -0.9356525 | 16 | 1.6 | | |
| DMR14:48703001 | 14 | 48703001 | 48704000 | 1000 | 1 | 3.43E-05 | -0.7511851 | 10 | 1 | | |
| DMR14:54147001 | 14 | 54147001 | 54148000 | 1000 | 1 | 4.40E-05 | 0.5329138 | 13 | 1.3 | | |
| DMR14:59952001 | 14 | 59952001 | 59953000 | 1000 | 1 | 3.05E-05 | -0.7824302 | 14 | 1.4 | | |
| DMR14:84073001 | 14 | 84073001 | 84074000 | 1000 | 1 | 8.80E-05 | -1.0398141 | 14 | 1.4 | Osbp2 | Receptor |
| DMR14:91833001 | 14 | 91833001 | 91834000 | 1000 | 1 | 3.90E-05 | 1.0428743 | 9 | 0.9 | Ikzf1 | Transcription |
| DMR14:98121001 | 14 | 98121001 | 98122000 | 1000 | 1 | 8.74E-05 | 0.7241651 | 13 | 1.3 | | |
| DMR14:104268001 | 14 | 104268001 | 104269000 | 1000 | 1 | 2.30E-06 | -0.7257362 | 29 | 2.9 | Spred2 | Signaling |
| DMR14:107084001 | 14 | 107084001 | 107086000 | 2000 | 1 | 1.11E-05 | -0.6902162 | 36 | 1.8 | Ehbp1 | Unknown |
| DMR15:2917001 | 15 | 2917001 | 2918000 | 1000 | 1 | 6.52E-05 | -0.8246895 | 24 | 2.4 | Kat6b | Epigenetic |
| DMR15:4493001 | 15 | 4493001 | 4494000 | 1000 | 1 | 9.66E-05 | -0.9036143 | 28 | 2.8 | | |
| DMR15:9990001 | 15 | 9990001 | 9991000 | 1000 | 1 | 9.27E-05 | 0.7682507 | 12 | 1.2 | | |
| DMR15:10104001 | 15 | 10104001 | 10105000 | 1000 | 1 | 3.99E-05 | -0.7855187 | 20 | 2 | | |
| DMR15:14029001 | 15 | 14029001 | 14030000 | 1000 | 1 | 5.59E-05 | -0.8041585 | 12 | 1.2 | AABR07017159.1 | |
| DMR15:20141001 | 15 | 20141001 | 20142000 | 1000 | 1 | 3.82E-05 | -0.5170915 | 11 | 1.1 | | |
| DMR15:20947001 | 15 | 20947001 | 20948000 | 1000 | 1 | 4.01E-05 | 0.7420312 | 5 | 0.5 | | |
| DMR15:36941001 | 15 | 36941001 | 36942000 | 1000 | 1 | 9.54E-06 | -0.9633383 | 11 | 1.1 | AABR07018038.3;Mphosph8;Pspc1 | Transcription |
| DMR15:42372001 | 15 | 42372001 | 42373000 | 1000 | 1 | 1.20E-05 | -0.7139619 | 10 | 1 | | |
| DMR15:44935001 | 15 | 44935001 | 44936000 | 1000 | 1 | 4.18E-05 | 0.8212905 | 8 | 0.8 | | |
| DMR15:65172001 | 15 | 65172001 | 65175000 | 3000 | 1 | 8.48E-08 | 0.8505893 | 19 | 0.633 | | |
| DMR15:75595001 | 15 | 75595001 | 75596000 | 1000 | 1 | 2.31E-05 | 0.5173599 | 7 | 0.7 | | |
| DMR15:77369001 | 15 | 77369001 | 77370000 | 1000 | 1 | 5.22E-05 | 0.9926899 | 9 | 0.9 | | |
| DMR15:81438001 | 15 | 81438001 | 81440000 | 2000 | 1 | 9.55E-05 | 0.7638531 | 21 | 1.05 | | |
| DMR15:85214001 | 15 | 85214001 | 85215000 | 1000 | 1 | 6.10E-06 | -0.736078 | 15 | 1.5 | | |
| DMR15:90774001 | 15 | 90774001 | 90776000 | 2000 | 1 | 5.38E-05 | -1.268814 | 23 | 1.15 | Mycbp2 | Metabolism |
| DMR15:97620001 | 15 | 97620001 | 97621000 | 1000 | 1 | 1.77E-05 | 0.8232334 | 4 | 0.4 | | |
| DMR15:109686001 | 15 | 109686001 | 109687000 | 1000 | 1 | 2.96E-05 | -1.1016719 | 11 | 1.1 | | |
| DMR16:6555001 | 16 | 6555001 | 6556000 | 1000 | 1 | 7.35E-06 | -1.051944 | 13 | 1.3 | Dcp1a | Transcription |
| DMR16:7602001 | 16 | 7602001 | 7603000 | 1000 | 1 | 5.42E-05 | 1.0822652 | 13 | 1.3 | Eaf1 | Apoptosis |
| DMR16:9373001 | 16 | 9373001 | 9374000 | 1000 | 1 | 8.13E-05 | 0.8075371 | 14 | 1.4 | Wdfy4 | |
| DMR16:11703001 | 16 | 11703001 | 11704000 | 1000 | 1 | 3.76E-06 | -0.8098088 | 19 | 1.9 | Grid1 | Receptor |
| DMR16:14151001 | 16 | 14151001 | 14153000 | 2000 | 1 | 3.72E-05 | -0.5155727 | 38 | 1.9 | Ccser2 | |
| DMR16:19565001 | 16 | 19565001 | 19566000 | 1000 | 1 | 1.02E-05 | 1.0268454 | 7 | 0.7 | | |
| DMR16:20110001 | 16 | 20110001 | 20111000 | 1000 | 1 | 7.37E-05 | 0.8781009 | 15 | 1.5 | AABR07024869.1;Jak3 | Signaling |
| DMR16:20413001 | 16 | 20413001 | 20414000 | 1000 | 1 | 5.04E-05 | -0.7572667 | 35 | 3.5 | Mast3;Pik3r2 | Cytoskeleton;Signaling |
| DMR16:24212001 | 16 | 24212001 | 24213000 | 1000 | 1 | 2.02E-05 | -0.7056028 | 30 | 3 | | |
| DMR16:34235001 | 16 | 34235001 | 34236000 | 1000 | 1 | 6.33E-05 | 0.9555682 | 7 | 0.7 | | |
| DMR16:35298001 | 16 | 35298001 | 35299000 | 1000 | 1 | 2.57E-06 | -0.6308765 | 12 | 1.2 | | |
| DMR16:46632001 | 16 | 46632001 | 46633000 | 1000 | 1 | 2.53E-05 | 1.1123419 | 11 | 1.1 | | |
| DMR16:53246001 | 16 | 53246001 | 53247000 | 1000 | 1 | 4.20E-05 | 0.940437 | 9 | 0.9 | | |
| DMR16:57764001 | 16 | 57764001 | 57765000 | 1000 | 1 | 2.69E-05 | 0.8077448 | 4 | 0.4 | | |
| DMR16:62610001 | 16 | 62610001 | 62611000 | 1000 | 1 | 6.17E-05 | -0.673002 | 16 | 1.6 | Wrn | |
| DMR16:63888001 | 16 | 63888001 | 63889000 | 1000 | 1 | 4.70E-05 | 0.759881 | 15 | 1.5 | Nrg1 | Signaling |
| DMR16:65657001 | 16 | 65657001 | 65659000 | 2000 | 1 | 6.92E-05 | 0.8665165 | 18 | 0.9 | | |
| DMR16:70979001 | 16 | 70979001 | 70980000 | 1000 | 1 | 2.35E-05 | -1.2791294 | 12 | 1.2 | Kcnu1 | |
| DMR16:71825001 | 16 | 71825001 | 71826000 | 1000 | 1 | 6.13E-06 | -1.1208589 | 15 | 1.5 | Adam9 | Protease |
| DMR16:83404001 | 16 | 83404001 | 83405000 | 1000 | 1 | 3.08E-06 | 1.1653785 | 20 | 2 | Col4a2 | Cytoskeleton |
| DMR16:89195001 | 16 | 89195001 | 89196000 | 1000 | 1 | 4.44E-05 | 0.7180093 | 12 | 1.2 | | |
| DMR16:90106001 | 16 | 90106001 | 90107000 | 1000 | 1 | 6.53E-05 | -0.7579057 | 34 | 3.4 | | |
| DMR17:14001 | 17 | 14001 | 15000 | 1000 | 1 | 2.82E-05 | -0.6527067 | 35 | 3.5 | | |
| DMR17:5774001 | 17 | 5774001 | 5775000 | 1000 | 1 | 3.08E-06 | -0.8425829 | 24 | 2.4 | AABR07026936.1 | |
| DMR17:5936001 | 17 | 5936001 | 5937000 | 1000 | 1 | 5.54E-05 | -0.7961374 | 21 | 2.1 | Ntrk2 | Receptor |
| DMR17:7441001 | 17 | 7441001 | 7443000 | 2000 | 1 | 3.84E-05 | -0.4887868 | 48 | 2.4 | | |
| DMR17:10835001 | 17 | 10835001 | 10836000 | 1000 | 1 | 2.93E-05 | -0.8499713 | 20 | 2 | | |
| DMR17:12776001 | 17 | 12776001 | 12777000 | 1000 | 1 | 4.27E-05 | -0.8923175 | 15 | 1.5 | | |

| | | | | | | | | | | | |
|---|---|---|---|---|---|---|---|---|---|---|---|
| DMR17:18926001 | 17 | 18926001 | 18927000 | 1000 | 1 | 9.32E-05 | -0.5482089 | 29 | 2.9 | | |
| DMR17:28313001 | 17 | 28313001 | 28316000 | 3000 | 1 | 7.77E-05 | 0.6250175 | 22 | 0.733 | | |
| DMR17:28951001 | 17 | 28951001 | 28952000 | 1000 | 1 | 3.27E-05 | 0.9367378 | 13 | 1.3 | | |
| DMR17:33595001 | 17 | 33595001 | 33596000 | 1000 | 1 | 5.55E-05 | 0.5328882 | 16 | 1.6 | Gmds | Metabolism |
| DMR17:55386001 | 17 | 55386001 | 55387000 | 1000 | 1 | 1.20E-05 | 0.9817173 | 12 | 1.2 | | |
| DMR17:78483001 | 17 | 78483001 | 78484000 | 1000 | 1 | 1.49E-05 | -0.9031731 | 22 | 2.2 | Fam107b | |
| DMR17:81562001 | 17 | 81562001 | 81563000 | 1000 | 1 | 1.54E-05 | 1.2440664 | 17 | 1.7 | AABR07028691.1 | |
| DMR17:84421001 | 17 | 84421001 | 84422000 | 1000 | 1 | 4.67E-05 | -0.8814887 | 20 | 2 | Nebl | Cytoskeleton |
| DMR17:84957001 | 17 | 84957001 | 84959000 | 2000 | 1 | 9.77E-05 | -0.8780401 | 37 | 1.85 | Mllt10 | Transcription |
| DMR17:85150001 | 17 | 85150001 | 85151000 | 1000 | 1 | 6.75E-05 | -0.6255491 | 18 | 1.8 | Dnajc1 | Protein Binding |
| DMR17:87759001 | 17 | 87759001 | 87760000 | 1000 | 1 | 1.53E-05 | -0.8454164 | 44 | 4.4 | Arhgap21 | |
| DMR17:88078001 | 17 | 88078001 | 88079000 | 1000 | 1 | 5.85E-06 | -1.1806371 | 16 | 1.6 | Enkur;7SK | Metabolism |
| DMR18:4260001 | 18 | 4260001 | 4262000 | 2000 | 1 | 3.92E-06 | -1.2298257 | 39 | 1.95 | Osbpl1a | Binding Protein |
| DMR18:4282001 | 18 | 4282001 | 4283000 | 1000 | 1 | 2.04E-06 | -0.5574041 | 17 | 1.7 | Osbpl1a | Binding Protein |
| DMR18:17579001 | 18 | 17579001 | 17580000 | 1000 | 1 | 6.76E-05 | 0.6185393 | 10 | 1 | RGD1562608 | EST |
| DMR18:21283001 | 18 | 21283001 | 21284000 | 1000 | 1 | 5.82E-05 | 0.8108781 | 6 | 0.6 | | |
| DMR18:22436001 | 18 | 22436001 | 22437000 | 1000 | 1 | 7.97E-05 | 0.8063742 | 11 | 1.1 | | |
| DMR18:24455001 | 18 | 24455001 | 24456000 | 1000 | 1 | 2.48E-05 | -0.6616465 | 9 | 0.9 | Sap130 | Transcription |
| DMR18:26651001 | 18 | 26651001 | 26652000 | 1000 | 1 | 2.62E-05 | 0.8507968 | 14 | 1.4 | Epb41l4a | |
| DMR18:29054001 | 18 | 29054001 | 29055000 | 1000 | 1 | 7.27E-06 | 1.1365284 | 17 | 1.7 | | |
| DMR18:37949001 | 18 | 37949001 | 37950000 | 1000 | 1 | 8.37E-05 | 0.964605 | 8 | 0.8 | Jakmip2 | |
| DMR18:44794001 | 18 | 44794001 | 44795000 | 1000 | 1 | 8.48E-05 | -0.9277112 | 9 | 0.9 | | |
| DMR18:50868001 | 18 | 50868001 | 50869000 | 1000 | 1 | 8.20E-05 | 0.7765617 | 5 | 0.5 | | |
| DMR18:57790001 | 18 | 57790001 | 57792000 | 2000 | 1 | 9.23E-05 | -0.5315229 | 9 | 0.45 | Htr4 | Receptor |
| DMR18:60618001 | 18 | 60618001 | 60619000 | 1000 | 1 | 1.39E-05 | 0.8639702 | 12 | 1.2 | Nedd4l | Protease |
| DMR18:73349001 | 18 | 73349001 | 73350000 | 1000 | 1 | 2.60E-05 | -0.9647943 | 15 | 1.5 | Katnal2 | Cytoskeleton |
| DMR18:74336001 | 18 | 74336001 | 74338000 | 2000 | 1 | 1.93E-05 | -0.6084028 | 35 | 1.75 | Epg5 | |
| DMR18:77098001 | 18 | 77098001 | 77101000 | 3000 | 1 | 4.22E-05 | -1.1263825 | 61 | 2.033 | | |
| DMR18:78439001 | 18 | 78439001 | 78440000 | 1000 | 1 | 1.07E-05 | -1.193072 | 20 | 2 | | |
| DMR18:88057001 | 18 | 88057001 | 88058000 | 1000 | 1 | 1.92E-06 | 0.8075668 | 13 | 1.3 | | |
| DMR19:320001 | 19 | 320001 | 321000 | 1000 | 1 | 2.85E-05 | 0.646622 | 8 | 0.8 | AABR07042611.1 | |
| DMR19:1982001 | 19 | 1982001 | 1983000 | 1000 | 1 | 8.49E-06 | -1.1772273 | 9 | 0.9 | | |
| DMR19:10982001 | 19 | 10982001 | 10983000 | 1000 | 1 | 7.50E-05 | 0.8027405 | 8 | 0.8 | Nlrc5;AABR07042821.1 | |
| DMR19:11487001 | 19 | 11487001 | 11489000 | 2000 | 1 | 1.11E-05 | -0.8562619 | 61 | 3.05 | Amfr | Metabolism |
| DMR19:15788001 | 19 | 15788001 | 15789000 | 1000 | 1 | 7.94E-05 | 1.0287958 | 14 | 1.4 | | |
| DMR19:19197001 | 19 | 19197001 | 19198000 | 1000 | 1 | 3.68E-05 | -0.7344393 | 16 | 1.6 | | |
| DMR19:25485001 | 19 | 25485001 | 25486000 | 1000 | 1 | 4.42E-05 | 0.6496544 | 9 | 0.9 | | |
| DMR19:26927001 | 19 | 26927001 | 26930000 | 3000 | 1 | 8.50E-05 | 0.5084653 | 36 | 1.2 | | |
| DMR19:28982001 | 19 | 28982001 | 28983000 | 1000 | 1 | 7.33E-06 | 0.9922295 | 19 | 1.9 | AABR07043510.1 | |
| DMR19:35206001 | 19 | 35206001 | 35207000 | 1000 | 1 | 6.05E-05 | 0.8755385 | 12 | 1.2 | | |
| DMR19:44080001 | 19 | 44080001 | 44081000 | 1000 | 1 | 7.62E-05 | 1.0933906 | 4 | 0.4 | Cfdp1;Tmem170a | |
| DMR19:44706001 | 19 | 44706001 | 44708000 | 2000 | 1 | 7.33E-05 | -0.7390165 | 34 | 1.7 | | |
| DMR19:48852001 | 19 | 48852001 | 48853000 | 1000 | 1 | 7.46E-05 | -0.6103465 | 24 | 2.4 | | |
| DMR19:53134001 | 19 | 53134001 | 53135000 | 1000 | 1 | 3.86E-05 | -0.6610956 | 28 | 2.8 | | |
| DMR19:55184001 | 19 | 55184001 | 55185000 | 1000 | 1 | 2.01E-05 | -0.6411139 | 23 | 2.3 | Mlnr | |
| DMR19:56330001 | 19 | 56330001 | 56331000 | 1000 | 1 | 1.39E-05 | 1.1142256 | 14 | 1.4 | Gas8;7SK;5S_rRNA | Cell Cycle |
| DMR19:56766001 | 19 | 56766001 | 56767000 | 1000 | 1 | 3.86E-06 | -0.7909609 | 17 | 1.7 | Abcb10 | Transport |
| DMR19:59505001 | 19 | 59505001 | 59506000 | 1000 | 1 | 1.02E-05 | 0.9777604 | 14 | 1.4 | Irf2bp2 | |
| DMR19:59684001 | 19 | 59684001 | 59686000 | 2000 | 1 | 8.31E-05 | 0.8006625 | 20 | 1 | AABR07072667.1 | |
| DMR20:2079001 | 20 | 2079001 | 2080000 | 1000 | 1 | 8.95E-05 | 0.7882347 | 5 | 0.5 | RT1-M6-1;RT1-M6-2;Znrd1as1;Znrd | Immune;Transcription |
| DMR20:3953001 | 20 | 3953001 | 3954000 | 1000 | 1 | 4.10E-07 | -1.4289848 | 18 | 1.8 | RT1-DMb | |
| DMR20:5497001 | 20 | 5497001 | 5498000 | 1000 | 1 | 4.14E-05 | -0.8948988 | 18 | 1.8 | AA926063;AC128962.3;AC128962.2 | |
| DMR20:5618001 | 20 | 5618001 | 5619000 | 1000 | 1 | 3.87E-05 | 1.0868536 | 27 | 2.7 | Bak1;Ggnbp1 | Signaling |
| DMR20:6679001 | 20 | 6679001 | 6680000 | 1000 | 1 | 3.78E-05 | 1.1633981 | 11 | 1.1 | Ppil1 | Immune |
| DMR20:7379001 | 20 | 7379001 | 7380000 | 1000 | 1 | 3.74E-05 | 0.9458047 | 11 | 1.1 | LOC294154 | |
| DMR20:14930001 | 20 | 14930001 | 14931000 | 1000 | 1 | 5.66E-05 | 1.0066869 | 9 | 0.9 | | |
| DMR20:18052001 | 20 | 18052001 | 18053000 | 1000 | 1 | 3.18E-06 | -0.7617799 | 8 | 0.8 | LOC100364062;AABR07044711.1 | |
| DMR20:18875001 | 20 | 18875001 | 18877000 | 2000 | 1 | 9.40E-06 | -0.6430933 | 58 | 2.9 | Bicc1 | Transcription |
| DMR20:24494001 | 20 | 24494001 | 24495000 | 1000 | 1 | 4.90E-05 | 0.7435574 | 7 | 0.7 | | |
| DMR20:28682001 | 20 | 28682001 | 28683000 | 1000 | 1 | 1.59E-06 | -0.6029695 | 28 | 2.8 | Sh3rf3 | |
| DMR20:46147001 | 20 | 46147001 | 46149000 | 2000 | 1 | 7.65E-05 | -1.0497232 | 29 | 1.45 | Ak9 | |
| DMR20:50233001 | 20 | 50233001 | 50234000 | 1000 | 1 | 6.55E-05 | -0.8247211 | 38 | 3.8 | Prep | Protease |
| DMRX:14945001 | X | 14945001 | 14946000 | 1000 | 1 | 1.83E-06 | 0.8696046 | 5 | 0.5 | | |
| DMRX:143388001 | X | 143388001 | 143390000 | 2000 | 1 | 1.11E-05 | -0.5818821 | 48 | 2.4 | Atp11c | Transport |
