# Supplementary material for: Epigenome-wide association study for pesticide (Permethrin and DEET) induced DNA methylation epimutation biomarkers for specific transgenerational disease
Source: Environ Health. 2020 Nov 4;19:109. doi: 10.1186/s12940-020-00666-y (PMC7643320; doi:10.1186/s12940-020-00666-y)
Supplement: Supplementary file 1 — Additional file 1K Supplemental Table S1. DMR Site List Prostate p < 1e-04. DMR name, chromosome, start, stop, length, number signature windows, minimum p-value, max log-fold change, CpG number, CpG density, gene annotation, and gene category are presented. Supplemental Table S2. DMR Site List Kidney p < 1e-04. DMR name, chromosome, start, stop, length, number signature windows, minimum p-value, max log-fold change, CpG number, CpG density, gene annotation, and gene category are presented. Supplemental Table S3. DMR Site List Testis p < 1e-04. DMR name, chromosome, start, stop, length, number signature windows, minimum p-value, max log-fold change, CpG number, CpG density, gene annotation, and gene category are presented. Supplemental Table S4. DMR Site List Multiple p < 1e-04. DMR name, chromosome, start, stop, length, number signature windows, minimum p-value, max log-fold change, CpG number, CpG density, gene annotation, and gene category are presented. [file 12940_2020_666_MOESM1_ESM.zip › SuppTable-S3_siteTable.pesticides.testes.1e-04R2.pdf]

Supplemental Table S3
DMR Site Table Pesticides Testis Disease p<1e-04

DMR Name	Chr	Start	Stop	Length	# Sig Win	minP	maxLFC	CpG #	CpG Density	Gene Annotation	Gene Category
DMR1:14113001	1	14113001	14114000	1000	1	1.72E-05	-0.9952686	12	1.2	RGD1560303	
DMR1:23756001	1	23756001	23757000	1000	1	4.99E-05	-1.7238872	5	0.5		
DMR1:43967001	1	43967001	43968000	1000	1	2.53E-05	-1.2117023	19	1.9		
DMR1:48355001	1	48355001	48356000	1000	1	1.47E-05	0.9899698	23	2.3	Slc22a2	Transport
DMR1:65860001	1	65860001	65861000	1000	1	3.35E-05	-1.2728874	8	0.8	AABR07002068.1;Zscan18	Transcription
DMR1:69477001	1	69477001	69478000	1000	1	1.62E-06	-1.1762215	5	0.5		
DMR1:79308001	1	79308001	79309000	1000	1	2.88E-07	-1.6656471	4	0.4	AABR07002659.1	
DMR1:97096001	1	97096001	97097000	1000	1	4.18E-05	-1.7242562	5	0.5	AABR07003167.2	
DMR1:101039001	1	101039001	101041000	2000	1	7.25E-05	-0.8362783	76	3.8	Prr12;Prrg2	Metabolism
DMR1:101471001	1	101471001	101472000	1000	1	1.02E-05	-1.7859714	16	1.6	Dhdh;Tulp2;Nucb1	Metabolism;Transcription
DMR1:102045001	1	102045001	102046000	1000	1	5.55E-05	0.876501	30	3	Nomo1	Development
DMR1:107365001	1	107365001	107366000	1000	1	8.26E-06	-1.7806011	5	0.5	Gas2;Svip	Growth Factors & Cytokines
DMR1:123114001	1	123114001	123116000	2000	1	6.02E-05	-1.8062735	13	0.65		
DMR1:131634001	1	131634001	131635000	1000	1	1.19E-06	0.7222618	14	1.4		
DMR1:142115001	1	142115001	142116000	1000	1	2.38E-05	0.9158957	6	0.6	Prc1;Rccd1;Unc45a	Cytoskeleton;Signaling;Translation
DMR1:157841001	1	157841001	157843000	2000	1	5.96E-05	-1.0514347	33	1.65		
DMR1:158298001	1	158298001	158299000	1000	1	1.59E-06	-1.9760707	8	0.8		
DMR1:179620001	1	179620001	179621000	1000	1	2.09E-06	-2.7655772	3	0.3		
DMR1:182168001	1	182168001	182169000	1000	1	2.32E-05	-1.3606734	6	0.6		
DMR1:216147001	1	216147001	216148000	1000	1	3.75E-05	-1.7843859	9	0.9	Ascl2	Transcription
DMR1:263236001	1	263236001	263237000	1000	1	3.91E-05	-0.7519815	5	0.5	Cnnm1;Got1	Metabolism
DMR2:5725001	2	5725001	5726000	1000	1	1.49E-06	-1.3347477	7	0.7		
DMR2:12951001	2	12951001	12952000	1000	1	9.80E-05	-1.3936245	5	0.5		
DMR2:28843001	2	28843001	28844000	1000	1	6.96E-05	-1.1509647	6	0.6	AABR07007798.1;AABR07007799.1	
DMR2:62369001	2	62369001	62370000	1000	1	6.44E-05	-1.1441364	12	1.2	Golph3	Golgi
DMR2:73432001	2	73432001	73433000	1000	1	4.96E-05	-1.940806	11	1.1		
DMR2:88995001	2	88995001	88996000	1000	1	1.32E-05	-1.4436488	9	0.9		
DMR2:112711001	2	112711001	112713000	2000	1	1.44E-06	0.9489921	18	0.9		
DMR2:141605001	2	141605001	141606000	1000	1	3.64E-05	0.7162723	19	1.9		
DMR2:158086001	2	158086001	158087000	1000	1	5.58E-05	-1.293822	3	0.3	Veph1	Unknown
DMR2:163835001	2	163835001	163836000	1000	1	2.59E-06	-2.1938286	6	0.6		
DMR2:187590001	2	187590001	187591000	1000	1	4.79E-05	0.8616936	16	1.6	AABR07072748.1;Mir3597-1	
DMR2:203327001	2	203327001	203328000	1000	1	3.27E-05	-1.4773907	13	1.3	Ttf2	Transcription
DMR2:207868001	2	207868001	207869000	1000	1	9.02E-05	0.9581995	9	0.9	AABR07012774.2	
DMR2:217086001	2	217086001	217087000	1000	1	8.18E-05	0.6985522	13	1.3		
DMR2:235561001	2	235561001	235562000	1000	1	2.01E-05	0.738814	17	1.7		
DMR2:244166001	2	244166001	244167000	1000	1	3.08E-06	1.3170497	19	1.9	Tspan5	Cytoskeleton
DMR3:39836001	3	39836001	39837000	1000	1	8.34E-05	-1.1563895	12	1.2		
DMR3:50064001	3	50064001	50065000	1000	1	9.02E-05	1.1234908	15	1.5	Fign	Development
DMR3:93280001	3	93280001	93281000	1000	1	4.82E-06	-1.1097581	7	0.7		
DMR3:114490001	3	114490001	114491000	1000	1	5.82E-05	-1.4853232	6	0.6	Slc28a2	Metabolism
DMR3:126984001	3	126984001	126985000	1000	1	5.51E-05	0.8245181	8	0.8		
DMR3:140336001	3	140336001	140337000	1000	1	4.47E-05	-1.2820238	12	1.2	AABR07054096.1	
DMR3:165933001	3	165933001	165934000	1000	1	2.23E-05	-1.4629153	8	0.8		
DMR3:166543001	3	166543001	166544000	1000	1	5.46E-05	-1.3616196	24	2.4		
DMR3:170023001	3	170023001	170025000	2000	1	1.37E-05	1.0553493	32	1.6	Cbln4	Signaling
DMR4:20245001	4	20245001	20246000	1000	1	5.88E-05	-1.606837	4	0.4		
DMR4:26194001	4	26194001	26195000	1000	1	5.38E-07	1.3111164	13	1.3	Cdk14	Cell Cycle
DMR4:85246001	4	85246001	85247000	1000	1	2.82E-05	-1.5242158	17	1.7	Gars	Metabolism
DMR4:126009001	4	126009001	126010000	1000	1	6.67E-05	-1.1424807	16	1.6		
DMR4:142922001	4	142922001	142923000	1000	1	5.68E-05	-1.6913971	5	0.5	Grm7	Receptor
DMR4:146654001	4	146654001	146655000	1000	1	2.43E-05	1.0116666	11	1.1	Atg7	Apoptosis
DMR4:157177001	4	157177001	157178000	1000	1	4.72E-05	-1.2130601	14	1.4	Lpcat3	
DMR5:17466001	5	17466001	17467000	1000	1	6.89E-05	-1.2689892	9	0.9		
DMR5:19035001	5	19035001	19036000	1000	1	2.62E-05	-1.7223815	6	0.6		
DMR5:21747001	5	21747001	21749000	2000	1	6.25E-05	-1.5980406	33	1.65		
DMR5:26445001	5	26445001	26446000	1000	1	1.21E-05	-1.3166606	14	1.4		
DMR5:50919001	5	50919001	50920000	1000	1	6.99E-05	-1.9921083	9	0.9		
DMR5:57103001	5	57103001	57104000	1000	1	5.59E-05	0.9623447	13	1.3		
DMR5:60079001	5	60079001	60080000	1000	1	9.07E-05	-1.5580413	14	1.4	Pax5	Transcription
DMR5:90028001	5	90028001	90029000	1000	1	5.30E-08	0.7410233	15	1.5		
DMR5:98684001	5	98684001	98688000	4000	1	4.83E-05	-1.1419026	35	0.875	AABR07048957.1;AABR07048966.1	
DMR5:131033001	5	131033001	131034000	1000	1	7.30E-05	-1.4561025	4	0.4		
DMR5:144608001	5	144608001	144609000	1000	1	5.45E-06	-1.0413093	9	0.9	Clspn	
DMR5:156339001	5	156339001	156340000	1000	1	8.77E-05	0.7962245	12	1.2		
DMR5:158406001	5	158406001	158408000	2000	1	1.19E-06	-1.5705265	29	1.45		
DMR5:169796001	5	169796001	169797000	1000	1	9.27E-05	-1.0318796	10	1		
DMR5:171713001	5	171713001	171716000	3000	1	8.40E-05	0.7386103	69	2.3	Prdm16	Transcription
DMR6:807001	6	807001	808000	1000	1	7.75E-05	0.8330247	17	1.7	Crim1	Development

DMR6:3281001	6	3281001	3282000	1000	1	7.12E-05	0.6744271	22	2.2	Map4k3	Signaling
DMR6:7967001	6	7967001	7968000	1000	1	7.15E-05	-1.6218292	25	2.5	Abcg5;Abcg8	Transport
DMR6:33579001	6	33579001	33580000	1000	1	7.17E-05	-0.9720852	6	0.6	AC142360.1	
DMR6:41225001	6	41225001	41226000	1000	1	3.39E-06	-1.7598972	5	0.5		
DMR6:58489001	6	58489001	58490000	1000	1	6.64E-05	0.9374834	10	1	Etv1	Transcription
DMR6:75485001	6	75485001	75486000	1000	1	6.39E-05	0.7731818	14	1.4		
DMR6:100458001	6	100458001	100459000	1000	1	2.27E-06	-1.8484529	5	0.5	Fut8;U6	Metabolism
DMR6:118204001	6	118204001	118205000	1000	1	3.18E-05	-1.5911478	6	0.6		
DMR6:134606001	6	134606001	134607000	1000	1	1.67E-06	1.1397934	12	1.2		
DMR6:135855001	6	135855001	135856000	1000	1	7.40E-05	-1.1180991	13	1.3	Lbhd2	
DMR7:41482001	7	41482001	41483000	1000	1	6.17E-05	-1.0687674	11	1.1	Dusp6	Signaling
DMR7:70497001	7	70497001	70498000	1000	1	2.50E-05	-1.6653493	16	1.6	Pip4k2c;AC114111.2	Signaling
DMR7:72376001	7	72376001	72377000	1000	1	8.14E-05	-1.3153318	2	0.2		
DMR7:100597001	7	100597001	100598000	1000	1	6.21E-06	-1.6791478	10	1		
DMR7:119820001	7	119820001	119823000	3000	1	1.76E-05	-1.819668	38	1.267	Cyth4	Signaling
DMR7:124400001	7	124400001	124401000	1000	1	7.58E-05	-1.2446728	14	1.4	Bik	
DMR7:134323001	7	134323001	134324000	1000	1	4.95E-05	-1.4864974	8	0.8		
DMR8:5901001	8	5901001	5903000	2000	1	7.84E-05	-1.6151136	14	0.7	Mmp7	Proteolysis
DMR8:9197001	8	9197001	9198000	1000	1	5.05E-05	-1.3931179	9	0.9		
DMR8:12625001	8	12625001	12626000	1000	1	4.75E-05	0.9475555	13	1.3		
DMR8:14752001	8	14752001	14753000	1000	1	3.41E-06	-1.477554	5	0.5	Fat3	Transcription
DMR8:24210001	8	24210001	24211000	1000	1	6.20E-05	-1.2088645	10	1		
DMR8:56316001	8	56316001	56317000	1000	1	2.74E-06	-2.1368296	8	0.8		
DMR8:63333001	8	63333001	63334000	1000	1	4.44E-05	0.8864629	9	0.9	Cd276	
DMR8:111138001	8	111138001	111139000	1000	1	2.91E-05	-0.7780596	18	1.8		
DMR9:26045001	9	26045001	26046000	1000	1	2.91E-05	-1.2769738	9	0.9		
DMR9:29586001	9	29586001	29588000	2000	1	5.34E-05	0.9801618	21	1.05	AABR07067082.1	
DMR9:33510001	9	33510001	33511000	1000	1	4.07E-05	-1.450734	8	0.8		
DMR9:41223001	9	41223001	41224000	1000	1	6.44E-05	-1.5668276	15	1.5		
DMR9:77894001	9	77894001	77895000	1000	1	4.88E-05	1.1640372	5	0.5	Vwc2l	
DMR9:78192001	9	78192001	78193000	1000	1	6.52E-05	-1.1572656	11	1.1		
DMR10:47927001	10	47927001	47928000	1000	1	3.75E-06	0.8924216	17	1.7	AC134746.1;Grap	Signaling
DMR10:81088001	10	81088001	81089000	1000	1	7.22E-05	-1.2600029	12	1.2	Car10	Metabolism
DMR10:81884001	10	81884001	81885000	1000	1	2.68E-05	1.0890016	19	1.9	AABR07030334.1	
DMR10:91273001	10	91273001	91274000	1000	1	3.08E-05	-1.2705149	13	1.3	Fmnl1;LOC100361655	Cytoskeleton
DMR10:95462001	10	95462001	95464000	2000	1	3.64E-05	0.661458	20	1	AABR07030605.1	
DMR10:96650001	10	96650001	96651000	1000	1	3.21E-05	-1.0643969	17	1.7	Apoh	Binding Protein
DMR10:103618001	10	103618001	103619000	1000	1	7.36E-08	-1.5892467	11	1.1	Cd300e	Receptor
DMR10:111433001	10	111433001	111435000	2000	1	9.15E-06	-1.5553773	15	0.75		
DMR11:15390001	11	15390001	15391000	1000	1	2.54E-07	-2.0159527	11	1.1		
DMR11:24315001	11	24315001	24316000	1000	1	8.05E-05	-1.1192893	6	0.6	Gabpa	Transcription
DMR11:63918001	11	63918001	63919000	1000	1	2.67E-05	-0.8993539	12	1.2		
DMR11:71024001	11	71024001	71025000	1000	1	3.02E-05	-1.8195822	11	1.1	Iqcg;Lrch3	Unknown
DMR11:77037001	11	77037001	77038000	1000	1	8.43E-05	0.8655219	6	0.6		
DMR11:82384001	11	82384001	82385000	1000	1	7.90E-05	-1.5243937	14	1.4	Tra2b	Translation
DMR12:18914001	12	18914001	18915000	1000	1	3.67E-07	-1.5851856	8	0.8		
DMR13:16038001	13	16038001	16039000	1000	1	5.89E-05	-2.1091071	2	0.2		
DMR13:26693001	13	26693001	26694000	1000	1	2.12E-05	0.9240508	18	1.8	Bcl2	Signaling
DMR13:31523001	13	31523001	31524000	1000	1	9.74E-05	-2.0364771	6	0.6		
DMR13:44686001	13	44686001	44689000	3000	1	6.94E-05	0.7619939	59	1.967	Zranb3	Transcription
DMR13:47650001	13	47650001	47651000	1000	1	7.56E-05	-1.8523159	8	0.8	Il20	Signaling
DMR13:74032001	13	74032001	74033000	1000	1	7.62E-05	0.8991014	12	1.2	Soat1	Metabolism
DMR13:81980001	13	81980001	81981000	1000	1	8.80E-05	0.781813	6	0.6	Mettl11b	Epigenetic
DMR13:89929001	13	89929001	89930000	1000	1	1.09E-06	1.1765595	20	2	Itln1	
DMR13:107738001	13	107738001	107739000	1000	1	2.34E-05	-1.3641513	14	1.4	Kcnk2	Transport
DMR14:83427001	14	83427001	83428000	1000	1	3.36E-05	-1.4066331	9	0.9	Eif4enif1	
DMR14:101736001	14	101736001	101737000	1000	1	6.06E-05	-1.5127747	23	2.3		
DMR14:111278001	14	111278001	111279000	1000	1	5.88E-06	-1.7848443	3	0.3		
DMR15:7766001	15	7766001	7767000	1000	1	2.82E-05	-0.8715759	22	2.2		
DMR15:10140001	15	10140001	10141000	1000	1	2.98E-05	-1.4506241	17	1.7	Rarb	Signaling
DMR15:13561001	15	13561001	13563000	2000	1	2.98E-05	-1.3348499	28	1.4		
DMR15:39843001	15	39843001	39845000	2000	1	9.83E-05	0.957004	18	0.9	Cab39l	Signaling
DMR15:59630001	15	59630001	59631000	1000	1	5.60E-05	0.7770884	18	1.8		
DMR15:61866001	15	61866001	61867000	1000	1	8.57E-05	0.9197194	16	1.6	Elf1;LOC100360244;Sugt1;AC123280.1	Transcription
DMR15:84017001	15	84017001	84019000	2000	1	9.96E-06	0.880863	36	1.8	AABR07019020.1	
DMR15:89841001	15	89841001	89843000	2000	1	7.39E-05	-1.2605052	34	1.7	Mycbp2	Metabolism
DMR15:97201001	15	97201001	97202000	1000	1	2.57E-05	-1.5918867	4	0.4		
DMR16:14383001	16	14383001	14384000	1000	1	9.22E-05	-2.2168328	26	2.6	Ghitm	Growth Factors & Cytokines
DMR16:36485001	16	36485001	36486000	1000	1	4.06E-05	-1.5426792	10	1		
DMR16:36774001	16	36774001	36775000	1000	1	8.20E-05	-1.7007271	5	0.5		
DMR16:47702001	16	47702001	47703000	1000	1	7.27E-05	0.7174871	5	0.5		
DMR16:64088001	16	64088001	64089000	1000	1	6.92E-06	-1.6341283	5	0.5		
DMR16:72814001	16	72814001	72815000	1000	1	3.79E-05	-0.9422599	6	0.6	Zmat4	Transcription
DMR16:77803001	16	77803001	77804000	1000	1	8.99E-05	-1.559764	10	1		

DMR16:79528001	16	79528001	79529000	1000	1	4.95E-05	-1.7880174	10	1	AABR07026503.1	
DMR17:8074001	17	8074001	8075000	1000	1	1.03E-05	0.6092562	6	0.6		
DMR17:12294001	17	12294001	12296000	2000	1	3.39E-05	0.8160355	40	2		
DMR17:14973001	17	14973001	14974000	1000	1	2.38E-06	-1.9127017	6	0.6		
DMR17:17815001	17	17815001	17816000	1000	1	2.46E-05	-1.1870672	9	0.9	Rnf144b	Proteolysis
DMR17:22659001	17	22659001	22660000	1000	1	6.04E-05	-1.9559728	11	1.1	Adtrp;LOC100362172	Development
DMR17:57808001	17	57808001	57811000	3000	1	5.59E-07	1.2561284	47	1.567	LOC103694120	
DMR17:79754001	17	79754001	79755000	1000	1	2.19E-05	-1.520348	14	1.4	Mindy3;AABR07028665.1	
DMR17:80634001	17	80634001	80635000	1000	1	8.98E-06	-1.2742629	12	1.2	Cubn	Receptor
DMR18:24504001	18	24504001	24505000	1000	1	1.09E-05	0.9409845	15	1.5	Sap130	Transcription
DMR18:28973001	18	28973001	28974000	1000	1	2.85E-06	1.0129672	22	2.2	Nrg2	Signaling
DMR18:81575001	18	81575001	81576000	1000	1	3.20E-06	-2.2468439	22	2.2	Dipk1c	
DMR19:7613001	19	7613001	7614000	1000	1	4.60E-05	1.2117896	16	1.6		
DMR19:47740001	19	47740001	47741000	1000	1	3.80E-05	1.0665226	13	1.3		
DMR20:18234001	20	18234001	18235000	1000	1	1.58E-05	0.9420626	21	2.1		
DMR20:42172001	20	42172001	42173000	1000	1	2.62E-05	-1.5648177	3	0.3		
DMR20:52227001	20	52227001	52228000	1000	1	2.70E-05	-1.443977	5	0.5		
DMRX:18033001	X	18033001	18034000	1000	1	5.48E-05	-1.6818459	12	1.2		
DMRX:30003001	X	30003001	30004000	1000	1	5.63E-05	1.0912353	12	1.2		
DMRX:154585001	X	154585001	154586000	1000	1	2.01E-05	-0.7337535	5	0.5	AABR07042385.1	
