# Supplementary material for: Epigenome-wide association study for pesticide (Permethrin and DEET) induced DNA methylation epimutation biomarkers for specific transgenerational disease
Source: Environ Health. 2020 Nov 4;19:109. doi: 10.1186/s12940-020-00666-y (PMC7643320; doi:10.1186/s12940-020-00666-y)
Supplement: Supplementary file 1 — Additional file 1K Supplemental Table S1. DMR Site List Prostate p < 1e-04. DMR name, chromosome, start, stop, length, number signature windows, minimum p-value, max log-fold change, CpG number, CpG density, gene annotation, and gene category are presented. Supplemental Table S2. DMR Site List Kidney p < 1e-04. DMR name, chromosome, start, stop, length, number signature windows, minimum p-value, max log-fold change, CpG number, CpG density, gene annotation, and gene category are presented. Supplemental Table S3. DMR Site List Testis p < 1e-04. DMR name, chromosome, start, stop, length, number signature windows, minimum p-value, max log-fold change, CpG number, CpG density, gene annotation, and gene category are presented. Supplemental Table S4. DMR Site List Multiple p < 1e-04. DMR name, chromosome, start, stop, length, number signature windows, minimum p-value, max log-fold change, CpG number, CpG density, gene annotation, and gene category are presented. [file 12940_2020_666_MOESM1_ESM.zip › SuppTable-S4_siteTable.pesticides.multiple.1e-04R2.pdf]

Supplemental Table S4
DMR Site Table Pesticides Multiple Disease p<1e-04

DMR Name	Chr	Start	Stop	Length	# Sig Win	minP	maxLFC	CpG #	CpG Density	Gene Annotation	Gene Category
DMR1:62001	1	62001	63000	1000	1	8.53E-05	0.65024187	2	0.2		
DMR1:2151001	1	2151001	2152000	1000	1	2.53E-05	0.48787343	4	0.4		
DMR1:7305001	1	7305001	7306000	1000	1	9.41E-05	-0.48820691	0	0	Zc2hc1b;AABR07000261.1	
DMR1:7312001	1	7312001	7316000	4000	1	5.51E-05	0.41193608	50	1.25	Zc2hc1b;AABR07000261.1;Ltv1	Development
DMR1:7822001	1	7822001	7823000	1000	1	4.77E-06	-0.73671674	24	2.4	Aig1	
DMR1:8256001	1	8256001	8257000	1000	1	6.60E-05	-0.73989904	11	1.1		
DMR1:8801001	1	8801001	8802000	1000	1	7.76E-07	0.58295237	9	0.9		
DMR1:11162001	1	11162001	11163000	1000	1	6.29E-05	0.85509124	6	0.6		
DMR1:11478001	1	11478001	11479000	1000	1	2.62E-06	-1.00772578	7	0.7		
DMR1:12770001	1	12770001	12771000	1000	1	3.51E-05	-0.54501409	16	1.6	AC128394.3	
DMR1:12900001	1	12900001	12901000	1000	1	1.82E-05	0.59248032	7	0.7	AC128394.1	
DMR1:12905001	1	12905001	12907000	2000	1	1.73E-05	-0.46382537	77	3.85	AC128394.1;Txlnb	Immune
DMR1:13210001	1	13210001	13211000	1000	1	2.75E-06	-0.35489395	6	0.6	Reps1	Signaling
DMR1:14546001	1	14546001	14547000	1000	1	1.17E-05	-0.85006815	12	1.2	AABR07000484.1	
DMR1:14952001	1	14952001	14953000	1000	1	1.54E-05	-0.62037191	25	2.5	AABR07000493.1	
DMR1:15223001	1	15223001	15225000	2000	1	2.23E-05	0.72525121	32	1.6	Il20ra	
DMR1:16216001	1	16216001	16217000	1000	1	5.94E-05	1.05806677	9	0.9		
DMR1:17465001	1	17465001	17466000	1000	1	7.81E-05	-0.56091186	16	1.6	Ptprk	Signaling
DMR1:17798001	1	17798001	17799000	1000	1	7.31E-05	-0.63669307	16	1.6	Ptprk	Signaling
DMR1:18085001	1	18085001	18086000	1000	1	8.75E-05	0.52996482	6	0.6	AABR07000574.1	
DMR1:18366001	1	18366001	18368000	2000	1	4.81E-05	0.81245296	26	1.3		
DMR1:19388001	1	19388001	19390000	2000	1	2.16E-05	0.91610387	26	1.3		
DMR1:22445001	1	22445001	22446000	1000	1	2.60E-06	1.0396484	6	0.6	Taar7h;AC128759.2	Receptor
DMR1:23416001	1	23416001	23417000	1000	1	6.26E-05	0.76206337	15	1.5	Eya4	Transcription
DMR1:28039001	1	28039001	28040000	1000	1	1.86E-06	-0.56954733	2	0.2	Nkain2	Transport
DMR1:31804001	1	31804001	31805000	1000	1	4.61E-05	0.59849809	5	0.5		
DMR1:32027001	1	32027001	32028000	1000	1	5.26E-05	-0.58414877	13	1.3		
DMR1:34633001	1	34633001	34636000	3000	1	2.72E-07	0.57911486	32	1.067		
DMR1:37783001	1	37783001	37784000	1000	1	5.75E-05	-0.75367261	15	1.5	Mtrr	Epigenetic
DMR1:39450001	1	39450001	39451000	1000	1	8.13E-05	-0.61282619	16	1.6		
DMR1:39536001	1	39536001	39540000	4000	1	1.06E-05	-0.49847662	158	3.95		
DMR1:39683001	1	39683001	39685000	2000	1	1.26E-05	-0.4805272	64	3.2		
DMR1:39717001	1	39717001	39718000	1000	1	3.99E-05	-0.49234983	35	3.5	AABR07001348.1	
DMR1:42393001	1	42393001	42394000	1000	1	8.47E-05	0.41665235	4	0.4		
DMR1:44593001	1	44593001	44595000	2000	1	6.79E-05	0.69632378	20	1	Nox3	Metabolism
DMR1:46550001	1	46550001	46552000	2000	1	4.14E-05	-0.39859871	23	1.15	Zdhhc14	Unknown
DMR1:48208001	1	48208001	48209000	1000	1	2.01E-05	-0.52877976	17	1.7	Igf2r;AC129234.1	Growth Factors & Cytokines
DMR1:51621001	1	51621001	51622000	1000	1	5.77E-06	-0.92375841	21	2.1	Pabpc6	
DMR1:52714001	1	52714001	52715000	1000	1	3.10E-05	0.32851141	12	1.2		
DMR1:52730001	1	52730001	52731000	1000	1	3.90E-05	0.60742177	2	0.2		
DMR1:53543001	1	53543001	53544000	1000	1	6.56E-05	0.84630775	15	1.5	Tcp10b	
DMR1:54176001	1	54176001	54177000	1000	1	4.01E-05	0.59041714	7	0.7		
DMR1:56960001	1	56960001	56961000	1000	1	3.71E-06	-0.68818772	11	1.1	LOC100125364;Phf10;Tcte3	Transcription
DMR1:57311001	1	57311001	57313000	2000	1	7.73E-05	0.63628244	43	2.15	Dll1	Receptor
DMR1:60365001	1	60365001	60367000	2000	1	1.96E-05	-0.48898063	65	3.25		
DMR1:60762001	1	60762001	60763000	1000	1	4.85E-05	-1.00863023	0	0		
DMR1:61303001	1	61303001	61304000	1000	1	1.07E-05	-0.84736799	11	1.1		
DMR1:63481001	1	63481001	63483000	2000	1	2.14E-05	-0.45754406	23	1.15		
DMR1:63563001	1	63563001	63566000	3000	1	3.93E-06	0.44356351	25	0.833	Vom2r26	
DMR1:64106001	1	64106001	64107000	1000	1	7.13E-06	0.90561526	4	0.4	Tmc4;Tsen34l1;Mboat7	Metabolism;Receptor
DMR1:71873001	1	71873001	71875000	2000	1	8.56E-07	0.60160985	19	0.95	Nlrp9	
DMR1:72194001	1	72194001	72195000	1000	1	2.12E-06	-1.18907898	1	0.1	Vom1r36	
DMR1:72940001	1	72940001	72941000	1000	1	4.29E-05	-0.78746451	8	0.8	Eps8l1	Receptor
DMR1:75455001	1	75455001	75456000	1000	1	1.37E-05	0.57175943	12	1.2	Pla2g4c;7SK;AC127640.1	Metabolism
DMR1:76581001	1	76581001	76583000	2000	1	5.20E-05	-0.87892438	13	0.65	Sult2a1	Metabolism
DMR1:77316001	1	77316001	77317000	1000	1	1.44E-05	-0.91554034	2	0.2		
DMR1:79740001	1	79740001	79741000	1000	1	4.94E-05	0.622967	5	0.5		
DMR1:79960001	1	79960001	79961000	1000	1	7.23E-07	-0.75824622	14	1.4	Sympk;Rsph6a	Extracellular Matrix;Cytoskeleton
DMR1:80990001	1	80990001	80991000	1000	1	7.69E-07	-0.5595954	63	6.3	AC118165.1;AABR07002683.1	
DMR1:81001001	1	81001001	81002000	1000	1	6.76E-05	0.79823555	7	0.7	AC118165.1	
DMR1:81982001	1	81982001	81983000	1000	1	6.99E-07	-0.88456899	25	2.5	Pou2f2	Transcription
DMR1:82499001	1	82499001	82501000	2000	1	3.43E-05	-0.7918952	34	1.7	Tgfb1;Ccdc97	Signaling
DMR1:82565001	1	82565001	82566000	1000	1	9.25E-05	-0.39132062	12	1.2	Axl	Receptor
DMR1:84451001	1	84451001	84452000	1000	1	4.50E-05	-0.55327431	11	1.1	Akt2	Signaling
DMR1:85251001	1	85251001	85253000	2000	1	5.49E-06	-0.50850535	21	1.05	Ifnl1	
DMR1:86788001	1	86788001	86790000	2000	1	4.54E-06	0.79217972	10	0.5		

DMR1:86811001	1	86811001	86813000	2000	1	2.28E-06	0.46660831	12	0.6	AABR07002845.1	
DMR1:87010001	1	87010001	87011000	1000	1	4.03E-05	-0.76413516	18	1.8	Hnrnpl;Ech1;Lgals4	Transcription;Metabolism;Signaling
DMR1:87305001	1	87305001	87307000	2000	1	9.20E-05	0.65621445	29	1.45	Sipa1l3	Signaling
DMR1:89128001	1	89128001	89129000	1000	1	1.16E-08	-0.74789139	26	2.6	Haus5;AC141526.1	
DMR1:89220001	1	89220001	89221000	1000	1	7.46E-05	-0.58952728	21	2.1	Dmkn	
DMR1:89901001	1	89901001	89902000	1000	1	1.91E-05	0.45435205	14	1.4	Scgb2b24;Apbh	Unknown
DMR1:90512001	1	90512001	90513000	1000	1	5.87E-05	-0.37874861	43	4.3	Kctd15	Signaling
DMR1:91193001	1	91193001	91195000	2000	2	6.06E-06	0.65954423	29	1.45		
DMR1:91400001	1	91400001	91401000	1000	1	9.88E-05	0.32869606	15	1.5		
DMR1:92366001	1	92366001	92367000	1000	1	4.11E-05	1.02955789	7	0.7		
DMR1:92608001	1	92608001	92609000	1000	1	6.05E-05	-0.56143464	16	1.6		
DMR1:92716001	1	92716001	92718000	2000	1	6.99E-05	-0.45280827	38	1.9	Tshz3;7SK	Transcription
DMR1:92811001	1	92811001	92813000	2000	1	7.98E-05	-0.63922165	41	2.05		
DMR1:94508001	1	94508001	94509000	1000	1	3.44E-06	0.54154352	7	0.7		
DMR1:94645001	1	94645001	94646000	1000	1	9.43E-05	0.39146657	5	0.5		
DMR1:95295001	1	95295001	95296000	1000	1	4.20E-05	-0.4777131	15	1.5		
DMR1:95319001	1	95319001	95321000	2000	1	4.69E-06	0.78955557	15	0.75		
DMR1:97745001	1	97745001	97746000	1000	1	1.51E-05	0.88939387	7	0.7		
DMR1:99057001	1	99057001	99058000	1000	1	4.85E-06	0.28279661	10	1	Vom2r37	
DMR1:100482001	1	100482001	100488000	6000	3	4.15E-07	0.45705805	53	0.883	Aspdh;Josd2;Emc10	Metabolism;Protease
DMR1:100825001	1	100825001	100827000	2000	1	7.19E-05	-0.56163582	33	1.65	Nup62;Il4i1;Tbc1d17	Metabolism;Signaling
DMR1:102596001	1	102596001	102597000	1000	1	7.56E-05	-0.400154	2	0.2	Sergef	Signaling
DMR1:105318001	1	105318001	105319000	1000	1	7.96E-05	0.91335066	6	0.6	Slc6a5	Transport
DMR1:105566001	1	105566001	105568000	2000	1	7.49E-05	0.81653225	30	1.5	Nell1	Development
DMR1:112382001	1	112382001	112383000	1000	1	3.25E-05	-0.60581467	5	0.5	Luzp2;Gabrg3	Receptor
DMR1:112854001	1	112854001	112856000	2000	1	7.55E-06	0.60287149	18	0.9	Luzp2;Gabra5	Receptor
DMR1:112952001	1	112952001	112953000	1000	1	1.25E-07	0.8289579	7	0.7	Luzp2;Gabra5;AABR07003500.2	Receptor
DMR1:114404001	1	114404001	114405000	1000	1	5.81E-05	-0.84567053	12	1.2	Nipa1	Development
DMR1:119999001	1	119999001	1.20E+08	1000	1	4.61E-06	0.7433993	3	0.3		
DMR1:121354001	1	121354001	121355000	1000	1	8.40E-05	0.9686349	5	0.5		
DMR1:124109001	1	124109001	124110000	1000	1	1.90E-05	-0.84525056	11	1.1		
DMR1:125325001	1	125325001	125326000	1000	1	7.90E-05	0.54856059	8	0.8		
DMR1:127993001	1	127993001	127994000	1000	1	7.44E-06	-0.73984932	23	2.3	Adamts17	Proteolysis
DMR1:128712001	1	128712001	128713000	1000	1	9.42E-08	-0.88436408	13	1.3	Synm	Cytoskeleton
DMR1:128827001	1	128827001	128828000	1000	1	4.08E-05	0.4756302	17	1.7		
DMR1:129164001	1	129164001	129165000	1000	1	3.77E-05	-0.52791114	36	3.6	Igf1r	Growth Factors & Cytokines
DMR1:131257001	1	131257001	131258000	1000	1	1.53E-05	0.41202542	4	0.4		
DMR1:131755001	1	131755001	131756000	1000	1	1.89E-05	0.46228408	9	0.9		
DMR1:135192001	1	135192001	135193000	1000	1	1.24E-05	-0.58929983	19	1.9		
DMR1:143522001	1	143522001	143523000	1000	1	7.53E-05	0.42449965	4	0.4	Homer2	Signaling
DMR1:143530001	1	143530001	143531000	1000	1	3.08E-05	-0.51273029	27	2.7	Homer2	Signaling
DMR1:143568001	1	143568001	143569000	1000	1	7.76E-05	-0.77171951	10	1		
DMR1:144102001	1	144102001	144103000	1000	1	5.19E-05	-0.41786376	9	0.9	Sh3gl3	Signaling
DMR1:144215001	1	144215001	144216000	1000	1	1.57E-05	0.57772201	12	1.2		
DMR1:144402001	1	144402001	144403000	1000	1	4.99E-06	-0.81287373	22	2.2	Adamtsl3	Extracellular Matrix
DMR1:144478001	1	144478001	144480000	2000	1	6.81E-05	0.62221545	12	0.6	Adamtsl3	Extracellular Matrix
DMR1:147502001	1	147502001	147503000	1000	1	2.97E-05	0.69252297	3	0.3		
DMR1:147833001	1	147833001	147834000	1000	1	6.83E-05	0.45753268	5	0.5	Cyp2c6v1	Metabolism
DMR1:148425001	1	148425001	148430000	5000	1	2.89E-08	-0.78295329	246	4.92	Vbp1	Protein Binding
DMR1:148455001	1	148455001	148457000	2000	1	6.90E-05	-0.76505759	53	2.65		
DMR1:150449001	1	150449001	150450000	1000	1	2.84E-06	0.52998614	3	0.3		
DMR1:153060001	1	153060001	153064000	4000	1	8.96E-05	0.43933308	39	0.975	Tmem135	Unknown
DMR1:153085001	1	153085001	153086000	1000	1	2.10E-05	0.74835504	9	0.9	Tmem135	Unknown
DMR1:154086001	1	154086001	154087000	1000	1	6.34E-05	0.52998542	3	0.3	Ccdc81	
DMR1:159151001	1	159151001	159152000	1000	1	1.62E-05	-1.10136379	2	0.2		
DMR1:164310001	1	164310001	164311000	1000	1	3.65E-06	0.86280015	6	0.6	Serpinh1	Development
DMR1:164784001	1	164784001	164785000	1000	1	8.99E-06	0.64885093	9	0.9		
DMR1:166661001	1	166661001	166662000	1000	1	4.92E-05	0.56373912	7	0.7	Art2b	
DMR1:166693001	1	166693001	166694000	1000	1	8.43E-05	0.82126257	8	0.8		
DMR1:169871001	1	169871001	169873000	2000	1	9.80E-05	0.39802259	21	1.05	Olr181	Receptor
DMR1:171715001	1	171715001	171716000	1000	1	8.82E-05	-0.6290168	14	1.4	Syt9	Transport
DMR1:174250001	1	174250001	174251000	1000	1	2.75E-05	1.06963924	11	1.1	Dennd2b	
DMR1:175021001	1	175021001	175023000	2000	1	7.36E-05	-0.43959339	75	3.75	Sbf2	Epigenetic
DMR1:175143001	1	175143001	175145000	2000	1	5.34E-05	-0.34049864	30	1.5	Sbf2	Epigenetic
DMR1:177838001	1	177838001	177839000	1000	1	1.98E-05	-0.52947959	21	2.1		
DMR1:179684001	1	179684001	179685000	1000	1	3.18E-08	0.63266805	4	0.4		
DMR1:183697001	1	183697001	183698000	1000	1	3.18E-05	-0.74984637	17	1.7	Copb1	Metabolism
DMR1:184420001	1	184420001	184421000	1000	1	1.03E-05	-0.61557218	6	0.6		
DMR1:184428001	1	184428001	184429000	1000	1	7.24E-05	0.81805187	10	1		
DMR1:185504001	1	185504001	185506000	2000	1	7.60E-05	1.016513	25	1.25	Plekha7	Signaling

DMR1:185760001	1	185760001	185761000	1000	1	3.21E-05	-0.52789056	37	3.7	Sox6	Development
DMR1:190357001	1	190357001	190358000	1000	1	4.50E-05	1.14715652	6	0.6	Abca16	
DMR1:192206001	1	192206001	192207000	1000	1	5.64E-05	0.67059202	9	0.9		
DMR1:192526001	1	192526001	192527000	1000	1	4.19E-05	-0.54370033	22	2.2	Prkcb	Signaling
DMR1:194860001	1	194860001	194864000	4000	1	1.88E-05	0.3450661	61	1.525	SNORD116	
DMR1:195287001	1	195287001	195289000	2000	1	4.77E-05	-0.608878	13	0.65		
DMR1:195693001	1	195693001	195695000	2000	1	6.98E-06	0.60485925	13	0.65		
DMR1:197299001	1	197299001	197300000	1000	1	3.43E-05	-0.76118464	26	2.6		
DMR1:199134001	1	199134001	199136000	2000	1	5.93E-05	-0.52079137	40	2		
DMR1:200691001	1	200691001	200694000	3000	1	4.89E-05	-0.50174823	83	2.767	Fgfr2	Receptor
DMR1:206089001	1	206089001	206090000	1000	1	3.21E-06	-0.58518842	13	1.3	Adam12	Protease
DMR1:209076001	1	209076001	209077000	1000	1	3.54E-05	0.49666558	5	0.5	AABR07005961.1	
DMR1:210195001	1	210195001	210197000	2000	1	4.55E-06	0.43196109	14	0.7		
DMR1:214380001	1	214380001	214381000	1000	1	2.39E-05	1.02862741	7	0.7	Taldo1;Gatd1	Metabolism
DMR1:214785001	1	214785001	214786000	1000	1	1.88E-05	-0.98605486	17	1.7	Muc5b	Extracellular Matrix
DMR1:218047001	1	218047001	218048000	1000	1	8.08E-05	-0.41918663	5	0.5		
DMR1:218326001	1	218326001	218327000	1000	1	1.84E-05	-0.4931685	22	2.2		
DMR1:218824001	1	218824001	218825000	1000	1	4.56E-06	-0.81191988	23	2.3	Lrp5	Receptor
DMR1:219703001	1	219703001	219704000	1000	1	2.69E-05	0.63000591	6	0.6	Syt12	Transport
DMR1:220685001	1	220685001	220686000	1000	1	2.44E-05	0.59257771	7	0.7	Gal3st3	Golgi
DMR1:221469001	1	221469001	221470000	1000	1	4.44E-06	0.38212461	8	0.8	Cdca5;Naaladl1	Protease
DMR1:222063001	1	222063001	222064000	1000	1	1.83E-05	-0.40547685	11	1.1		
DMR1:223635001	1	223635001	223636000	1000	1	7.60E-06	0.80156007	16	1.6		
DMR1:224036001	1	224036001	224038000	2000	2	1.23E-06	0.60570831	18	0.9		
DMR1:224486001	1	224486001	224489000	3000	1	4.73E-05	0.71676726	23	0.767	Ust5r	Metabolism
DMR1:226424001	1	226424001	226426000	2000	1	8.02E-05	0.47811731	16	0.8	Syt7	
DMR1:226440001	1	226440001	226441000	1000	1	4.86E-06	-0.80730686	24	2.4	Syt7	
DMR1:226792001	1	226792001	226794000	2000	1	4.82E-05	-0.37753591	11	0.55	Cd5;AABR07072028.1	Receptor
DMR1:228009001	1	228009001	228011000	2000	1	3.83E-05	0.44817526	9	0.45	Oosp2	
DMR1:228869001	1	228869001	228871000	2000	1	2.19E-05	0.48794533	10	0.5		
DMR1:229987001	1	229987001	229988000	1000	1	3.00E-05	0.75805822	7	0.7		
DMR1:231132001	1	231132001	231133000	1000	1	5.10E-05	0.48623576	51	5.1		
DMR1:238439001	1	238439001	238441000	2000	1	2.07E-05	-0.48141842	13	0.65	Tmc1	
DMR1:240592001	1	240592001	240594000	2000	1	7.54E-05	-0.74378547	26	1.3	Trpm3;Aldh1a7	Receptor
DMR1:242676001	1	242676001	242678000	2000	1	2.14E-07	0.61834639	6	0.3	Pgm5	Metabolism
DMR1:245785001	1	245785001	245786000	1000	1	4.58E-07	-0.65649497	10	1		
DMR1:246625001	1	246625001	246626000	1000	1	4.80E-05	-0.61752965	15	1.5	Glis3	Transcription
DMR1:247219001	1	247219001	247220000	1000	1	1.79E-06	0.76028125	8	0.8	Rcl1	Transcription
DMR1:247767001	1	247767001	247768000	1000	1	6.43E-05	-0.46431335	18	1.8	Ric1	
DMR1:248382001	1	248382001	248384000	2000	1	9.04E-05	0.39432267	7	0.35	Gldc	Metabolism
DMR1:248776001	1	248776001	248777000	1000	1	5.41E-05	0.60075367	5	0.5		
DMR1:251544001	1	251544001	251547000	3000	1	5.70E-05	0.36772927	38	1.267		
DMR1:251620001	1	251620001	251621000	1000	1	2.84E-06	1.06448386	11	1.1		
DMR1:251633001	1	251633001	251635000	2000	1	2.16E-05	-0.28715099	3	0.15	AC094647.2;7SK	
DMR1:252044001	1	252044001	252045000	1000	1	4.67E-06	-0.73968299	11	1.1	Rnls	Metabolism
DMR1:252598001	1	252598001	252600000	2000	1	4.50E-05	0.57083615	11	0.55	Fas;U6	Receptor
DMR1:253673001	1	253673001	253674000	1000	1	7.76E-06	0.52394557	2	0.2		
DMR1:254387001	1	254387001	254388000	1000	1	3.83E-05	0.5520142	15	1.5		
DMR1:255985001	1	255985001	255986000	1000	1	9.22E-05	0.65825511	11	1.1	Ide	Protease
DMR1:258740001	1	258740001	258741000	1000	1	3.44E-06	0.56138525	1	0.1	Cyp2c12	
DMR1:260845001	1	260845001	260846000	1000	1	7.62E-05	-0.57900052	24	2.4	Slit1	Receptor
DMR1:261837001	1	261837001	261838000	1000	1	7.37E-05	0.69640602	12	1.2	R3hcc1l;Loxl4	Receptor
DMR1:263928001	1	263928001	263929000	1000	1	7.36E-05	-0.52514387	40	4	Bloc1s2;Pkd2l1;U6	Transport
DMR1:264359001	1	264359001	264361000	2000	1	1.52E-05	-0.55844967	23	1.15		
DMR1:265557001	1	265557001	265558000	1000	1	3.27E-05	-0.26868467	3	0.3	LOC100911951	Signaling
DMR1:266985001	1	266985001	266986000	1000	1	2.19E-05	-0.53485911	16	1.6	Neurl1	Protease
DMR1:267537001	1	267537001	267538000	1000	1	5.11E-05	-0.57635903	20	2	Cfap43	
DMR1:271374001	1	271374001	271375000	1000	1	1.25E-05	0.95043106	2	0.2	Cfap58l1	
DMR1:271665001	1	271665001	271666000	1000	1	3.08E-05	-0.70950518	24	2.4		
DMR1:274110001	1	274110001	274112000	2000	1	9.08E-06	-0.47556648	34	1.7	Smndc1	Transcription
DMR1:274268001	1	274268001	274269000	1000	1	2.04E-06	0.54246344	5	0.5	Dusp5	Signaling
DMR1:274399001	1	274399001	274402000	3000	1	1.90E-07	0.71101836	32	1.067	Rbm20	
DMR1:275891001	1	275891001	275894000	3000	1	2.23E-06	-0.63594061	136	4.533	Gpam	Metabolism
DMR1:277615001	1	277615001	277616000	1000	1	3.92E-05	-0.73864195	22	2.2	Ccdc186	
DMR1:277998001	1	277998001	277999000	1000	1	9.57E-06	-1.10185469	19	1.9	AABR07007032.1	
DMR1:278430001	1	278430001	278431000	1000	1	7.90E-05	-0.55522391	22	2.2		
DMR1:278457001	1	278457001	278459000	2000	2	3.05E-06	0.46366894	14	0.7		
DMR1:278500001	1	278500001	278501000	1000	1	6.02E-06	0.85565443	6	0.6		
DMR2:3634001	2	3634001	3635000	1000	1	1.38E-06	-0.87483004	4	0.4	Mctp1;Gm24519	Unknown
DMR2:3871001	2	3871001	3873000	2000	1	3.39E-05	0.62525417	9	0.45	Mctp1	Unknown
DMR2:4037001	2	4037001	4038000	1000	1	4.26E-05	-0.80734675	3	0.3	Mctp1	Unknown

DMR2:7257001	2	7257001	7258000	1000	1	9.68E-07	0.52502221	2	0.2		
DMR2:7298001	2	7298001	7300000	2000	1	2.65E-05	0.40259604	8	0.4		
DMR2:9879001	2	9879001	9880000	1000	1	1.93E-05	0.45138078	3	0.3		
DMR2:19457001	2	19457001	19459000	2000	1	1.91E-05	0.58338095	11	0.55	AABR07007566.1	
DMR2:20016001	2	20016001	20018000	2000	1	8.79E-05	-0.64019846	34	1.7	Atg10	
DMR2:22682001	2	22682001	22683000	1000	1	9.07E-05	-0.8109758	23	2.3	Cmya5	
DMR2:23532001	2	23532001	23533000	1000	1	5.69E-05	0.39486818	12	1.2	Arsb	Metabolism
DMR2:25086001	2	25086001	25087000	1000	1	5.14E-05	-0.47295502	25	2.5	Aggf1	
DMR2:25394001	2	25394001	25395000	1000	1	2.65E-05	0.83862234	7	0.7		
DMR2:26418001	2	26418001	26420000	2000	1	3.92E-05	0.90274826	29	1.45	Iqgap2	Signaling
DMR2:26771001	2	26771001	26772000	1000	1	1.61E-05	-0.69052957	10	1		
DMR2:27034001	2	27034001	27036000	2000	1	2.70E-05	0.44979773	19	0.95	Poc5	
DMR2:27390001	2	27390001	27391000	1000	1	9.15E-05	-0.57914723	3	0.3	Cert1	
DMR2:27550001	2	27550001	27552000	2000	1	1.16E-05	0.59162572	8	0.4		
DMR2:27867001	2	27867001	27868000	1000	1	5.02E-05	0.60277907	19	1.9		
DMR2:28011001	2	28011001	28012000	1000	1	3.90E-05	-0.3940102	13	1.3	Hexb	Golgi
DMR2:28221001	2	28221001	28222000	1000	1	9.28E-05	0.64377436	6	0.6		
DMR2:28717001	2	28717001	28718000	1000	1	3.05E-05	0.85687019	14	1.4		
DMR2:33263001	2	33263001	33264000	1000	1	2.60E-05	0.9023606	15	1.5		
DMR2:36671001	2	36671001	36673000	2000	1	5.39E-06	0.60165692	7	0.35		
DMR2:38406001	2	38406001	38407000	1000	1	6.39E-05	0.51830484	3	0.3		
DMR2:44261001	2	44261001	44262000	1000	1	3.44E-08	0.96416748	7	0.7	AABR07008118.1	
DMR2:44553001	2	44553001	44556000	3000	1	5.59E-05	0.57912767	24	0.8	Slc38a9;AABR07008123.1	
DMR2:44778001	2	44778001	44779000	1000	1	7.88E-05	0.42623111	4	0.4	Mtrex;Dhx29	Transcription
DMR2:46023001	2	46023001	46024000	1000	1	9.21E-07	-0.53134002	26	2.6	Arl15	Signaling
DMR2:46267001	2	46267001	46268000	1000	1	3.43E-06	1.00111914	8	0.8		
DMR2:46483001	2	46483001	46484000	1000	1	2.56E-05	-0.57546462	11	1.1	Ndufs4	Metabolism
DMR2:46513001	2	46513001	46515000	2000	1	9.82E-05	0.57024741	17	0.85		
DMR2:48979001	2	48979001	48980000	1000	1	9.65E-05	-0.49975391	14	1.4	LOC499530	
DMR2:49588001	2	49588001	49589000	1000	1	7.29E-06	1.20676233	7	0.7		
DMR2:49970001	2	49970001	49971000	1000	1	8.26E-06	-0.54117744	44	4.4		
DMR2:51014001	2	51014001	51015000	1000	1	1.73E-05	-0.52216668	12	1.2		
DMR2:53406001	2	53406001	53407000	1000	1	7.62E-05	-0.46969475	32	3.2	Ghr	Receptor
DMR2:54130001	2	54130001	54131000	1000	1	3.77E-05	-0.78670087	10	1		
DMR2:57715001	2	57715001	57716000	1000	1	2.31E-05	-0.74484904	12	1.2		
DMR2:60845001	2	60845001	60847000	2000	1	5.82E-05	0.85383298	16	0.8		
DMR2:62583001	2	62583001	62585000	2000	1	1.31E-06	0.98147705	32	1.6	Pdzd2	
DMR2:62719001	2	62719001	62721000	2000	1	6.06E-05	-0.76749009	11	0.55		
DMR2:63144001	2	63144001	63147000	3000	1	2.41E-05	0.43320732	17	0.567	Cdh6	Cytoskeleton
DMR2:70179001	2	70179001	70180000	1000	1	4.31E-05	-1.24220233	4	0.4		
DMR2:74342001	2	74342001	74345000	3000	1	2.63E-05	0.75810018	25	0.833		
DMR2:80515001	2	80515001	80516000	1000	1	4.39E-05	0.84757348	11	1.1	Trio	Signaling
DMR2:80590001	2	80590001	80591000	1000	1	5.85E-05	-0.5126533	25	2.5	Trio	Signaling
DMR2:80820001	2	80820001	80821000	1000	1	2.84E-05	-0.55386471	4	0.4		
DMR2:80908001	2	80908001	80909000	1000	1	8.63E-06	0.55863773	10	1		
DMR2:85529001	2	85529001	85530000	1000	1	6.47E-05	-0.66812136	10	1	Sema5a	Signaling
DMR2:87633001	2	87633001	87634000	1000	1	3.12E-07	0.63912053	10	1		
DMR2:89361001	2	89361001	89362000	1000	1	1.16E-05	0.52346632	11	1.1		
DMR2:91489001	2	91489001	91490000	1000	1	8.58E-05	-0.55526706	5	0.5	Pcsk1	Protease
DMR2:93615001	2	93615001	93616000	1000	1	8.47E-05	0.84734539	8	0.8	Chmp4c	Epigenetic
DMR2:97829001	2	97829001	97830000	1000	1	4.64E-05	-0.435505	48	4.8		
DMR2:98363001	2	98363001	98364000	1000	1	1.64E-05	0.74496975	4	0.4		
DMR2:101107001	2	101107001	101108000	1000	1	2.36E-05	-0.59346765	17	1.7		
DMR2:101537001	2	101537001	101540000	3000	1	3.76E-05	0.45252087	13	0.433		
DMR2:103427001	2	103427001	103428000	1000	1	6.56E-05	0.68498076	4	0.4		
DMR2:104707001	2	104707001	104709000	2000	1	1.99E-05	0.38620946	6	0.3		
DMR2:104820001	2	104820001	104821000	1000	1	9.76E-05	0.6892035	10	1	Hps3	Development
DMR2:105228001	2	105228001	105229000	1000	1	7.74E-05	-0.440606	46	4.6	Agtr1b	Receptor
DMR2:106554001	2	106554001	106555000	1000	1	3.03E-05	-0.77267506	7	0.7		
DMR2:106718001	2	106718001	106719000	1000	1	2.02E-05	-0.63758904	6	0.6		
DMR2:106836001	2	106836001	106837000	1000	1	9.12E-05	0.45286147	5	0.5		
DMR2:108945001	2	108945001	108947000	2000	1	3.10E-07	-0.40510114	73	3.65		
DMR2:112329001	2	112329001	112330000	1000	1	8.87E-05	0.65149072	7	0.7	Spata16	
DMR2:113149001	2	113149001	113150000	1000	1	6.37E-05	-0.51549635	39	3.9	Fndc3b	Cytoskeleton
DMR2:115655001	2	115655001	115656000	1000	1	9.17E-05	-1.04752961	7	0.7		
DMR2:116025001	2	116025001	116026000	1000	1	2.61E-05	-0.60313611	27	2.7	Phc3	Transcription
DMR2:122214001	2	122214001	122215000	1000	1	5.97E-06	-0.34947322	37	3.7		
DMR2:124112001	2	124112001	124114000	2000	1	4.93E-05	-0.48418643	33	1.65	Fgf2	Growth Factors & Cytokines
DMR2:128816001	2	128816001	128820000	4000	1	2.17E-05	-0.40395533	258	6.45	5_8S_rRNA	
DMR2:129507001	2	129507001	129510000	3000	1	8.59E-05	0.71960675	24	0.8		
DMR2:131987001	2	131987001	131989000	2000	1	1.25E-05	-0.5444999	11	0.55		
DMR2:133487001	2	133487001	133488000	1000	1	9.02E-06	0.59951997	8	0.8		

DMR2:133951001	2	133951001	133952000	1000	1	3.81E-05	-0.38592292	42	4.2		
DMR2:138902001	2	138902001	138903000	1000	1	6.22E-05	-0.65818724	7	0.7		
DMR2:139703001	2	139703001	139704000	1000	1	5.15E-05	-1.22570783	3	0.3		
DMR2:139944001	2	139944001	139946000	2000	1	7.22E-05	-0.46009678	27	1.35	AABR07010550.2	
DMR2:140782001	2	140782001	140783000	1000	1	1.88E-06	-0.62096866	7	0.7		
DMR2:141304001	2	141304001	141308000	4000	1	9.79E-05	0.34407117	46	1.15		
DMR2:145297001	2	145297001	145298000	1000	1	9.57E-05	-0.90149888	12	1.2		
DMR2:148079001	2	148079001	148081000	2000	1	9.00E-05	-0.70306987	33	1.65		
DMR2:151898001	2	151898001	151899000	1000	1	4.59E-05	-0.70692511	4	0.4		
DMR2:154691001	2	154691001	154692000	1000	1	2.32E-05	-0.48167986	31	3.1	Vom2r46	
DMR2:155135001	2	155135001	155137000	2000	1	9.33E-05	0.25757149	21	1.05		
DMR2:155299001	2	155299001	155300000	1000	1	8.91E-05	0.58944107	11	1.1	AABR07010935.1	
DMR2:156435001	2	156435001	156437000	2000	1	1.19E-05	-0.27018738	14	0.7		
DMR2:156677001	2	156677001	156678000	1000	1	1.18E-05	0.3386393	6	0.6	AABR07011013.1	
DMR2:156992001	2	156992001	156993000	1000	1	8.60E-05	-0.44027409	48	4.8		
DMR2:159998001	2	159998001	159999000	1000	1	1.40E-13	-0.54034317	21	2.1		
DMR2:160717001	2	160717001	160719000	2000	1	4.95E-05	-0.31187652	24	1.2		
DMR2:162051001	2	162051001	162052000	1000	1	8.27E-05	-0.55260183	17	1.7		
DMR2:162414001	2	162414001	162416000	2000	1	8.88E-05	-0.30549979	24	1.2		
DMR2:164146001	2	164146001	164147000	1000	1	5.82E-06	0.91960376	10	1		
DMR2:164592001	2	164592001	164593000	1000	1	2.05E-05	-0.48488137	29	2.9	Mlf1;Gfm1	Signaling;Transcription
DMR2:164759001	2	164759001	164760000	1000	1	3.09E-06	-0.52911768	29	2.9	Mfsd1	Transport
DMR2:177957001	2	177957001	177958000	1000	1	1.50E-07	-0.54110277	11	1.1	Rapgef2	
DMR2:180115001	2	180115001	180116000	1000	1	7.29E-05	-0.93670309	9	0.9	Pdgfc	Growth Factors & Cytokines
DMR2:185401001	2	185401001	185402000	1000	1	8.34E-05	-0.66229154	9	0.9	Sh3d19	
DMR2:185671001	2	185671001	185672000	1000	1	8.17E-05	1.11275103	6	0.6	Lrba;U1	Signaling
DMR2:185807001	2	185807001	185808000	1000	1	9.79E-05	-0.63501667	9	0.9	Lrba;AABR07012115.1	Signaling
DMR2:187055001	2	187055001	187056000	1000	1	1.93E-06	1.24371037	6	0.6	Arhgef11	Signaling
DMR2:188127001	2	188127001	188128000	1000	1	8.10E-05	-0.58254841	19	1.9	Syt11	Development
DMR2:188142001	2	188142001	188144000	2000	1	6.00E-08	-0.6572847	33	1.65	Syt11;Gon4l	Development;Transcription
DMR2:189035001	2	189035001	189038000	3000	1	2.83E-07	-0.49211949	17	0.567		
DMR2:189880001	2	189880001	189882000	2000	1	7.28E-05	0.52476005	59	2.95	Ilf2;Snapin;Chtop	Transcription;Epigenetic
DMR2:191949001	2	191949001	191950000	1000	1	9.54E-05	0.43364792	2	0.2		
DMR2:192557001	2	192557001	192558000	1000	1	3.10E-05	-0.67659403	6	0.6	AABR07012302.1	
DMR2:194004001	2	194004001	194005000	1000	1	8.69E-05	0.82524765	4	0.4	RGD1564513	Unknown
DMR2:195918001	2	195918001	195919000	1000	1	3.18E-05	-0.50017123	32	3.2	Tuft1;Cgn	Unknown;Cytoskeleton
DMR2:196321001	2	196321001	196322000	1000	1	2.92E-05	0.35903071	10	1	Tnfaip8l2	Unknown
DMR2:197402001	2	197402001	197403000	1000	1	1.64E-05	0.93888956	10	1		
DMR2:199866001	2	199866001	199867000	1000	1	3.23E-06	0.51570031	1	0.1	Pde4dip	
DMR2:201325001	2	201325001	201326000	1000	1	6.97E-05	0.86487571	5	0.5	Tbx15	Transcription
DMR2:203518001	2	203518001	203520000	2000	1	3.20E-05	0.79362261	17	0.85		
DMR2:204505001	2	204505001	204506000	1000	1	1.93E-05	0.78582779	6	0.6	Casq2	Signaling
DMR2:206032001	2	206032001	206034000	2000	1	1.74E-05	0.53991504	25	1.25		
DMR2:210158001	2	210158001	210160000	2000	1	3.92E-05	1.23323902	29	1.45		
DMR2:210493001	2	210493001	210495000	2000	2	2.97E-05	0.64627818	12	0.6		
DMR2:210898001	2	210898001	210899000	1000	1	2.19E-05	0.76608174	14	1.4	Gnat2;Gnai3	Signaling
DMR2:211514001	2	211514001	211515000	1000	1	5.71E-05	-0.36958938	17	1.7	Gpsm2	Signaling
DMR2:212820001	2	212820001	212821000	1000	1	2.80E-05	0.4226705	5	0.5	Ntng1	Development
DMR2:217089001	2	217089001	217090000	1000	1	4.58E-05	0.56913733	10	1		
DMR2:222150001	2	222150001	222151000	1000	1	5.54E-05	0.82834706	9	0.9	Dpyd	Metabolism
DMR2:224963001	2	224963001	224964000	1000	1	4.81E-05	0.60174406	19	1.9		
DMR2:225218001	2	225218001	225219000	1000	1	3.41E-05	0.6696295	17	1.7		
DMR2:225288001	2	225288001	225289000	1000	1	1.89E-05	0.89756969	19	1.9		
DMR2:225793001	2	225793001	225795000	2000	1	4.59E-06	0.74953277	19	0.95	Abca4;AABR07013165.1	Transport
DMR2:226017001	2	226017001	226018000	1000	1	6.53E-05	-0.97158354	9	0.9		
DMR2:227584001	2	227584001	227585000	1000	1	5.77E-05	1.24900185	9	0.9	Mettl14;AABR07013207.1	Epigenetic
DMR2:228062001	2	228062001	228064000	2000	1	9.96E-06	0.62087522	19	0.95		
DMR2:228538001	2	228538001	228539000	1000	1	5.66E-05	-0.45880104	9	0.9	Tram1l1	
DMR2:230423001	2	230423001	230424000	1000	1	2.24E-05	-0.684153	34	3.4		
DMR2:231274001	2	231274001	231275000	1000	1	9.19E-05	0.76436503	9	0.9	Ank2	Cytoskeleton
DMR2:231971001	2	231971001	231974000	3000	1	7.77E-07	0.58538571	31	1.033	Neurog2	Transcription
DMR2:233638001	2	233638001	233639000	1000	1	1.35E-05	0.45494177	13	1.3		
DMR2:237180001	2	237180001	237181000	1000	1	4.78E-07	0.53717525	5	0.5	Dkk2	
DMR2:237964001	2	237964001	237966000	2000	1	1.68E-06	-0.5527897	32	1.6	Tbck;AABR07013453.1	Unknown
DMR2:238489001	2	238489001	238491000	2000	1	7.64E-05	-0.58493223	33	1.65		
DMR2:239073001	2	239073001	239074000	1000	1	1.04E-05	0.55176945	5	0.5	AABR07013477.2	
DMR2:240315001	2	240315001	240316000	1000	1	2.70E-05	-0.68005758	9	0.9		
DMR2:240437001	2	240437001	240438000	1000	1	3.81E-05	0.74226244	14	1.4	Cenpe	Cytoskeleton
DMR2:242623001	2	242623001	242625000	2000	1	5.01E-06	0.60546833	7	0.35	Emcn	Development
DMR2:243139001	2	243139001	243140000	1000	1	4.39E-05	0.65957258	17	1.7	Dnajb14	Protein Binding
DMR2:243239001	2	243239001	243241000	2000	1	5.42E-05	0.51390329	6	0.3		
DMR2:243529001	2	243529001	243530000	1000	1	6.33E-05	0.50064746	10	1		

DMR2:244425001	2	244425001	244426000	1000	1	8.14E-06	-0.51876781	18	1.8		
DMR2:247618001	2	247618001	247619000	1000	1	2.69E-05	-0.39932256	10	1		
DMR2:248571001	2	248571001	248573000	2000	1	1.10E-05	0.78124669	12	0.6	AABR07013654.1	
DMR2:252189001	2	252189001	252190000	1000	1	7.29E-05	0.39092923	15	1.5		
DMR2:252624001	2	252624001	252626000	2000	1	3.95E-06	-0.58384095	39	1.95	Prkacb	Signaling
DMR2:252872001	2	252872001	252873000	1000	1	5.67E-06	-0.54312339	13	1.3	AABR07013718.1	
DMR2:253139001	2	253139001	253140000	1000	1	6.96E-06	-0.6551657	9	0.9		
DMR2:253309001	2	253309001	253310000	1000	1	6.35E-05	0.93470549	6	0.6		
DMR2:254485001	2	254485001	254486000	1000	1	7.63E-07	0.67173345	5	0.5		
DMR2:258126001	2	258126001	258127000	1000	1	7.46E-08	-0.67594596	24	2.4		
DMR2:258729001	2	258729001	258730000	1000	1	4.03E-05	-0.43871953	24	2.4		
DMR2:259075001	2	259075001	259077000	2000	1	9.56E-05	0.9279737	24	1.2		
DMR2:259232001	2	259232001	259233000	1000	1	2.85E-05	0.71574095	12	1.2		
DMR2:260027001	2	260027001	260028000	1000	1	1.60E-05	-1.0505951	9	0.9	AABR07013859.1	
DMR2:262999001	2	262999001	2.63E+08	1000	1	6.29E-05	-0.77627056	18	1.8	Negr1	Growth Factors & Cytokines
DMR3:1140001	3	1140001	1141000	1000	1	4.02E-05	-0.62361408	6	0.6		
DMR3:1696001	3	1696001	1698000	2000	1	1.10E-05	-0.52174813	73	3.65		
DMR3:2219001	3	2219001	2220000	1000	1	8.89E-05	-0.92576808	21	2.1	Pnpla7	Metabolism
DMR3:4017001	3	4017001	4018000	1000	1	8.21E-05	-0.44003066	15	1.5	AC111292.4;AC111292.7;U6	
DMR3:5441001	3	5441001	5444000	3000	1	2.28E-06	-0.80783408	96	3.2	Surf6;Med22	Development;Transcription
DMR3:5934001	3	5934001	5935000	1000	1	8.15E-06	0.74051204	8	0.8	Vav2	Signaling
DMR3:6551001	3	6551001	6552000	1000	1	1.04E-05	-0.55962804	29	2.9	Col5a1	Extracellular Matrix
DMR3:6797001	3	6797001	6798000	1000	1	2.91E-06	-0.51775798	46	4.6	Olfm1	Receptor
DMR3:7301001	3	7301001	7302000	1000	1	6.01E-05	0.54210022	15	1.5	Ak8	
DMR3:7597001	3	7597001	7598000	1000	1	6.52E-05	-0.36952026	18	1.8	Cfap77	
DMR3:9285001	3	9285001	9286000	1000	1	7.02E-05	-0.56185069	7	0.7	Lamc3	Extracellular Matrix
DMR3:13154001	3	13154001	13156000	2000	1	3.07E-06	0.9703699	21	1.05		
DMR3:15322001	3	15322001	15323000	1000	1	7.15E-06	-0.38003176	13	1.3		
DMR3:20704001	3	20704001	20705000	1000	1	4.70E-06	-0.65448156	8	0.8	AABR07051746.1;U6	
DMR3:21400001	3	21400001	21401000	1000	1	2.61E-05	0.90500298	8	0.8		
DMR3:21822001	3	21822001	21823000	1000	1	2.49E-06	0.91059899	3	0.3	Rabgap1;Strbp	Signaling;Translation
DMR3:22378001	3	22378001	22379000	1000	1	6.18E-05	-0.58784842	14	1.4	Dennd1a	Signaling
DMR3:23107001	3	23107001	23108000	1000	1	7.59E-06	0.44176505	2	0.2		
DMR3:25000001	3	25000001	25001000	1000	1	7.41E-05	0.81036635	5	0.5		
DMR3:28536001	3	28536001	28537000	1000	1	5.76E-05	0.64187587	8	0.8	Kynu	Metabolism
DMR3:28605001	3	28605001	28606000	1000	1	5.52E-05	0.45314147	11	1.1		
DMR3:30068001	3	30068001	30069000	1000	1	3.84E-06	0.46748329	9	0.9		
DMR3:30151001	3	30151001	30152000	1000	1	9.31E-06	0.47576107	2	0.2		
DMR3:30657001	3	30657001	30659000	2000	1	9.45E-05	0.52625823	15	0.75		
DMR3:31316001	3	31316001	31318000	2000	1	5.89E-05	-0.47659992	12	0.6		
DMR3:33282001	3	33282001	33283000	1000	1	4.04E-05	-0.66253198	11	1.1		
DMR3:33516001	3	33516001	33518000	2000	1	1.18E-05	0.78337271	9	0.45		
DMR3:34221001	3	34221001	34222000	1000	1	1.10E-05	-0.54607659	5	0.5		
DMR3:38884001	3	38884001	38885000	1000	1	1.34E-05	-0.65512775	3	0.3		
DMR3:39169001	3	39169001	39171000	2000	1	8.66E-05	-0.82109331	11	0.55		
DMR3:40723001	3	40723001	40725000	2000	1	6.04E-05	-0.97000369	8	0.4		
DMR3:41776001	3	41776001	41778000	2000	1	8.53E-05	-0.37484548	12	0.6	5S_rRNA;AABR07052199.1	
DMR3:44508001	3	44508001	44509000	1000	1	9.92E-06	-0.52978116	24	2.4	Acvr1	Receptor
DMR3:44765001	3	44765001	44766000	1000	1	2.70E-05	0.73593897	3	0.3	7SK	
DMR3:46166001	3	46166001	46167000	1000	1	7.13E-05	-0.47221352	21	2.1		
DMR3:51260001	3	51260001	51262000	2000	1	1.93E-05	0.60134026	7	0.35	Cobll1	Development
DMR3:54394001	3	54394001	54396000	2000	1	9.70E-06	0.42117548	16	0.8	Stk39	
DMR3:54680001	3	54680001	54681000	1000	1	6.97E-06	-0.81049976	18	1.8		
DMR3:56191001	3	56191001	56192000	1000	1	6.90E-05	0.47264906	4	0.4	Ubr3	Metabolism
DMR3:56296001	3	56296001	56297000	1000	1	9.72E-06	0.79039183	9	0.9		
DMR3:56675001	3	56675001	56677000	2000	1	5.59E-05	0.82231757	22	1.1		
DMR3:58320001	3	58320001	58321000	1000	1	2.82E-09	0.74612407	6	0.6		
DMR3:58699001	3	58699001	58700000	1000	1	9.70E-05	0.81249743	17	1.7	Rapgef4	Development
DMR3:59774001	3	59774001	59775000	1000	1	3.06E-05	-0.4661462	22	2.2		
DMR3:60071001	3	60071001	60072000	1000	1	1.68E-05	0.7140122	5	0.5	Gpr155	Receptor
DMR3:63634001	3	63634001	63635000	1000	1	6.31E-05	0.85559031	9	0.9		
DMR3:65016001	3	65016001	65017000	1000	1	2.73E-05	0.87216961	14	1.4		
DMR3:71586001	3	71586001	71588000	2000	1	6.67E-05	0.60407559	21	1.05		
DMR3:72439001	3	72439001	72440000	1000	1	9.56E-06	0.92388738	2	0.2	P2rx3;Ssrp1	Receptor;Transcription
DMR3:75422001	3	75422001	75426000	4000	2	3.29E-06	0.63962988	39	0.975	Olr560;AC118490.1	
DMR3:75499001	3	75499001	75501000	2000	1	2.49E-07	0.68536319	15	0.75		
DMR3:76165001	3	76165001	76166000	1000	1	2.85E-05	-0.68684049	18	1.8	AC131848.1	
DMR3:82380001	3	82380001	82381000	1000	1	6.86E-05	-0.24276568	20	2	AABR07052895.2	
DMR3:84315001	3	84315001	84316000	1000	1	3.46E-05	0.44428756	3	0.3		
DMR3:85581001	3	85581001	85583000	2000	1	9.09E-05	0.63544284	17	0.85	Lrrc4c	Extracellular Matrix
DMR3:86232001	3	86232001	86233000	1000	1	3.76E-05	-0.95201838	4	0.4	Lrrc4c	Extracellular Matrix
DMR3:88979001	3	88979001	88981000	2000	1	4.74E-06	0.66171385	12	0.6		

DMR3:89772001	3	89772001	89773000	1000	1	9.62E-06	0.70033453	8	0.8		
DMR3:91723001	3	91723001	91724000	1000	1	1.92E-06	-0.46913889	21	2.1	Ldlrad3	Receptor
DMR3:96892001	3	96892001	96893000	1000	1	7.14E-05	-0.33143561	22	2.2		
DMR3:98854001	3	98854001	98855000	1000	1	7.96E-05	0.58734546	5	0.5		
DMR3:101387001	3	101387001	101388000	1000	1	8.84E-05	0.49922751	3	0.3		
DMR3:102536001	3	102536001	102537000	1000	1	8.45E-05	-0.7670351	6	0.6	Olr752	
DMR3:108717001	3	108717001	108718000	1000	1	1.24E-05	0.54465465	7	0.7	AABR07053471.1	
DMR3:108734001	3	108734001	108735000	1000	1	2.25E-11	0.66082143	8	0.8		
DMR3:112925001	3	112925001	112926000	1000	1	9.29E-05	0.52036154	11	1.1	Tmem62;AABR07072916.1	Transcription
DMR3:113530001	3	113530001	113531000	1000	1	7.68E-05	-0.87750541	12	1.2	Frmd5	Signaling
DMR3:114991001	3	114991001	114992000	1000	1	7.21E-06	-0.49403992	23	2.3		
DMR3:117962001	3	117962001	117964000	2000	1	5.27E-08	0.70276715	7	0.35	Shc4	Signaling
DMR3:119256001	3	119256001	119257000	1000	1	6.66E-06	0.77380157	13	1.3	Usp50;Trpm7	Protease;Development
DMR3:119570001	3	119570001	119572000	2000	1	6.31E-06	-0.64493355	37	1.85	Blvra	Metabolism
DMR3:120313001	3	120313001	120315000	2000	1	9.19E-05	-0.46665377	11	0.55	Mall;AABR07053687.1	
DMR3:123231001	3	123231001	123232000	1000	1	1.13E-05	0.48156972	10	1	Itpa;Slc4a11;U6	Signaling;Transport
DMR3:125345001	3	125345001	125346000	1000	1	1.69E-09	-0.67309816	39	3.9	Shld1	
DMR3:127483001	3	127483001	127485000	2000	1	2.96E-05	-0.86087414	20	1	Hao1	Metabolism
DMR3:132069001	3	132069001	132070000	1000	1	8.62E-05	-0.7664919	2	0.2		
DMR3:132686001	3	132686001	132687000	1000	1	8.25E-07	0.39975301	7	0.7	Sptlc3	Metabolism
DMR3:135957001	3	135957001	135958000	1000	1	5.61E-05	0.506604	5	0.5		
DMR3:137885001	3	137885001	137886000	1000	1	3.07E-05	-0.50431968	21	2.1	Pcsk2	Proteolysis
DMR3:138793001	3	138793001	138795000	2000	1	9.80E-05	0.63514948	14	0.7	Dtd1	Transcription
DMR3:139758001	3	139758001	139759000	1000	1	6.00E-05	-0.62154621	13	1.3	Slc24a3	Transport
DMR3:140264001	3	140264001	140265000	1000	1	5.94E-05	1.09547575	5	0.5	AABR07054096.1	
DMR3:140381001	3	140381001	140384000	3000	1	3.45E-05	0.96779298	39	1.3	AABR07054096.1	
DMR3:141182001	3	141182001	141183000	1000	1	3.41E-05	-0.46327073	39	3.9	Kiz	
DMR3:144458001	3	144458001	144460000	2000	1	9.84E-05	-0.87403561	6	0.3	AABR07054232.1	
DMR3:149229001	3	149229001	149231000	2000	1	1.23E-08	1.00266286	14	0.7	Mapre1	Cytoskeleton
DMR3:149852001	3	149852001	149853000	1000	1	7.12E-05	-0.45074734	35	3.5	Cdk5rap1	Signaling
DMR3:150357001	3	150357001	150358000	1000	1	2.92E-05	-0.6655958	16	1.6	Raly	Transcription
DMR3:151098001	3	151098001	151101000	3000	1	2.38E-05	-0.47026328	43	1.433	Gss	Metabolism
DMR3:152120001	3	152120001	152121000	1000	1	1.44E-05	-0.81545192	14	1.4		
DMR3:156306001	3	156306001	156307000	1000	1	1.11E-05	0.44163882	15	1.5		
DMR3:159536001	3	159536001	159538000	2000	1	5.33E-05	0.57885513	15	0.75		
DMR3:159698001	3	159698001	159700000	2000	1	2.26E-05	-0.4013988	21	1.05	Tox2	
DMR3:160631001	3	160631001	160632000	1000	1	7.55E-06	0.86229983	16	1.6	Semg1	
DMR3:160865001	3	160865001	160866000	1000	1	4.54E-05	-0.44213353	20	2	Rbpjl;Sdc4	Transcription;Cytoskeleton
DMR3:162239001	3	162239001	162243000	4000	1	1.20E-05	0.35020918	44	1.1	AABR07054583.1	
DMR3:162593001	3	162593001	162595000	2000	1	7.88E-05	-0.53928922	55	2.75		
DMR3:163805001	3	163805001	163807000	2000	1	8.82E-06	1.27091934	25	1.25	Znfx1;Gm25878;AC130053.4	Transcription
DMR3:164561001	3	164561001	164562000	1000	1	7.47E-06	-0.53320364	26	2.6		
DMR3:164992001	3	164992001	164993000	1000	1	9.30E-05	0.50470592	12	1.2	Dpm1;Mocs3	Golgi;Metabolism
DMR3:165846001	3	165846001	165847000	1000	1	7.36E-05	-0.48468299	20	2		
DMR3:165900001	3	165900001	165901000	1000	1	1.90E-06	0.65556131	21	2.1		
DMR3:166220001	3	166220001	166222000	2000	1	3.37E-08	0.54321257	16	0.8		
DMR3:166890001	3	166890001	166891000	1000	1	5.91E-08	-0.49353343	16	1.6		
DMR3:167094001	3	167094001	167095000	1000	1	6.59E-05	-0.51058358	28	2.8		
DMR3:168971001	3	168971001	168973000	2000	1	4.44E-06	0.96606948	24	1.2		
DMR3:170003001	3	170003001	170004000	1000	1	5.20E-07	0.42302452	12	1.2		
DMR3:170572001	3	170572001	170574000	2000	1	4.13E-05	0.48190736	21	1.05		
DMR3:171366001	3	171366001	171368000	2000	1	1.09E-05	-0.56589685	72	3.6		
DMR3:171910001	3	171910001	171912000	2000	1	7.52E-05	0.5303534	14	0.7	Apcdd1l	
DMR3:172181001	3	172181001	172183000	2000	1	1.58E-05	-0.62448622	47	2.35	Stx16	Transcription
DMR3:173061001	3	173061001	173064000	3000	1	1.88E-06	0.59519023	21	0.7		
DMR3:173440001	3	173440001	173441000	1000	1	8.16E-05	-0.50174217	10	1		
DMR3:174869001	3	174869001	174871000	2000	1	4.67E-08	0.4984718	24	1.2		
DMR3:175147001	3	175147001	175151000	4000	1	8.26E-05	0.63324266	39	0.975	Cdh4	Extracellular Matrix
DMR3:176278001	3	176278001	176280000	2000	1	3.08E-05	0.5656431	21	1.05	Bhlhe23	Transcription
DMR4:411001	4	411001	412000	1000	1	6.91E-05	-0.52531091	18	1.8		
DMR4:468001	4	468001	469000	1000	1	5.82E-05	-0.50713357	28	2.8	Cnpy1	
DMR4:699001	4	699001	701000	2000	1	3.51E-05	-0.60143875	40	2	Rbm33	
DMR4:1048001	4	1048001	1049000	1000	1	1.24E-05	-0.64528689	1	0.1		
DMR4:1213001	4	1213001	1214000	1000	1	9.23E-05	-0.60757514	10	1		
DMR4:3973001	4	3973001	3974000	1000	1	4.87E-05	0.78686674	7	0.7	Paxip1	
DMR4:3975001	4	3975001	3977000	2000	1	8.32E-05	-0.65892745	40	2	Paxip1	
DMR4:4170001	4	4170001	4171000	1000	1	2.01E-05	-0.55599007	37	3.7	Dpp6	Proteolysis
DMR4:5934001	4	5934001	5935000	1000	1	6.82E-05	-0.64000919	17	1.7		
DMR4:6305001	4	6305001	6306000	1000	1	3.17E-05	-0.50731701	21	2.1	Galnt11;AABR07059168.2	Metabolism
DMR4:10493001	4	10493001	10494000	1000	1	5.74E-05	0.32197967	12	1.2	Gsap	Receptor
DMR4:11641001	4	11641001	11642000	1000	1	4.39E-07	0.67100938	7	0.7		

DMR4:11955001	4	11955001	11956000	1000	1	9.43E-05	0.91779266	8	0.8		
DMR4:12611001	4	12611001	12612000	1000	1	1.04E-05	-0.90843129	2	0.2		
DMR4:14858001	4	14858001	14860000	2000	1	1.18E-06	0.84780808	13	0.65		
DMR4:19791001	4	19791001	19792000	1000	1	1.50E-06	-1.0825012	10	1		
DMR4:21584001	4	21584001	21585000	1000	1	7.83E-05	0.6376686	9	0.9	RGD1563349	EST
DMR4:22376001	4	22376001	22378000	2000	1	1.26E-05	0.51475222	14	0.7	Abcb4	Receptor
DMR4:24620001	4	24620001	24621000	1000	1	9.84E-06	-0.8886098	8	0.8	LOC100912481	Transcription
DMR4:27165001	4	27165001	27167000	2000	1	6.15E-05	0.6648903	21	1.05	Cyp51	Electron Transport
DMR4:29086001	4	29086001	29087000	1000	1	4.25E-07	0.39349013	6	0.6	Bet1	Golgi
DMR4:29447001	4	29447001	29449000	2000	2	1.04E-06	0.61896293	12	0.6		
DMR4:30411001	4	30411001	30413000	2000	1	2.64E-06	-0.44577255	19	0.95	Asb4	Protein Binding
DMR4:34218001	4	34218001	34219000	1000	1	8.06E-05	-0.8034467	15	1.5		
DMR4:38583001	4	38583001	38584000	1000	1	6.78E-05	0.63127891	19	1.9		
DMR4:40103001	4	40103001	40104000	1000	1	2.47E-06	-0.52099085	32	3.2	Bmt2	
DMR4:41102001	4	41102001	41103000	1000	1	2.92E-07	0.2887166	2	0.2		
DMR4:44582001	4	44582001	44583000	1000	1	5.98E-05	0.67661834	6	0.6	Cav2	Epigenetic
DMR4:44708001	4	44708001	44709000	1000	1	1.49E-05	0.43990504	5	0.5		
DMR4:45255001	4	45255001	45256000	1000	1	5.35E-05	0.79546815	7	0.7	ST7	Unknown
DMR4:47986001	4	47986001	47987000	1000	1	4.16E-06	0.61016127	1	0.1		
DMR4:50453001	4	50453001	50454000	1000	1	1.44E-05	-0.54564712	14	1.4	Cadps2	Metabolism
DMR4:51020001	4	51020001	51021000	1000	1	9.37E-05	0.48642561	8	0.8		
DMR4:51838001	4	51838001	51840000	2000	1	8.12E-11	-0.79185099	33	1.65	Gpr37	Receptor
DMR4:55325001	4	55325001	55326000	1000	1	3.68E-05	0.7036324	9	0.9	Grm8	Receptor
DMR4:55384001	4	55384001	55385000	1000	1	8.25E-05	-0.60495697	22	2.2	Grm8	Receptor
DMR4:56649001	4	56649001	56650000	1000	1	2.22E-05	-0.76234712	14	1.4	Calu;Opn1sw	Signaling;Receptor
DMR4:59095001	4	59095001	59098000	3000	1	1.19E-05	0.57661972	32	1.067		
DMR4:62134001	4	62134001	62136000	2000	1	1.80E-06	0.52266493	6	0.3		
DMR4:62201001	4	62201001	62202000	1000	1	1.65E-05	0.67860294	25	2.5	AC103335.1	
DMR4:62531001	4	62531001	62533000	2000	1	4.45E-05	-0.52281404	26	1.3	RGD1565367	
DMR4:62939001	4	62939001	62940000	1000	1	4.52E-05	-0.4456399	17	1.7		
DMR4:63830001	4	63830001	63831000	1000	1	2.06E-07	0.5250761	5	0.5		
DMR4:67830001	4	67830001	67831000	1000	1	3.83E-05	-0.59738989	4	0.4		
DMR4:69365001	4	69365001	69366000	1000	1	3.31E-05	0.90079734	11	1.1		
DMR4:72232001	4	72232001	72233000	1000	1	5.74E-05	-0.50042026	4	0.4	Olr806;Olr807	Receptor
DMR4:76963001	4	76963001	76964000	1000	1	3.96E-07	0.59934146	3	0.3	AABR07060487.1	
DMR4:77894001	4	77894001	77895000	1000	1	9.40E-05	0.707119	6	0.6		
DMR4:78294001	4	78294001	78295000	1000	1	9.25E-05	0.89219532	5	0.5	Gimap8	Unknown
DMR4:79301001	4	79301001	79302000	1000	1	6.87E-05	-0.41058143	7	0.7		
DMR4:80940001	4	80940001	80942000	2000	1	6.59E-07	-0.66533389	52	2.6		
DMR4:82410001	4	82410001	82411000	1000	1	1.70E-05	1.091858	10	1		
DMR4:85940001	4	85940001	85941000	1000	1	1.13E-07	-1.37249748	13	1.3		
DMR4:87157001	4	87157001	87158000	1000	1	1.54E-05	0.704199	9	0.9	Fkbp9	Signaling
DMR4:89101001	4	89101001	89102000	1000	1	9.88E-05	-0.56219331	26	2.6	Herc3	Metabolism
DMR4:89873001	4	89873001	89874000	1000	1	6.03E-05	-0.46293884	49	4.9		
DMR4:90979001	4	90979001	90980000	1000	1	8.35E-05	-0.67109238	8	0.8		
DMR4:96386001	4	96386001	96387000	1000	1	5.27E-06	-0.79978017	19	1.9		
DMR4:98950001	4	98950001	98952000	2000	1	3.72E-05	0.69225827	22	1.1		
DMR4:99924001	4	99924001	99925000	1000	1	3.94E-05	-0.49617315	7	0.7		
DMR4:101005001	4	101005001	101006000	1000	1	5.47E-05	0.66974633	6	0.6	Dnah6;5S_rRNA	
DMR4:113052001	4	113052001	113054000	2000	1	6.04E-05	0.88049278	14	0.7		
DMR4:115125001	4	115125001	115127000	2000	1	1.79E-06	0.67839087	18	0.9	Tet3	
DMR4:115862001	4	115862001	115864000	2000	1	2.11E-05	1.18065294	20	1	Dysf	Transport
DMR4:116206001	4	116206001	116208000	2000	1	2.00E-05	0.3877707	9	0.45		
DMR4:117311001	4	117311001	117312000	1000	1	1.86E-05	-0.41388678	9	0.9	5S_rRNA	
DMR4:117493001	4	117493001	117495000	2000	1	2.93E-05	-0.47454382	70	3.5	Nat8f3	
DMR4:118011001	4	118011001	118012000	1000	1	2.94E-07	0.40508578	5	0.5	Tgfa	Growth Factors & Cytokines
DMR4:120069001	4	120069001	120070000	1000	1	1.99E-06	0.7547912	9	0.9		
DMR4:122420001	4	122420001	122421000	1000	1	1.04E-05	-0.58637653	18	1.8		
DMR4:123501001	4	123501001	123502000	1000	1	6.41E-05	-0.27396566	6	0.6	Slc41a3	Transport
DMR4:123604001	4	123604001	123605000	1000	1	3.28E-05	-0.47458718	10	1	Grip2	Signaling
DMR4:124154001	4	124154001	124155000	1000	1	2.52E-05	0.64811017	8	0.8		
DMR4:124617001	4	124617001	124618000	1000	1	2.09E-05	0.5374932	4	0.4		
DMR4:130390001	4	130390001	130391000	1000	1	2.15E-05	-0.59394461	24	2.4	Mitf	Transcription
DMR4:130479001	4	130479001	130481000	2000	1	4.65E-06	0.48239551	25	1.25		
DMR4:130615001	4	130615001	130618000	3000	2	5.77E-05	0.64765191	29	0.967		
DMR4:132166001	4	132166001	132167000	1000	1	3.63E-05	0.66651491	2	0.2	Prok2	Signaling
DMR4:134351001	4	134351001	134352000	1000	1	7.28E-05	1.0259566	7	0.7		
DMR4:134378001	4	134378001	134379000	1000	1	6.86E-05	0.90921672	9	0.9		
DMR4:137122001	4	137122001	137123000	1000	1	2.27E-05	0.66626108	6	0.6		
DMR4:138229001	4	138229001	138231000	2000	1	1.91E-06	-0.94683325	25	1.25		
DMR4:149150001	4	149150001	149152000	2000	1	6.11E-05	0.59395188	18	0.9		
DMR4:150412001	4	150412001	150414000	2000	1	9.70E-05	0.82767629	13	0.65		

DMR4:150524001	4	150524001	150525000	1000	1	5.58E-05	-0.54859222	20	2	U4;Zfp9	Transcription
DMR4:152217001	4	152217001	152218000	1000	1	1.08E-05	1.0256749	8	0.8	Erc1	Signaling
DMR4:153468001	4	153468001	153469000	1000	1	5.34E-05	0.63342138	19	1.9	Bid	
DMR4:153563001	4	153563001	153564000	1000	1	9.27E-05	-0.59726819	22	2.2	Mical3	
DMR4:154409001	4	154409001	154410000	1000	1	2.73E-05	-0.54319008	47	4.7	Mug1	Immune
DMR4:155164001	4	155164001	155165000	1000	1	8.68E-06	0.59995065	6	0.6	AABR07061962.1	
DMR4:155167001	4	155167001	155168000	1000	1	1.37E-05	0.57645295	9	0.9	AABR07061962.1	
DMR4:157067001	4	157067001	157068000	1000	1	1.77E-05	-0.69128133	9	0.9	Clstn3;AC128967.2	Extracellular Matrix
DMR4:157794001	4	157794001	157796000	2000	1	9.93E-05	-0.67388027	30	1.5	Tuba3a	Cytoskeleton
DMR4:158609001	4	158609001	158610000	1000	1	8.86E-05	0.53254387	7	0.7		
DMR4:160305001	4	160305001	160307000	2000	1	7.15E-05	-0.65312524	37	1.85	Prmt8	Epigenetic
DMR4:161583001	4	161583001	161584000	1000	1	1.98E-07	0.48186758	10	1	Tead4	Transcription
DMR4:162022001	4	162022001	162023000	1000	1	3.57E-05	0.93463212	13	1.3	Klrb1b	Immune
DMR4:162566001	4	162566001	162567000	1000	1	5.09E-06	-0.47413272	47	4.7		
DMR4:165141001	4	165141001	165142000	1000	1	7.12E-05	-0.4181225	57	5.7		
DMR4:168117001	4	168117001	168118000	1000	1	8.06E-06	0.8107266	14	1.4		
DMR4:168905001	4	168905001	168907000	2000	1	8.17E-05	-0.44886845	30	1.5	Hebp1;AABR07062323.1	Metabolism
DMR4:170725001	4	170725001	170726000	1000	1	2.23E-06	0.50421601	3	0.3	Gucy2c	Metabolism
DMR4:171998001	4	171998001	171999000	1000	1	6.26E-07	-0.50613797	26	2.6	Slc15a5	Transport
DMR4:174187001	4	174187001	174188000	1000	1	5.06E-05	0.73971099	7	0.7	Plcz1;Capza3	Metabolism;Cytoskeleton
DMR4:174512001	4	174512001	174514000	2000	1	4.66E-05	0.40092264	14	0.7		
DMR4:175440001	4	175440001	175441000	1000	1	4.88E-05	-0.70255026	27	2.7	Pde3a	Signaling
DMR4:176296001	4	176296001	176298000	2000	1	4.40E-11	-0.6078992	91	4.55	Slc21a4	
DMR4:176387001	4	176387001	176388000	1000	1	3.57E-05	0.52099214	4	0.4	Slco1a6	
DMR4:177153001	4	177153001	177154000	1000	1	6.01E-06	0.52627298	13	1.3	St8sia1	Metabolism
DMR4:177493001	4	177493001	177494000	1000	1	8.15E-05	-0.52299863	4	0.4		
DMR4:177558001	4	177558001	177560000	2000	1	2.24E-05	0.50350588	24	1.2		
DMR4:177613001	4	177613001	177614000	1000	1	8.39E-07	0.72225108	9	0.9		
DMR4:180761001	4	180761001	180762000	1000	1	6.54E-05	-0.45661233	20	2		
DMR4:181666001	4	181666001	181667000	1000	1	2.96E-06	-0.52471607	14	1.4	AABR07062539.2	
DMR5:210001	5	210001	211000	1000	1	6.85E-05	1.05967696	10	1		
DMR5:1788001	5	1788001	1789000	1000	1	9.30E-05	-0.80644671	49	4.9		
DMR5:1894001	5	1894001	1896000	2000	1	2.73E-05	-0.77441643	16	0.8		
DMR5:3572001	5	3572001	3573000	1000	1	5.41E-05	0.99577895	4	0.4		
DMR5:4013001	5	4013001	4016000	3000	1	3.35E-05	0.38848178	15	0.5		
DMR5:5033001	5	5033001	5034000	1000	1	2.81E-05	0.62746496	11	1.1		
DMR5:5346001	5	5346001	5347000	1000	1	2.09E-06	0.50695395	40	4		
DMR5:5890001	5	5890001	5891000	1000	1	3.61E-06	0.97879091	8	0.8	Slco5a1	Metabolism
DMR5:8775001	5	8775001	8777000	2000	1	7.79E-05	-0.43546181	46	2.3	Cspp1	Cell Cycle
DMR5:8905001	5	8905001	8906000	1000	1	4.75E-08	0.67339435	8	0.8		
DMR5:12597001	5	12597001	12598000	1000	1	6.59E-05	0.95605938	7	0.7		
DMR5:15161001	5	15161001	15162000	1000	1	1.11E-06	-1.05408529	3	0.3		
DMR5:15499001	5	15499001	15500000	1000	1	2.43E-06	0.77727198	10	1		
DMR5:16714001	5	16714001	16716000	2000	1	9.77E-05	0.33534663	15	0.75	Rps20;Gm24016	Translation
DMR5:16907001	5	16907001	16908000	1000	1	5.76E-05	0.35484608	7	0.7	Sdr16c5	Metabolism
DMR5:19051001	5	19051001	19052000	1000	1	4.56E-05	-0.7016815	4	0.4		
DMR5:21930001	5	21930001	21931000	1000	1	9.87E-05	-0.53847515	45	4.5	Chd7	Epigenetic
DMR5:23859001	5	23859001	23860000	1000	1	9.20E-06	-0.76311786	5	0.5		
DMR5:24158001	5	24158001	24159000	1000	1	9.12E-05	-0.66890803	17	1.7	MGC94199	EST
DMR5:24347001	5	24347001	24348000	1000	1	5.23E-05	-0.85997172	17	1.7		
DMR5:25205001	5	25205001	25206000	1000	1	1.29E-05	0.61889953	10	1	Rad54b;AABR07047249.1	Transcription
DMR5:26209001	5	26209001	26210000	1000	1	3.36E-05	0.63622405	5	0.5		
DMR5:27070001	5	27070001	27072000	2000	1	1.02E-05	-0.27449139	10	0.5		
DMR5:27470001	5	27470001	27471000	1000	1	5.95E-05	-0.91136531	8	0.8		
DMR5:36359001	5	36359001	36360000	1000	1	3.71E-05	-0.71055132	6	0.6		
DMR5:37051001	5	37051001	37052000	1000	1	5.04E-06	0.63485835	7	0.7		
DMR5:40247001	5	40247001	40249000	2000	1	6.56E-06	0.47381353	16	0.8	Fut9	Metabolism
DMR5:44862001	5	44862001	44863000	1000	1	6.26E-05	-0.52215319	24	2.4		
DMR5:45470001	5	45470001	45471000	1000	1	4.08E-05	0.9256225	2	0.2		
DMR5:47133001	5	47133001	47134000	1000	1	1.14E-05	-0.76130189	14	1.4		
DMR5:47419001	5	47419001	47420000	1000	1	9.65E-05	0.45489967	16	1.6		
DMR5:47818001	5	47818001	47820000	2000	1	1.45E-05	0.80379117	21	1.05		
DMR5:57435001	5	57435001	57436000	1000	1	1.29E-05	-0.52392297	13	1.3	Aqp3;Nol6	Transport;Transcription
DMR5:57599001	5	57599001	57600000	1000	1	1.94E-05	-0.49185786	20	2	Ubap2	
DMR5:57883001	5	57883001	57885000	2000	1	7.80E-05	-0.57310769	35	1.75	Myorg;RGD1561916	
DMR5:58327001	5	58327001	58329000	2000	1	4.63E-05	-0.48662592	17	0.85		
DMR5:58707001	5	58707001	58708000	1000	1	4.99E-05	-0.85944207	16	1.6	Unc13b;AABR07048046.1	Receptor
DMR5:62392001	5	62392001	62394000	2000	1	5.74E-05	-0.54450513	34	1.7	Gabbr2	Receptor
DMR5:63936001	5	63936001	63937000	1000	1	4.78E-05	1.00597452	10	1	Erp44	Metabolism
DMR5:66763001	5	66763001	66765000	2000	1	8.18E-06	0.68469596	18	0.9		
DMR5:68459001	5	68459001	68460000	1000	1	1.44E-06	0.59616993	7	0.7		
DMR5:69993001	5	69993001	69994000	1000	1	4.80E-06	-0.45981693	0	0	Abca1	Receptor

DMR5:71123001	5	71123001	71124000	1000	1	2.98E-05	0.6002293	10	1		
DMR5:74014001	5	74014001	74015000	1000	1	1.02E-06	0.62004954	16	1.6	Frrs1l	
DMR5:75529001	5	75529001	75533000	4000	1	7.08E-05	0.52405095	44	1.1		
DMR5:75708001	5	75708001	75710000	2000	1	1.43E-05	-0.54085209	30	1.5		
DMR5:75797001	5	75797001	75798000	1000	1	1.38E-05	0.89038491	9	0.9		
DMR5:78010001	5	78010001	78011000	1000	1	2.60E-05	-0.45940033	9	0.9	AABR07048483.1	
DMR5:78640001	5	78640001	78642000	2000	1	2.19E-06	0.58761619	31	1.55		
DMR5:80164001	5	80164001	80166000	2000	1	9.16E-05	0.64961627	27	1.35		
DMR5:85613001	5	85613001	85614000	1000	1	4.96E-05	0.8454571	8	0.8	AABR07048636.1	
DMR5:86611001	5	86611001	86612000	1000	1	7.83E-05	-0.79994983	5	0.5	Megf9	Extracellular Matrix
DMR5:86796001	5	86796001	86801000	5000	1	6.77E-05	0.33504881	16	0.32		
DMR5:88020001	5	88020001	88021000	1000	1	9.86E-05	-0.71881407	4	0.4		
DMR5:88815001	5	88815001	88816000	1000	1	8.09E-05	-0.77399507	8	0.8		
DMR5:95897001	5	95897001	95898000	1000	1	3.30E-06	-0.89827262	4	0.4		
DMR5:96837001	5	96837001	96838000	1000	1	2.62E-06	-0.89730668	2	0.2		
DMR5:97367001	5	97367001	97368000	1000	1	9.48E-06	-0.748335	4	0.4		
DMR5:102937001	5	102937001	102938000	1000	1	8.18E-05	-0.71787259	0	0		
DMR5:105219001	5	105219001	105221000	2000	1	1.17E-05	0.68904709	8	0.4	LOC100911372;Acer2	Translation;Metabolism
DMR5:105508001	5	105508001	105510000	2000	1	7.76E-05	-0.5156361	26	1.3	Slc24a2	Transport
DMR5:107321001	5	107321001	107322000	1000	1	7.99E-05	0.830698	10	1	Klhl9;AABR07049134.1	Transcription
DMR5:113044001	5	113044001	113045000	1000	1	1.26E-05	-0.48591557	52	5.2		
DMR5:114204001	5	114204001	114205000	1000	1	6.03E-08	0.52699002	2	0.2	AABR07049292.1	
DMR5:115176001	5	115176001	115177000	1000	1	6.49E-07	-0.59930835	27	2.7	Cyp2j10	
DMR5:116435001	5	116435001	116436000	1000	1	3.40E-06	-0.50244855	14	1.4	Nfia	Transcription
DMR5:118366001	5	118366001	118368000	2000	1	1.73E-05	0.4248017	13	0.65		
DMR5:119298001	5	119298001	119300000	2000	1	3.51E-05	-0.58866037	5	0.25		
DMR5:120092001	5	120092001	120093000	1000	1	3.20E-05	0.41439237	6	0.6	Jak1;U1	Signaling
DMR5:121415001	5	121415001	121416000	1000	1	1.95E-05	-0.4341745	23	2.3		
DMR5:121804001	5	121804001	121805000	1000	1	2.46E-06	-0.85711954	10	1		
DMR5:122127001	5	122127001	122129000	2000	1	2.58E-06	-0.66336459	34	1.7	Pde4b;AABR07049466.1	Metabolism
DMR5:123214001	5	123214001	123215000	1000	1	9.65E-05	0.65741567	20	2		
DMR5:124364001	5	124364001	124365000	1000	1	1.81E-05	-0.62666247	20	2	C8a	Immune
DMR5:125286001	5	125286001	125287000	1000	1	1.38E-06	1.0380338	6	0.6		
DMR5:127261001	5	127261001	127263000	2000	1	9.63E-05	-0.57515567	40	2	Dmrtb1	Transcription
DMR5:128084001	5	128084001	128086000	2000	1	9.34E-05	-0.94661415	16	0.8	AABR07049550.1;Tut4	
DMR5:129230001	5	129230001	129231000	1000	1	2.96E-06	0.45816433	15	1.5		
DMR5:129905001	5	129905001	129906000	1000	1	1.35E-05	-0.78029083	15	1.5		
DMR5:130765001	5	130765001	130766000	1000	1	4.66E-07	0.46248758	2	0.2		
DMR5:131360001	5	131360001	131361000	1000	1	2.24E-05	0.42582217	3	0.3		
DMR5:132045001	5	132045001	132046000	1000	1	4.38E-05	0.65117262	5	0.5		
DMR5:133240001	5	133240001	133242000	2000	1	2.04E-06	0.57894192	15	0.75	Trabd2b	
DMR5:135974001	5	135974001	135977000	3000	1	5.85E-05	-0.53747445	86	2.867	Ptch2	
DMR5:136280001	5	136280001	136281000	1000	1	1.44E-05	-0.7510741	21	2.1		
DMR5:137010001	5	137010001	137012000	2000	1	9.24E-05	-0.62796543	33	1.65	Kdm4a	Epigenetic
DMR5:137139001	5	137139001	137140000	1000	1	3.81E-06	-0.67096135	18	1.8		
DMR5:137416001	5	137416001	137417000	1000	1	9.47E-05	-0.39676038	36	3.6	Cfap57	
DMR5:138091001	5	138091001	138092000	1000	1	4.42E-05	1.03847475	8	0.8		
DMR5:139766001	5	139766001	139767000	1000	1	5.18E-05	-0.96282386	14	1.4		
DMR5:141903001	5	141903001	141904000	1000	1	8.10E-05	-0.53357303	30	3		
DMR5:143155001	5	143155001	143156000	1000	1	7.46E-05	-0.49126406	18	1.8		
DMR5:143892001	5	143892001	143894000	2000	1	8.03E-05	-0.46401666	22	1.1		
DMR5:145542001	5	145542001	145543000	1000	1	8.28E-05	-0.42538645	5	0.5		
DMR5:145648001	5	145648001	145649000	1000	1	3.61E-05	0.62748697	8	0.8		
DMR5:145671001	5	145671001	145672000	1000	1	3.99E-05	-0.56353411	19	1.9		
DMR5:146540001	5	146540001	146542000	2000	1	8.41E-05	-0.67189614	41	2.05	Csmd2	Unknown
DMR5:146857001	5	146857001	146858000	1000	1	1.81E-05	-0.44694371	34	3.4		
DMR5:148205001	5	148205001	148206000	1000	1	1.04E-05	0.50594718	8	0.8	Adgrb2	
DMR5:148207001	5	148207001	148208000	1000	1	1.51E-05	-0.44278494	16	1.6	Adgrb2	
DMR5:148389001	5	148389001	148391000	2000	1	3.20E-06	-0.53694399	40	2	Tinagl1	Signaling
DMR5:149094001	5	149094001	149095000	1000	1	1.57E-05	0.39272797	11	1.1	Matn1	Extracellular Matrix
DMR5:149484001	5	149484001	149485000	1000	1	2.66E-06	0.64256294	4	0.4		
DMR5:151288001	5	151288001	151290000	2000	1	1.87E-06	-0.4155188	54	2.7		
DMR5:151724001	5	151724001	151726000	2000	1	1.89E-07	0.37274461	19	0.95		
DMR5:152873001	5	152873001	152876000	3000	1	3.20E-05	-0.61930887	17	0.567	Man1c1	Metabolism
DMR5:153483001	5	153483001	153484000	1000	1	3.29E-05	-0.42799069	36	3.6		
DMR5:153974001	5	153974001	153975000	1000	1	6.00E-05	0.40862779	16	1.6	LOC500567	
DMR5:154001001	5	154001001	154002000	1000	1	5.87E-05	0.46273564	8	0.8		
DMR5:156571001	5	156571001	156574000	3000	1	3.09E-06	0.86360953	33	1.1	Eif4g3;Hp1bp3	Transcription
DMR5:156989001	5	156989001	156990000	1000	1	5.32E-06	1.13913373	27	2.7		
DMR5:157275001	5	157275001	157276000	1000	1	5.93E-05	-0.28545316	12	1.2	Pla2g5;U1;Pla2g2a	Metabolism;Signaling
DMR5:158219001	5	158219001	158221000	2000	1	7.98E-05	-0.5775396	38	1.9	Pax7	Transcription
DMR5:158542001	5	158542001	158544000	2000	1	3.21E-05	-0.46331963	43	2.15	Igsf21	Immune

DMR5:158931001	5	158931001	158932000	1000	1	2.12E-05	-0.41203107	19	1.9		
DMR5:158987001	5	158987001	158989000	2000	1	5.81E-05	0.74017815	38	1.9		
DMR5:159306001	5	159306001	159307000	1000	1	9.80E-05	0.40578072	9	0.9	Padi3	Metabolism
DMR5:160026001	5	160026001	160028000	2000	1	2.86E-05	-0.45923741	58	2.9	Spen	Transcription
DMR5:160670001	5	160670001	160673000	3000	1	2.11E-05	-0.72474717	71	2.367	Kazn	
DMR5:160946001	5	160946001	160947000	1000	1	2.34E-05	0.41738793	3	0.3		
DMR5:161518001	5	161518001	161519000	1000	1	8.63E-05	0.529937	3	0.3	AABR07050336.1	
DMR5:161699001	5	161699001	161700000	1000	1	1.62E-06	0.54055454	10	1		
DMR5:161806001	5	161806001	161807000	1000	1	2.09E-05	-0.7952465	15	1.5	Prdm2	Transcription
DMR5:162105001	5	162105001	162107000	2000	1	7.27E-05	0.59692016	64	3.2		
DMR5:162650001	5	162650001	162652000	2000	1	3.36E-06	1.02004275	12	0.6		
DMR5:162878001	5	162878001	162879000	1000	1	1.05E-06	-0.64661215	9	0.9		
DMR5:163615001	5	163615001	163617000	2000	1	6.98E-05	0.77030278	5	0.25	AABR07050391.2	
DMR5:164653001	5	164653001	164656000	3000	1	3.15E-05	1.08322723	16	0.533	AC097784.1	
DMR5:165432001	5	165432001	165434000	2000	1	3.29E-06	-0.53495317	21	1.05	Masp2;Tardbp;AABR07050449.2	Protease;Transcription
DMR5:166646001	5	166646001	166647000	1000	1	6.33E-05	-0.2560452	8	0.8		
DMR5:166684001	5	166684001	166685000	1000	1	5.25E-05	-0.57691687	23	2.3	Tmem201	
DMR5:167683001	5	167683001	167684000	1000	1	5.34E-05	1.06743904	13	1.3	Slc45a1	Transport
DMR5:168097001	5	168097001	168100000	3000	1	1.03E-05	-0.42949019	65	2.167	Per3	Development
DMR5:168198001	5	168198001	168199000	1000	1	2.21E-06	-0.63757523	24	2.4	Camta1	Transcription
DMR5:168405001	5	168405001	168406000	1000	1	2.73E-05	0.50508182	5	0.5	Camta1	Transcription
DMR5:168533001	5	168533001	168535000	2000	1	2.42E-05	-0.52450782	33	1.65	Camta1	Transcription
DMR5:168590001	5	168590001	168591000	1000	1	7.00E-07	-0.48804752	33	3.3	Camta1	Transcription
DMR5:168738001	5	168738001	168739000	1000	1	5.71E-05	-0.52609562	11	1.1	Camta1	Transcription
DMR5:169617001	5	169617001	169618000	1000	1	1.47E-05	0.65965	30	3	Kcnab2	Transport
DMR5:169722001	5	169722001	169725000	3000	1	9.36E-05	-0.48848017	71	2.367	Nphp4	Development
DMR5:169881001	5	169881001	169884000	3000	1	5.87E-05	0.44521821	12	0.4		
DMR5:170488001	5	170488001	170489000	1000	1	4.13E-06	-0.47376004	29	2.9		
DMR5:170820001	5	170820001	170821000	1000	1	1.93E-06	-0.93878251	7	0.7		
DMR5:170860001	5	170860001	170861000	1000	1	3.70E-05	-0.48475381	16	1.6		
DMR5:171353001	5	171353001	171354000	1000	1	7.77E-05	-0.63324783	14	1.4	Tp73	Transcription
DMR5:171637001	5	171637001	171638000	1000	1	8.22E-06	-0.54433798	11	1.1	Arhgef16	
DMR5:171667001	5	171667001	171670000	3000	1	1.45E-05	-0.59659086	70	2.333	Prdm16	Transcription
DMR5:171796001	5	171796001	171797000	1000	1	3.27E-05	-0.41909734	22	2.2		
DMR5:172669001	5	172669001	172671000	2000	1	7.59E-05	0.41946653	18	0.9	Prkcz	Signaling
DMR5:172756001	5	172756001	172757000	1000	1	3.17E-05	-0.6929845	13	1.3	Prkcz	Signaling
DMR5:173061001	5	173061001	173062000	1000	1	4.35E-11	-0.73404575	15	1.5	Cdk11b	Signaling
DMR5:173497001	5	173497001	173498000	1000	1	8.48E-05	0.56352065	10	1	Mir429;Mir3548;Mir200b	
DMR6:103001	6	103001	104000	1000	1	3.21E-05	-0.89718201	16	1.6		
DMR6:831001	6	831001	833000	2000	1	3.39E-05	-0.40377303	46	2.3	Crim1	Development
DMR6:1393001	6	1393001	1394000	1000	1	1.31E-05	-0.99469262	18	1.8	Heatr5b	Receptor
DMR6:2625001	6	2625001	2628000	3000	1	6.21E-05	0.75304411	40	1.333		
DMR6:2642001	6	2642001	2644000	2000	1	4.13E-05	0.49902833	21	1.05		
DMR6:2944001	6	2944001	2945000	1000	1	7.23E-05	-0.88993841	12	1.2	Dhx57	Transcription
DMR6:3548001	6	3548001	3549000	1000	1	1.05E-05	-0.45235959	13	1.3		
DMR6:4208001	6	4208001	4210000	2000	2	2.56E-05	0.76308709	11	0.55		
DMR6:5460001	6	5460001	5461000	1000	1	3.19E-05	-0.82445371	2	0.2		
DMR6:6041001	6	6041001	6042000	1000	1	4.45E-05	0.66487617	13	1.3		
DMR6:7088001	6	7088001	7089000	1000	1	4.08E-05	0.59244886	7	0.7		
DMR6:8723001	6	8723001	8724000	1000	1	5.89E-05	-0.47259473	29	2.9	Camkmt	
DMR6:8942001	6	8942001	8943000	1000	1	9.37E-05	-0.57924408	29	2.9	Six2	
DMR6:10513001	6	10513001	10514000	1000	1	2.46E-06	0.51761367	10	1	Atp6v1e2	Transport
DMR6:10877001	6	10877001	10878000	1000	1	9.94E-05	-0.53429344	20	2	Mcfd2	Immune
DMR6:12982001	6	12982001	12983000	1000	1	1.09E-05	-0.56014797	22	2.2	Fshr	Receptor
DMR6:15136001	6	15136001	15137000	1000	1	3.11E-06	0.49961358	0	0	Nrxn1	Receptor
DMR6:18191001	6	18191001	18192000	1000	1	1.34E-06	-0.45766675	45	4.5		
DMR6:18648001	6	18648001	18649000	1000	1	1.87E-05	-0.87453766	1	0.1		
DMR6:24142001	6	24142001	24144000	2000	1	8.16E-05	0.73664943	22	1.1		
DMR6:25516001	6	25516001	25518000	2000	1	2.03E-05	0.48360867	19	0.95	Plb1	Metabolism
DMR6:25692001	6	25692001	25693000	1000	1	2.39E-05	-0.82412523	7	0.7	Babam2	
DMR6:26653001	6	26653001	26654000	1000	1	5.77E-08	0.75425713	13	1.3	Slc30a3;Cad	Metabolism
DMR6:27243001	6	27243001	27244000	1000	1	1.28E-05	-0.46865236	21	2.1	Cib4	Signaling
DMR6:27665001	6	27665001	27666000	1000	1	4.34E-05	0.9042309	9	0.9	Rab10	Signaling
DMR6:30117001	6	30117001	30118000	1000	1	2.90E-06	-0.52056093	11	1.1	Itsn2	EST
DMR6:30402001	6	30402001	30404000	2000	1	4.58E-06	-0.45034202	33	1.65		
DMR6:30868001	6	30868001	30869000	1000	1	1.14E-05	0.96475045	7	0.7		
DMR6:33642001	6	33642001	33643000	1000	1	9.57E-05	-0.35112854	3	0.3		
DMR6:34208001	6	34208001	34209000	1000	1	7.92E-05	0.88954214	8	0.8		
DMR6:35969001	6	35969001	35972000	3000	1	7.01E-06	-0.5134548	24	0.8		
DMR6:36588001	6	36588001	36589000	1000	1	1.48E-06	0.59700883	5	0.5		
DMR6:37279001	6	37279001	37280000	1000	1	1.50E-05	-1.02286077	17	1.7		

DMR6:39603001	6	39603001	39605000	2000	1	4.95E-05	0.68861675	14	0.7		
DMR6:40595001	6	40595001	40596000	1000	1	4.64E-05	1.15952274	2	0.2		
DMR6:41449001	6	41449001	41450000	1000	1	6.04E-05	1.04280663	9	0.9		
DMR6:47526001	6	47526001	47527000	1000	1	4.77E-05	-0.85972055	9	0.9		
DMR6:47948001	6	47948001	47949000	1000	1	1.32E-05	-0.66863944	11	1.1	Adi1;Trappc12	Metabolism
DMR6:48807001	6	48807001	48808000	1000	1	4.03E-07	-0.70661871	10	1	Myt1l	Transcription
DMR6:49159001	6	49159001	49160000	1000	1	9.37E-05	0.60028345	10	1	Sntg2	Cytoskeleton
DMR6:49410001	6	49410001	49412000	2000	1	4.92E-06	0.94250833	14	0.7	AABR07063802.1	
DMR6:50267001	6	50267001	50268000	1000	1	1.41E-05	0.81812733	7	0.7		
DMR6:52925001	6	52925001	52926000	1000	1	3.88E-05	-0.66664513	28	2.8		
DMR6:54671001	6	54671001	54672000	1000	1	2.19E-05	0.97181259	4	0.4		
DMR6:55105001	6	55105001	55106000	1000	1	9.98E-06	0.46376635	0	0		
DMR6:55948001	6	55948001	55949000	1000	1	1.60E-05	0.71815585	6	0.6	Crppa	
DMR6:58691001	6	58691001	58692000	1000	1	2.06E-05	-0.79024361	10	1		
DMR6:60456001	6	60456001	60457000	1000	1	1.05E-07	0.9195004	8	0.8		
DMR6:62540001	6	62540001	62542000	2000	1	3.31E-05	-0.79525971	10	0.5		
DMR6:66776001	6	66776001	66778000	2000	1	8.74E-05	0.55645796	31	1.55		
DMR6:69816001	6	69816001	69817000	1000	1	4.68E-06	1.08904187	9	0.9		
DMR6:71875001	6	71875001	71876000	1000	1	1.70E-05	0.96741067	6	0.6		
DMR6:73250001	6	73250001	73252000	2000	1	1.73E-05	0.4921714	13	0.65		
DMR6:73685001	6	73685001	73687000	2000	1	1.47E-05	-0.60821945	19	0.95	Akap6	Signaling
DMR6:75268001	6	75268001	75270000	2000	1	1.38E-07	1.10772378	25	1.25		
DMR6:75471001	6	75471001	75473000	2000	1	1.88E-05	-1.0519593	23	1.15	AABR07064312.1	
DMR6:76290001	6	76290001	76291000	1000	1	7.29E-05	-0.75195376	7	0.7		
DMR6:77340001	6	77340001	77341000	1000	1	9.63E-05	0.97231605	10	1		
DMR6:80663001	6	80663001	80664000	1000	1	5.41E-06	0.55435743	6	0.6		
DMR6:82274001	6	82274001	82275000	1000	1	9.78E-06	-0.63287822	13	1.3		
DMR6:82307001	6	82307001	82308000	1000	1	6.96E-05	-0.91159937	1	0.1		
DMR6:83981001	6	83981001	83983000	2000	1	5.14E-05	0.87151782	9	0.45		
DMR6:84342001	6	84342001	84343000	1000	1	8.67E-05	-0.68915348	6	0.6		
DMR6:84405001	6	84405001	84406000	1000	1	4.65E-05	-0.74759284	4	0.4		
DMR6:91086001	6	91086001	91088000	2000	1	9.28E-06	-0.95211546	31	1.55		
DMR6:94047001	6	94047001	94049000	2000	1	6.59E-07	-0.49297375	64	3.2		
DMR6:95420001	6	95420001	95421000	1000	1	4.20E-06	-0.57924428	42	4.2	Dhrs7l1	
DMR6:96661001	6	96661001	96663000	2000	1	7.92E-07	0.49932511	13	0.65	Prkch	Binding Protein
DMR6:98345001	6	98345001	98346000	1000	1	3.76E-06	0.60092355	13	1.3	Rhoj	Signaling
DMR6:99116001	6	99116001	99117000	1000	1	1.39E-07	-0.59424823	23	2.3	AABR07064873.1	
DMR6:99243001	6	99243001	99245000	2000	1	9.91E-05	-0.56113567	17	0.85	Tex21;AC128637.1	
DMR6:99447001	6	99447001	99448000	1000	1	5.67E-05	0.4152637	2	0.2	Ppp1r36	
DMR6:99492001	6	99492001	99493000	1000	1	4.09E-05	-0.85024994	18	1.8		
DMR6:99725001	6	99725001	99726000	1000	1	6.32E-05	-0.55677376	13	1.3	Sptb	Cytoskeleton
DMR6:103303001	6	103303001	103304000	1000	1	9.47E-05	-0.67501038	7	0.7	Zfp36l1	Transcription
DMR6:103840001	6	103840001	103841000	1000	1	5.43E-05	0.91759741	7	0.7		
DMR6:104528001	6	104528001	104529000	1000	1	1.07E-05	-0.99967425	7	0.7	Susd6	
DMR6:105736001	6	105736001	105737000	1000	1	6.31E-05	-0.48479664	31	3.1	Pcnx1	
DMR6:106926001	6	106926001	106927000	1000	1	3.97E-05	0.51986974	2	0.2	Dpf3	Transcription
DMR6:107208001	6	107208001	107209000	1000	1	1.78E-06	1.05903626	11	1.1	Psen1	Signaling
DMR6:107980001	6	107980001	107982000	2000	1	1.30E-05	0.46237182	15	0.75		
DMR6:108591001	6	108591001	108592000	1000	1	4.82E-09	-0.74476918	13	1.3	Ltbp2	Receptor
DMR6:110834001	6	110834001	110835000	1000	1	5.47E-05	-0.54651879	20	2	LOC108351370	
DMR6:113052001	6	113052001	113053000	1000	1	7.39E-05	-1.13637751	3	0.3	Nrxn3	Receptor
DMR6:113671001	6	113671001	113672000	1000	1	4.53E-05	-0.56489951	7	0.7	Nrxn3	Receptor
DMR6:114361001	6	114361001	114362000	1000	1	8.76E-05	0.73976527	10	1		
DMR6:114512001	6	114512001	114513000	1000	1	2.67E-05	0.45708707	4	0.4		
DMR6:121230001	6	121230001	121231000	1000	1	4.57E-06	0.94434603	7	0.7		
DMR6:122854001	6	122854001	122855000	1000	1	1.25E-07	0.50269916	3	0.3	Eml5	Cytoskeleton
DMR6:123148001	6	123148001	123149000	1000	1	2.39E-05	1.1187688	10	1	AABR07065349.1	
DMR6:123581001	6	123581001	123582000	1000	1	4.63E-05	-0.52248344	15	1.5	Foxn3	
DMR6:124407001	6	124407001	124409000	2000	1	4.91E-06	1.10704823	24	1.2	Ttc7b	Metabolism
DMR6:124473001	6	124473001	124474000	1000	1	7.32E-05	0.32054209	0	0		
DMR6:125118001	6	125118001	125119000	1000	1	1.47E-05	0.53798108	12	1.2		
DMR6:125364001	6	125364001	125365000	1000	1	4.39E-05	0.49814603	9	0.9		
DMR6:125381001	6	125381001	125382000	1000	1	7.65E-05	0.71237677	9	0.9		
DMR6:125918001	6	125918001	125919000	1000	1	5.57E-06	0.5324246	22	2.2	AABR07065406.2	
DMR6:131083001	6	131083001	131084000	1000	1	5.02E-05	0.38640193	10	1		
DMR6:131562001	6	131562001	131564000	2000	1	3.21E-05	-0.44963201	56	2.8		
DMR6:132008001	6	132008001	132009000	1000	1	2.37E-07	0.43099944	2	0.2		
DMR6:132397001	6	132397001	132398000	1000	1	8.58E-05	0.50455021	5	0.5	Eml1	
DMR6:132665001	6	132665001	132666000	1000	1	2.56E-08	1.16721288	11	1.1		
DMR6:133061001	6	133061001	133064000	3000	1	1.45E-08	0.72732396	22	0.733		
DMR6:133598001	6	133598001	133601000	3000	2	2.26E-07	0.45010974	10	0.333		
DMR6:134608001	6	134608001	134609000	1000	1	6.86E-05	0.43422671	7	0.7		

DMR6:135139001	6	135139001	135141000	2000	1	4.29E-08	0.63571216	19	0.95	LOC103690996	
DMR6:135299001	6	135299001	135300000	1000	1	9.31E-05	-0.82143621	12	1.2	Cinp	Cell Cycle
DMR6:136358001	6	136358001	136359000	1000	1	9.99E-07	-0.75431519	34	3.4	Klc1	Cytoskeleton
DMR6:136565001	6	136565001	136566000	1000	1	3.67E-05	-0.39362674	21	2.1	Tdrd9	Transcription
DMR6:137717001	6	137717001	137718000	1000	1	3.10E-05	-0.49026782	46	4.6	Jag2;AABR07065617.1	Growth Factors & Cytokines
DMR6:137740001	6	137740001	137743000	3000	1	9.80E-05	-0.58557515	55	1.833	Jag2;Nudt14	Growth Factors & Cytokines;Transcription
DMR6:138152001	6	138152001	138154000	2000	1	9.68E-06	0.75768527	7	0.35	Ighm	
DMR6:142826001	6	142826001	142827000	1000	1	1.56E-05	-0.63604348	30	3	AABR07065821.1	
DMR6:143191001	6	143191001	143192000	1000	1	6.92E-05	0.97565503	8	0.8	AABR07065837.1	
DMR6:145285001	6	145285001	145286000	1000	1	1.11E-05	-0.45101367	25	2.5		
DMR6:146764001	6	146764001	146765000	1000	1	1.97E-05	1.12512755	7	0.7		
DMR6:146810001	6	146810001	146811000	1000	1	4.63E-05	0.49312152	7	0.7	Abcb5	Transcription
DMR7:663001	7	663001	664000	1000	1	7.01E-05	0.57577147	5	0.5		
DMR7:711001	7	711001	714000	3000	2	1.76E-06	-0.50535264	210	7	5_8S_rRNA	
DMR7:801001	7	801001	802000	1000	1	1.73E-05	-0.39951848	33	3.3		
DMR7:854001	7	854001	855000	1000	1	6.36E-05	1.05939526	11	1.1		
DMR7:933001	7	933001	934000	1000	1	4.70E-05	-0.36410356	6	0.6		
DMR7:1510001	7	1510001	1511000	1000	1	9.18E-05	0.70756492	7	0.7		
DMR7:1624001	7	1624001	1628000	4000	2	3.91E-05	-0.37224932	247	6.175	5_8S_rRNA	
DMR7:1693001	7	1693001	1694000	1000	1	4.35E-05	1.20071635	8	0.8		
DMR7:2171001	7	2171001	2172000	1000	1	9.56E-06	0.3834199	5	0.5		
DMR7:2249001	7	2249001	2252000	3000	1	7.04E-05	-0.44475473	120	4		
DMR7:4012001	7	4012001	4013000	1000	1	2.29E-05	-0.61967245	37	3.7		
DMR7:5833001	7	5833001	5835000	2000	1	4.69E-06	0.45682329	16	0.8		
DMR7:6527001	7	6527001	6528000	1000	1	8.60E-05	0.43410895	11	1.1		
DMR7:7087001	7	7087001	7088000	1000	1	5.21E-06	-0.77796217	7	0.7		
DMR7:12197001	7	12197001	12198000	1000	1	5.74E-05	-0.47998176	67	6.7	Mex3d;AC120291.1	Transcription
DMR7:12239001	7	12239001	12242000	3000	1	9.24E-05	-0.54542456	93	3.1	Adamtsl5;AC120291.3;Reep6;Pcsk4	Receptor;Protease
DMR7:12643001	7	12643001	12645000	2000	1	1.10E-05	0.82762072	59	2.95	Arhgap45;Cfd;Elane;Prtn3;Plppr3	Immune;Protease
DMR7:13056001	7	13056001	13057000	1000	1	3.17E-05	-0.4083308	26	2.6	Mier2;AC115214.1;Plpp2	Development
DMR7:14444001	7	14444001	14445000	1000	1	8.71E-05	0.71863382	10	1	Pglyrp2;Cyp4f39	Receptor;Metabolism
DMR7:15022001	7	15022001	15024000	2000	1	8.17E-06	1.09481806	16	0.8	Zfp871	
DMR7:15570001	7	15570001	15571000	1000	1	1.78E-05	0.62353476	8	0.8	Olr1090	Receptor
DMR7:18095001	7	18095001	18096000	1000	1	5.08E-05	-0.98328936	8	0.8		
DMR7:20638001	7	20638001	20639000	1000	1	7.77E-06	0.63736751	7	0.7	AABR07056156.1	
DMR7:22054001	7	22054001	22057000	3000	1	3.02E-06	-0.53320405	149	4.967		
DMR7:22451001	7	22451001	22453000	2000	1	1.24E-05	-0.43187336	59	2.95	5_8S_rRNA	
DMR7:22485001	7	22485001	22488000	3000	1	2.83E-05	0.29791504	18	0.6	AABR07056330.1	
DMR7:22609001	7	22609001	22611000	2000	1	3.25E-06	0.85420865	23	1.15		
DMR7:23042001	7	23042001	23045000	3000	1	2.89E-05	-0.31821187	23	0.767		
DMR7:23361001	7	23361001	23363000	2000	1	1.42E-06	0.73297508	12	0.6		
DMR7:23729001	7	23729001	23730000	1000	1	1.10E-05	0.42466371	0	0	Syn3	Development
DMR7:25386001	7	25386001	25387000	1000	1	1.02E-05	0.48717275	4	0.4		
DMR7:25524001	7	25524001	25526000	2000	1	4.90E-05	0.57844372	24	1.2		
DMR7:25837001	7	25837001	25838000	1000	1	1.39E-05	-0.5776442	19	1.9	Rfx4	Transcription
DMR7:26737001	7	26737001	26740000	3000	1	4.25E-05	-0.46521768	56	1.867	Chst11	Metabolism
DMR7:26839001	7	26839001	26840000	1000	1	6.67E-05	-0.57581428	19	1.9	Chst11	Metabolism
DMR7:27155001	7	27155001	27156000	1000	1	6.32E-05	-0.5077341	26	2.6		
DMR7:27849001	7	27849001	27851000	2000	1	6.67E-05	0.70775296	19	0.95		
DMR7:27950001	7	27950001	27951000	1000	1	2.71E-05	0.53921667	5	0.5		
DMR7:28799001	7	28799001	28800000	1000	1	4.80E-05	-0.63495995	5	0.5	Washc3	
DMR7:29731001	7	29731001	29733000	2000	1	8.69E-05	0.46421862	17	0.85		
DMR7:29830001	7	29830001	29831000	1000	1	1.50E-05	1.09603508	8	0.8		
DMR7:30114001	7	30114001	30115000	1000	1	1.32E-05	1.13556308	8	0.8	Nr1h4	Receptor
DMR7:30926001	7	30926001	30928000	2000	1	2.54E-05	0.46838588	10	0.5	Anks1b	Receptor
DMR7:32380001	7	32380001	32381000	1000	1	1.38E-05	-0.75529129	5	0.5		
DMR7:33349001	7	33349001	33351000	2000	1	9.35E-05	-0.69767508	20	1		
DMR7:33526001	7	33526001	33527000	1000	1	6.60E-06	-0.85838533	18	1.8		
DMR7:34101001	7	34101001	34103000	2000	1	4.84E-05	-0.44605359	58	2.9	Elk3	Transcription
DMR7:34830001	7	34830001	34831000	1000	1	5.29E-05	-0.5155147	4	0.4	SNORA73	
DMR7:35920001	7	35920001	35922000	2000	1	4.20E-05	-0.50113324	53	2.65	Plxnc1	Signaling
DMR7:36999001	7	36999001	3.70E+07	1000	1	1.58E-07	0.65502008	1	0.1		
DMR7:37466001	7	37466001	37467000	1000	1	4.62E-05	-0.70193671	13	1.3		
DMR7:42336001	7	42336001	42337000	1000	1	9.87E-05	-0.5082897	29	2.9	Kitlg	Growth Factors & Cytokines
DMR7:44343001	7	44343001	44345000	2000	1	7.95E-05	-0.81983641	8	0.4		
DMR7:44370001	7	44370001	44371000	1000	1	1.15E-05	-0.52855724	14	1.4		
DMR7:51463001	7	51463001	51464000	1000	1	9.32E-05	-0.62520418	10	1	Ppp1r12a	Signaling
DMR7:55279001	7	55279001	55280000	1000	1	6.08E-06	-0.48654385	26	2.6		
DMR7:55390001	7	55390001	55391000	1000	1	6.64E-05	-0.56567882	8	0.8		
DMR7:58093001	7	58093001	58095000	2000	1	4.76E-05	-0.47780044	14	0.7	Tph2	Metabolism

DMR7:58625001	7	58625001	58627000	2000	1	4.46E-05	-0.56924589	30	1.5	AABR07057171.1	
DMR7:58674001	7	58674001	58675000	1000	1	3.88E-06	-0.56530627	27	2.7		
DMR7:59020001	7	59020001	59021000	1000	1	4.02E-05	-0.74762458	10	1		
DMR7:59283001	7	59283001	59284000	1000	1	1.78E-05	0.83992355	18	1.8	Ptprr	Signaling
DMR7:60757001	7	60757001	60758000	1000	1	7.64E-06	-0.51914209	30	3	Slc35e3	Transport
DMR7:60816001	7	60816001	60817000	1000	1	4.82E-05	-0.58179764	12	1.2	Nup107	Transport
DMR7:62936001	7	62936001	62937000	1000	1	9.36E-05	-0.75356264	14	1.4	Msrb3	Metabolism
DMR7:62948001	7	62948001	62950000	2000	1	4.23E-05	-0.61712093	37	1.85	Msrb3	Metabolism
DMR7:64241001	7	64241001	64242000	1000	1	6.09E-05	-0.93244524	8	0.8	Srgap1	Signaling
DMR7:66397001	7	66397001	66398000	1000	1	9.58E-06	-0.63326058	11	1.1		
DMR7:66740001	7	66740001	66741000	1000	1	3.27E-05	-0.54328184	11	1.1	Mon2	Proteolysis
DMR7:68006001	7	68006001	68007000	1000	1	7.84E-05	-0.51363651	17	1.7		
DMR7:70194001	7	70194001	70196000	2000	1	7.87E-06	-0.62801813	62	3.1	Atp23	
DMR7:70314001	7	70314001	70316000	2000	1	9.41E-05	-0.59603051	32	1.6	Avil;Tsfm;Eef1akmt3	Cytoskeleton;Transcription
DMR7:70809001	7	70809001	70810000	1000	1	2.85E-05	-0.65903312	13	1.3	R3hdm2;Stac3	Unknown
DMR7:73133001	7	73133001	73135000	2000	1	7.14E-06	0.44011161	8	0.4	Matn2;AABR07057475.1	Cytoskeleton
DMR7:74609001	7	74609001	74610000	1000	1	4.86E-05	0.73353773	7	0.7		
DMR7:75324001	7	75324001	75325000	1000	1	9.32E-06	-0.73593421	21	2.1	Snx31	
DMR7:77083001	7	77083001	77085000	2000	1	2.50E-05	0.84418381	22	1.1	Odf1	Cytoskeleton
DMR7:79333001	7	79333001	79335000	2000	1	4.59E-05	0.45884028	30	1.5		
DMR7:80695001	7	80695001	80696000	1000	1	8.69E-05	-0.41431856	11	1.1	Oxr1	Development
DMR7:83598001	7	83598001	83599000	1000	1	4.15E-05	-1.00633666	5	0.5	Sybu	
DMR7:91633001	7	91633001	91634000	1000	1	4.88E-05	0.68576018	12	1.2		
DMR7:94461001	7	94461001	94462000	1000	1	2.74E-08	-0.54560504	43	4.3		
DMR7:94586001	7	94586001	94587000	1000	1	6.78E-05	0.64156542	8	0.8		
DMR7:97814001	7	97814001	97815000	1000	1	5.13E-05	-0.47965175	22	2.2	Tbc1d31	
DMR7:98027001	7	98027001	98028000	1000	1	1.31E-05	0.84595623	6	0.6	Atad2	Metabolism
DMR7:99152001	7	99152001	99153000	1000	1	4.20E-05	0.46163118	3	0.3	Cyp2b1	Electron Transport
DMR7:99482001	7	99482001	99487000	5000	1	3.52E-05	-0.75516607	259	5.18	5_8S_rRNA	
DMR7:100014001	7	100014001	100016000	2000	1	2.87E-06	0.69917363	14	0.7		
DMR7:101474001	7	101474001	101475000	1000	1	2.09E-05	-0.9225917	5	0.5		
DMR7:111862001	7	111862001	111863000	1000	1	1.92E-05	-0.48188377	42	4.2		
DMR7:119182001	7	119182001	119184000	2000	1	6.19E-05	0.9665881	26	1.3	Foxred2;Eif3d	Metabolism;Transcription
DMR7:119308001	7	119308001	119312000	4000	2	2.49E-05	0.50991444	61	1.525	Cacng2	Transport
DMR7:119462001	7	119462001	119463000	1000	1	6.20E-05	0.72831112	12	1.2		
DMR7:120394001	7	120394001	120395000	1000	1	1.68E-05	-0.546711	25	2.5	AC096473.3;Polr2f;Sox10	Transcription
DMR7:121001001	7	121001001	121003000	2000	1	3.10E-05	0.49428988	18	0.9	Dnal4;Nptxr	Cytoskeleton
DMR7:123310001	7	123310001	123311000	1000	1	4.61E-05	-0.559035	23	2.3	LOC100362109;Mei1	
DMR7:123397001	7	123397001	123399000	2000	1	4.09E-05	-0.36888518	93	4.65	Srebf2	Transcription
DMR7:124794001	7	124794001	124795000	1000	1	9.08E-05	-0.71586616	29	2.9	Efcab6	Signaling
DMR7:126123001	7	126123001	126125000	2000	1	5.73E-05	-0.42624752	39	1.95	Fbln1	Cytoskeleton
DMR7:126171001	7	126171001	126173000	2000	1	9.40E-05	0.57827876	23	1.15	Fbln1	Cytoskeleton
DMR7:127026001	7	127026001	127027000	1000	1	9.97E-05	-0.55714789	24	2.4	Cerk	Signaling
DMR7:127727001	7	127727001	127728000	1000	1	6.30E-05	0.57301539	5	0.5		
DMR7:128011001	7	128011001	128012000	1000	1	2.57E-07	0.73084182	2	0.2	AABR07058618.1	
DMR7:129522001	7	129522001	129523000	1000	1	1.98E-05	0.61060519	13	1.3		
DMR7:129579001	7	129579001	129580000	1000	1	2.13E-05	-0.91757557	14	1.4	AABR07058647.1	
DMR7:130397001	7	130397001	130398000	1000	1	2.70E-08	-0.65401792	37	3.7	Cpt1b;Chkb;AC125982.1	Signaling
DMR7:130700001	7	130700001	130702000	2000	1	4.42E-05	0.39700566	13	0.65		
DMR7:131254001	7	131254001	131255000	1000	1	5.84E-05	0.46985048	9	0.9		
DMR7:132139001	7	132139001	132141000	2000	1	2.29E-05	-0.6689168	59	2.95	Kif21a	Cytoskeleton
DMR7:132868001	7	132868001	132869000	1000	1	1.21E-05	0.5449757	10	1	Lrrk2	Unknown
DMR7:134007001	7	134007001	134009000	2000	1	1.33E-05	1.16686053	27	1.35	Pdzrn4	
DMR7:134233001	7	134233001	134234000	1000	1	4.32E-06	0.69581397	8	0.8		
DMR7:135730001	7	135730001	135732000	2000	1	2.31E-05	-0.50473164	42	2.1		
DMR7:136659001	7	136659001	136661000	2000	1	8.49E-05	0.49880503	17	0.85	Nell2	Signaling
DMR7:137606001	7	137606001	137607000	1000	1	5.62E-06	-0.51647962	22	2.2		
DMR7:138027001	7	138027001	138029000	2000	1	9.11E-05	-0.38908418	30	1.5	Slc38a1	
DMR7:140650001	7	140650001	140653000	3000	1	6.77E-05	0.53240173	39	1.3	Tuba1a	Cytoskeleton
DMR7:140914001	7	140914001	140915000	1000	1	2.43E-05	0.9560314	17	1.7	Kcnh3;U1;Mcrs1;SNORA70	Transport;Transcription
DMR7:141750001	7	141750001	141751000	1000	1	1.75E-05	-0.59097268	42	4.2	Dip2b	Development
DMR7:141848001	7	141848001	141849000	1000	1	5.72E-05	-0.44306709	33	3.3	Dip2b	Development
DMR7:142211001	7	142211001	142212000	1000	1	1.12E-05	-0.53809073	37	3.7	Pou6f1	Transcription
DMR7:143687001	7	143687001	143688000	1000	1	5.75E-05	-0.46928906	14	1.4	Eif4b	Transcription
DMR7:143792001	7	143792001	143793000	1000	1	2.70E-05	-0.67122978	17	1.7	Csad	Metabolism
DMR8:1353001	8	1353001	1354000	1000	1	1.20E-05	-0.47589895	39	3.9		
DMR8:1358001	8	1358001	1359000	1000	1	9.12E-05	-0.52938054	41	4.1		
DMR8:1841001	8	1841001	1844000	3000	1	8.16E-05	-0.75843164	10	0.333	Gria4	Signaling
DMR8:2362001	8	2362001	2364000	2000	1	2.95E-05	0.9245497	5	0.25		
DMR8:5389001	8	5389001	5390000	1000	1	8.16E-05	-0.57854183	17	1.7	Dync2h1	Cytoskeleton
DMR8:7079001	8	7079001	7081000	2000	1	5.85E-05	-0.42238492	29	1.45		

DMR8:12034001	8	12034001	12037000	3000	1	2.29E-05	-0.50779237	55	1.833	Maml2	Transcription
DMR8:15291001	8	15291001	15293000	2000	1	8.57E-06	0.36697206	12	0.6		
DMR8:17485001	8	17485001	17486000	1000	1	6.45E-05	-0.99894834	9	0.9	Naalad2	Protease
DMR8:17652001	8	17652001	17653000	1000	1	5.08E-05	0.44763214	1	0.1	7SK	
DMR8:19307001	8	19307001	19308000	1000	1	2.49E-05	-0.45140409	42	4.2		
DMR8:22629001	8	22629001	22630000	1000	1	9.03E-07	0.49971313	4	0.4	Carm1;Yipf2;Timm29	Metabolism
DMR8:22723001	8	22723001	22724000	1000	1	8.32E-05	-0.59464715	26	2.6	Smarca4;Gm27373;U6	Epigenetic
DMR8:22962001	8	22962001	22964000	2000	1	1.34E-05	-0.61969602	17	0.85	Plppr2;Swsap1;Epor	Receptor
DMR8:23009001	8	23009001	23012000	3000	1	1.02E-06	0.87902008	20	0.667	Rgl3;Ccdc151;Prkcsh	Signaling
DMR8:23224001	8	23224001	23225000	1000	1	8.39E-05	-0.62854336	5	0.5		
DMR8:23352001	8	23352001	23357000	5000	3	1.10E-05	0.61278328	0	0	Anln	
DMR8:26527001	8	26527001	26528000	1000	1	5.73E-05	-0.82189893	7	0.7		
DMR8:27804001	8	27804001	27805000	1000	1	8.32E-05	-0.51440772	27	2.7	B3gat1;Glb1l2	Metabolism
DMR8:28355001	8	28355001	28356000	1000	1	5.66E-05	0.34431503	4	0.4	Igsf9b	
DMR8:30377001	8	30377001	30378000	1000	1	7.69E-06	1.03809113	6	0.6		
DMR8:31200001	8	31200001	31201000	1000	1	1.62E-05	0.42532986	1	0.1		
DMR8:31751001	8	31751001	31752000	1000	1	4.60E-05	0.61272057	4	0.4		
DMR8:32831001	8	32831001	32832000	1000	1	1.38E-05	-0.68631201	12	1.2		
DMR8:33427001	8	33427001	33428000	1000	1	9.54E-05	0.64275426	6	0.6	Kcnj5	Transport
DMR8:34032001	8	34032001	34033000	1000	1	3.27E-05	0.4732703	11	1.1		
DMR8:34077001	8	34077001	34078000	1000	1	7.76E-05	0.66889568	5	0.5		
DMR8:38058001	8	38058001	38059000	1000	1	3.79E-05	-0.37711441	8	0.8		
DMR8:38094001	8	38094001	38095000	1000	1	1.29E-05	-0.85605258	18	1.8		
DMR8:38557001	8	38557001	38558000	1000	1	6.06E-05	-0.92592551	2	0.2	LOC100912405	
DMR8:40171001	8	40171001	40172000	1000	1	1.66E-05	0.49029043	6	0.6		
DMR8:44017001	8	44017001	44019000	2000	1	2.68E-06	0.64728942	10	0.5		
DMR8:44860001	8	44860001	44861000	1000	1	6.29E-05	-0.75083647	17	1.7	Clmp	Receptor
DMR8:45034001	8	45034001	45036000	2000	1	2.58E-05	0.55070922	21	1.05		
DMR8:45924001	8	45924001	45925000	1000	1	8.06E-05	-0.46832571	28	2.8		
DMR8:45970001	8	45970001	45971000	1000	1	5.22E-06	-0.70019989	5	0.5		
DMR8:46055001	8	46055001	46056000	1000	1	7.68E-05	-0.80701919	7	0.7		
DMR8:46168001	8	46168001	46170000	2000	1	8.31E-05	0.62435687	21	1.05		
DMR8:46598001	8	46598001	46600000	2000	1	1.08E-05	0.46331943	25	1.25	AC133265.1;Tecta	Extracellular Matrix
DMR8:47105001	8	47105001	47106000	1000	1	1.55E-05	-0.69033583	4	0.4		
DMR8:47373001	8	47373001	47374000	1000	1	4.65E-05	-0.63996863	12	1.2	Arhgef12	Signaling
DMR8:48070001	8	48070001	48073000	3000	1	1.88E-05	0.46624271	43	1.433		
DMR8:49288001	8	49288001	49289000	1000	1	8.46E-05	1.06116493	9	0.9	Cd3g;Cd3d;Cd3e	Immune;Receptor
DMR8:50488001	8	50488001	50489000	1000	1	2.20E-05	-0.6080039	19	1.9	Sik3	Receptor
DMR8:50796001	8	50796001	50799000	3000	1	8.49E-05	0.66430823	32	1.067		
DMR8:50806001	8	50806001	50807000	1000	1	6.80E-05	0.52649744	7	0.7		
DMR8:50892001	8	50892001	50893000	1000	1	7.81E-05	0.36536606	23	2.3		
DMR8:51188001	8	51188001	51190000	2000	1	5.94E-05	-0.8493863	17	0.85		
DMR8:52254001	8	52254001	52256000	2000	1	3.56E-05	-0.388434	13	0.65		
DMR8:52435001	8	52435001	52436000	1000	1	9.05E-06	0.59952535	13	1.3		
DMR8:52491001	8	52491001	52492000	1000	1	7.42E-05	-0.79830329	5	0.5		
DMR8:52845001	8	52845001	52846000	1000	1	2.68E-05	0.40337331	12	1.2		
DMR8:53474001	8	53474001	53475000	1000	1	1.69E-05	0.79698267	8	0.8		
DMR8:56626001	8	56626001	56628000	2000	1	4.46E-05	-0.7402161	55	2.75	Mir6329	
DMR8:56643001	8	56643001	56645000	2000	1	1.28E-05	1.1380847	25	1.25	Zc3h12c;AABR07070185.1	Transcription
DMR8:61554001	8	61554001	61556000	2000	1	4.86E-05	-0.54351163	69	3.45	Cspg4	Cytoskeleton
DMR8:63123001	8	63123001	63124000	1000	1	9.98E-06	0.4411128	7	0.7	Rpl38	Transcription
DMR8:63585001	8	63585001	63586000	1000	1	1.01E-05	-0.52461673	20	2		
DMR8:64097001	8	64097001	64098000	1000	1	1.37E-05	-0.79288779	6	0.6	Adpgk	Metabolism
DMR8:69908001	8	69908001	69909000	1000	1	2.12E-05	0.76024399	7	0.7		
DMR8:69958001	8	69958001	69959000	1000	1	4.59E-06	0.78228021	5	0.5		
DMR8:72326001	8	72326001	72329000	3000	1	3.72E-07	-0.66616853	46	1.533		
DMR8:72442001	8	72442001	72443000	1000	1	7.68E-05	-0.48587383	22	2.2	Ca12	
DMR8:75539001	8	75539001	75540000	1000	1	7.74E-06	1.30063802	12	1.2	Rora	Receptor
DMR8:77157001	8	77157001	77158000	1000	1	2.60E-06	-0.82310928	3	0.3	Adam10	Protease
DMR8:79836001	8	79836001	79838000	2000	1	7.10E-05	-0.85036081	13	0.65		
DMR8:81514001	8	81514001	81515000	1000	1	4.50E-05	-0.55564503	10	1	AABR07070714.1	
DMR8:85968001	8	85968001	85969000	1000	1	8.58E-07	0.67286002	7	0.7	Cd109	Immune
DMR8:87622001	8	87622001	87623000	1000	1	8.64E-05	0.86534436	9	0.9	Myo6	Cytoskeleton
DMR8:89057001	8	89057001	89058000	1000	1	7.20E-05	-0.71430683	4	0.4		
DMR8:90669001	8	90669001	90672000	3000	1	9.51E-05	0.85023263	30	1	Hmgn3	Epigenetic
DMR8:92854001	8	92854001	92856000	2000	1	3.99E-06	0.51365715	9	0.45		
DMR8:92894001	8	92894001	92895000	1000	1	1.00E-05	-0.73914278	5	0.5		
DMR8:99448001	8	99448001	99449000	1000	1	3.57E-05	-0.79308666	5	0.5		
DMR8:100244001	8	100244001	100246000	2000	1	1.38E-07	0.73076938	12	0.6		
DMR8:107173001	8	107173001	107174000	1000	1	1.43E-05	-0.49052059	19	1.9		
DMR8:107296001	8	107296001	107297000	1000	1	8.52E-06	0.83398055	11	1.1	Pik3cb	Signaling
DMR8:108728001	8	108728001	108732000	4000	2	2.41E-05	0.75215553	43	1.075		

DMR8:110689001	8	110689001	110690000	1000	1	1.59E-05	0.5831152	12	1.2	Ephb1	Receptor
DMR8:110962001	8	110962001	110963000	1000	1	5.69E-08	-0.81991465	19	1.9		
DMR8:111559001	8	111559001	111560000	1000	1	1.05E-05	-0.79431633	18	1.8	Slco2a1	Metabolism
DMR8:112108001	8	112108001	112109000	1000	1	2.64E-05	-0.62915442	13	1.3		
DMR8:113310001	8	113310001	113314000	4000	1	6.13E-05	0.68281146	37	0.925	Cpne4	Development
DMR8:114917001	8	114917001	114919000	2000	1	9.89E-05	-0.49986002	79	3.95	Alas1;Tlr9;Twf2	Metabolism;Receptor;Cytoskeleton
DMR8:115228001	8	115228001	115229000	1000	1	3.39E-06	-0.45191828	40	4	Iqcf3	
DMR8:116274001	8	116274001	116276000	2000	1	7.18E-06	0.40287237	21	1.05	Cacna2d2	Transport
DMR8:116449001	8	116449001	116450000	1000	1	3.52E-05	-0.40150219	22	2.2	Sema3f	Growth Factors & Cytokines
DMR8:119762001	8	119762001	119764000	2000	1	9.78E-05	-0.67998093	21	1.05		
DMR8:120138001	8	120138001	120139000	1000	1	1.27E-05	1.1687921	10	1		
DMR8:120509001	8	120509001	120510000	1000	1	6.37E-06	0.55401427	9	0.9		
DMR8:120521001	8	120521001	120523000	2000	1	8.94E-05	0.42483776	5	0.25		
DMR8:120570001	8	120570001	120571000	1000	1	4.91E-05	0.76223862	8	0.8		
DMR8:121116001	8	121116001	121117000	1000	1	2.47E-05	0.58105743	5	0.5	AC098284.1	
DMR8:121549001	8	121549001	121550000	1000	1	5.34E-05	-0.68731854	4	0.4		
DMR8:123373001	8	123373001	123374000	1000	1	2.93E-05	-0.46972381	19	1.9	Stt3b	
DMR8:123937001	8	123937001	123938000	1000	1	2.60E-05	0.47416456	8	0.8		
DMR8:125047001	8	125047001	125048000	1000	1	6.89E-07	-0.57912973	13	1.3	Rbms3	Epigenetic
DMR8:125551001	8	125551001	125553000	2000	1	1.82E-06	0.57913567	17	0.85	Rbms3	Epigenetic
DMR8:125839001	8	125839001	125840000	1000	1	6.56E-05	0.68142396	2	0.2		
DMR8:126075001	8	126075001	126077000	2000	1	1.34E-05	-0.45786338	57	2.85	AABR07071659.2	
DMR8:126883001	8	126883001	126884000	1000	1	4.25E-05	-0.66269025	68	6.8	5_8S_rRNA	
DMR8:128159001	8	128159001	128160000	1000	1	4.06E-05	-0.53056478	33	3.3	Scn5a;Exog	Transport
DMR8:128171001	8	128171001	128173000	2000	1	1.11E-05	-0.48563716	102	5.1	Scn5a	Transport
DMR8:128390001	8	128390001	128393000	3000	2	3.39E-06	0.44683835	30	1	Scn10a	Transport
DMR8:128990001	8	128990001	128991000	1000	1	7.72E-05	0.56884712	8	0.8	Myrip	Metabolism
DMR8:129535001	8	129535001	129536000	1000	1	1.59E-07	-0.69330676	30	3		
DMR8:130327001	8	130327001	130329000	2000	1	4.38E-05	0.85528827	11	0.55	Sec22c	Transport
DMR8:130816001	8	130816001	130817000	1000	1	5.02E-05	-0.59904968	16	1.6	Ano10	Signaling
DMR8:132238001	8	132238001	132239000	1000	1	3.41E-05	1.08781057	13	1.3	Exosc7;Clec3b	Transcription;Extracellular Matrix
DMR8:132336001	8	132336001	132337000	1000	1	2.54E-05	-0.50066559	3	0.3	Tmem158	Unknown
DMR9:608001	9	608001	609000	1000	1	2.37E-05	-0.76558639	21	2.1	AABR07066020.1	
DMR9:3046001	9	3046001	3047000	1000	1	1.38E-05	-0.71646293	12	1.2	AABR07066114.1	
DMR9:3382001	9	3382001	3383000	1000	1	1.49E-05	0.82389958	5	0.5	Kcnh8;U1	Transport
DMR9:3575001	9	3575001	3576000	1000	1	7.35E-05	-0.61483533	3	0.3	Kcnh8	Transport
DMR9:5228001	9	5228001	5229000	1000	1	3.90E-05	-0.43489631	44	4.4		
DMR9:5278001	9	5278001	5279000	1000	1	4.98E-05	-0.76800609	5	0.5		
DMR9:5701001	9	5701001	5702000	1000	1	2.62E-05	0.756654	8	0.8		
DMR9:7464001	9	7464001	7468000	4000	1	7.72E-05	-0.33544534	198	4.95	5_8S_rRNA	
DMR9:8883001	9	8883001	8884000	1000	1	2.72E-05	0.58236004	9	0.9		
DMR9:10577001	9	10577001	10579000	2000	1	9.27E-05	0.44608544	9	0.45		
DMR9:11355001	9	11355001	11356000	1000	1	3.95E-05	-0.66516316	26	2.6		
DMR9:12374001	9	12374001	12376000	2000	1	9.03E-05	-0.28679977	60	3		
DMR9:12444001	9	12444001	12445000	1000	1	2.22E-06	-0.28256518	38	3.8		
DMR9:12764001	9	12764001	12766000	2000	1	5.51E-07	-0.73331631	42	2.1	Rftn1	Unknown
DMR9:13088001	9	13088001	13090000	2000	1	7.43E-06	-0.68563923	39	1.95	Kif6	Cytoskeleton
DMR9:14042001	9	14042001	14046000	4000	2	8.09E-07	0.50517795	27	0.675		
DMR9:15291001	9	15291001	15292000	1000	1	2.49E-05	-0.50597825	22	2.2	Frs3	Unknown
DMR9:15425001	9	15425001	15426000	1000	1	5.18E-06	-0.86573384	16	1.6		
DMR9:16839001	9	16839001	16841000	2000	1	3.20E-05	-0.49418504	51	2.55	Cul9;Dnph1	
DMR9:17009001	9	17009001	17010000	1000	1	3.60E-06	0.53155503	5	0.5		
DMR9:17707001	9	17707001	17711000	4000	1	3.95E-05	0.39395498	30	0.75	Mrpl14;Tmem63b	Transcription;EST
DMR9:19110001	9	19110001	19112000	2000	1	2.90E-05	0.53779363	10	0.5	Clic5	Transport
DMR9:19822001	9	19822001	19825000	3000	1	3.94E-06	0.42763661	22	0.733	Cyp39a1	Metabolism
DMR9:20120001	9	20120001	20121000	1000	1	1.26E-05	-0.49670072	27	2.7	Adgrf5	
DMR9:21475001	9	21475001	21477000	2000	1	9.01E-05	0.62083634	5	0.25		
DMR9:24860001	9	24860001	24861000	1000	1	2.39E-05	-0.48743169	9	0.9		
DMR9:25362001	9	25362001	25363000	1000	1	3.02E-06	-0.67221641	8	0.8	Tfap2d	Transcription
DMR9:29772001	9	29772001	29773000	1000	1	8.14E-06	0.43529725	4	0.4		
DMR9:31302001	9	31302001	31303000	1000	1	2.79E-07	-0.7848293	22	2.2	Adgrb3	
DMR9:31485001	9	31485001	31486000	1000	1	9.64E-07	1.24488124	14	1.4	Adgrb3	
DMR9:37083001	9	37083001	37085000	2000	1	9.36E-05	-0.68173877	33	1.65	Phf3	
DMR9:37976001	9	37976001	37979000	3000	1	5.19E-05	-0.4775783	79	2.633	Dst	Cell Junction
DMR9:41656001	9	41656001	41657000	1000	1	2.21E-05	-0.67298124	20	2		
DMR9:42488001	9	42488001	42490000	2000	1	1.41E-05	0.69505152	24	1.2		
DMR9:44108001	9	44108001	44110000	2000	1	1.92E-05	-0.53093037	27	1.35	Mgat4a;AC133270.2	Golgi
DMR9:44539001	9	44539001	44541000	2000	1	4.88E-07	0.76213744	20	1	Lyg1;NEWGENE_1308196;Mitd1	
DMR9:44908001	9	44908001	44909000	1000	1	5.22E-05	0.97903095	16	1.6	Aff3	Unknown
DMR9:45528001	9	45528001	45529000	1000	1	5.94E-05	-0.65931582	11	1.1	Chst10	Metabolism

DMR9:46448001	9	46448001	46449000	1000	1	9.53E-05	-0.46208104	25	2.5	Rfx8	Transcription
DMR9:46937001	9	46937001	46940000	3000	1	4.01E-05	0.63677634	35	1.167		
DMR9:47467001	9	47467001	47468000	1000	1	8.29E-06	0.4206632	18	1.8	Slc9a2	Transport
DMR9:51106001	9	51106001	51107000	1000	1	2.38E-05	0.92462239	7	0.7		
DMR9:51447001	9	51447001	51448000	1000	1	8.46E-06	-0.52545778	7	0.7	Gulp1	Development
DMR9:52255001	9	52255001	52256000	1000	1	1.07E-07	-1.31300775	4	0.4		
DMR9:53847001	9	53847001	53848000	1000	1	2.86E-05	0.51457963	4	0.4		
DMR9:54063001	9	54063001	54064000	1000	1	7.46E-06	0.76983925	11	1.1		
DMR9:55089001	9	55089001	55091000	2000	1	5.13E-05	-0.84953771	16	0.8		
DMR9:55368001	9	55368001	55369000	1000	1	4.33E-06	0.88014564	4	0.4		
DMR9:55646001	9	55646001	55647000	1000	1	3.84E-05	0.40879504	3	0.3	Tmeff2	Signaling
DMR9:56608001	9	56608001	56609000	1000	1	5.01E-05	-0.61592015	28	2.8		
DMR9:58541001	9	58541001	58542000	1000	1	7.30E-06	-0.92310601	5	0.5		
DMR9:60170001	9	60170001	60171000	1000	1	5.80E-05	-0.58983093	9	0.9	Dnah7	Cytoskeleton
DMR9:61608001	9	61608001	61609000	1000	1	9.62E-06	0.93400041	9	0.9	Sf3b1	Transcription
DMR9:63403001	9	63403001	63404000	1000	1	3.39E-05	-0.65117318	8	0.8	AABR07067775.1	
DMR9:63695001	9	63695001	63696000	1000	1	4.74E-06	-0.6584772	29	2.9		
DMR9:65131001	9	65131001	65132000	1000	1	2.89E-05	0.91349326	16	1.6	Aox4;AABR07067810.2	
DMR9:65677001	9	65677001	65679000	2000	1	8.72E-06	0.43832956	17	0.85	Flacc1	
DMR9:68379001	9	68379001	68382000	3000	1	3.00E-05	0.77370775	31	1.033		
DMR9:69183001	9	69183001	69184000	1000	1	3.47E-05	1.17990009	6	0.6	Pard3b	Cell Junction
DMR9:71984001	9	71984001	71985000	1000	1	4.87E-05	0.65155524	10	1	Pikfyve	Protein Binding
DMR9:72088001	9	72088001	72089000	1000	1	5.07E-05	-0.93115426	6	0.6	Pth2r	Receptor
DMR9:74551001	9	74551001	74552000	1000	1	4.36E-05	-0.91264122	8	0.8		
DMR9:79556001	9	79556001	79557000	1000	1	3.26E-06	0.90485971	9	0.9	U2	
DMR9:79937001	9	79937001	79938000	1000	1	7.90E-05	-0.6167754	35	3.5	Smarcal1	Epigenetic
DMR9:81264001	9	81264001	81265000	1000	1	5.38E-05	-0.651099	14	1.4	Tns1	Signaling
DMR9:81544001	9	81544001	81546000	2000	1	3.68E-05	-0.55336197	31	1.55	Arpc2	
DMR9:81634001	9	81634001	81635000	1000	1	6.80E-06	-0.53329123	22	2.2	Catip;Pnkd	Development
DMR9:84012001	9	84012001	84015000	3000	2	9.38E-06	-0.74079448	63	2.1	Pax3	Transcription
DMR9:84616001	9	84616001	84617000	1000	1	7.96E-05	-0.52533198	8	0.8		
DMR9:85437001	9	85437001	85439000	2000	1	1.63E-05	-0.61287906	22	1.1	Ap1s3	Transport
DMR9:89787001	9	89787001	89788000	1000	1	4.50E-07	0.47493346	11	1.1		
DMR9:89932001	9	89932001	89933000	1000	1	2.02E-05	-0.45465388	5	0.5	AABR07068198.2	
DMR9:94563001	9	94563001	94564000	1000	1	9.90E-05	1.00284822	13	1.3	Ngef;Snorc	Transcription
DMR9:95400001	9	95400001	95402000	2000	1	1.61E-06	0.68176815	16	0.8	Trpm8	Receptor
DMR9:97935001	9	97935001	97937000	2000	1	3.95E-05	1.05883262	12	0.6	AABR07068316.1	
DMR9:98713001	9	98713001	98715000	2000	1	5.53E-05	0.51030473	18	0.9		
DMR9:99327001	9	99327001	99328000	1000	1	2.46E-05	-0.65112789	26	2.6		
DMR9:99501001	9	99501001	99502000	1000	1	3.72E-05	0.62939785	8	0.8		
DMR9:99910001	9	99910001	99911000	1000	1	6.37E-05	0.37899461	6	0.6		
DMR9:101486001	9	101486001	101487000	1000	1	1.84E-05	0.72645398	10	1		
DMR9:101767001	9	101767001	101768000	1000	1	7.40E-06	0.62539221	3	0.3		
DMR9:103427001	9	103427001	103428000	1000	1	7.20E-06	0.70646926	8	0.8		
DMR9:106406001	9	106406001	106407000	1000	1	1.41E-05	-0.45684445	6	0.6		
DMR9:107071001	9	107071001	107072000	1000	1	9.51E-05	-0.4580291	5	0.5		
DMR9:108733001	9	108733001	108734000	1000	1	3.65E-05	0.43743979	7	0.7		
DMR9:110075001	9	110075001	110076000	1000	1	2.65E-05	0.34653926	20	2	Efna5	Signaling
DMR9:110124001	9	110124001	110125000	1000	1	6.08E-05	1.06566568	21	2.1	Efna5	Signaling
DMR9:110161001	9	110161001	110163000	2000	1	1.87E-06	0.50842261	31	1.55	Efna5	Signaling
DMR9:110344001	9	110344001	110345000	1000	1	1.23E-06	0.70309062	10	1		
DMR9:110848001	9	110848001	110849000	1000	1	2.62E-06	-0.62336787	14	1.4	Fbxl17	Proteolysis
DMR9:113005001	9	113005001	113006000	1000	1	4.59E-05	-0.47239312	19	1.9	Tmem232	
DMR9:113659001	9	113659001	113660000	1000	1	2.47E-08	-0.64512204	70	7	Ankrd12	Transcription
DMR9:114401001	9	114401001	114402000	1000	1	1.36E-05	-0.54471745	27	2.7		
DMR9:114454001	9	114454001	114456000	2000	1	1.11E-05	0.81544515	33	1.65		
DMR9:115243001	9	115243001	115245000	2000	1	8.18E-05	0.70356276	22	1.1	Ptprm	Receptor
DMR9:117553001	9	117553001	117554000	1000	1	2.52E-05	-0.45784256	21	2.1	Epb41l3	Cytoskeleton
DMR9:118197001	9	118197001	118198000	1000	1	4.94E-05	-0.55715865	11	1.1		
DMR10:4024001	10	4024001	4025000	1000	1	1.75E-07	-0.86733651	25	2.5	Snx29	Cytoskeleton
DMR10:4273001	10	4273001	4275000	2000	1	1.81E-05	-0.44501591	67	3.35		
DMR10:4277001	10	4277001	4282000	5000	1	1.93E-06	-0.52173416	133	2.66		
DMR10:5749001	10	5749001	5751000	2000	1	3.99E-05	-0.81552794	36	1.8		
DMR10:10158001	10	10158001	10159000	1000	1	3.79E-07	-0.61973434	7	0.7		
DMR10:12788001	10	12788001	12791000	3000	1	2.34E-06	0.70563671	29	0.967		
DMR10:12872001	10	12872001	12873000	1000	1	8.32E-05	0.40506329	14	1.4	AABR07029195.1	
DMR10:15474001	10	15474001	15476000	2000	1	7.84E-05	0.4591187	8	0.4	Nme4;Tmem8a	Signaling
DMR10:15960001	10	15960001	15961000	1000	1	4.24E-05	-0.98337365	49	4.9	RGD1311343	
DMR10:17462001	10	17462001	17464000	2000	2	3.54E-09	1.01014192	9	0.45	Stk10	Signaling
DMR10:19685001	10	19685001	19686000	1000	1	2.29E-05	-0.66346131	21	2.1		
DMR10:20318001	10	20318001	20319000	1000	1	1.25E-05	-0.41804165	20	2	Slit3	Development
DMR10:21123001	10	21123001	21124000	1000	1	1.54E-05	0.54262432	8	0.8	Tenm2	

DMR10:21274001	10	21274001	21275000	1000	1	6.85E-06	-0.55266216	13	1.3	Tenm2	
DMR10:23597001	10	23597001	23598000	1000	1	4.84E-05	0.61224798	13	1.3		
DMR10:26090001	10	26090001	26092000	2000	1	4.74E-05	-0.46520506	46	2.3		
DMR10:28435001	10	28435001	28436000	1000	1	1.27E-06	-0.95198281	4	0.4		
DMR10:29972001	10	29972001	29973000	1000	1	7.94E-05	-0.80737762	23	2.3		
DMR10:30787001	10	30787001	30789000	2000	1	8.43E-05	0.79611778	37	1.85		
DMR10:30892001	10	30892001	30894000	2000	1	6.75E-05	-0.71599475	25	1.25	Clint1	Transport
DMR10:31392001	10	31392001	31394000	2000	1	5.88E-06	-0.64545176	89	4.45	Cyfip2	Development
DMR10:31478001	10	31478001	31479000	1000	1	1.20E-06	0.53103344	7	0.7	Itk	Signaling
DMR10:31785001	10	31785001	31787000	2000	1	5.17E-06	-0.49997123	32	1.6	Timd2	Immune
DMR10:35385001	10	35385001	35389000	4000	1	2.55E-05	0.44106494	48	1.2	Rasgef1c	Signaling
DMR10:36380001	10	36380001	36382000	2000	1	2.48E-05	-0.3587249	20	1	AC115666.1;Zfp2	Transcription
DMR10:36804001	10	36804001	36805000	1000	1	4.59E-05	0.49153677	5	0.5	LOC103693323	
DMR10:38467001	10	38467001	38468000	1000	1	2.67E-05	-0.59054994	19	1.9	Fstl4	Hormone
DMR10:40365001	10	40365001	40366000	1000	1	2.47E-05	-0.57369555	19	1.9	Anxa6;AC093965.2	Binding Protein
DMR10:40834001	10	40834001	40835000	1000	1	3.49E-05	1.02111767	5	0.5	G3bp1;Gm22884	Signaling
DMR10:41729001	10	41729001	41731000	2000	1	1.82E-06	-0.8982509	22	1.1		
DMR10:42854001	10	42854001	42855000	1000	1	1.03E-05	-0.57975316	16	1.6		
DMR10:43578001	10	43578001	43579000	1000	1	3.85E-05	-0.54305359	17	1.7	Gemin5	
DMR10:45754001	10	45754001	45755000	1000	1	4.36E-05	-0.62803961	18	1.8	Snap47	Cytoskeleton
DMR10:47276001	10	47276001	47277000	1000	1	2.58E-07	1.04828345	11	1.1	Kcnj12	Transport
DMR10:49238001	10	49238001	49240000	2000	1	2.65E-06	1.2424687	12	0.6	Trim16	Metabolism
DMR10:52322001	10	52322001	52323000	1000	1	2.29E-05	-0.50860516	47	4.7		
DMR10:54406001	10	54406001	54407000	1000	1	3.15E-06	0.57314422	12	1.2	Usp43	
DMR10:57055001	10	57055001	57056000	1000	1	5.55E-06	-0.56066245	11	1.1	Arrb2;Med11;Cxcl16;Zmynd15	Signaling;Transcription;Growth Factors & Cytokines
DMR10:59110001	10	59110001	59112000	2000	1	5.73E-05	-0.38270584	25	1.25	Spns3	Transport
DMR10:59708001	10	59708001	59709000	1000	1	1.93E-07	-0.6229197	70	7	Itgae;Haspin;AABR07072145.1	Extracellular Matrix
DMR10:60780001	10	60780001	60782000	2000	1	1.30E-05	-0.98086693	6	0.3	Olr1504	
DMR10:62047001	10	62047001	62048000	1000	1	5.26E-05	-0.49074484	25	2.5	Rtn4rl1	Receptor
DMR10:66351001	10	66351001	66352000	1000	1	5.69E-05	-0.64055138	23	2.3	Ksr1	Signaling
DMR10:67232001	10	67232001	67233000	1000	1	4.86E-05	-0.54551033	20	2		
DMR10:68565001	10	68565001	68566000	1000	1	2.00E-07	-0.38026952	0	0		
DMR10:70352001	10	70352001	70358000	6000	2	8.52E-05	0.46357288	18	0.3	Slfn13;AC128859.5;AC128859.2	Translation
DMR10:74613001	10	74613001	74614000	1000	1	4.51E-08	0.50289973	8	0.8	Ppm1e	Signaling
DMR10:75167001	10	75167001	75168000	1000	1	6.84E-05	-0.57671089	29	2.9	Mks1;Epx	Extracellular Matrix;Metabolism
DMR10:75918001	10	75918001	75919000	1000	1	2.24E-05	-0.69798967	22	2.2	Msi2	Translation
DMR10:76984001	10	76984001	76985000	1000	1	3.02E-05	-0.80390834	6	0.6		
DMR10:77759001	10	77759001	77760000	1000	1	5.37E-06	-0.45293374	21	2.1	Mmd	Development
DMR10:77926001	10	77926001	77927000	1000	1	4.56E-05	-0.59319691	30	3	Hlf	Transcription
DMR10:78135001	10	78135001	78137000	2000	1	2.66E-05	0.89990257	24	1.2	Stxbp4;Cox11	Cytoskeleton;Metabolism
DMR10:78222001	10	78222001	78223000	1000	1	1.21E-06	-0.61897765	17	1.7	Tom1l1;AABR07030257.1	Transport
DMR10:82170001	10	82170001	82172000	2000	1	1.89E-05	-0.29657512	23	1.15	Cacna1g	Transport
DMR10:83377001	10	83377001	83378000	1000	1	8.23E-05	-0.55048872	18	1.8		
DMR10:84632001	10	84632001	84633000	1000	1	1.40E-05	-0.45886076	15	1.5	Snx11	Signaling
DMR10:85352001	10	85352001	85354000	2000	1	4.42E-06	-0.5427686	26	1.3		
DMR10:87359001	10	87359001	87360000	1000	1	9.71E-05	-0.57947502	21	2.1	Krt20	Cytoskeleton
DMR10:88332001	10	88332001	88333000	1000	1	7.66E-06	-0.49666327	30	3	P3h4;Fkbp10;Nt5c3b	Signaling;Metabolism
DMR10:88380001	10	88380001	88381000	1000	1	1.99E-05	-0.66098996	15	1.5	Klhl10;Klhl11	Cytoskeleton;Transcription
DMR10:88578001	10	88578001	88579000	1000	1	1.44E-06	-0.548737	26	2.6	AABR07030473.1	
DMR10:89850001	10	89850001	89852000	2000	1	7.83E-05	-0.46663302	29	1.45		
DMR10:90599001	10	90599001	90601000	2000	1	3.16E-05	-0.59471658	39	1.95		
DMR10:91937001	10	91937001	91938000	1000	1	2.96E-05	-0.44602935	16	1.6	Nsf	Development
DMR10:92072001	10	92072001	92073000	1000	1	5.99E-06	0.39005012	6	0.6		
DMR10:93333001	10	93333001	93334000	1000	1	4.40E-06	1.09799042	11	1.1	Efcab3	
DMR10:93380001	10	93380001	93381000	1000	1	3.26E-05	0.4362612	10	1	Mettl2;U1	Metabolism
DMR10:94004001	10	94004001	94005000	1000	1	6.38E-07	0.96947739	4	0.4	Tanc2;U6	Unknown
DMR10:94552001	10	94552001	94554000	2000	1	3.58E-06	0.54674712	15	0.75	Scn4a	Transport
DMR10:94843001	10	94843001	94845000	2000	1	1.83E-06	-0.5722715	49	2.45		
DMR10:95498001	10	95498001	95499000	1000	1	7.99E-07	-0.62306947	25	2.5		
DMR10:96373001	10	96373001	96374000	1000	1	5.21E-05	-0.80128469	15	1.5	Prkca	Binding Protein
DMR10:96643001	10	96643001	96644000	1000	1	3.84E-05	0.57181527	12	1.2	Apoh	Binding Protein
DMR10:97467001	10	97467001	97468000	1000	1	6.76E-05	0.7770246	2	0.2		
DMR10:97734001	10	97734001	97735000	1000	1	3.55E-05	-0.44761965	37	3.7	Slc16a6	
DMR10:97973001	10	97973001	97975000	2000	1	2.08E-05	0.46617654	22	1.1	Fam20a	Unknown
DMR10:98174001	10	98174001	98178000	4000	1	1.14E-05	0.78438025	58	1.45	AABR07030660.1	
DMR10:98749001	10	98749001	98750000	1000	1	4.02E-06	0.47433734	8	0.8	Map2k6	Signaling
DMR10:99781001	10	99781001	99783000	2000	1	6.41E-05	0.62334463	8	0.4	AABR07030690.1	
DMR10:100263001	10	100263001	100265000	2000	1	4.32E-05	-0.60468052	5	0.25		
DMR10:103046001	10	103046001	103048000	2000	1	4.97E-05	0.47904142	27	1.35		
DMR10:103523001	10	103523001	103524000	1000	1	3.78E-05	0.82160727	6	0.6		

DMR10:103732001	10	103732001	103733000	1000	1	9.94E-05	-0.63115761	14	1.4	Slc9a3r1;Nat9;Tmem104	Metabolism
DMR10:103832001	10	103832001	103833000	1000	1	7.26E-05	0.60800182	16	1.6	Fdxr;Fads6	Metabolism
DMR10:104934001	10	104934001	104935000	1000	1	3.20E-05	0.47327165	9	0.9	RGD1562667	Immune
DMR10:105591001	10	105591001	105592000	1000	1	1.52E-07	-0.49731489	35	3.5	Rhbdf2	Protease
DMR10:105981001	10	105981001	105982000	1000	1	8.19E-05	-0.3610682	11	1.1		
DMR10:106192001	10	106192001	106193000	1000	1	8.11E-08	-0.70074805	40	4		
DMR10:106279001	10	106279001	106280000	1000	1	9.50E-05	-0.35733166	13	1.3	9-Sep	Cytoskeleton
DMR10:107077001	10	107077001	107079000	2000	1	6.81E-05	-0.46672357	66	3.3	Dnah17	Cytoskeleton
DMR10:107272001	10	107272001	107273000	1000	1	6.31E-05	-0.48172666	18	1.8	Cyth1	Signaling
DMR10:107521001	10	107521001	107523000	2000	1	4.26E-06	0.5455058	35	1.75	Engase;Rbfox3	Metabolism
DMR10:107791001	10	107791001	107792000	1000	1	4.24E-05	-0.53553072	32	3.2		
DMR10:109192001	10	109192001	109193000	1000	1	3.15E-08	1.46818297	36	3.6	Baiap2;Aatk;Mir3065	Receptor
DMR10:109800001	10	109800001	109802000	2000	1	5.26E-06	1.16191475	18	0.9	Pcyt2;Sirt7;AC131537.4;Mafg	Metabolism;Transcription
DMR11:540001	11	540001	541000	1000	1	3.24E-06	-0.63565181	6	0.6	Epha3	Receptor
DMR11:1906001	11	1906001	1907000	1000	1	7.97E-07	-1.59245988	34	3.4	Cggbp1	
DMR11:5589001	11	5589001	5590000	1000	1	3.37E-06	-0.82851478	11	1.1		
DMR11:7072001	11	7072001	7073000	1000	1	6.03E-05	-0.67244772	10	1		
DMR11:7302001	11	7302001	7304000	2000	1	6.21E-05	-0.34857621	27	1.35	Gbe1	Metabolism
DMR11:9650001	11	9650001	9651000	1000	1	2.83E-06	0.62818586	1	0.1	Robo1	Development
DMR11:10718001	11	10718001	10719000	1000	1	2.29E-05	0.98614126	8	0.8		
DMR11:13573001	11	13573001	13574000	1000	1	7.90E-05	0.51967449	7	0.7		
DMR11:14127001	11	14127001	14128000	1000	1	3.23E-05	0.87502469	8	0.8		
DMR11:17123001	11	17123001	17124000	1000	1	1.94E-05	-0.64126384	31	3.1	RGD1563888	
DMR11:18917001	11	18917001	18919000	2000	1	7.47E-05	0.49045061	3	0.15	AABR07033366.1	
DMR11:20196001	11	20196001	20197000	1000	1	4.87E-05	-0.75180933	6	0.6		
DMR11:23959001	11	23959001	23960000	1000	1	3.91E-05	0.95931005	9	0.9		
DMR11:24064001	11	24064001	24065000	1000	1	3.78E-07	1.11534025	5	0.5		
DMR11:24338001	11	24338001	24339000	1000	1	6.95E-06	0.45651107	1	0.1		
DMR11:24438001	11	24438001	24439000	1000	1	1.01E-07	-0.69565555	20	2	App	Signaling
DMR11:26611001	11	26611001	26612000	1000	1	5.59E-05	0.50161857	2	0.2		
DMR11:30726001	11	30726001	30729000	3000	2	5.39E-07	0.55821869	12	0.4		
DMR11:30819001	11	30819001	30821000	2000	1	8.21E-05	0.47840229	24	1.2		
DMR11:32348001	11	32348001	32352000	4000	2	2.25E-05	-0.81065713	35	0.875		
DMR11:32817001	11	32817001	32818000	1000	1	2.81E-05	0.43386368	21	2.1	Runx1	Transcription
DMR11:34089001	11	34089001	34091000	2000	1	9.05E-05	-0.42818926	38	1.9	Morc3	Transcription
DMR11:34456001	11	34456001	34457000	1000	1	1.92E-05	1.01921187	16	1.6	Hlcs	Metabolism
DMR11:35197001	11	35197001	35198000	1000	1	9.25E-05	-0.45868846	25	2.5		
DMR11:36238001	11	36238001	36240000	2000	1	8.20E-05	-0.76611914	16	0.8		
DMR11:36872001	11	36872001	36873000	1000	1	2.71E-05	0.48735076	0	0	Pcp4	
DMR11:37632001	11	37632001	37633000	1000	1	6.64E-05	0.56492647	7	0.7		
DMR11:37745001	11	37745001	37746000	1000	1	3.69E-05	0.51792458	34	3.4		
DMR11:38395001	11	38395001	38396000	1000	1	3.58E-06	0.89034527	5	0.5	C2cd2	
DMR11:38582001	11	38582001	38583000	1000	1	6.10E-05	-0.58543142	9	0.9	Nsun3	Transcription
DMR11:39088001	11	39088001	39093000	5000	1	4.72E-05	-0.33113968	156	3.12		
DMR11:39193001	11	39193001	39196000	3000	1	9.38E-06	-0.53543118	221	7.367	5_8S_rRNA	
DMR11:39639001	11	39639001	39642000	3000	1	5.15E-05	0.4458744	21	0.7	AABR07033829.1	
DMR11:43554001	11	43554001	43557000	3000	1	5.21E-05	0.63410026	26	0.867	Olr1553	Receptor
DMR11:44504001	11	44504001	44506000	2000	1	6.62E-06	0.67672874	25	1.25	AABR07033921.1	
DMR11:55895001	11	55895001	55896000	1000	1	5.37E-05	0.46886132	4	0.4		
DMR11:59020001	11	59020001	59021000	1000	1	9.29E-05	-0.60256391	13	1.3	Lsamp	Extracellular Matrix
DMR11:60853001	11	60853001	60854000	1000	1	3.33E-05	0.92144059	13	1.3	Cd200r1	Receptor
DMR11:64020001	11	64020001	64022000	2000	1	4.34E-05	-0.3526126	6	0.3		
DMR11:64326001	11	64326001	64328000	2000	1	2.49E-05	0.41043613	6	0.3	Igsf11	Extracellular Matrix
DMR11:67055001	11	67055001	67056000	1000	1	2.95E-05	-0.69978489	18	1.8		
DMR11:67058001	11	67058001	67059000	1000	1	1.21E-07	-0.47727032	15	1.5		
DMR11:67532001	11	67532001	67533000	1000	1	9.37E-05	-1.08157707	2	0.2	AABR07034362.2	
DMR11:69238001	11	69238001	69239000	1000	1	5.50E-05	0.82251109	9	0.9		
DMR11:69475001	11	69475001	69476000	1000	1	8.61E-06	-0.68664189	14	1.4	Kalrn	Signaling
DMR11:69805001	11	69805001	69806000	1000	1	7.52E-05	-0.68636285	19	1.9	Kalrn	Signaling
DMR11:70409001	11	70409001	70410000	1000	1	7.98E-05	0.86852628	4	0.4	Slc12a8	Transport
DMR11:71034001	11	71034001	71035000	1000	1	2.01E-07	-0.41810241	28	2.8	Iqcg;Lrch3	Unknown
DMR11:73157001	11	73157001	73159000	2000	1	7.06E-05	0.64748574	29	1.45	Acap2	Transcription
DMR11:74348001	11	74348001	74349000	1000	1	7.25E-05	-0.54650351	14	1.4		
DMR11:74573001	11	74573001	74574000	1000	1	3.43E-06	0.57325544	12	1.2		
DMR11:75171001	11	75171001	75172000	1000	1	9.17E-05	-0.4656485	17	1.7		
DMR11:77368001	11	77368001	77369000	1000	1	8.59E-05	0.76506179	3	0.3		
DMR11:77465001	11	77465001	77466000	1000	1	3.12E-05	-0.7288613	13	1.3	Il1rap	Receptor
DMR11:83338001	11	83338001	83339000	1000	1	4.11E-05	0.45239323	8	0.8		
DMR11:83631001	11	83631001	83632000	1000	1	1.10E-05	1.04783987	8	0.8		
DMR11:86316001	11	86316001	86319000	3000	2	1.33E-05	-0.37977741	21	0.7	Ufd1	
DMR11:86472001	11	86472001	86473000	1000	1	1.48E-05	0.55146578	3	0.3		
DMR11:86760001	11	86760001	86761000	1000	1	1.30E-05	-0.59398011	42	4.2	Arvcf;AC121199.1	Extracellular Matrix

DMR11:88570001	11	88570001	88571000	1000	1	4.59E-05	-0.66564559	14	1.4	LOC100912028	
DMR11:89585001	11	89585001	89587000	2000	1	7.58E-05	-0.64895516	10	0.5	Ube2v2	Proteolysis
DMR12:46001	12	46001	49000	3000	1	5.46E-05	0.36911031	21	0.7	AABR07034833.1;AABR07034833.2	
DMR12:170001	12	170001	171000	1000	1	4.32E-06	-0.44580354	27	2.7		
DMR12:328001	12	328001	330000	2000	1	9.59E-07	-0.49371703	88	4.4		
DMR12:345001	12	345001	346000	1000	1	8.86E-06	-0.5250044	63	6.3	5_8S_rRNA	
DMR12:441001	12	441001	442000	1000	1	6.88E-08	-0.44051657	49	4.9		
DMR12:851001	12	851001	852000	1000	1	3.37E-05	-0.48992049	26	2.6		
DMR12:924001	12	924001	925000	1000	1	5.08E-05	0.71878605	19	1.9		
DMR12:1372001	12	1372001	1373000	1000	1	7.82E-05	-0.63033172	8	0.8		
DMR12:3226001	12	3226001	3227000	1000	1	1.38E-05	-0.58728309	28	2.8	AABR07035008.3;AABR07035008.1;AABR07035008.2;AABR07035012.1	
DMR12:3253001	12	3253001	3254000	1000	1	7.33E-05	0.57140247	10	1	AABR07035008.3;AABR07035008.1;AABR07035008.2;AABR07035012.1	
DMR12:4282001	12	4282001	4284000	2000	1	9.88E-07	-0.51760485	74	3.7	AABR07035089.1	
DMR12:5636001	12	5636001	5637000	1000	1	7.57E-05	-0.45758111	18	1.8	Fry	Development
DMR12:5963001	12	5963001	5965000	2000	1	8.78E-05	0.57666974	45	2.25		
DMR12:7114001	12	7114001	7116000	2000	1	1.96E-05	0.52625932	21	1.05	Metazoa_SRP;AABR07035218.2	
DMR12:8161001	12	8161001	8163000	2000	1	1.14E-05	-0.6228693	29	1.45	Mtus2	Cytoskeleton
DMR12:9932001	12	9932001	9933000	1000	1	1.21E-05	-0.53727091	22	2.2		
DMR12:11894001	12	11894001	11897000	3000	1	4.67E-07	0.47754964	42	1.4		
DMR12:11916001	12	11916001	11917000	1000	1	1.08E-05	-0.51381939	16	1.6		
DMR12:12039001	12	12039001	12040000	1000	1	1.30E-06	-0.6217753	37	3.7		
DMR12:12724001	12	12724001	12725000	1000	1	6.91E-06	-0.61984429	16	1.6	Pms2;AC126486.1	DNA Repair
DMR12:13132001	12	13132001	13133000	1000	1	4.41E-06	-0.66433298	18	1.8	Daglb	
DMR12:13343001	12	13343001	13344000	1000	1	7.55E-05	-0.55811423	19	1.9	Zfp316	
DMR12:13400001	12	13400001	13402000	2000	1	4.97E-05	0.45451731	20	1	Spdye4	Cell Cycle
DMR12:14024001	12	14024001	14025000	1000	1	9.10E-09	0.62473972	9	0.9	Mmd2	Development
DMR12:15280001	12	15280001	15281000	1000	1	6.60E-05	-0.57401278	14	1.4		
DMR12:16153001	12	16153001	16154000	1000	1	8.31E-07	-0.38211687	2	0.2		
DMR12:16458001	12	16458001	16460000	2000	1	2.14E-06	-0.89968405	18	0.9	Mad1l1	
DMR12:16558001	12	16558001	16559000	1000	1	4.77E-05	1.16083876	9	0.9	Mad1l1	
DMR12:17128001	12	17128001	17129000	1000	1	3.76E-05	0.41112348	4	0.4		
DMR12:22296001	12	22296001	22297000	1000	1	9.08E-05	-0.5005404	24	2.4	AABR07035778.1;AABR07035778.2	
DMR12:24872001	12	24872001	24873000	1000	1	1.09E-05	-0.40051344	0	0		
DMR12:25500001	12	25500001	25501000	1000	1	1.72E-05	-0.56013181	28	2.8	Ncf1;Gtf2ird2	Transcription
DMR12:25636001	12	25636001	25637000	1000	1	1.65E-06	0.63240983	17	1.7	Castor2	
DMR12:25675001	12	25675001	25677000	2000	1	3.57E-06	0.49092659	15	0.75		
DMR12:26179001	12	26179001	26180000	1000	1	4.91E-05	-0.42376242	1	0.1		
DMR12:26520001	12	26520001	26521000	1000	1	2.33E-05	0.50779044	5	0.5		
DMR12:26620001	12	26620001	26622000	2000	1	1.05E-05	0.52180694	10	0.5		
DMR12:28210001	12	28210001	28211000	1000	1	4.99E-05	-0.76110626	11	1.1	Auts2	Development
DMR12:28412001	12	28412001	28414000	2000	1	6.35E-06	-0.5187938	54	2.7	Galnt17	
DMR12:29453001	12	29453001	29454000	1000	1	3.07E-05	0.50413286	6	0.6	Caln1	Signaling
DMR12:29922001	12	29922001	29923000	1000	1	6.92E-06	-1.01658927	15	1.5	Tyw1;Sbds	Translation;Transcription
DMR12:31180001	12	31180001	31182000	2000	1	7.20E-06	-0.48753085	47	2.35	Adgrd1	
DMR12:31543001	12	31543001	31544000	1000	1	1.25E-06	0.46324763	13	1.3	Rimbp2	Unknown
DMR12:37024001	12	37024001	37025000	1000	1	2.34E-05	-0.39261349	45	4.5	Ncor2;AABR07036302.1	Transcription
DMR12:37326001	12	37326001	37327000	1000	1	7.02E-05	-0.36605989	94	9.4		
DMR12:37332001	12	37332001	37333000	1000	1	6.95E-06	0.59094936	2	0.2		
DMR12:37378001	12	37378001	37380000	2000	1	2.77E-05	-0.36288142	59	2.95	Atp6v0a2	Metabolism
DMR12:38116001	12	38116001	38117000	1000	1	9.70E-05	-0.46148209	18	1.8	Denr;AABR07036318.1	Transcription
DMR12:38770001	12	38770001	38771000	1000	1	5.58E-07	-0.7980929	21	2.1	Wdr66	Unknown
DMR12:38809001	12	38809001	38810000	1000	1	1.09E-05	-0.49688146	19	1.9	Psmd9	Proteolysis
DMR12:40492001	12	40492001	40493000	1000	1	2.76E-05	-0.68687962	29	2.9	Aldh2	Metabolism
DMR12:43461001	12	43461001	43463000	2000	1	3.29E-07	-0.87040564	32	1.6	Med13l	Transcription
DMR12:44226001	12	44226001	44228000	2000	1	1.26E-05	-0.66112665	40	2	Nos1	Metabolism
DMR12:44549001	12	44549001	44550000	1000	1	6.66E-05	-0.51871321	34	3.4	Ksr2	Signaling
DMR12:44731001	12	44731001	44732000	1000	1	2.63E-05	-0.5163601	9	0.9	Ksr2	Signaling
DMR12:44800001	12	44800001	44801000	1000	1	7.72E-05	-0.81186799	19	1.9	Ksr2	Signaling
DMR12:44915001	12	44915001	44917000	2000	1	1.70E-05	0.51766682	20	1	Ksr2	Signaling
DMR12:45874001	12	45874001	45875000	1000	1	7.77E-05	-0.62336739	16	1.6	Srrm4	Translation
DMR12:46353001	12	46353001	46356000	3000	1	6.03E-05	-0.49146302	76	2.533	Cit	Signaling
DMR12:46929001	12	46929001	46930000	1000	1	1.98E-05	-0.72199204	21	2.1	Msi1	Transcription
DMR12:47570001	12	47570001	47571000	1000	1	1.01E-05	-0.54537489	39	3.9	Ankrd13a	
DMR12:48067001	12	48067001	48070000	3000	1	4.99E-05	-0.43446034	58	1.933	AABR07036556.1	
DMR12:50401001	12	50401001	50402000	1000	1	6.68E-05	0.42531703	10	1	Crybb1;Cryba4	Unknown

DMR12:50775001	12	50775001	50779000	4000	1	2.37E-05	-0.4710274	69	1.725		
DMR12:51241001	12	51241001	51242000	1000	1	3.95E-05	0.87226343	18	1.8	Mn1	Unknown
DMR12:51389001	12	51389001	51390000	1000	1	3.25E-06	-0.57197439	35	3.5	Ttc28	Unknown
DMR12:51837001	12	51837001	51838000	1000	1	5.59E-06	-0.56681229	14	1.4	AC095390.2;Chek2	Signaling
DMR13:5536001	13	5536001	5537000	1000	1	2.39E-05	-0.66901872	22	2.2		
DMR13:5765001	13	5765001	5766000	1000	1	6.73E-06	-0.72972501	6	0.6		
DMR13:8243001	13	8243001	8246000	3000	1	4.34E-05	0.78313148	25	0.833		
DMR13:8683001	13	8683001	8684000	1000	1	2.64E-05	-0.75225033	10	1		
DMR13:10360001	13	10360001	10361000	1000	1	7.93E-05	0.43436949	5	0.5		
DMR13:10942001	13	10942001	10943000	1000	1	9.33E-05	-0.43452387	19	1.9		
DMR13:12958001	13	12958001	12959000	1000	1	3.86E-05	-0.47713446	60	6		
DMR13:12984001	13	12984001	12985000	1000	1	2.79E-06	-0.5061224	34	3.4		
DMR13:13102001	13	13102001	13103000	1000	1	8.68E-05	0.66970576	7	0.7		
DMR13:16784001	13	16784001	16785000	1000	1	5.78E-06	0.57365725	11	1.1		
DMR13:18819001	13	18819001	18820000	1000	1	5.48E-05	-0.42175681	18	1.8		
DMR13:21095001	13	21095001	21096000	1000	1	5.70E-05	-0.75270661	8	0.8		
DMR13:21178001	13	21178001	21180000	2000	1	8.74E-05	-0.39262783	36	1.8		
DMR13:21242001	13	21242001	21243000	1000	1	1.80E-05	-0.41552784	35	3.5		
DMR13:24697001	13	24697001	24698000	1000	1	9.41E-05	0.87961355	7	0.7		
DMR13:24723001	13	24723001	24724000	1000	1	1.13E-05	0.98847375	5	0.5		
DMR13:26794001	13	26794001	26796000	2000	1	4.72E-05	0.97534473	30	1.5	Kdsr	Metabolism
DMR13:29135001	13	29135001	29137000	2000	1	8.72E-06	-1.33293871	3	0.15		
DMR13:30315001	13	30315001	30316000	1000	1	6.50E-05	-0.84188901	2	0.2		
DMR13:30725001	13	30725001	30726000	1000	1	1.65E-05	-0.60753904	4	0.4		
DMR13:32741001	13	32741001	32742000	1000	1	5.50E-06	-0.62444364	21	2.1		
DMR13:35663001	13	35663001	35665000	2000	1	7.07E-05	0.31914967	8	0.4	Epb41l5	
DMR13:36562001	13	36562001	36563000	1000	1	9.70E-05	0.54148324	4	0.4		
DMR13:37572001	13	37572001	37574000	2000	1	3.23E-06	-0.40026961	23	1.15		
DMR13:37585001	13	37585001	37588000	3000	1	6.10E-05	0.40223794	18	0.6		
DMR13:38418001	13	38418001	38419000	1000	1	4.26E-05	0.5716524	8	0.8		
DMR13:39059001	13	39059001	39062000	3000	1	7.99E-05	-0.54530731	67	2.233		
DMR13:41872001	13	41872001	41873000	1000	1	1.08E-05	-0.42560481	5	0.5		
DMR13:41898001	13	41898001	41899000	1000	1	2.09E-05	0.59213413	8	0.8	Slc35f5	Binding Protein
DMR13:42188001	13	42188001	42189000	1000	1	7.18E-06	0.5491583	3	0.3		
DMR13:45288001	13	45288001	45289000	1000	1	6.37E-05	-0.58066558	15	1.5		
DMR13:47636001	13	47636001	47638000	2000	1	1.11E-06	0.75922335	13	0.65	Il20	Signaling
DMR13:49119001	13	49119001	49121000	2000	1	3.48E-05	0.39602494	21	1.05	Nuak2	Signaling
DMR13:50182001	13	50182001	50183000	1000	1	1.72E-05	0.56210267	7	0.7	Lax1	
DMR13:50650001	13	50650001	50651000	1000	1	8.23E-05	-0.52348782	19	1.9		
DMR13:50913001	13	50913001	50915000	2000	1	1.09E-05	0.75351558	38	1.9	Btg2	Transcription
DMR13:51595001	13	51595001	51596000	1000	1	1.04E-05	0.52442639	7	0.7	Ppp1r12b	Signaling
DMR13:52150001	13	52150001	52152000	2000	1	2.87E-05	0.91877709	25	1.25	Lmod1	Cytoskeleton
DMR13:52699001	13	52699001	52700000	1000	1	1.08E-06	-0.62992099	7	0.7	Pkp1	
DMR13:52882001	13	52882001	52883000	1000	1	7.25E-05	0.54518304	15	1.5	Ascl5;Cacna1s	Transcription;Transport
DMR13:53329001	13	53329001	53330000	1000	1	2.43E-05	-0.60233642	20	2	Ddx59	Transcription
DMR13:55341001	13	55341001	55342000	1000	1	3.82E-05	-0.66089589	13	1.3		
DMR13:55838001	13	55838001	55839000	1000	1	6.41E-05	0.98846679	13	1.3		
DMR13:57927001	13	57927001	57929000	2000	1	8.87E-05	-0.68389961	10	0.5		
DMR13:59211001	13	59211001	59213000	2000	1	8.63E-05	0.97881065	6	0.3		
DMR13:59621001	13	59621001	59623000	2000	2	8.04E-06	0.54883808	1	0.05		
DMR13:60879001	13	60879001	60880000	1000	1	6.12E-06	-0.86581441	7	0.7		
DMR13:63627001	13	63627001	63628000	1000	1	3.11E-05	0.83605108	7	0.7	Brinp3	
DMR13:64807001	13	64807001	64809000	2000	1	8.18E-05	0.68813064	11	0.55		
DMR13:67292001	13	67292001	67293000	1000	1	6.75E-05	0.47679812	7	0.7		
DMR13:68618001	13	68618001	68619000	1000	1	1.18E-05	0.760765	10	1		
DMR13:69549001	13	69549001	69550000	1000	1	6.06E-06	-0.40621874	6	0.6		
DMR13:70084001	13	70084001	70085000	1000	1	8.42E-05	-0.3476864	16	1.6	Rgl1	Signaling
DMR13:71816001	13	71816001	71817000	1000	1	1.74E-05	0.36088138	5	0.5		
DMR13:71976001	13	71976001	71977000	1000	1	9.25E-06	1.03274922	10	1	Cacna1e	Transport
DMR13:74147001	13	74147001	74148000	1000	1	5.56E-05	0.45983453	9	0.9	Abl2	Signaling
DMR13:74255001	13	74255001	74256000	1000	1	2.33E-05	-0.64911695	14	1.4	Abl2;Tor3a	Signaling;Development
DMR13:77883001	13	77883001	77884000	1000	1	5.66E-06	0.50674222	1	0.1	Tnn;AABR07021545.1;AABR07021546.1	Extracellular Matrix
DMR13:79287001	13	79287001	79288000	1000	1	8.85E-06	0.62509944	10	1	Tnfsf4	
DMR13:79845001	13	79845001	79846000	1000	1	3.93E-05	0.57546107	7	0.7		
DMR13:80120001	13	80120001	80121000	1000	1	7.87E-06	-0.4838271	13	1.3	Dnm3;AABR07021591.1;Mir199a2;Mir3120	Cytoskeleton
DMR13:81804001	13	81804001	81807000	3000	1	4.20E-05	0.52023319	18	0.6		
DMR13:82013001	13	82013001	82014000	1000	1	5.19E-05	1.00867862	7	0.7	Mettl11b	Epigenetic
DMR13:83067001	13	83067001	83068000	1000	1	2.10E-05	-0.84204201	12	1.2	Dpt	Development
DMR13:86270001	13	86270001	86271000	1000	1	1.35E-05	0.94119653	19	1.9		
DMR13:86679001	13	86679001	86680000	1000	1	5.65E-05	0.68381224	5	0.5	AABR07021704.1	

DMR13:87106001	13	87106001	87107000	1000	1	1.89E-06	0.73806237	3	0.3		
DMR13:88267001	13	88267001	88268000	1000	1	6.62E-06	0.78554233	21	2.1	Ccdc190	
DMR13:93343001	13	93343001	93345000	2000	1	3.39E-05	0.60333867	17	0.85		
DMR13:93375001	13	93375001	93376000	1000	1	2.79E-05	0.52667589	1	0.1		
DMR13:94460001	13	94460001	94461000	1000	1	4.03E-05	1.12212487	13	1.3		
DMR13:95739001	13	95739001	95740000	1000	1	3.17E-06	0.77074175	8	0.8		
DMR13:96650001	13	96650001	96651000	1000	1	7.50E-05	0.61093321	19	1.9		
DMR13:99693001	13	99693001	99694000	1000	1	7.42E-05	-0.40313177	9	0.9	Cnih3	Signaling
DMR13:99833001	13	99833001	99834000	1000	1	5.28E-05	-0.51879493	26	2.6		
DMR13:102306001	13	102306001	102307000	1000	1	6.72E-08	0.51698614	10	1		
DMR13:102675001	13	102675001	102676000	1000	1	2.54E-06	0.48917516	5	0.5		
DMR13:103015001	13	103015001	103017000	2000	1	5.41E-05	-0.50795212	89	4.45		
DMR13:104161001	13	104161001	104162000	1000	1	5.96E-06	0.48714494	8	0.8	AABR07022046.1	
DMR13:104552001	13	104552001	104553000	1000	1	3.43E-05	0.97667088	9	0.9		
DMR13:106106001	13	106106001	106108000	2000	1	2.80E-05	1.11883994	12	0.6		
DMR13:106123001	13	106123001	106124000	1000	1	3.46E-05	-0.67366938	21	2.1		
DMR13:106427001	13	106427001	106429000	2000	1	4.82E-06	0.4148921	21	1.05		
DMR13:107184001	13	107184001	107185000	1000	1	6.49E-05	0.8503486	16	1.6	Ush2a	Extracellular Matrix
DMR13:107871001	13	107871001	107872000	1000	1	1.13E-05	0.41413824	15	1.5	Kcnk2	Transport
DMR13:108675001	13	108675001	108677000	2000	1	9.79E-05	-0.72159993	65	3.25	Smyd2	Transcription
DMR13:108762001	13	108762001	108763000	1000	1	9.79E-07	-0.45560055	25	2.5	Ptpn14	Signaling
DMR13:109409001	13	109409001	109410000	1000	1	3.16E-05	0.3824997	11	1.1	Rps6kc1	Signaling
DMR13:111286001	13	111286001	111289000	3000	1	6.77E-05	0.55583558	42	1.4	Hhat	Metabolism
DMR13:111305001	13	111305001	111306000	1000	1	7.60E-05	0.52293977	6	0.6	Hhat	Metabolism
DMR13:112131001	13	112131001	112132000	1000	1	1.17E-06	-0.85644198	13	1.3		
DMR13:112274001	13	112274001	112275000	1000	1	1.42E-05	0.44436431	6	0.6		
DMR14:2263001	14	2263001	2264000	1000	1	4.67E-05	-0.62318392	9	0.9	Pcgf3	Transcription
DMR14:3209001	14	3209001	3212000	3000	1	1.83E-05	-0.52441773	36	1.2	Rpap2;Glmn	Signaling
DMR14:3959001	14	3959001	3960000	1000	1	5.03E-05	0.84108328	14	1.4	Hfm1	Epigenetic
DMR14:4902001	14	4902001	4903000	1000	1	2.16E-05	0.63439674	6	0.6		
DMR14:4972001	14	4972001	4974000	2000	1	2.40E-05	0.46579784	9	0.45		
DMR14:4992001	14	4992001	4994000	2000	1	1.23E-05	-0.59124423	42	2.1	Zfp326	Unknown
DMR14:6741001	14	6741001	6742000	1000	1	3.07E-05	-0.54158485	11	1.1		
DMR14:7224001	14	7224001	7225000	1000	1	6.98E-08	-0.79540023	28	2.8	Klhl8;Aff1	Cytoskeleton;Transcription
DMR14:7292001	14	7292001	7294000	2000	1	9.87E-05	-0.49758807	80	4	Aff1	Transcription
DMR14:7559001	14	7559001	7560000	1000	1	4.35E-05	-0.49359634	27	2.7		
DMR14:7841001	14	7841001	7842000	1000	1	5.39E-05	0.70825868	13	1.3	Ptpn13	Signaling
DMR14:9496001	14	9496001	9497000	1000	1	1.74E-08	0.54159005	7	0.7		
DMR14:9706001	14	9706001	9707000	1000	1	6.59E-05	-0.58873642	27	2.7		
DMR14:10163001	14	10163001	10164000	1000	1	9.72E-05	0.76611535	18	1.8		
DMR14:10883001	14	10883001	10886000	3000	1	2.88E-05	-0.77389894	47	1.567	Sec31a	Transport
DMR14:10947001	14	10947001	10948000	1000	1	4.13E-06	-0.50765447	21	2.1	AABR07014350.1	
DMR14:11144001	14	11144001	11145000	1000	1	8.87E-05	-0.3139122	18	1.8	Tmem150c	Unknown
DMR14:11892001	14	11892001	11893000	1000	1	2.97E-05	0.7621723	13	1.3		
DMR14:12300001	14	12300001	12301000	1000	1	1.00E-07	-0.59175756	23	2.3	Prkg2	Signaling
DMR14:16573001	14	16573001	16574000	1000	1	2.49E-06	0.73259748	5	0.5		
DMR14:16760001	14	16760001	16761000	1000	1	2.20E-05	0.62895961	6	0.6	Shroom3	Cytoskeleton
DMR14:17067001	14	17067001	17069000	2000	1	1.82E-07	0.66977168	12	0.6	Scarb2	Receptor
DMR14:17336001	14	17336001	17338000	2000	1	9.58E-07	-0.6381009	22	1.1	Ppef2	Signaling
DMR14:17661001	14	17661001	17662000	1000	1	8.05E-05	-0.53461537	42	4.2	AC136013.3	
DMR14:17797001	14	17797001	17798000	1000	1	2.19E-05	1.14548055	8	0.8		
DMR14:20948001	14	20948001	20949000	1000	1	7.10E-06	-0.72538753	18	1.8	Dck	Signaling
DMR14:23268001	14	23268001	23269000	1000	1	2.58E-05	-0.41812287	3	0.3	Tmprss11a	Protease
DMR14:23562001	14	23562001	23563000	1000	1	5.11E-05	0.76793952	8	0.8	Uba6	Proteolysis
DMR14:23941001	14	23941001	23942000	1000	1	5.55E-05	0.55923766	5	0.5	U1	
DMR14:23977001	14	23977001	23978000	1000	1	9.97E-05	0.41516948	3	0.3	AABR07014630.1;Tmprss11f	Protease
DMR14:26520001	14	26520001	26521000	1000	1	2.09E-06	-0.46320948	45	4.5		
DMR14:26960001	14	26960001	26961000	1000	1	4.25E-06	0.40616207	2	0.2		
DMR14:28567001	14	28567001	28568000	1000	1	4.92E-08	0.75833087	9	0.9	Adgrl3	
DMR14:29310001	14	29310001	29311000	1000	1	6.46E-05	0.52253053	1	0.1		
DMR14:29587001	14	29587001	29589000	2000	1	1.97E-05	0.57349213	8	0.4		
DMR14:30983001	14	30983001	30984000	1000	1	1.82E-05	0.68087164	4	0.4		
DMR14:33576001	14	33576001	33577000	1000	1	1.33E-05	1.18915078	7	0.7	Paics;Ppat	Metabolism
DMR14:34083001	14	34083001	34084000	1000	1	1.61E-05	-0.66701693	16	1.6	Cep135	Cell Cycle
DMR14:34239001	14	34239001	34240000	1000	1	3.96E-05	-0.64292005	12	1.2		
DMR14:34245001	14	34245001	34246000	1000	1	5.45E-05	0.55724262	15	1.5		
DMR14:35775001	14	35775001	35777000	2000	1	4.66E-05	-0.67837784	43	2.15	AABR07014882.1	
DMR14:35849001	14	35849001	35850000	1000	1	5.25E-06	0.52960958	2	0.2		
DMR14:36334001	14	36334001	36336000	2000	1	5.98E-05	0.7550721	16	0.8	Scfd2	Unknown
DMR14:37226001	14	37226001	37228000	2000	1	6.46E-05	-0.43997862	42	2.1	Dcun1d4	Proteolysis
DMR14:37820001	14	37820001	37821000	1000	1	3.86E-08	-0.75972412	22	2.2	Slain2	
DMR14:39118001	14	39118001	39119000	1000	1	4.09E-05	0.47456332	4	0.4	Gabrb1	Receptor

DMR14:39374001	14	39374001	39375000	1000	1	5.39E-07	-0.65659881	19	1.9	Cox7b2	Metabolism
DMR14:40092001	14	40092001	40094000	2000	1	3.75E-06	0.76097231	3	0.15		
DMR14:40913001	14	40913001	40915000	2000	1	7.72E-05	-0.66920695	7	0.35		
DMR14:41806001	14	41806001	41807000	1000	1	4.24E-05	-0.44349867	16	1.6		
DMR14:43407001	14	43407001	43408000	1000	1	6.39E-05	0.85439975	11	1.1		
DMR14:44564001	14	44564001	44565000	1000	1	5.58E-05	-0.50968707	22	2.2	AABR07015003.1;AABR07015002.1	
DMR14:45094001	14	45094001	45095000	1000	1	5.39E-05	0.50170816	11	1.1		
DMR14:54147001	14	54147001	54148000	1000	1	3.04E-05	0.40269985	13	1.3		
DMR14:57060001	14	57060001	57061000	1000	1	3.72E-05	0.89705615	3	0.3		
DMR14:59576001	14	59576001	59578000	2000	1	4.40E-08	0.32889526	7	0.35		
DMR14:66705001	14	66705001	66706000	1000	1	8.54E-05	-0.37113302	30	3	Kcnip4	Metabolism
DMR14:68932001	14	68932001	68933000	1000	1	3.80E-05	-0.80729978	13	1.3		
DMR14:69809001	14	69809001	69811000	2000	1	7.13E-06	-0.52151295	23	1.15	Lcorl	Transcription
DMR14:70338001	14	70338001	70340000	2000	1	1.32E-05	-0.6408416	19	0.95		
DMR14:73576001	14	73576001	73577000	1000	1	3.31E-05	0.72580791	9	0.9		
DMR14:77920001	14	77920001	77921000	1000	1	2.34E-05	0.57274685	7	0.7	Stk32b	Signaling
DMR14:78384001	14	78384001	78385000	1000	1	2.54E-05	-0.40966722	6	0.6	AABR07015812.1;AABR07015812.2	
DMR14:78663001	14	78663001	78664000	1000	1	1.20E-06	-0.55532819	38	3.8	Wfs1	Development
DMR14:79009001	14	79009001	79011000	2000	1	2.18E-07	-0.48489988	76	3.8	Bloc1s4	
DMR14:80085001	14	80085001	80086000	1000	1	2.73E-05	0.44366852	6	0.6	Ablim2	Cytoskeleton
DMR14:81013001	14	81013001	81014000	1000	1	8.72E-05	0.64854576	12	1.2	Rgs12	
DMR14:82510001	14	82510001	82511000	1000	1	8.62E-05	-0.53788808	31	3.1		
DMR14:84570001	14	84570001	84571000	1000	1	6.74E-05	-0.47592786	24	2.4	U4;Hormad2	Transcription
DMR14:85743001	14	85743001	85744000	1000	1	5.42E-05	-0.56891443	20	2	Xbp1	Transcription
DMR14:86361001	14	86361001	86363000	2000	1	9.38E-07	0.47954317	19	0.95	Npc1l1;U4	Receptor
DMR14:87025001	14	87025001	87027000	2000	1	9.44E-05	0.45554992	21	1.05		
DMR14:87092001	14	87092001	87093000	1000	1	8.83E-05	-0.49103241	24	2.4		
DMR14:87324001	14	87324001	87327000	3000	1	9.89E-07	0.59888912	32	1.067	Adcy1	Signaling
DMR14:89017001	14	89017001	89018000	1000	1	7.53E-06	0.51289634	5	0.5	5S_rRNA	
DMR14:91563001	14	91563001	91564000	1000	1	3.47E-06	0.69561285	3	0.3	Zpbp;Spata48	Development
DMR14:94161001	14	94161001	94163000	2000	1	8.43E-05	-0.63553755	7	0.35		
DMR14:95333001	14	95333001	95334000	1000	1	6.92E-05	-0.30151673	11	1.1		
DMR14:95682001	14	95682001	95684000	2000	1	7.28E-05	0.37846478	33	1.65		
DMR14:95687001	14	95687001	95688000	1000	1	3.27E-05	0.52600841	24	2.4		
DMR14:96478001	14	96478001	96479000	1000	1	3.84E-05	-0.90833704	6	0.6		
DMR14:98515001	14	98515001	98516000	1000	1	4.03E-06	0.46364653	4	0.4		
DMR14:101413001	14	101413001	101414000	1000	1	2.74E-05	0.36672027	7	0.7		
DMR14:102184001	14	102184001	102186000	2000	1	1.26E-06	-0.69822589	20	1		
DMR14:102338001	14	102338001	102340000	2000	1	2.13E-05	-0.41336772	47	2.35		
DMR14:102591001	14	102591001	102592000	1000	1	7.24E-05	0.78497522	10	1		
DMR14:103352001	14	103352001	103353000	1000	1	9.99E-05	0.4631964	6	0.6	AABR07016556.1	
DMR14:104285001	14	104285001	104287000	2000	1	3.70E-06	-0.50486845	29	1.45	Spred2	Signaling
DMR14:104336001	14	104336001	104337000	1000	1	7.35E-05	-0.50924272	27	2.7	Actr2	Cytoskeleton
DMR14:104985001	14	104985001	104986000	1000	1	1.04E-05	0.68077017	7	0.7	LOC108352861	
DMR14:106323001	14	106323001	106324000	1000	1	1.14E-05	-0.5744004	29	2.9		
DMR14:107085001	14	107085001	107086000	1000	1	2.39E-05	-0.51108967	26	2.6	Ehbp1	Unknown
DMR14:108307001	14	108307001	108308000	1000	1	6.65E-05	-0.57766646	22	2.2	RGD1305110	
DMR14:109607001	14	109607001	109610000	3000	1	4.69E-08	-0.73986766	49	1.633		
DMR15:1548001	15	1548001	1549000	1000	1	9.83E-05	-0.84376209	2	0.2	AABR07016845.1	
DMR15:2087001	15	2087001	2088000	1000	1	9.68E-05	0.6388274	6	0.6		
DMR15:2917001	15	2917001	2918000	1000	1	7.99E-06	-0.60784807	24	2.4	Kat6b	Epigenetic
DMR15:3154001	15	3154001	3155000	1000	1	5.66E-05	-0.47461848	4	0.4	Adk	Signaling
DMR15:3317001	15	3317001	3318000	1000	1	4.83E-07	-0.73192824	16	1.6	Adk	Signaling
DMR15:4025001	15	4025001	4026000	1000	1	1.12E-05	0.54458135	7	0.7	Zswim8;Chchd1;U6;Fut11;Sec24c	Transcription;Metabolism;Transport
DMR15:4296001	15	4296001	4298000	2000	1	5.40E-05	-0.62063398	33	1.65	Cfap70;AC115418.1	
DMR15:5818001	15	5818001	5819000	1000	1	7.91E-05	0.48708482	8	0.8	LOC108348081;LOC102550314	
DMR15:6604001	15	6604001	6605000	1000	1	7.25E-05	-0.85941719	4	0.4		
DMR15:8564001	15	8564001	8565000	1000	1	6.02E-06	0.414368	1	0.1		
DMR15:8967001	15	8967001	8968000	1000	1	5.00E-06	0.43253746	7	0.7	Thrb	
DMR15:8969001	15	8969001	8970000	1000	1	9.19E-06	0.43584285	2	0.2	Thrb	
DMR15:9057001	15	9057001	9058000	1000	1	8.39E-07	-0.84534075	3	0.3	Thrb	
DMR15:9884001	15	9884001	9885000	1000	1	2.81E-06	0.49264879	1	0.1		
DMR15:10236001	15	10236001	10238000	2000	1	6.39E-05	0.81494225	13	0.65	Rarb	Signaling
DMR15:12632001	15	12632001	12633000	1000	1	2.74E-05	-0.51098364	7	0.7		
DMR15:13281001	15	13281001	13282000	1000	1	6.04E-06	0.56438312	10	1		
DMR15:15510001	15	15510001	15511000	1000	1	2.10E-05	0.4700327	2	0.2	Cadps	Metabolism
DMR15:16337001	15	16337001	16338000	1000	1	5.28E-05	0.50187135	7	0.7		
DMR15:17405001	15	17405001	17406000	1000	1	8.01E-05	-0.39924064	36	3.6		
DMR15:18500001	15	18500001	18501000	1000	1	6.72E-05	0.37981425	10	1	Kctd6	Transport

DMR15:19384001	15	19384001	19386000	2000	1	7.58E-05	0.42397158	15	0.75		
DMR15:20947001	15	20947001	20948000	1000	1	5.13E-05	0.62156765	5	0.5		
DMR15:21035001	15	21035001	21036000	1000	1	4.06E-06	-0.33484673	9	0.9		
DMR15:21127001	15	21127001	21128000	1000	1	5.13E-05	-0.44269603	4	0.4		
DMR15:21525001	15	21525001	21526000	1000	1	5.44E-05	-0.33598615	3	0.3		
DMR15:22945001	15	22945001	22947000	2000	1	6.53E-05	0.43774171	16	0.8		
DMR15:23920001	15	23920001	23921000	1000	1	2.82E-05	-0.7386368	21	2.1		
DMR15:24136001	15	24136001	24138000	2000	1	5.37E-06	-0.62560753	20	1	Lgals3	Cytoskeleton
DMR15:24631001	15	24631001	24632000	1000	1	7.37E-05	0.55844844	10	1		
DMR15:24872001	15	24872001	24873000	1000	1	3.31E-06	0.55316786	11	1.1	AABR07017513.1	
DMR15:25357001	15	25357001	25358000	1000	1	4.91E-06	0.89013661	10	1	Tmem260	
DMR15:31300001	15	31300001	31301000	1000	1	2.84E-05	-0.65647701	2	0.2	AABR07017814.1	
DMR15:32817001	15	32817001	32818000	1000	1	8.97E-05	0.41845732	10	1	AABR07017902.1	
DMR15:32866001	15	32866001	32867000	1000	1	9.54E-05	0.8622798	13	1.3	AABR07017902.1;Dad1	Metabolism
DMR15:36680001	15	36680001	36681000	1000	1	1.34E-05	0.52104768	12	1.2	Rnf17	
DMR15:36825001	15	36825001	36827000	2000	1	1.32E-07	0.46541903	30	1.5	AABR07018038.1	
DMR15:36938001	15	36938001	36939000	1000	1	4.06E-05	-0.46893076	21	2.1	AABR07018038.3;Mphosph8	
DMR15:37641001	15	37641001	37642000	1000	1	1.67E-05	-0.41196223	19	1.9	Cryl1	Metabolism
DMR15:37857001	15	37857001	37858000	1000	1	8.77E-05	0.66607231	11	1.1	Xpo4	Metabolism
DMR15:38283001	15	38283001	38286000	3000	1	7.09E-05	0.9551747	32	1.067	Micu2	
DMR15:38491001	15	38491001	38492000	1000	1	7.50E-05	0.77904867	6	0.6		
DMR15:40447001	15	40447001	40448000	1000	1	2.77E-05	-0.56111145	14	1.4		
DMR15:41336001	15	41336001	41337000	1000	1	9.24E-06	-0.5269859	8	0.8	Tnfrsf19	Receptor
DMR15:41481001	15	41481001	41483000	2000	1	1.83E-06	0.36645809	2	0.1	Sacs	
DMR15:41484001	15	41484001	41485000	1000	1	2.59E-05	0.43263061	2	0.2	Sacs	
DMR15:42406001	15	42406001	42407000	1000	1	3.23E-05	-0.39321655	11	1.1		
DMR15:43529001	15	43529001	43531000	2000	2	2.58E-07	0.43797885	9	0.45	Dpysl2	Metabolism
DMR15:45006001	15	45006001	45007000	1000	1	1.10E-06	0.91497163	8	0.8		
DMR15:45732001	15	45732001	45733000	1000	1	5.87E-06	0.87258161	23	2.3	Fam124a	
DMR15:47645001	15	47645001	47646000	1000	1	3.36E-05	0.79253268	8	0.8	Msra	Metabolism
DMR15:48016001	15	48016001	48017000	1000	1	4.84E-05	0.354983	2	0.2		
DMR15:49890001	15	49890001	49891000	1000	1	8.67E-05	-0.60580652	15	1.5		
DMR15:55228001	15	55228001	55230000	2000	1	2.45E-05	-0.44193785	14	0.7		
DMR15:56376001	15	56376001	56377000	1000	1	1.27E-05	-0.9502583	4	0.4		
DMR15:57729001	15	57729001	57731000	2000	1	4.45E-05	0.37192205	15	0.75		
DMR15:57862001	15	57862001	57863000	1000	1	9.45E-06	-0.47938496	14	1.4	AABR07018438.1	
DMR15:58218001	15	58218001	58219000	1000	1	7.69E-05	-0.38329409	44	4.4		
DMR15:59745001	15	59745001	59747000	2000	1	1.87E-05	-0.4961967	47	2.35	Enox1	Transcription
DMR15:60690001	15	60690001	60691000	1000	1	6.08E-05	-0.82361551	14	1.4	AABR07018476.1	
DMR15:61279001	15	61279001	61281000	2000	1	8.41E-05	1.00230212	32	1.6		
DMR15:61714001	15	61714001	61715000	1000	1	6.88E-05	0.7701387	2	0.2	AABR07018496.1	
DMR15:62937001	15	62937001	62938000	1000	1	3.21E-05	0.36107512	2	0.2		
DMR15:65172001	15	65172001	65176000	4000	1	1.15E-05	0.5624972	29	0.725		
DMR15:75464001	15	75464001	75465000	1000	1	9.05E-06	-0.65622915	30	3		
DMR15:76246001	15	76246001	76247000	1000	1	2.88E-05	-0.7342845	6	0.6		
DMR15:78230001	15	78230001	78232000	2000	1	3.73E-05	0.57557129	16	0.8		
DMR15:78447001	15	78447001	78448000	1000	1	7.10E-05	-0.75317552	10	1		
DMR15:78735001	15	78735001	78737000	2000	1	5.80E-08	0.80180684	12	0.6		
DMR15:79433001	15	79433001	79434000	1000	1	5.20E-06	-0.70123981	6	0.6		
DMR15:83064001	15	83064001	83065000	1000	1	6.56E-06	0.86293285	9	0.9		
DMR15:84329001	15	84329001	84331000	2000	1	1.13E-05	-0.6074642	19	0.95	Klf12	Transcription
DMR15:85214001	15	85214001	85215000	1000	1	1.96E-06	-0.52343971	15	1.5		
DMR15:86368001	15	86368001	86369000	1000	1	3.94E-06	0.53108619	7	0.7	Lmo7	Cytoskeleton
DMR15:86554001	15	86554001	86555000	1000	1	1.82E-05	-0.44337735	0	0		
DMR15:87371001	15	87371001	87373000	2000	1	4.59E-05	-0.73384144	23	1.15		
DMR15:89527001	15	89527001	89528000	1000	1	1.96E-05	0.40709546	5	0.5	Mycbp2	Metabolism
DMR15:89735001	15	89735001	89737000	2000	1	7.09E-05	-0.66279533	28	1.4	Mycbp2	Metabolism
DMR15:93321001	15	93321001	93322000	1000	1	9.56E-05	-0.85097824	3	0.3	Mycbp2	Metabolism
DMR15:95496001	15	95496001	95497000	1000	1	1.99E-06	0.55865939	1	0.1		
DMR15:98762001	15	98762001	98763000	1000	1	1.56E-05	-1.02720964	4	0.4		
DMR15:100010001	15	100010001	100011000	1000	1	3.16E-06	0.93375559	5	0.5		
DMR15:102778001	15	102778001	102779000	1000	1	6.29E-05	0.96186482	11	1.1	Gpc6	Extracellular Matrix
DMR15:102988001	15	102988001	102989000	1000	1	4.84E-05	0.57431453	6	0.6	Gpc6	Extracellular Matrix
DMR15:103783001	15	103783001	103785000	2000	1	9.14E-05	-0.45327578	54	2.7	Abcc4	Receptor
DMR15:104050001	15	104050001	104051000	1000	1	6.48E-05	0.4027323	11	1.1	Cldn10	Cell Junction
DMR15:104824001	15	104824001	104825000	1000	1	5.65E-05	-0.48386465	25	2.5	Hs6st3	Metabolism
DMR15:104858001	15	104858001	104860000	2000	1	1.16E-05	0.54404276	18	0.9	Hs6st3	Metabolism
DMR15:105293001	15	105293001	105294000	1000	1	3.74E-05	-0.52250873	25	2.5		
DMR15:105437001	15	105437001	105440000	3000	1	7.17E-05	0.47382362	19	0.633		
DMR15:106482001	15	106482001	106483000	1000	1	9.46E-05	-0.54666625	25	2.5	Farp1	Signaling
DMR15:107547001	15	107547001	107549000	2000	1	9.09E-05	-0.34628033	23	1.15		
DMR15:108030001	15	108030001	108032000	2000	1	7.78E-06	0.48304242	28	1.4	Dock9	Cell Cycle

DMR15:108138001	15	108138001	108139000	1000	1	2.49E-05	-0.58284832	7	0.7	Dock9	Cell Cycle
DMR15:110791001	15	110791001	110792000	1000	1	5.72E-07	-0.77033254	2	0.2		
DMR16:2185001	16	2185001	2187000	2000	1	2.75E-05	0.38618466	11	0.55	Slmap;SNORA71	Protein Binding
DMR16:2655001	16	2655001	2656000	1000	1	7.85E-05	0.70039699	14	1.4		
DMR16:3399001	16	3399001	3401000	2000	1	5.01E-06	0.65821313	6	0.3	Erc2	Cytoskeleton
DMR16:3897001	16	3897001	3898000	1000	1	4.32E-05	0.36076116	6	0.6	Anxa11	Signaling
DMR16:4306001	16	4306001	4307000	1000	1	8.01E-05	-0.68520635	16	1.6		
DMR16:5580001	16	5580001	5581000	1000	1	3.27E-05	0.67296233	11	1.1	Cacna2d3	Transport
DMR16:5853001	16	5853001	5854000	1000	1	1.96E-05	0.45897386	12	1.2		
DMR16:7240001	16	7240001	7241000	1000	1	2.54E-08	-0.68360577	34	3.4	Stab1	Extracellular Matrix
DMR16:7265001	16	7265001	7266000	1000	1	1.23E-05	-0.6715266	24	2.4	Nisch	Signaling
DMR16:8044001	16	8044001	8046000	2000	1	1.21E-05	-0.59882913	39	1.95		
DMR16:10549001	16	10549001	10551000	2000	1	1.08E-07	0.54653025	18	0.9	Syt15	Transport
DMR16:16166001	16	16166001	16167000	1000	1	3.99E-05	0.5351858	7	0.7		
DMR16:17640001	16	17640001	17641000	1000	1	3.09E-05	-0.6505695	22	2.2	Sh2d4b	
DMR16:17679001	16	17679001	17681000	2000	2	3.55E-05	0.60624575	13	0.65	Sh2d4b	
DMR16:18613001	16	18613001	18614000	1000	1	8.40E-05	0.8477284	8	0.8		
DMR16:19138001	16	19138001	19139000	1000	1	1.10E-05	0.78426183	7	0.7	Eps15l1	Signaling
DMR16:20896001	16	20896001	20899000	3000	1	9.30E-05	-0.27626102	3	0.1	Homer3	Signaling
DMR16:21787001	16	21787001	21788000	1000	1	3.23E-05	0.31221793	6	0.6		
DMR16:21869001	16	21869001	21873000	4000	1	1.28E-06	0.69300423	61	1.525		
DMR16:21905001	16	21905001	21907000	2000	1	1.79E-05	0.46647968	21	1.05		
DMR16:21919001	16	21919001	21922000	3000	1	8.05E-05	0.34742343	17	0.567		
DMR16:22022001	16	22022001	22024000	2000	1	7.75E-07	-0.28608555	33	1.65	Potem	
DMR16:22054001	16	22054001	22057000	3000	1	1.55E-05	0.2478184	42	1.4	Potem	
DMR16:22064001	16	22064001	22065000	1000	1	7.75E-05	0.41226483	11	1.1	Potem	
DMR16:23456001	16	23456001	23457000	1000	1	2.40E-05	0.870859	7	0.7	Psd3	Signaling
DMR16:24266001	16	24266001	24268000	2000	1	8.49E-05	0.50054802	33	1.65		
DMR16:24401001	16	24401001	24402000	1000	1	4.75E-05	0.89506885	7	0.7		
DMR16:31815001	16	31815001	31818000	3000	1	8.00E-05	-0.68566673	47	1.567	AABR07025295.1	
DMR16:31822001	16	31822001	31825000	3000	1	7.54E-05	0.89296047	28	0.933	AABR07025295.1	
DMR16:32126001	16	32126001	32127000	1000	1	4.24E-05	-0.53583091	26	2.6	Sh3rf1	
DMR16:34457001	16	34457001	34458000	1000	1	1.17E-05	-0.82877464	4	0.4		
DMR16:36484001	16	36484001	36486000	2000	1	6.49E-06	-0.93831668	21	1.05		
DMR16:39204001	16	39204001	39206000	2000	1	2.09E-05	0.51116856	16	0.8		
DMR16:41831001	16	41831001	41832000	1000	1	4.79E-06	-0.51585401	26	2.6		
DMR16:42370001	16	42370001	42372000	2000	1	9.06E-05	-0.58256004	14	0.7		
DMR16:44825001	16	44825001	44826000	1000	1	8.44E-06	0.56545546	2	0.2		
DMR16:45514001	16	45514001	45518000	4000	2	1.46E-08	0.89461068	44	1.1		
DMR16:45695001	16	45695001	45696000	1000	1	1.17E-05	0.57453423	2	0.2		
DMR16:45829001	16	45829001	45830000	1000	1	4.21E-05	-0.66610734	28	2.8		
DMR16:46746001	16	46746001	46748000	2000	1	3.28E-07	0.57892626	13	0.65	Tenm3	
DMR16:47484001	16	47484001	47486000	2000	1	8.04E-06	-0.57174856	39	1.95	Wwc2	Unknown
DMR16:48325001	16	48325001	48326000	1000	1	4.23E-06	0.49363873	5	0.5	Enpp6	Signaling
DMR16:49858001	16	49858001	49859000	1000	1	2.41E-07	0.65880813	3	0.3		
DMR16:50753001	16	50753001	50755000	2000	1	6.70E-06	0.70759186	8	0.4		
DMR16:54777001	16	54777001	54779000	2000	1	3.60E-05	-0.87857306	17	0.85	Mtmr7	Signaling
DMR16:59811001	16	59811001	59813000	2000	1	5.09E-06	0.5870766	12	0.6		
DMR16:61989001	16	61989001	61990000	1000	1	1.86E-05	0.58118542	2	0.2	Rbpms	Transcription
DMR16:62332001	16	62332001	62333000	1000	1	3.50E-06	-0.41569042	16	1.6	Tex15	
DMR16:63888001	16	63888001	63889000	1000	1	1.60E-05	0.67681815	15	1.5	Nrg1	Signaling
DMR16:68942001	16	68942001	68943000	1000	1	9.10E-05	0.43410853	14	1.4	AC126482.1	
DMR16:70627001	16	70627001	70628000	1000	1	4.90E-06	-0.710024	18	1.8		
DMR16:71004001	16	71004001	71005000	1000	1	6.17E-05	0.66540568	15	1.5	Kcnu1;Ash2l	Transcription
DMR16:71936001	16	71936001	71937000	1000	1	2.13E-05	-0.82602269	4	0.4	Adam32	Protease
DMR16:72104001	16	72104001	72106000	2000	1	4.31E-05	-0.61690659	44	2.2	Adam3a	
DMR16:72208001	16	72208001	72210000	2000	1	7.97E-05	-0.98888449	19	0.95	Adam18;Ido1	Protease;Signaling
DMR16:72486001	16	72486001	72488000	2000	1	5.49E-05	0.90452482	11	0.55		
DMR16:72552001	16	72552001	72553000	1000	1	1.70E-06	1.23392288	16	1.6		
DMR16:73644001	16	73644001	73645000	1000	1	9.58E-05	0.78339597	9	0.9	Gpat4	
DMR16:75677001	16	75677001	75679000	2000	1	2.57E-05	-0.87452562	13	0.65	Spag11a	Immune
DMR16:80533001	16	80533001	80534000	1000	1	1.89E-06	0.58420049	8	0.8		
DMR16:80628001	16	80628001	80630000	2000	1	4.65E-05	-0.49410124	49	2.45	Erich1	Unknown
DMR16:81213001	16	81213001	81214000	1000	1	8.81E-05	-0.60115749	26	2.6	Tmem255b;Gas6	Signaling
DMR16:82749001	16	82749001	82750000	1000	1	9.29E-07	0.50734788	4	0.4		
DMR16:83269001	16	83269001	83272000	3000	1	8.05E-05	1.08902216	53	1.767	Ing1	Transcription
DMR16:83717001	16	83717001	83718000	1000	1	1.80E-07	-1.15064095	12	1.2		
DMR16:85182001	16	85182001	85183000	1000	1	4.15E-05	0.55618036	15	1.5		
DMR16:86942001	16	86942001	86944000	2000	1	4.58E-05	-0.54729556	18	0.9		
DMR16:87511001	16	87511001	87512000	1000	1	1.44E-05	0.70480488	5	0.5	AABR07026641.1	
DMR17:14001	17	14001	15000	1000	1	5.20E-05	-0.44418136	35	3.5		
DMR17:594001	17	594001	596000	2000	1	4.98E-06	-0.75576068	6	0.3	Npepo	Proteolysis

DMR17:4665001	17	4665001	4666000	1000	1	3.49E-07	0.50061571	9	0.9		
DMR17:7465001	17	7465001	7467000	2000	1	6.39E-05	-0.58677451	54	2.7		
DMR17:8844001	17	8844001	8845000	1000	1	8.10E-05	0.43931613	5	0.5		
DMR17:9667001	17	9667001	9669000	2000	1	1.46E-05	-0.5426667	43	2.15	Pdlim7;AC121413.2	Receptor
DMR17:10300001	17	10300001	10301000	1000	1	7.65E-06	0.43714869	6	0.6		
DMR17:11762001	17	11762001	11763000	1000	1	5.20E-05	0.48114502	13	1.3		
DMR17:12216001	17	12216001	12217000	1000	1	2.37E-05	0.37846288	13	1.3		
DMR17:12332001	17	12332001	12333000	1000	1	1.46E-06	-0.53006695	23	2.3	Auh	Metabolism
DMR17:12511001	17	12511001	12513000	2000	1	3.38E-05	0.39414528	7	0.35		
DMR17:13424001	17	13424001	13427000	3000	1	2.74E-05	-0.55109654	50	1.667	AABR07027080.1	
DMR17:14141001	17	14141001	14142000	1000	1	2.66E-05	-0.38737854	38	3.8		
DMR17:14645001	17	14645001	14646000	1000	1	8.68E-05	-0.68679588	9	0.9	Omd	Cytoskeleton
DMR17:16068001	17	16068001	16069000	1000	1	4.24E-05	-0.6119119	19	1.9		
DMR17:16251001	17	16251001	16253000	2000	1	5.58E-07	-0.58213996	48	2.4	Phf2	
DMR17:16271001	17	16271001	16272000	1000	1	8.46E-05	-0.56708887	37	3.7	Phf2	
DMR17:16551001	17	16551001	16552000	1000	1	5.59E-05	-0.50872239	25	2.5		
DMR17:16554001	17	16554001	16555000	1000	1	9.88E-05	-0.49938511	27	2.7		
DMR17:16558001	17	16558001	16559000	1000	1	5.31E-05	-0.54636603	11	1.1		
DMR17:17375001	17	17375001	17377000	2000	1	4.20E-05	-0.61011166	36	1.8		
DMR17:17691001	17	17691001	17692000	1000	1	3.51E-05	0.93806599	10	1		
DMR17:18126001	17	18126001	18128000	2000	1	2.50E-05	0.43530687	26	1.3		
DMR17:18131001	17	18131001	18132000	1000	1	1.72E-05	0.53633979	5	0.5		
DMR17:18166001	17	18166001	18169000	3000	1	6.64E-05	-0.43908396	66	2.2	Kif13a	Cytoskeleton
DMR17:18783001	17	18783001	18785000	2000	1	4.16E-05	0.95284612	27	1.35		
DMR17:18945001	17	18945001	18946000	1000	1	2.69E-05	-0.45876546	3	0.3		
DMR17:19102001	17	19102001	19105000	3000	1	1.15E-05	-0.52792662	45	1.5		
DMR17:19408001	17	19408001	19409000	1000	1	2.25E-05	-0.52604665	24	2.4	Atxn1	Transcription
DMR17:19523001	17	19523001	19527000	4000	1	1.03E-06	0.32698708	63	1.575	Atxn1	Transcription
DMR17:19627001	17	19627001	19630000	3000	1	4.46E-05	0.7529407	44	1.467		
DMR17:20571001	17	20571001	20573000	2000	1	8.90E-05	0.78214003	36	1.8		
DMR17:22727001	17	22727001	22729000	2000	1	6.32E-05	-0.58764298	9	0.45		
DMR17:22905001	17	22905001	22908000	3000	1	1.40E-05	0.4843201	27	0.9		
DMR17:23852001	17	23852001	23853000	1000	1	1.36E-05	-0.79567077	22	2.2	Gfod1	Metabolism
DMR17:28339001	17	28339001	28340000	1000	1	7.50E-05	1.0021759	6	0.6		
DMR17:28638001	17	28638001	28639000	1000	1	3.67E-07	-0.86112194	12	1.2	F13a1	Immune
DMR17:28673001	17	28673001	28674000	1000	1	6.45E-05	-0.8069947	18	1.8	F13a1	Immune
DMR17:29837001	17	29837001	29839000	2000	1	1.10E-05	0.45365487	6	0.3	Cdyl	Metabolism
DMR17:31524001	17	31524001	31525000	1000	1	4.05E-05	-0.95362758	12	1.2	Bphl	Metabolism
DMR17:34117001	17	34117001	34119000	2000	1	3.05E-05	0.79305596	24	1.2		
DMR17:34409001	17	34409001	34410000	1000	1	7.45E-05	0.38649366	4	0.4		
DMR17:34825001	17	34825001	34826000	1000	1	3.90E-05	1.20170513	7	0.7	Exoc2	
DMR17:35258001	17	35258001	35259000	1000	1	2.33E-06	-0.63067974	9	0.9		
DMR17:35339001	17	35339001	35340000	1000	1	1.09E-05	-0.84223979	10	1		
DMR17:36632001	17	36632001	36633000	1000	1	1.70E-05	0.63570925	12	1.2	Cdkal1	Cell Cycle
DMR17:37249001	17	37249001	37250000	1000	1	1.89E-05	0.60408602	8	0.8	Cdkal1;AABR07027569.3	Cell Cycle
DMR17:40205001	17	40205001	40207000	2000	1	9.08E-06	0.78742871	16	0.8		
DMR17:43859001	17	43859001	43860000	1000	1	4.11E-05	-0.52431075	4	0.4	Btn2a2	
DMR17:43939001	17	43939001	43940000	1000	1	2.63E-05	-0.85165229	8	0.8	AABR07027758.1;Abt1	
DMR17:49658001	17	49658001	49659000	1000	1	3.26E-08	-0.42827657	46	4.6		
DMR17:52835001	17	52835001	52836000	1000	1	6.91E-05	-0.79259192	13	1.3		
DMR17:53610001	17	53610001	53611000	1000	1	2.61E-05	0.77635558	14	1.4	Hecw1	Protease
DMR17:60529001	17	60529001	60532000	3000	2	4.01E-05	0.64532808	32	1.067	Mkx	Epigenetic
DMR17:62501001	17	62501001	62502000	1000	1	2.39E-05	-0.84323704	1	0.1		
DMR17:70470001	17	70470001	70472000	2000	1	1.09E-05	0.52698069	26	1.3	Il15ra	Immune
DMR17:70842001	17	70842001	70843000	1000	1	8.02E-05	0.61774973	17	1.7		
DMR17:70967001	17	70967001	70970000	3000	1	5.91E-06	0.75556875	31	1.033	Prkcq	Binding Protein
DMR17:71316001	17	71316001	71319000	3000	1	2.62E-05	0.47817992	11	0.367	Gm23877	
DMR17:72832001	17	72832001	72833000	1000	1	3.12E-05	0.55304019	5	0.5		
DMR17:76675001	17	76675001	76676000	1000	1	1.52E-05	-0.66232802	21	2.1	Camk1d	Signaling
DMR17:77766001	17	77766001	77769000	3000	1	1.90E-05	-0.56552203	66	2.2		
DMR17:77939001	17	77939001	77940000	1000	1	3.65E-05	0.5285331	4	0.4		
DMR17:78801001	17	78801001	78802000	1000	1	2.38E-05	-0.53286546	19	1.9	Dclre1c;Mt1	
DMR17:79012001	17	79012001	79013000	1000	1	1.95E-05	0.48542208	8	0.8		
DMR17:81349001	17	81349001	81352000	3000	1	2.72E-05	0.48857878	30	1	Tmem236;Mrc1	Receptor
DMR17:82833001	17	82833001	82834000	1000	1	7.95E-06	0.58327785	5	0.5	Malrd1	
DMR17:84270001	17	84270001	84271000	1000	1	6.74E-06	-0.67105467	16	1.6	Nebl	Cytoskeleton
DMR17:85240001	17	85240001	85241000	1000	1	1.29E-05	1.05283022	8	0.8		
DMR17:85554001	17	85554001	85555000	1000	1	3.94E-05	-0.52784385	25	2.5	AABR07028769.1	
DMR17:85872001	17	85872001	85874000	2000	1	8.04E-06	0.88869147	22	1.1		
DMR17:86755001	17	86755001	86757000	2000	1	6.57E-05	0.48191333	11	0.55		
DMR17:87759001	17	87759001	87760000	1000	1	4.14E-05	-0.55868313	44	4.4	Arhgap21	
DMR17:87946001	17	87946001	87948000	2000	1	8.66E-05	0.67333664	25	1.25	Prtfdc1	Metabolism

DMR17:88851001	17	88851001	88852000	1000	1	5.29E-05	-1.02535532	6	0.6		
DMR18:1669001	18	1669001	1670000	1000	1	7.89E-05	-0.77330088	14	1.4		
DMR18:2221001	18	2221001	2222000	1000	1	8.51E-06	-0.39496236	7	0.7		
DMR18:2519001	18	2519001	2520000	1000	1	7.82E-05	0.49473692	7	0.7	AABR07031164.1	
DMR18:3430001	18	3430001	3431000	1000	1	8.35E-05	-0.4841771	21	2.1	Cables1	
DMR18:4282001	18	4282001	4283000	1000	1	1.83E-07	-0.5725699	17	1.7	Osbpl1a	Binding Protein
DMR18:4776001	18	4776001	4777000	1000	1	7.99E-06	0.9808632	11	1.1		
DMR18:5391001	18	5391001	5395000	4000	1	7.58E-05	0.9654309	43	1.075		
DMR18:8187001	18	8187001	8189000	2000	1	7.25E-05	-0.6472191	32	1.6	Cdh2	Extracellular Matrix
DMR18:9440001	18	9440001	9441000	1000	1	5.58E-05	-0.69117677	3	0.3	AABR07031328.1	
DMR18:10067001	18	10067001	10068000	1000	1	1.20E-05	0.88815413	9	0.9		
DMR18:10635001	18	10635001	10636000	1000	1	8.53E-05	1.05301307	1	0.1		
DMR18:11503001	18	11503001	11504000	1000	1	4.67E-05	0.46004912	4	0.4		
DMR18:15272001	18	15272001	15273000	1000	1	3.24E-05	0.58492238	4	0.4	7SK;LOC498826	
DMR18:18452001	18	18452001	18453000	1000	1	8.76E-05	-0.38890755	6	0.6		
DMR18:20928001	18	20928001	20929000	1000	1	9.50E-05	0.87336066	4	0.4		
DMR18:21691001	18	21691001	21693000	2000	1	4.62E-06	-0.64909473	24	1.2		
DMR18:23442001	18	23442001	23444000	2000	1	5.12E-05	0.46964861	2	0.1		
DMR18:24409001	18	24409001	24413000	4000	1	7.32E-05	0.36793706	23	0.575	Sap130	Transcription
DMR18:24455001	18	24455001	24456000	1000	1	7.60E-06	-0.58403527	9	0.9	Sap130	Transcription
DMR18:26045001	18	26045001	26047000	2000	1	1.26E-05	-0.81290672	10	0.5		
DMR18:29265001	18	29265001	29266000	1000	1	5.09E-05	0.88551965	12	1.2	Pfdn1	Translation
DMR18:31953001	18	31953001	31954000	1000	1	4.37E-05	0.5988859	14	1.4	Arhgap26	Signaling
DMR18:32310001	18	32310001	32311000	1000	1	8.10E-06	0.4575038	4	0.4	Fgf1	Growth Factors & Cytokines
DMR18:37949001	18	37949001	37950000	1000	1	4.13E-06	0.87908676	8	0.8	Jakmip2	
DMR18:41233001	18	41233001	41235000	2000	1	1.26E-05	-0.49161554	50	2.5		
DMR18:42791001	18	42791001	42792000	1000	1	9.15E-05	0.9052238	8	0.8		
DMR18:44759001	18	44759001	44760000	1000	1	3.46E-05	0.57946823	6	0.6	Tnfaip8	Unknown
DMR18:45141001	18	45141001	45142000	1000	1	8.98E-05	-0.50999969	9	0.9		
DMR18:47534001	18	47534001	47535000	1000	1	9.93E-06	-0.3872278	83	8.3	Lox	Metabolism
DMR18:48464001	18	48464001	48465000	1000	1	4.83E-05	-0.68190111	12	1.2	Prdm6	Transcription
DMR18:49392001	18	49392001	49393000	1000	1	5.42E-05	-0.89841303	20	2		
DMR18:49818001	18	49818001	49819000	1000	1	4.12E-05	1.16638274	6	0.6		
DMR18:50862001	18	50862001	50863000	1000	1	3.80E-05	-0.77176969	4	0.4		
DMR18:51436001	18	51436001	51437000	1000	1	3.01E-05	-0.59166106	27	2.7		
DMR18:51620001	18	51620001	51621000	1000	1	6.80E-05	-0.60822982	14	1.4	LOC688754;Aldh7a1	Metabolism
DMR18:55148001	18	55148001	55149000	1000	1	1.58E-05	-0.41078199	8	0.8		
DMR18:57176001	18	57176001	57178000	2000	1	2.17E-05	0.38102125	4	0.2	Ablim3	Cytoskeleton
DMR18:57231001	18	57231001	57232000	1000	1	4.73E-05	0.43306671	23	2.3	Ablim3	Cytoskeleton
DMR18:57540001	18	57540001	57542000	2000	1	4.79E-06	0.45504501	9	0.45	AABR07032261.3;AABR07032261.4	
DMR18:58109001	18	58109001	58111000	2000	1	3.97E-06	0.42402561	21	1.05		
DMR18:59332001	18	59332001	59333000	1000	1	9.86E-05	-0.40385714	15	1.5	Wdr7;U6;AABR07032277.3	Unknown
DMR18:59818001	18	59818001	59819000	1000	1	9.61E-05	-0.43296339	21	2.1	AABR07032289.1;RGD1562699	
DMR18:60565001	18	60565001	60566000	1000	1	2.09E-05	-0.44864634	22	2.2	Nedd4l	Protease
DMR18:62409001	18	62409001	62411000	2000	1	1.93E-05	-0.46840411	43	2.15	7SK	
DMR18:62510001	18	62510001	62511000	1000	1	7.04E-05	0.9933934	15	1.5		
DMR18:63103001	18	63103001	63104000	1000	1	2.34E-05	0.44109292	7	0.7	Cidea	Unknown
DMR18:63937001	18	63937001	63938000	1000	1	6.56E-05	-0.61081162	30	3	Ldlrad4	Receptor
DMR18:64415001	18	64415001	64416000	1000	1	9.43E-05	-0.58041855	11	1.1		
DMR18:65265001	18	65265001	65266000	1000	1	6.93E-05	-0.47507306	15	1.5	Tcf4	Transcription
DMR18:68032001	18	68032001	68034000	2000	1	3.14E-05	0.51634637	15	0.75		
DMR18:69972001	18	69972001	69973000	1000	1	8.14E-05	-0.59327427	22	2.2		
DMR18:70937001	18	70937001	70938000	1000	1	7.18E-05	0.45330188	4	0.4		
DMR18:72734001	18	72734001	72735000	1000	1	1.64E-06	-0.597537	18	1.8		
DMR18:72776001	18	72776001	72777000	1000	1	3.66E-05	-0.49902534	22	2.2		
DMR18:73115001	18	73115001	73116000	1000	1	5.07E-05	0.82192155	14	1.4		
DMR18:73646001	18	73646001	73647000	1000	1	3.48E-06	-0.64777821	35	3.5	Loxhd1	
DMR18:73690001	18	73690001	73692000	2000	1	6.17E-06	0.64449024	28	1.4	Loxhd1	
DMR18:73766001	18	73766001	73767000	1000	1	2.03E-07	-0.77460688	20	2	Loxhd1	
DMR18:73783001	18	73783001	73784000	1000	1	2.25E-05	0.76236881	3	0.3	Loxhd1	
DMR18:74844001	18	74844001	74846000	2000	2	9.62E-06	0.49650135	11	0.55		
DMR18:76823001	18	76823001	76824000	1000	1	1.46E-06	1.3994762	12	1.2	Kcng2	Transport
DMR18:77675001	18	77675001	77677000	2000	1	5.52E-07	0.84384336	7	0.35		
DMR18:78169001	18	78169001	78170000	1000	1	4.51E-06	-0.63466821	10	1		
DMR18:78305001	18	78305001	78306000	1000	1	7.06E-05	-0.58821946	17	1.7		
DMR18:79560001	18	79560001	79561000	1000	1	2.49E-05	-0.64438154	13	1.3		
DMR18:80218001	18	80218001	80219000	1000	1	1.02E-06	0.8605544	9	0.9	AABR07032758.2	
DMR18:80734001	18	80734001	80736000	2000	1	1.50E-06	-0.50904594	4	0.2		
DMR18:81197001	18	81197001	81199000	2000	1	1.09E-05	0.54318888	20	1	Zfp407	Transcription
DMR18:81383001	18	81383001	81385000	2000	1	2.96E-05	0.45082333	28	1.4	Zfp407	Transcription
DMR18:81619001	18	81619001	81620000	1000	1	4.28E-05	0.62288153	14	1.4		

DMR18:81863001	18	81863001	81864000	1000	1	3.63E-05	-0.45790667	21	2.1	Fbxo15	Transcription
DMR18:81904001	18	81904001	81906000	2000	1	6.92E-05	0.46852376	14	0.7		
DMR18:84086001	18	84086001	84087000	1000	1	1.31E-05	0.7969698	10	1		
DMR18:87127001	18	87127001	87129000	2000	1	5.05E-06	0.81419697	50	2.5		
DMR18:88082001	18	88082001	88084000	2000	1	6.76E-06	0.55406638	41	2.05		
DMR19:320001	19	320001	322000	2000	1	3.01E-05	0.50535389	15	0.75	AABR07042611.1	
DMR19:456001	19	456001	457000	1000	1	7.87E-05	0.82752431	6	0.6	LOC681364	EST
DMR19:1350001	19	1350001	1351000	1000	1	7.00E-05	0.55817104	4	0.4		
DMR19:3572001	19	3572001	3573000	1000	1	8.88E-07	0.73683703	6	0.6		
DMR19:6017001	19	6017001	6019000	2000	1	8.85E-05	0.7488495	21	1.05	AABR07042733.1;AABR07042733.2	
DMR19:6822001	19	6822001	6823000	1000	1	9.17E-05	-0.77806792	0	0		
DMR19:8261001	19	8261001	8262000	1000	1	4.30E-05	0.58619587	6	0.6	AABR07042780.1	
DMR19:8751001	19	8751001	8753000	2000	1	4.21E-05	0.59207579	9	0.45		
DMR19:10050001	19	10050001	10052000	2000	1	2.01E-06	0.44629929	20	1		
DMR19:10184001	19	10184001	10185000	1000	1	5.99E-05	-0.45579539	4	0.4	Tepp;Cngb1	Unknown;Receptor
DMR19:10686001	19	10686001	10688000	2000	1	6.57E-06	0.50881476	12	0.6	Ccl22	Signaling
DMR19:10982001	19	10982001	10983000	1000	1	1.29E-07	0.74681327	8	0.8	Nlrc5;AABR07042821.1	
DMR19:11081001	19	11081001	11082000	1000	1	1.10E-05	-0.8224902	23	2.3		
DMR19:11487001	19	11487001	11489000	2000	1	3.14E-06	-0.7460895	61	3.05	Amfr	Metabolism
DMR19:12829001	19	12829001	12830000	1000	1	3.08E-05	0.40415362	11	1.1	Large1	
DMR19:12843001	19	12843001	12846000	3000	1	1.01E-05	0.39689831	6	0.2	Large1	
DMR19:12848001	19	12848001	12849000	1000	1	3.07E-05	0.7691941	17	1.7	Large1	
DMR19:12935001	19	12935001	12936000	1000	1	2.50E-06	-0.51379519	21	2.1	Large1	
DMR19:15276001	19	15276001	15277000	1000	1	7.82E-06	-0.93971203	8	0.8	LOC501233	
DMR19:15384001	19	15384001	15385000	1000	1	6.65E-05	-0.48639602	18	1.8	Slc6a2	Metabolism
DMR19:16011001	19	16011001	16012000	1000	1	6.56E-05	-0.50084236	13	1.3		
DMR19:16372001	19	16372001	16374000	2000	1	5.72E-05	0.40355552	20	1		
DMR19:17893001	19	17893001	17894000	1000	1	3.34E-06	-0.50451646	22	2.2		
DMR19:19195001	19	19195001	19198000	3000	1	6.64E-06	-0.62681665	40	1.333		
DMR19:21183001	19	21183001	21185000	2000	1	9.09E-05	0.57906932	25	1.25		
DMR19:22010001	19	22010001	22011000	1000	1	6.44E-05	-0.52297955	22	2.2		
DMR19:22651001	19	22651001	22654000	3000	1	4.34E-05	0.54953751	7	0.233		
DMR19:23041001	19	23041001	23042000	1000	1	9.97E-05	0.4301114	11	1.1		
DMR19:24453001	19	24453001	24454000	1000	1	3.59E-05	0.73213207	20	2	Ucp1	Binding Protein
DMR19:24605001	19	24605001	24607000	2000	1	3.78E-05	0.430389	23	1.15	Scoc	Golgi
DMR19:25165001	19	25165001	25166000	1000	1	2.30E-05	-0.60808931	14	1.4	Il27ra;Rln3	Receptor
DMR19:25233001	19	25233001	25235000	2000	1	4.52E-06	0.47920118	10	0.5	Podnl1	Receptor
DMR19:25590001	19	25590001	25591000	1000	1	1.69E-05	-0.65618973	29	2.9	Cacna1a	Transport
DMR19:25757001	19	25757001	25759000	2000	1	4.47E-05	0.7470422	32	1.6	Cacna1a	Transport
DMR19:26931001	19	26931001	26932000	1000	1	8.44E-05	0.60671091	10	1		
DMR19:27671001	19	27671001	27675000	4000	1	1.50E-05	0.81222597	22	0.55	AABR07043354.1	
DMR19:28233001	19	28233001	28234000	1000	1	7.05E-05	-0.32070134	2	0.2	AABR07043421.1	
DMR19:28982001	19	28982001	28983000	1000	1	5.64E-05	0.72626785	19	1.9	AABR07043510.1	
DMR19:29141001	19	29141001	29143000	2000	1	2.75E-07	0.7467219	21	1.05	AABR07043523.1;AABR07043525.1	
DMR19:29245001	19	29245001	29248000	3000	1	2.68E-06	0.87747992	18	0.6	AABR07043564.1	
DMR19:29256001	19	29256001	29259000	3000	1	9.31E-06	0.61467694	23	0.767	AABR07043564.1	
DMR19:29324001	19	29324001	29326000	2000	1	2.60E-07	1.23308453	22	1.1	AABR07043564.1;AABR07043557.1	
DMR19:32400001	19	32400001	32401000	1000	1	4.01E-07	-0.64912878	26	2.6	Zfp827	Transcription
DMR19:35457001	19	35457001	35458000	1000	1	2.57E-05	0.58792353	1	0.1		
DMR19:36959001	19	36959001	36960000	1000	1	1.86E-06	0.69521117	3	0.3		
DMR19:40064001	19	40064001	40066000	2000	1	5.57E-05	0.85055405	24	1.2		
DMR19:41107001	19	41107001	41108000	1000	1	8.38E-07	-0.75704494	16	1.6	Hydin	Unknown
DMR19:44724001	19	44724001	44725000	1000	1	7.70E-06	-0.5199608	15	1.5		
DMR19:46136001	19	46136001	46137000	1000	1	2.64E-05	0.48692934	7	0.7		
DMR19:47013001	19	47013001	47015000	2000	1	6.27E-06	-0.58518984	32	1.6		
DMR19:47703001	19	47703001	47704000	1000	1	8.47E-06	1.10482401	12	1.2		
DMR19:48933001	19	48933001	48934000	1000	1	2.34E-05	-0.3683201	8	0.8		
DMR19:49352001	19	49352001	49353000	1000	1	3.55E-05	-0.51001707	4	0.4	Cenpn	Cell Cycle
DMR19:49944001	19	49944001	49945000	1000	1	3.51E-05	-0.65724048	19	1.9	Cmip	Signaling
DMR19:50981001	19	50981001	50983000	2000	1	3.75E-05	-0.50015472	16	0.8	Cdh13	Extracellular Matrix
DMR19:51126001	19	51126001	51127000	1000	1	2.87E-06	-0.5172663	18	1.8	Cdh13	Extracellular Matrix
DMR19:51234001	19	51234001	51235000	1000	1	5.98E-05	0.77819318	15	1.5	Cdh13	Extracellular Matrix
DMR19:51820001	19	51820001	51821000	1000	1	7.42E-05	0.70832737	9	0.9	Cdh13	Extracellular Matrix
DMR19:52054001	19	52054001	52056000	2000	1	1.36E-05	0.43599375	4	0.2	Mlycd;AABR07044001.4;AABR07044001.2;AABR07044001.3	
DMR19:52295001	19	52295001	52297000	2000	1	1.37E-07	0.49319285	29	1.45		
DMR19:54574001	19	54574001	54576000	2000	1	1.12E-05	-0.65209493	81	4.05	Jph3	Cell Junction
DMR19:55325001	19	55325001	55326000	1000	1	4.34E-05	-0.58004971	37	3.7	Piezo1	

DMR19:55944001	19	55944001	55945000	1000	1	2.90E-05	-0.60431645	27	2.7	Cpne7;AC119635.2;Sult5a1	Development;Metabolism
DMR19:56116001	19	56116001	56118000	2000	1	7.13E-06	-0.61672856	47	2.35	Fanca	
DMR19:56411001	19	56411001	56412000	1000	1	6.11E-05	-0.61401768	35	3.5	AABR07044079.1	
DMR19:56732001	19	56732001	56734000	2000	1	3.71E-05	-0.4263037	82	4.1	Nup133;Abcb10	Transport
DMR19:57373001	19	57373001	57374000	1000	1	5.84E-05	0.43287933	16	1.6	Capn9	Protease
DMR19:57379001	19	57379001	57380000	1000	1	9.49E-05	-0.34810042	5	0.5	Capn9	Protease
DMR19:57921001	19	57921001	57924000	3000	1	5.94E-05	-0.75289921	60	2	Disc1	
DMR19:59359001	19	59359001	59360000	1000	1	3.58E-06	-0.53062657	38	3.8	Tarbp1	
DMR19:59685001	19	59685001	59687000	2000	1	8.70E-05	0.73824707	7	0.35	AABR07072667.1	
DMR19:59922001	19	59922001	59925000	3000	1	1.67E-05	0.83900226	85	2.833	AC125920.1	
DMR19:60329001	19	60329001	60330000	1000	1	5.30E-05	0.64903893	8	0.8	Pard3	Cell Junction
DMR19:61823001	19	61823001	61824000	1000	1	1.50E-05	0.75330871	8	0.8		
DMR20:295001	20	295001	296000	1000	1	6.44E-05	-0.57526501	13	1.3	Clic2	Metabolism
DMR20:2063001	20	2063001	2064000	1000	1	1.79E-09	0.78979406	3	0.3	AC108572.6;RT1-M6-1	Immune
DMR20:2599001	20	2599001	2601000	2000	1	2.24E-07	0.90642484	20	1	AC120486.10	
DMR20:4293001	20	4293001	4294000	1000	1	7.33E-05	0.78461534	15	1.5	AABR07044388.6;C4b;LOC103689965	Immune
DMR20:4913001	20	4913001	4914000	1000	1	1.03E-06	0.66375947	10	1	RT1-CE1;RT1-CE4;AABR07044408.1;AABR07044408.2	Immune
DMR20:5722001	20	5722001	5723000	1000	1	8.31E-06	-0.55125817	27	2.7	Uqcc2;AC141521.3	
DMR20:5897001	20	5897001	5898000	1000	1	9.90E-08	-0.60002647	25	2.5	Slc26a8	Metabolism
DMR20:6054001	20	6054001	6055000	1000	1	7.44E-05	-0.66837735	53	5.3	Brpf3	Transcription
DMR20:6756001	20	6756001	6757000	1000	1	8.66E-06	-0.3712315	7	0.7	Ppil1;Grm4	Immune;Receptor
DMR20:7774001	20	7774001	7775000	1000	1	8.39E-05	-0.49069616	36	3.6	Zfp523	Transcription
DMR20:8052001	20	8052001	8054000	2000	1	9.50E-05	-0.58935699	45	2.25		
DMR20:8280001	20	8280001	8282000	2000	1	2.36E-05	0.55672165	28	1.4	Tbc1d22b;Rnf8	Signaling;Proteolysis
DMR20:8496001	20	8496001	8497000	1000	1	7.26E-07	-0.79344078	25	2.5	AABR07044509.1	
DMR20:8529001	20	8529001	8530000	1000	1	3.29E-06	-0.74897866	20	2	Mdga1	
DMR20:8954001	20	8954001	8956000	2000	1	2.73E-05	-0.6154984	41	2.05	Btbd9	Unknown
DMR20:9109001	20	9109001	9111000	2000	1	1.20E-05	-0.53873311	43	2.15	Btbd9	Unknown
DMR20:9576001	20	9576001	9577000	1000	1	9.26E-05	0.37882101	4	0.4	Glp1r	Receptor
DMR20:10744001	20	10744001	10745000	1000	1	1.10E-05	-0.53143682	23	2.3		
DMR20:10766001	20	10766001	10767000	1000	1	3.20E-05	-0.56346416	23	2.3	Hsf2bp	
DMR20:12106001	20	12106001	12107000	1000	1	2.25E-05	1.14941002	9	0.9	Adarb1	Epigenetic
DMR20:12146001	20	12146001	12147000	1000	1	5.39E-05	-0.64710675	16	1.6		
DMR20:12239001	20	12239001	12240000	1000	1	4.24E-06	0.80142585	19	1.9	Col18a1	Extracellular Matrix
DMR20:12752001	20	12752001	12753000	1000	1	7.82E-05	0.63574492	2	0.2		
DMR20:13524001	20	13524001	13525000	1000	1	8.44E-05	0.57698202	5	0.5	Slc5a4b	
DMR20:13877001	20	13877001	13878000	1000	1	9.52E-06	-0.41457547	6	0.6	Cabin1	Signaling
DMR20:15805001	20	15805001	15806000	1000	1	2.74E-06	-0.53157721	15	1.5		
DMR20:15905001	20	15905001	15906000	1000	1	3.64E-05	0.75142507	9	0.9		
DMR20:16225001	20	16225001	16226000	1000	1	8.05E-05	0.35531055	4	0.4		
DMR20:16363001	20	16363001	16364000	1000	1	1.30E-05	-0.81420908	33	3.3		
DMR20:16542001	20	16542001	16543000	1000	1	5.55E-06	-0.86658816	9	0.9	AABR07044668.1	
DMR20:18875001	20	18875001	18876000	1000	1	1.88E-05	-0.46399447	24	2.4	Bicc1	Transcription
DMR20:19115001	20	19115001	19116000	1000	1	1.14E-06	0.50430433	11	1.1		
DMR20:19233001	20	19233001	19234000	1000	1	3.10E-05	-0.38594892	5	0.5		
DMR20:19259001	20	19259001	19260000	1000	1	8.75E-05	0.38825815	6	0.6		
DMR20:20346001	20	20346001	20349000	3000	1	1.22E-05	0.64299795	69	2.3	Ank3;AABR07044777.1	Cytoskeleton
DMR20:20502001	20	20502001	20503000	1000	1	1.25E-05	0.4885947	9	0.9		
DMR20:20875001	20	20875001	20877000	2000	1	1.61E-05	0.60781279	27	1.35		
DMR20:21365001	20	21365001	21367000	2000	1	2.31E-05	-0.63606097	51	2.55	Cabcoco1	
DMR20:21420001	20	21420001	21421000	1000	1	3.56E-06	0.41760096	18	1.8	Cabcoco1	
DMR20:21668001	20	21668001	21670000	2000	1	1.40E-05	-0.79675582	32	1.6	Arid5b;Tmem26	Transcription
DMR20:26179001	20	26179001	26180000	1000	1	7.15E-06	0.49589771	4	0.4		
DMR20:28392001	20	28392001	28394000	2000	1	1.88E-05	-0.51838369	55	2.75		
DMR20:30270001	20	30270001	30272000	2000	1	8.84E-06	-0.320633	32	1.6	AABR07044980.1	
DMR20:30373001	20	30373001	30374000	1000	1	5.36E-07	-0.61032353	22	2.2	Unc5b	Receptor
DMR20:30781001	20	30781001	30782000	1000	1	7.40E-05	-0.71970834	18	1.8	Tbata	
DMR20:30923001	20	30923001	30924000	1000	1	1.48E-05	-0.53174299	10	1	Prf1	
DMR20:31870001	20	31870001	31871000	1000	1	2.95E-07	-0.5924054	17	1.7		
DMR20:34538001	20	34538001	34540000	2000	1	7.95E-05	-0.32736634	106	5.3		
DMR20:34989001	20	34989001	34990000	1000	1	8.65E-05	0.70518247	14	1.4	Fam184a;AABR07045135.1	Unknown
DMR20:37028001	20	37028001	37029000	1000	1	8.40E-05	0.74880337	10	1		
DMR20:38145001	20	38145001	38146000	1000	1	7.19E-06	0.75607642	17	1.7	AABR07045217.1	
DMR20:42867001	20	42867001	42868000	1000	1	2.11E-06	0.68833032	10	1		
DMR20:45620001	20	45620001	45622000	2000	1	2.85E-06	0.77775027	20	1		
DMR20:45755001	20	45755001	45756000	1000	1	3.45E-05	0.36681686	9	0.9		
DMR20:47644001	20	47644001	47645000	1000	1	3.29E-05	0.54960962	6	0.6	Scml4	Transcription
DMR20:48626001	20	48626001	48627000	1000	1	3.47E-05	0.4181042	4	0.4	Mettl24	Epigenetic

DMR20:52438001	20	52438001	52439000	1000	1	4.01E-05	0.53775621	13	1.3		
DMR20:54854001	20	54854001	54855000	1000	1	8.26E-05	0.36266424	9	0.9		
DMRX:2212001	X	2212001	2222000	10000	1	1.42E-05	0.38926268	73	0.73		
DMRX:10578001	X	10578001	10579000	1000	1	7.13E-05	0.88265012	6	0.6	Usp9x	Proteolysis
DMRX:22446001	X	22446001	22448000	2000	2	4.44E-10	0.71719111	8	0.4	AABR07037489.1	
DMRX:30211001	X	30211001	30212000	1000	1	7.94E-05	0.52718317	13	1.3		
DMRX:32496001	X	32496001	32499000	3000	1	1.54E-05	0.4940486	35	1.167	RGD1565844	
DMRX:34701001	X	34701001	34702000	1000	1	4.27E-05	-0.95245207	18	1.8	AABR07037878.2	
DMRX:37825001	X	37825001	37827000	2000	1	3.88E-05	0.6665839	8	0.4	Sh3kbp1	Signaling
DMRX:45464001	X	45464001	45466000	2000	1	6.01E-05	0.78673288	15	0.75		
DMRX:50268001	X	50268001	50269000	1000	1	1.90E-05	0.70875739	6	0.6		
DMRX:53564001	X	53564001	53565000	1000	1	1.08E-06	0.65593744	3	0.3		
DMRX:57243001	X	57243001	57245000	2000	1	4.63E-07	0.60924151	16	0.8		
DMRX:57752001	X	57752001	57753000	1000	1	2.12E-05	-0.44925127	37	3.7		
DMRX:59519001	X	59519001	59520000	1000	1	8.83E-06	1.0351535	5	0.5	U6	
DMRX:65774001	X	65774001	65775000	1000	1	6.27E-05	0.51614559	1	0.1		
DMRX:78571001	X	78571001	78572000	1000	1	2.04E-05	-0.33868336	39	3.9	AABR07039498.1	
DMRX:86605001	X	86605001	86606000	1000	1	6.13E-05	0.88729803	10	1		
DMRX:86632001	X	86632001	86633000	1000	1	8.02E-07	-0.71906694	16	1.6		
DMRX:91535001	X	91535001	91538000	3000	1	2.12E-06	-0.53738165	37	1.233		
DMRX:92207001	X	92207001	92209000	2000	1	5.58E-05	0.82168748	43	2.15		
DMRX:94297001	X	94297001	94299000	2000	2	2.92E-05	0.84512532	12	0.6	U6	
DMRX:95364001	X	95364001	95366000	2000	1	1.46E-05	0.86805962	39	1.95		
DMRX:96556001	X	96556001	96557000	1000	1	9.00E-10	-0.65580052	46	4.6		
DMRX:96598001	X	96598001	96600000	2000	1	6.24E-07	-0.50484526	79	3.95		
DMRX:100517001	X	100517001	100518000	1000	1	4.05E-05	0.80374216	10	1		
DMRX:103640001	X	103640001	103641000	1000	1	1.69E-06	-0.49277354	32	3.2		
DMRX:110091001	X	110091001	110092000	1000	1	6.19E-05	0.59611272	9	0.9		
DMRX:111113001	X	111113001	111115000	2000	1	5.36E-05	0.57028758	12	0.6	Cldn2	Cytoskeleton
DMRX:112386001	X	112386001	112395000	9000	1	2.65E-05	0.32122944	74	0.822	AABR07040864.1	
DMRX:113722001	X	113722001	113723000	1000	1	5.55E-05	0.47076973	8	0.8		
DMRX:114532001	X	114532001	114534000	2000	2	1.24E-05	0.55765035	11	0.55		
DMRX:119571001	X	119571001	119572000	1000	1	5.41E-05	0.60288477	10	1		
DMRX:120591001	X	120591001	120592000	1000	1	3.66E-07	0.58352471	3	0.3		
DMRX:123041001	X	123041001	123042000	1000	1	3.60E-05	-0.8589713	32	3.2		
DMRX:130251001	X	130251001	130252000	1000	1	1.45E-05	0.40264817	3	0.3		
DMRX:131649001	X	131649001	131650000	1000	1	2.85E-05	0.78286664	6	0.6		
DMRX:131666001	X	131666001	131667000	1000	1	1.46E-06	-0.36135044	52	5.2		
DMRX:139528001	X	139528001	139529000	1000	1	8.29E-05	0.44677641	4	0.4		
DMRX:155840001	X	155840001	155848000	8000	1	1.57E-06	-0.88723928	279	3.487	Dkc1	Transcription
DMRX:155850001	X	155850001	155851000	1000	1	4.15E-05	-1.07110677	67	6.7	Dkc1	Transcription
DMRX:155852001	X	155852001	155857000	5000	3	8.84E-09	-1.09881614	207	4.14	Dkc1	Transcription
DMRX:155858001	X	155858001	155860000	2000	1	1.66E-05	-0.93396561	69	3.45	Dkc1	Transcription
DMRX:156154001	X	156154001	156155000	1000	1	5.74E-05	-1.0353938	48	4.8	LOC102552182	
DMRX:156160001	X	156160001	156163000	3000	2	3.95E-06	-0.86825027	42	1.4	LOC102552182	
DMRX:156166001	X	156166001	156168000	2000	1	9.66E-06	-0.77426393	59	2.95		
DMRX:156176001	X	156176001	156180000	4000	3	1.63E-10	-1.09896571	86	2.15		
DMRX:156184001	X	156184001	156187000	3000	1	1.52E-09	-1.1416141	44	1.467		
DMRX:156207001	X	156207001	156212000	5000	3	3.13E-06	-0.86793197	90	1.8	AC095267.1;Olr1768	
DMRX:156219001	X	156219001	156221000	2000	2	4.99E-08	-1.08221938	28	1.4	LOC690348;AC095267.1;Olr1768	
DMRX:157056001	X	157056001	157058000	2000	1	3.01E-05	-0.43457693	60	3		
DMRY:2378001	Y	2378001	2379000	1000	1	6.08E-06	0.45645206	3	0.3		
